# Borylated cyclobutanes *via* thermal [2 + 2]-cycloaddition†

**DOI:** 10.1039/d3sc06600b

**Published:** 2024-01-25

**Authors:** Kateryna Prysiazhniuk, Oleksandr Polishchuk, Stanislav Shulha, Kyrylo Gudzikevych, Oleksandr P. Datsenko, Vladimir Kubyshkin, Pavel K. Mykhailiuk

**Affiliations:** a Enamine Ltd Winston Churchill St. 78 02094 Kyiv Ukraine Pavel.Mykhailiuk@gmail.com https://www.mykhailiukchem.org

## Abstract

A one-step approach to borylated cyclobutanes from amides of carboxylic acids and vinyl boronates is elaborated. The reaction proceeds *via* the thermal [2 + 2]-cycloaddition of *in situ*-generated keteniminium salts.

## Graphical abstract

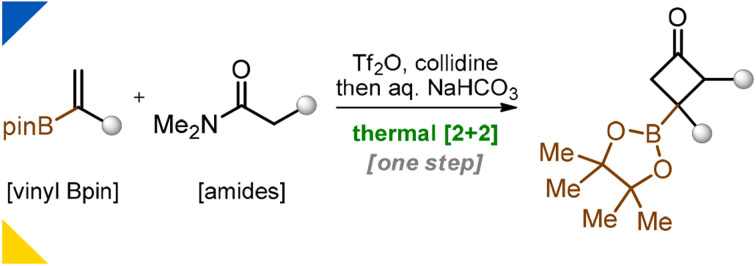

## Introduction

Small aliphatic rings attract considerable attention in contemporary research.^1^ For example, the cyclobutane ring is common within modern bioactive compounds^2^ and can be found in the structures of at least ten market-approved drugs.^3^ This motivated substantial development of cyclobutyl boronate chemistry during the past decade owing to the fact that the carbon–boron bond provides an excellent site for functionalization.^4^ The known approaches to the preparation of cyclobutyl boronates include the C–H activation of cyclobutanes,^5^ electrocyclization,^6^ functionalization of bicyclo[1.1.0]butanes,^7^ borylation^8^ and hydrogenation of cyclobutenes,^9^ along with other methods.^10,11^

**Scheme 1:**
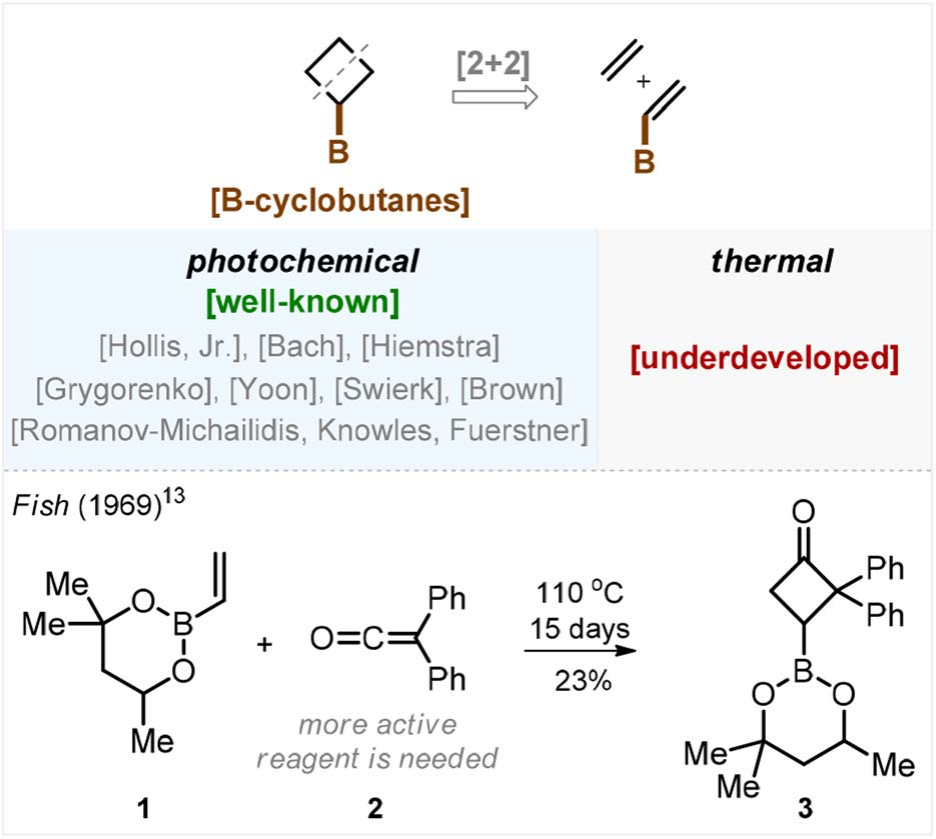
Retrosynthetic disconnection of borylated cyclobutanes *via* a [2 + 2]-reaction: photochemical *vs.* thermal strategies.

The most frequently used approach is a [2 + 2]-cycloaddition. Strikingly, while the photochemical version of this transformation has been elaborated in numerous studies by Hollis, Bach, Hiemstra, Grygorenko, Yoon, Swierk with Brown, Romanov-Michailidis with Knowles, and Fürstner,^12^ the thermal approach remained underdeveloped for unclear reasons (Scheme 1). We found only a single example in the literature on non-catalyzed thermal [2 + 2]-cycloaddition between alkene 1 and ketene 2 (Scheme 1). In 1969, Fish demonstrated that heating this mixture in a sealed vial for 15 days afforded the target cyclobutane 3 in 23% yield.^13^ Also, recently Brown showed an example of a thermal [2 + 2]-cycloaddition between a borylated alkene and an allene that required, however, Lewis acid catalysis.^14^

In this work, we developed a one-step approach to borylated cyclobutanes by thermal [2 + 2]-cycloaddition between vinyl boronates and *in situ*-generated keteniminium salts.

## Results and discussion

From the pioneering study of Fish (Scheme 1),^13^ it seemed reasonable to assume that vinyl boronates are amenable to non-catalytic thermal [2 + 2] cycloadditions; however, a more active partner than a ketene was needed. We turned our attention to keteniminium salts that are known to be more active than ketenes.^15^ Moreover, the [2 + 2]-cycloaddition of keteniminium salts with alkenes has been reported.^16,17^ Despite the substantial recent progress in keteniminium chemistry,^15^ we found no literature mentioning the reaction of borylated alkenes with keteniminium salts. Initially, we suspected that the conditions for their generation, which typically involved treatment with triflic anhydride,^17^ do not tolerate the Bpin group and the latter might decompose. Nonetheless, we decided to examine the feasibility of this transformation.

**Scheme 2:**
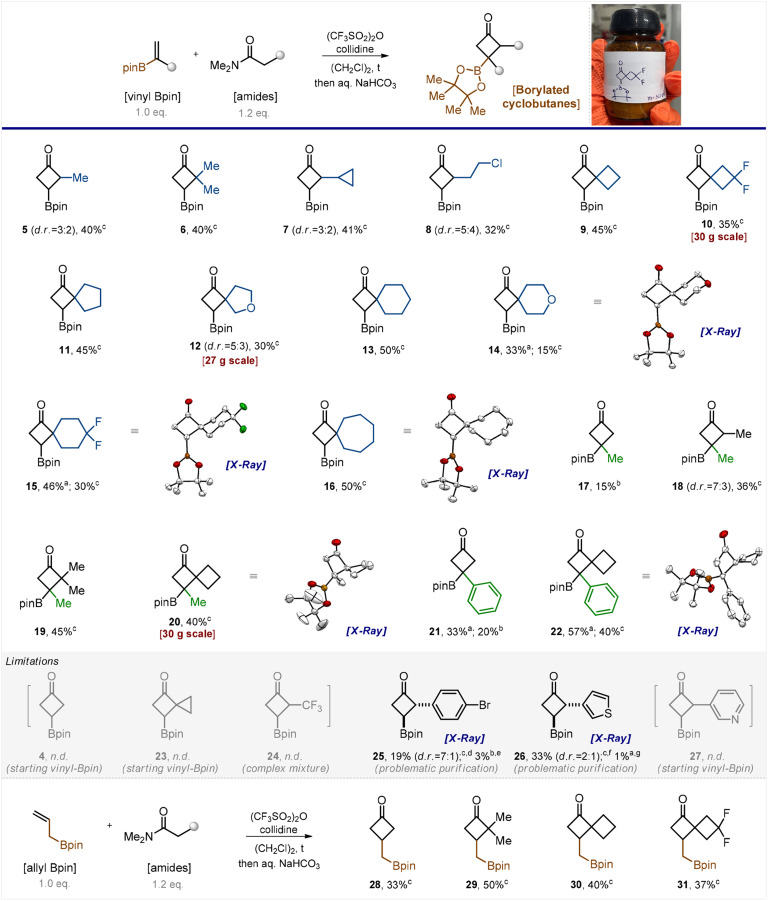
Reaction conditions: (i) vinyl Bpin/allyl Bpin (1.0 equiv.), amide (1.2 equiv.), triflic anhydride (1.4 equiv.), collidine or lutidine (1.4 equiv.), 1,2-dichloroethane, reflux, and 16 h; (ii) aqueous NaHCO_3_; (iii) purification (vacuum distillation or column chromatography). The scale of the synthesis: ^a^ 100–500 mg; ^b^ 1–7 g; ^c^ 10–50 g of the isolated product. ^d^ Product 25 (d.r. = 7 : 1) was obtained with *ca.* 70% purity. ^e^ Additional purification by column chromatography provided the pure product 25 as a single diastereomer in 3% yield. ^f^ Product 26 (d.r. = 2 : 1) was obtained with *ca.* 50% purity. ^g^ Additional purification by column chromatography provided the pure product 26 as a single diastereomer in 1% yield. X-ray crystal structures of compounds 14–16, 20, and 22 are shown as thermal ellipsoids at a 50% probability level; carbon – white, oxygen – red, boron – brown, and fluorine – green; hydrogen atoms are omitted for clarity.

The model reaction between *N*,*N*-dimethylacetamide and vinyl Bpin did not produce the desired product 4 even at trace amounts when performed in refluxed dichloroethane. Lowering the temperature to 60 °C or increasing the number of keteniminium equivalents from 1.2 to 3, 4, and 5 were as unsuccessful (Scheme 2, part limitations). Only the starting vinyl Bpin along with unidentified side products was detected in the reaction mixture. We were quite discouraged by the futility of our initial efforts, yet we attempted another reaction involving the homologous *N*,*N*-dimethylpropanamide. Serendipitously, the reaction worked out. Activation of the amide with (CF_3_SO_2_)_2_O/collidine (*in situ* formation of the keteniminium salt) followed by its reaction with vinyl Bpin produced the desired product 5 (Scheme 2).

After short optimization of the reaction conditions, we found that performing the reaction in refluxing dichloroethane for 12 hours produced excellent conversion of the starting vinyl boronate (see Table S1 in the ESI†). Thus, we examined the reaction scope by taking various amide counterparts and obtained borylated cyclobutanes 5–16 in decent yields (Scheme 2). The reaction was found compatible with the presence of the cyclopropyl ring (7), the active chlorine atom (8), and the *gem*-difluoro motif (10 and 15) in the product substances. Substituted alkenes, CH_2_=C(Me)-Bpin and CH_2_=C(Ph)-Bpin, gave the desired borylated cyclobutanes 17–22 as well. Products 5, 7, 8, 12, and 18 were obtained as inseparable mixtures of two diastereomers. The structure of products 14–16, 20 and 22 was confirmed by X-ray crystallographic analysis.^18^

The developed reaction showed few limitations, however. The keteniminium salt obtained from *N*,*N*-dimethylacetamide reacted with substituted vinyl boronates (products 17 and 21) but failed to react with vinyl Bpin (4). Attempts towards the synthesis of compounds 23, 24 and 27 failed as well. Analysis of the reaction mixture revealed either the presence of unreacted vinyl Bpin (23, 27) or the formation of a complex mixture (24). Some products, such as 25 and 26, were obtained in low isolated yields because they required rather tedious purification. The purification led to isolation of a single diastereomer in each case, and the *trans*-configuration of compounds 25 and 26 was revealed by X-ray crystallography.^18^

The analogous reaction between amides of carboxylic acids and the homologous CH_2_=CH–CH_2_-Bpin also produced the desired products 28–31 (Scheme 2).

It is important to note that the reaction demonstrated good performance on milligram, gram, and even multigram scales (10, 12, and 20). When carrying out the reaction on a small scale, we purified products by silica gel column chromatography. On a gram-to-multigram scale, we isolated the products by distillation under reduced pressure, which is more practical. Despite the seeming simplicity of the current approach to borylated cyclobutanes, to the best of our knowledge, none of the obtained products depicted in Scheme 2 has been reported in the literature.

**Scheme 3:**
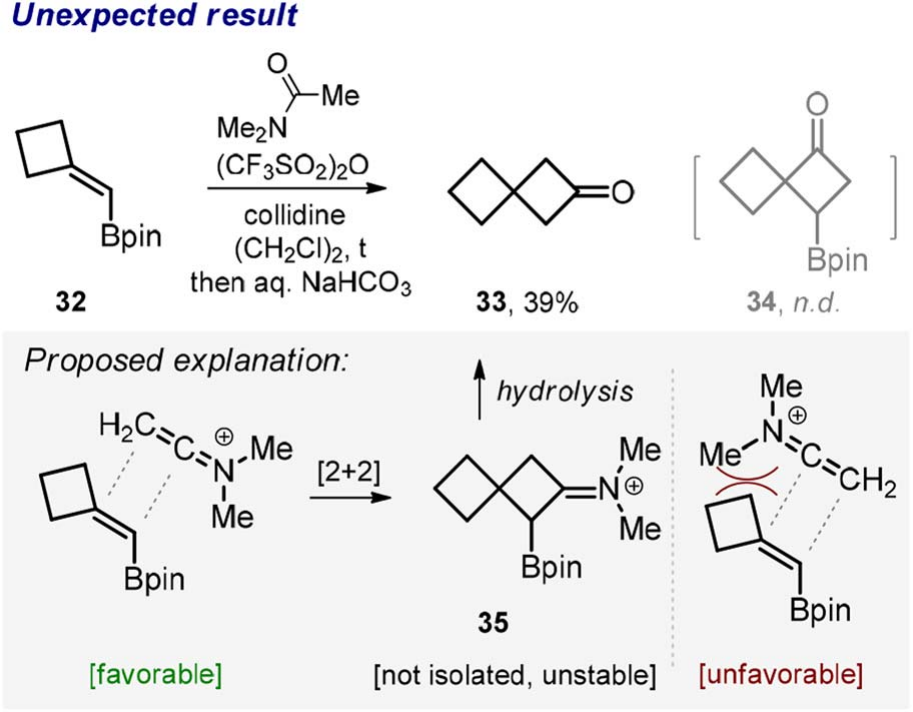
Unexpected synthesis of ketone 33.

Somewhat unexpectedly, the reaction between β,β-disubstituted vinyl boronate 32 and *N*,*N*-dimethylacetamide produced ketone 33 rather than the borylated cyclobutane 34 (Scheme 3). While we did not examine the exact mechanism of this transformation,^19^ we found a fairly reasonable explanation for the observed outcome. We reasoned that the bulky C=NMe_2_ moiety approached the tertiary rather than quaternary carbon of the amide in the course of the cycloaddition probably due to steric reasons (Scheme 3, proposed explanation). Effectively, this steered the reaction towards the formation of the intermediate compound 35, which is related to the class of unstable α-borylated ketones^20^ prone to hydrolytic protodeborylation, thus producing ketone 33.

**Scheme 4:**
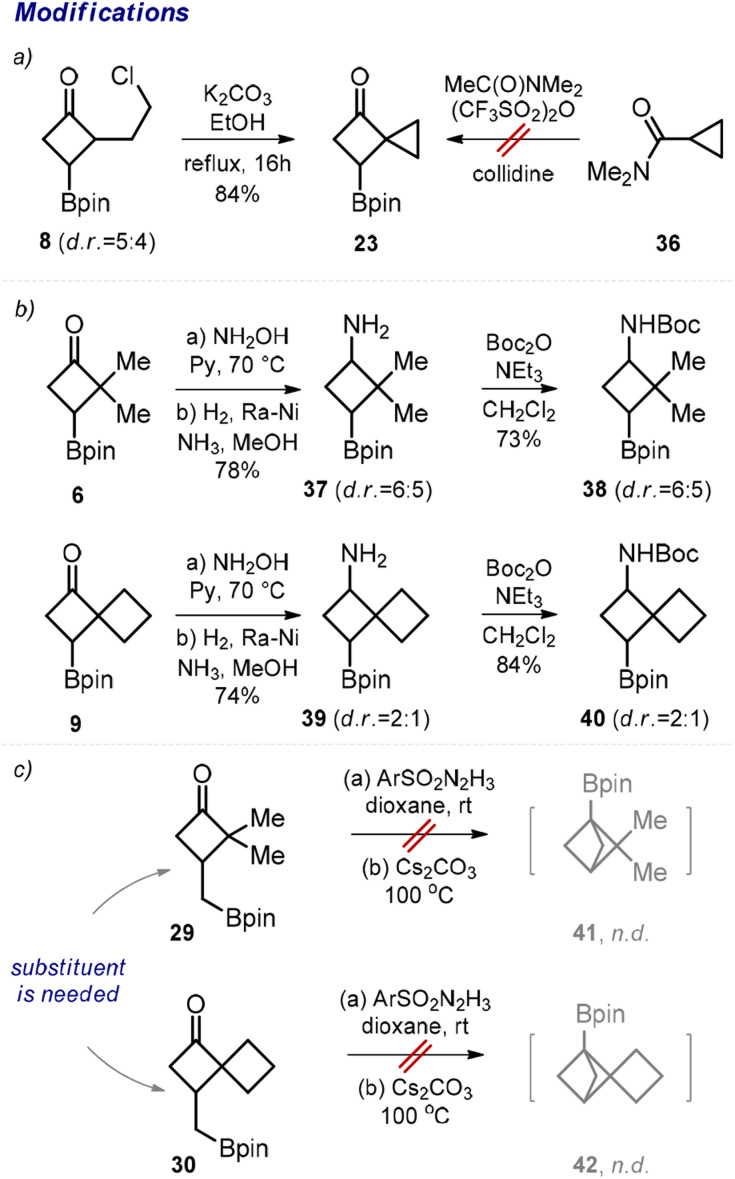
Modifications of borylated cyclobutanes. (a) Synthesis of borylated cyclobutane 23. (b) Synthesis of *N*-Boc amino boronates 38 and 40. (c) An attempted synthesis of bicyclo[1.1.1]pentanes 41 and 42.

Next, we performed transformations of the obtained products. For example, despite the failed attempt to direct synthesis of cyclobutane 23 from amide 36 (Scheme 2, limitations), we were able to obtain compound 23 by an intramolecular cyclization of the previously synthesized chloride 8 in 84% yield (Scheme 4a). From ketone 6, the corresponding amino boronate 37 was synthesized in two steps (Scheme 4b). The subsequent *N*-protection provided *N*-Boc amino boronate 38, which represents a useful medicinal chemistry precursor. Analogously, the *N*-Boc amino boronate 40 was obtained from ketone 9*via* amine 39.

In 2021, Qin and colleagues developed an elegant intramolecular coupling towards multi-substituted bicycloalkyl boronates.^11*a*^ The corresponding starting materials were synthesized in multiple steps. In this work, we obtained ketones 29 and 30 in one step, and we also attempted their cyclization into the desired bicyclo[1.1.1]pentanes 41 and 42 (Scheme 4c). Unfortunately, under the original conditions reported by the Qin group, the formation of the desired products was not observed. Our unsuccessful efforts corroborate the seminal conclusion that the presence of an additional substituent at the cyclobutane ring is crucial for the formation of bicyclo[1.1.1]pentane in this reaction.^11*a*^

**Scheme 5:**
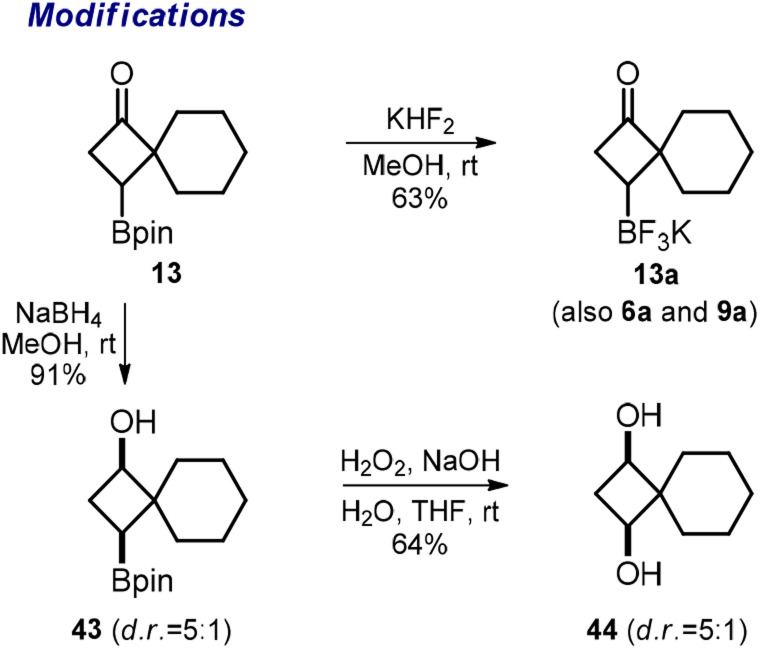
Synthesis of potassium trifluoroborate salts 6a, 9a, and 13; and diol 44.

Finally, we performed a few other representative modifications. The reaction of pinacol boronates 6, 9, and 13 with KHF_2_ produced potassium trifluoroborates 6a, 9a, and 13a (Scheme 5). The reduction of the ketone group in 13 with NaBH_4_ gave alcohol 43. The oxidative cleavage of the Bpin group in the latter substance gave diol 44. We assume that similar modifications could be performed with the other obtained Bpin ketones following our protocols.

## Conclusions

Here, we elaborated a thermal [2 + 2]-cycloaddition between vinyl boronates and *in situ* generated keteniminium salts. This practical approach allows the preparation of borylated cyclobutanes in one step. The obtained compounds can be used in the syntheses of various functionalized cyclobutanes.

## Data availability

The ESI† contains method description, product characterization data, and NMR spectra.

## Author contributions

O. P. D. and P. K. M. designed the project. K. P., O. P., S. S., K. G., and O. P. D. carried out experiments. V. K. analysed the data. V. K. and P. K. M. wrote the manuscript and all authors provided comments.

## Conflicts of interest

The authors are employees of a chemical supplier Enamine.

## Supplementary Material

SC-015-D3SC06600B-s001

SC-015-D3SC06600B-s002

## References

[cit1] Bauer M. R., Di Fruscia P., Lucas S. C. C., Michaelides I. N., Nelson J. E., Storer R. I., Whitehurst B. C. (2021). Put a ring on it: application of small aliphatic rings in medicinal chemistry. RSC Med. Chem..

[cit2] van der Kolk M. R., Janssen M. A. C. H., Rutjes F. P. J. T., Blanco-Ania D. (2022). Cyclobutanes in Small-Molecule Drug Candidates. ChemMedChem.

[cit3] Chemical structure search drugbank online, https://go.drugbank.com/structures/search/small_molecule_drugs/structure, accessed December 2023, filters used: “substucture”+“approved”+”vet approved”

[cit4] Armstrong R. J., Aggarwal V. (2017). 50 Years of Zweifel Olefination: a Transition-Metal-Free Coupling. Synthesis.

[cit5] He J., Shao Q., Wu Q., Yu J.-Q. (2017). Pd(II)-Catalyzed Enantioselective C(sp^3^)-H Borylation. J. Am. Chem. Soc..

[cit6] Giustra Z. X., Yang X., Chen M., Bettinger H. F., Liu S.-Y. (2019). Accessing 1,2-Substituted Cyclobutanes Through 1,2-Azaborine Photoisomerization. Angew. Chem., Int. Ed..

[cit7] Silvi M., Aggarwal V. K. (2019). Radical Addition to Strained σ-Bonds Enables the Stereocontrolled Synthesis of Cyclobutyl Boronic Esters. J. Am. Chem. Soc..

[cit8] Brener L., Brown H. C. (1977). Hydroboration. 47. Unique Stereospecificity of the Hydroboration of 1, 3-Dimethylcycloalkenes with 9-Borabicyclo [3.3.1] Nonane. J. Org. Chem..

[cit9] Parsutkar M. M., Pagar V. V., RajanBabu T. V. (2019). Catalytic Enantioselective Synthesis of Cyclobutenes From Alkynes and Alkenyl Derivatives. J. Am. Chem. Soc..

[cit10] Hari D. P., Abell J. C., Fasano V., Aggarwal V. K. (2020). Ring-Expansion Induced 1,2-Metalate Rearrangements: Highly Diastereoselective Synthesis of Cyclobutyl Boronic Esters. J. Am. Chem. Soc..

[cit11] Yang Y., Tsien J., Hughes J. M. E., Peters B. K., Merchant R. R., Qin T. (2021). An intramolecular coupling approach to alkyl bioisosteres for the synthesis of multisubstituted bicycloalkyl boronates. Nat. Chem..

[cit12] Hollis Jr. W. G., Lappenbusch W. C., Everberg K. A., Woleben C. M. (1993). The Use of Alkenylboronate Esters in [2 + 2] Enone-Olefin Photocycloadditions. Tetrahedron Lett..

[cit13] Fish R. H. (1969). The Cycloaddition of Diphenylketene to 2-Vinyl-4,6,6-trimethyl-1,3,2-dioxaborinane. J. Org. Chem..

[cit14] Conner M. L., Brown M. K. (2016). Synthesis of 1,3-Substituted Cyclobutanes by Allenoate-Alkene [2 + 2] Cycloaddition. J. Org. Chem..

[cit15] Madelaine C., Valerio V., Maulide N. (2011). Revisiting Keteniminium Salts: More than the Nitrogen Analogs of Ketenes. Chem.–Asian J..

[cit16] Marchand-Brynaert J., Ghosez L. (1972). Cycloadditions of Keteneimmonium Cations to Olefins and Dienes. A new Synthesis of Four-Membered Rings. J. Am. Chem. Soc..

[cit17] Falmagne J.-B., Escudero J., Taleb-Sahraoui S., Ghosez L. (1981). Cyclobutanone and Cyclobutenone Derivatives by Reaction of Tertiary Amides with Alkenes or Alkynes. Angew. Chem., Int. Ed..

[cit18] Cambridge Crystallographic Data Centre (CCDC) deposition numbers: 2312008 (14), 2312007 (15), 2312010 (16), 2312009 (20), 2312011 (22), 2321052 (25), and 2321392 (26)

[cit19] Saimoto H., Houge C., Hesbain-Frisque A.-M., Mockel A., Ghosez L. (1983). Nonstereospecificity in the cycloadditions of keteneiminium salts to olefins. Evidence for a stepwise mechanism. Tetrahedron Lett..

[cit20] He Z., Zajdlik A., Yudin A. K. (2014). α-Borylcarbonyl compounds: from transient intermediates to robust building blocks. Dalton Trans..

