# Supplementary material for: Borylated cyclobutanes via thermal [2 + 2]-cycloaddition
Source: Chem Sci. 2024 Jan 25;15(9):3249–54. doi: 10.1039/d3sc06600b (PMC10901489; doi:10.1039/d3sc06600b)
Supplement: SC-015-D3SC06600B-s001 [file SC-015-D3SC06600B-s001.pdf]

Electronic Supplementary Material (ESI) for Chemical Science.
This journal is © The Royal Society of Chemistry 2024

Borylated Cyclobutanes *via* thermal [2+2]-cycloaddition

Kateryna Prysiazhniuk, Oleksandr Polishchuk, Stanislav Shulha, Kyrylo Gudzikevych, Oleksandr P. Datsenko, Vladimir Kubyshkin, Pavel K. Mykhailiuk*

Enamine Ltd.; Chervonotkatska 60, 02094 Kyiv (Ukraine), www.enamine.net; www.mykhailiukchem.org
Corresponding author: Pavel.Mykhailiuk@gmail.com

Supplementary Information

Table of Contents

General information S2
1. Starting materials S2
2. Thermal [2+2]-cycloaddition of vinyl boronates S7
   Optimization of the reaction conditions S7
3. Derivative compounds S17
4. Checkcif for compound X-ray crystal structures S23
5. Copies of the NMR spectra S30
   Starting materials S30
   [2+2] cycloaddition products S69
   $^{1}H\{^{13}C\}$ HSQC multiplicity edited for compound 8 .... S77
   Derivative compounds S121
6. Supporting references S146

General information

Chemicals were obtained from commercial sources and used without additional purification. Vinil and allyl boronic acids were commercial substances. *N*,*N*-Dimethylamides were purchased or prepared from commercial acid chloroanhydrides as described below. Reaction solvents were anhydrous, other solvents were of standard commercial grade. NMR spectra were recorded at 400, 500 and 600 MHz ^{1}H frequencies, 126 and 151 MHz ^{13}C frequencies, and 376 MHz ^{19}F frequency. ^{13}C and ^{19}F NMR spectra were recorded with ^{1}H decoupling during acquisition. Spectra were calibrated using conventional deuterium lock internal referencing. The melting points were not corrected. High resolution mass spectra (HRMS) were recorded using electrospray ionization– time of flight setup.

1. Starting materials

Starting *N*,*N*-dimethylamides were prepared from acid chloroanhydrides using a general procedure. Amides **a1-a16** were prepared with dimethylamine, while amide **a17**-**a18** were prepared with pyrrolidine.

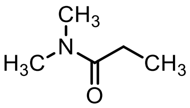
a1

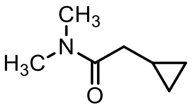
a2

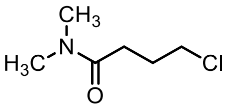
a3

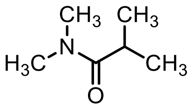
a4

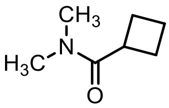
a5

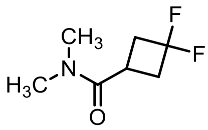
a6

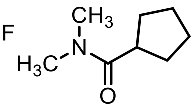
a7

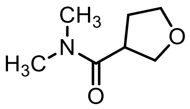
a8

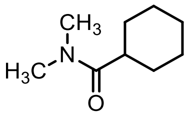
a9

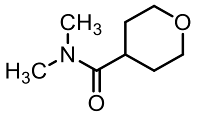
a10

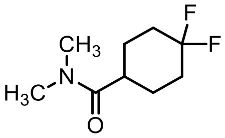
a11

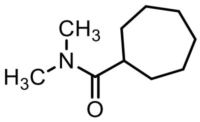
a12

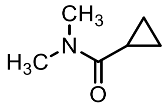
a13

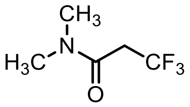
a14

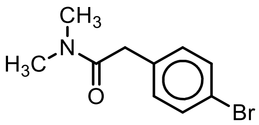
a15

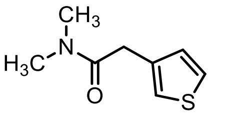
a16

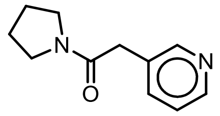
a17

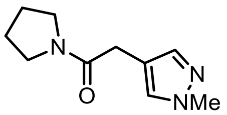
a18

General procedure A for preparation of amides **a1**-**a18** (amide **a4**):

***N*,*N*-dimethylisobutyramide (a4)**

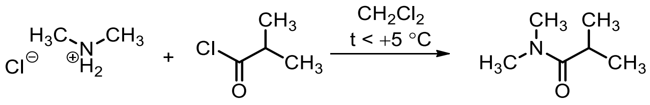

Dichloromethane (2 L) was added to dimethylamine hydrochloride (149.2 g, 1.83 mol, 1.2 equiv.), the mixture was cooled down to −10 °C, and triethylamine (490 mL, 3.52 mol, 2.5 equiv.) was added. Isobutyryl chloride (147 mL, 1.40 mol, 1 equiv.) was added dropwise within 1 hour, while the temperature was maintained below +5 °C. The reaction mixture was stirred at the room temperature for the next 16 hours. The mixture was filtered, the filtrate was washed with water (2×500 mL), brine (1×800 mL), and dried over sodium sulphate. The solvent was removed under

reduced pressure, the target compound was purified by vacuum distillation at 20 Torr (67 °C fraction) to yield amide **a4** (127 g, 1.10 mol, 79%) as a colorless liquid.

^{1}H NMR ($CDCl_3$, 500 MHz), δ: 3.03 (broad s, 3H), 2.95 (broad s, 3H), 2.81 (hept., *J* = 6.7 Hz, 1H), 1.11 (d, *J* = 6.7 Hz, 6H). ^{13}C{^{1}H} NMR ($CDCl_3$, 151 MHz), δ: 177.0, 36.7 (broad), 35.6 (broad), 30.2, 19.2. HRMS: calculated m/z for $[M+H]^+$ $C_6H_{14}NO^+$ 116.1070, found 116.1067.

N,N-Dimethylpropionamide (a1)

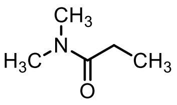

Obtained using **general procedure A**, yield 70 g, 80%, colorless liquid, b.p.= 74 °C (20 Torr). ^{1}H NMR ($CDCl_3$, 500 MHz), δ: 2.97 (broad s, 3H), 2.91 (broad s, 3H), 2.30 (q, *J* = 7.5 Hz, 2H), 1.11 (t, *J* = 7.5 Hz, 3H). ^{13}C{^{1}H} NMR ($CDCl_3$, 126 MHz), δ: 173.8, 37.1, 35.5, 26.5, 9.3. HRMS: calculated m/z for $[M+H]^+$ $C_5H_{12}NO^+$ 102.0914, found 102.0916.

2-Cyclopropyl-*N,N*-dimethylacetamide (a2)

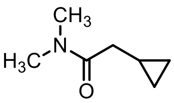

Obtained using general **procedure A**, yield 60 g, 80%, colorless liquid, b.p.= 40 °C (1 Torr). ^{1}H NMR ($CDCl_3$, 500 MHz), δ: 2.96 (s, 6H), 2.26 (d, J = 6.8 Hz, 2H), 1.06 (m, 1H), 0.54 (m, 2H), 0.15 (m, 2H). ^{13}C{^{1}H} NMR ($CDCl_3$, 126 MHz), δ: HRMS: calculated m/z for $[M+H]^+$ $C_7H_{14}NO^+$ 128.10.70, found 128.1069.The NMR data are consistent with the previously reported in the literature.[S1,S2]

4-Chloro-*N,N*-dimethylbutanamide (a3)

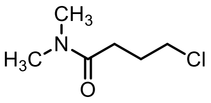

Obtained using **general procedure A**, yield 90 g, 70%, yellow liquid, b.p. = 55 °C (1 Torr). ^{1}H NMR (DMSO-d_6, 400 MHz), δ: 3.62 (t, *J* = 6.5 Hz, 2H), 3.01 (s, 3H), 2.86 (s, 3H), 2.43 (t, *J* = 6.9 Hz, 2H), 1.99 (quint., *J* = 6.6 Hz, 2H). ^{13}C{^{1}H} NMR (DMSO-d_6+CCl_4, 126 MHz), δ: 170.2, 44.5, 36.5, 34.7, 29.2, 27.6. HRMS: calculated m/z for $[M+H]^+$ $C_6H_{13}ClNO^+$ 150.0681, found 150.0677.

N,N-Dimethylcyclobutanecarboxamide (a5)

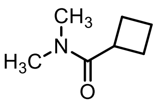

Obtained using general **procedure A** yield 130 g, 81%, yellow liquid, b.p. = 55 °C (1 Torr). ^{1}H NMR ($CDCl_3$, 500 MHz), δ: 3.24 (quint. *J* = 8.6 Hz, 1H), 2.89 (s, 6H), 2.30 (m, 2H), 2.12 (m, 2H), 1.92 (m, 1H), 1.82 (m, 1H). ^{13}C{^{1}H} NMR ($CDCl_3$, 126 MHz), δ: 174.0, 52.9, 36.9, 35.9 (broad), 34.9 (broad), 24.5, 17.3. HRMS: calculated m/z for $[M+H]^+$ C7H14NO+ 128.1070, found 128.1070. The NMR data are consistent with the previously reported in the literature.[S2]

3,3-Difluoro-*N,N*-dimethylcyclobutanecarboxamide (a6)

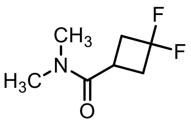

Obtained using general **procedure A** yield 70 g, 75%, yellow liquid, b.p. = 40 °C (1 Torr). ^{1}H NMR ($CDCl_3$, 500 MHz), δ: 3.07 (quint.d, *J* = 8.6, 2.1 Hz, 1H), 2.96 (s, 6H), 2.89 (m, 2H), 2.71 (m, 2H). ^{13}C{^{1}H} NMR ($CDCl_3$, 126 MHz), δ: 171.7 (t, *J* = 2 Hz), 118.8 (dd, *J* = 286, 268 Hz), 38.7 (t, *J* = 25 Hz), 38.6 (t, *J* = 24 Hz), 36.7 (s), 35.7 (s), 25.5 (dd, *J* = 16, 4 Hz). ^{19}F{^{1}H} NMR ($CDCl_3$, 376 MHz): −82.7 (d, *J* = 193 Hz, 1F), −98.9 (d, *J* = 193 Hz, 1F). HRMS: calculated m/z for $[M+H]^+$ $C_7H_{12}F_2NO^+$ 164.0882, found 164.0881.

_N,N_-dimethylcyclopentanecarboxamide (a7)

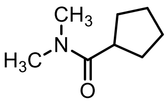

Obtained using **general procedure A**, yield 97 g, 79%, colorless liquid, b.p.= 74 °C (1 Torr). ^{1}H NMR ($CDCl_3$, 500 MHz), δ: 2.99 (broad s, 6H), 2.90 (quint. J = 7.8 Hz, 1H), 1.80 (m, 4H), 1.73 (m, 2H), 1.56 (m, 2H). ^{13}C{^{1}H} NMR ($CDCl_3$, 151 MHz), δ: 176.2, 41.2, 36.4 (broad), 30.0, 26.0. The NMR data is consistent with the literature.[S2] HRMS: calculated m/z for $[M+Na]^+$ $C_8H_{15}NNaO^+$ 164.1046, found 164.1044.

_N,N_-dimethyltetrahydrofuran-3-carboxamide (a8)

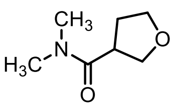

Obtained using **general procedure A**, yield 90 g, 75%, yellow liquid, b.p.= 70 °C (1 Torr). ^{1}H NMR ($CDCl_3$, 400 MHz), δ: 4.05 (t, *J* = 8.2 Hz, 1H), 3.88 (quint., *J* = 7.0 Hz, 3H), 3.27 (quint., *J* = 7.8 Hz, 1H), 3.03 (broad s, 6H), 2.22 (m, 1H), 2.10 (m, 1H). ^{13}C{^{1}H} NMR ($CDCl_3$, 126 MHz), δ: 172.3, 70.1 67.9, 40.9, 36.6 (broad), 35.2 (broad), 29.6. HRMS: calculated m/z for $[M+H]^+$ $C_7H_{14}NO_2^+$ 114.1020, found 144.1018.

_N,N_-dimethylcyclohexanecarboxamide (a9)

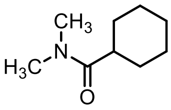

Obtained using **general procedure A**, yield 243 g, 91%, colorless liquid, b.p.= 81 °C (1 Torr). ^{1}H NMR ($CDCl_3$, 500 MHz), δ: 2.98 (broad s, 6H), 2.48 (tt, J = 11.6, 3.1 Hz), 1.79 (m, 2H), 1.70 (m, 3H), 1.51 (m, 2H), 1.26 (m, 3H). ^{13}C{^{1}H} NMR ($CDCl_3$, 151 MHz), δ: 176.1, 40.7, 37.0, 35.5, 29.1, 25.9, 25.8. The NMR data is consistent with the literature.[S3] HRMS: calculated m/z/ for $[M+H]^+$ $C_9H_{18}NO^+$ 156.1383, found 156.1381.

_N,N_-dimethyltetrahydro-2H-pyran-4-carboxamide (a10)

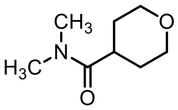

Obtained using **general procedure A**, yield 85 g, 85%, yellow oil, b.p.= 90 °C (1 Torr). ^{1}H NMR ($CDCl_3$, 500 MHz), δ: 3.99 (dm, *J* = 10.7 Hz, 2H), 3.42 (t, *J* = 11.7 Hz, 2H), 3.02 (broad s, 3H), 2.94 (broad s, 3H), 2.72 (tt, *J* = 11.3, 3.6 Hz, 1H), 1.86 (m, 2H), 1.57 (d, *J* = 13.1 Hz, 2H). ^{13}C{^{1}H} NMR ($CDCl_3$, 126 MHz), δ: 173.8, 66.6, 37.3, 36.5 (broad), 35.2 (broad), 28.3. The NMR data is consistent with the literature.[S4] HRMS: calculated m/z for $[M+H]^+$ $C_8H_{16}NO_2^+$ 158.1176, found 158.1172.

4,4-Difluoro-_N,N_-dimethylcyclohexanecarboxamide (a11)

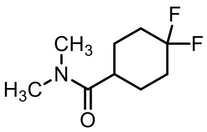

Obtained using **general procedure A**, yield 80°g, 70%, white crystalline solid, b.p.= 75 °C (1 Torr), m.p. 83 °C. ^{1}H NMR (DMSO-d_6, 400 MHz), δ: 3.02 (s, 3H), 2.80 (s, 3H), 2.77 (m, 1H), 2.02 (m, 2H), 1.91 (m, 1H), 1.82 (m, 1H), 1.70 (m, 2H), 1.56 (m, 2H). ^{13}C{^{1}H} NMR (DMSO-d_6, 126 MHz), δ: 173.5 (d, *J* = 2 Hz), 123.7 (dd, *J* = 242, 239 Hz), 36.6 (s), 36.5 (s), 34.9 (s), 32.2 (dd, *J* = 25, 23 Hz), 25.3 (d, *J* = 10 Hz). ^{19}F{^{1}H} NMR (DMSO-d_6, 376 MHz): −89.8 (d, *J* = 232 Hz, 1F), −100.0 (d, *J* = 232 Hz, 1F). HRMS: calculated m/z for $[M+H]^+$ $C_9H_{16}F_2NO^+$ 192.1195, found 192.1192.

_N,N_-Dimethylcycloheptanecarboxamide (a12)

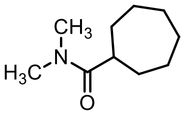

Obtained using **general procedure A**, yield 70 g, 85%, colorless liquid, b.p.= 90 °C (1 Torr). ^{1}H NMR ($CDCl_3$, 500 MHz), δ: 2.97 (broad s, 6H), 2.64 (tt, *J* = 9.8, 3.6 Hz, 1H), 1.78 (m, 4H), 1.68 (m, 2H), 1.57 (m, 4H), 1.44 (m, 2H). ^{13}C{^{1}H} NMR ($CDCl_3$, 126 MHz), δ: 176.8, 41.2, 36.6 (broad), 35.0 (broad), 30.6, 27.7, 26.2. The

NMR data is consistent with the literature.[S5] HRMS: calculated m/z for $[M+H]^+$ $C_{10}H_{20}NO^+$ 170.1540, found 170.1536.

***N*,*N*-dimethylcyclopropanecarboxamide (a13, 36)**

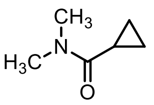

Obtained using **general procedure A**, yield 7 g, 70%, colorless liquid, b.p. = 80 °C (1 Torr). ^{1}H NMR ($CDCl_3$, 500 MHz), δ: 3.06 (broad s, 6H), 1.73 (tt, *J* = 8.0, 4.6 Hz, 1H), 0.95 (m, 2H), 0.74 (m, 2H). ^{13}C{^{1}H} NMR ($CDCl_3$, 126 MHz), δ: 172.9, 36.5 (broad), 35.4 (broad), 10.5, 6.8. The NMR data is consistent with the literature.[S2] HRMS: calculated m/z for $[M+H]^+$ $C_6H_{12}NO^+$ 114.0914, found 114.0913.

3,3,3-Trifluoro-*N*,*N*-dimethylpropanamide (a14)

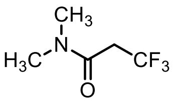

Obtained using **general procedure A**, yield 50 g, 75%, white crystalline solid, b.p. = 70 °C (100 Torr), m.p. 46 °C. ^{1}H NMR ($CDCl_3$, 500 MHz), δ: 3.22 (q, *J* = 10.5 Hz, 2H), 3.04 (s, 3H), 2.98 (s, 3H). ^{13}C{^{1}H} NMR ($CDCl_3$, 126 MHz), δ: 162.6 (q, *J* = 3 Hz), 123.6 (q, *J* = 277 Hz), 37.6 (q, *J* = 29 Hz), 37.2 (s), 35.1 (s). ^{19}F{^{1}H} NMR (DMSO-d_6, 376 MHz): −63.1 (s). The NMR data is consistent with the literature.[S6,S7] HRMS: calculated m/z for $[M+H]^+$ $C_5H_8F_3NO$ 156.0631, found 156.0631.

2-(4-Bromophenyl)-*N,N*-dimethylacetamide (a15)

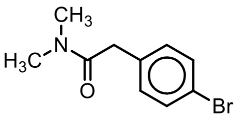

Obtained using general **procedure A** yield 96 g, 93%, yellow powder, m.p. 76 °C. The compound was used without purification.. ^{1}H NMR ($CDCl_3$, 500 MHz), δ: 7.43 (d, *J* = 8.3 Hz, 2H), 7.12 (d, *J* = 8.3 Hz, 2H), 3.65 (s, 2H), 2.98 and 2.97 (two s, 6H). ^{13}C{^{1}H} NMR ($CDCl_3$, 151 MHz), δ: 170.5, 134.0, 131.7, 130.6, 120.7, 40.2, 37.7 (broad), 36.7 (broad). The NMR data are consistent with the previously reported in the literature.[S8] HRMS: calculated m/z for $[M+H]^+$ $C_{10}H_{13}BrNO^+$ 242.0176 (^{79}Br) and 244.0155 (^{81}Br), found 242.0172 and 244.0153.

***N,N*-Dimethyl-2-(thiophen-3-yl)acetamide (a16)**

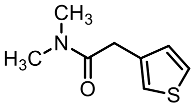

Obtained using general **procedure A** yield 9.0 g, 85%, beige powder, m.p. 56 °C. The compound was used without purification. ^{1}H NMR ($CDCl_3$, 500 MHz), δ: 7.28 (dd, *J* = 4.6, 3.0 Hz, 1H), 7.08 (s, 1H), 7.02 (d, *J* = 5.0 Hz, 1H), 3.72 (s, 2H), 2.99 (broad s, 6H). ^{13}C{^{1}H} NMR ($CDCl_3$, 151 MHz), δ: 170.8, 134.8, 128.3, 125.7, 121.9, 37.7 (broad), 35.7, 35.6 (broad). The NMR data are consistent with the previously reported in the literature.[S9] HRMS: calculated m/z for $[M+H]^+$ $C_8H_{12}NOS^+$ 170.0635, found 170.0634.

2-(Pyridin-4-yl)-1-(pyrrolidin-1-yl)ethanone (a17)

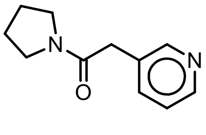

Obtained using general **procedure A** yield 5.2 g, 75%, beige low-melting solid. The compound was used without purification. ^{1}H NMR ($CDCl_3$, 500 MHz), δ: 8.50 (m, 2H), 7.74 (d, J = 8.0 Hz, 1H), 7.30 (dd, J = 7.8, 4.9 Hz, 1H), 3.65 (s, 3H), 3.48 (m, 4H), 1.97 (quint., J = 6.8 Hz, 2H), 1.86 (quint., J = 6.8 Hz, 2H). ^{13}C{^{1}H} NMR ($CDCl_3$, 151 MHz), δ: HRMS: calculated m/z for $[M+H]^+$ $C_{11}H_{15}N_2O^+$ 191.1179, found 191.1182.

2-(1-Methyl-1*H*-pyrazol-4-yl)-1-(pyrrolidin-1-yl)ethanone (a18)

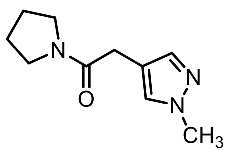

Obtained using general **procedure A** yield 37 g, 87%, yellow powder, m.p. 70 °C. The compound was used without purification. ^{1}H NMR ($CDCl_3$, 500 MHz), δ: 7.41 (s, 1H), 7.39 (s, 1H), 3.88 (s, 3H), 3.46 (m, 6H), 1.95 (quint., *J* = 6.8 Hz, 2H), 1.85 (quint. *J* = 6.8 Hz, 2H). $^{13}C\{^{1}H\}$ NMR ($CDCl_3$, 151 MHz), δ: 169.4, 138.9, 129.6, 114.2, 46.8, 45.9, 38.9, 31.0, 26.1, 24.4. HRMS: calculated m/z for $[M+H]^+$ $C_{10}H_{16}N_3O^+$ 194.1288, found 194.1288.

The counterpart vinyl boronates as well as one vinyl stannane were available at Enamine (https://enaminestore.com/search), and were taken from stocks:

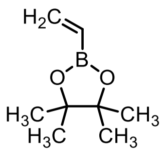

EN300-92920

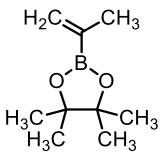

EN300-196435

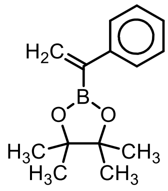

EN300-213139

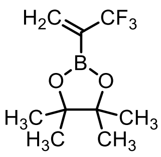

EN300-214510

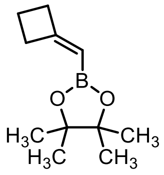

EN300-701233

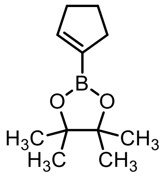

EN300-98773

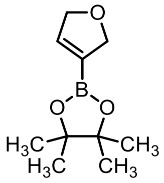

EN300-257047

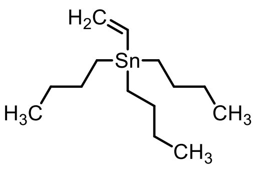

EN300-52493

2. Thermal [2+2]-cycloaddition of vinyl boronates

Optimization of the reaction conditions

The reaction conditions were optimized using reaction of amide **a4** with vinyl pinacol boronate as a benchmark reaction. We varied solvent, reaction time, and the number of equivalents of the reagents. The reaction loading was 0.5-2 g of the starting vinyl pinacol boronate, and the solvent volume was 40 mL. At the end of the reaction performance, the reaction mixture was concentrated in vacuum, and the crude material was analyzed by ^{1}H NMR. The conversion was calculated by comparing the integral intensities of the vinamidinium salt with the resonances of collidine.

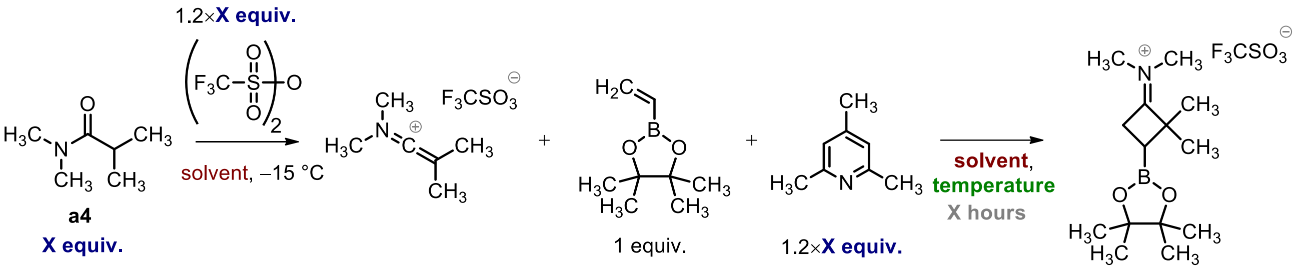

Table S1 Optimization of the reaction conditions.

equivalents of **a4**	solvent	temperature, °C	time, hours	conversion, %
1.2	$CHCl_3$	60	12	87
1.2	CCl_4	60	12	27
1.2	CH_3CN	60	12	22
1.2	dioxane	60	12	no product
1.2	toluene	60	12	53
1.2	$ClCH_2CH_2Cl$	83	12	87
0.6[a]	$ClCH_2CH_2Cl$	83	12	83
2.0	$ClCH_2CH_2Cl$	83	12	79
1.2	$ClCH_2CH_2Cl$	83	8	79

a The reaction was performed with 1.7× excess of vinyl pinacol boronate to the amide.

General procedure B for preparation of compounds **5-31** (compound **8** as example):

2-(2-Chloroethyl)-3-(4,4,5,5-tetramethyl-1,3,2-dioxaborolan-2-yl)cyclobutanone (**8**)

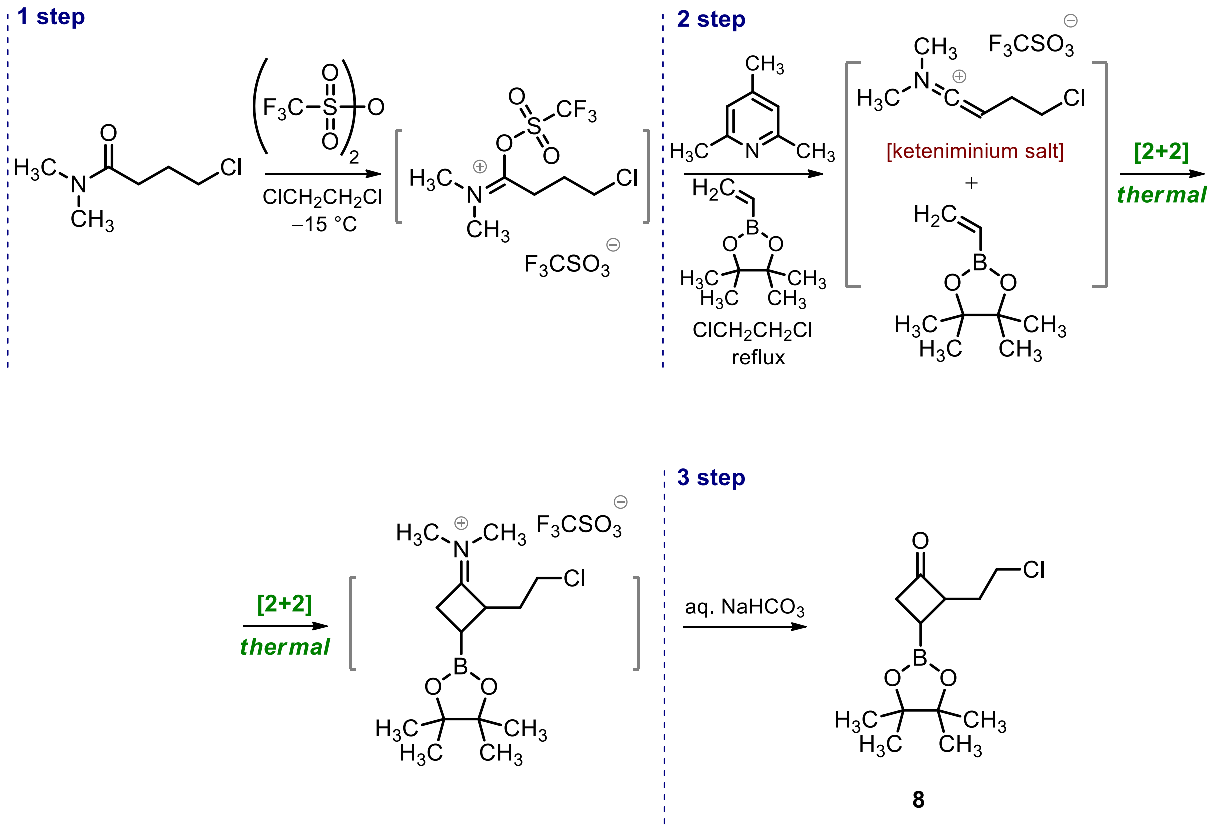

4-Chloro-*N*,*N*-dimethylbutanamide (116.5 g, 0.78 mol, 1.2 equiv.) was dissolved in dichloroethane (1 L), and resulting solution was cooled down to −15 °C. Triflic anhydride (153 mL, 0.91 mol, 1.4 equiv.) was added dropwise, while the temperature did not rise above 0 °C. The mixture was stirred for additional 15 min under temperature below 0 °C. A mixture of 2,4,6-collidine (118 mL, 0.91 mol, 1.4 equiv.) and 4,4,5,5-tetramethyl-2-vinyl-1,3,2-dioxaborolane (100 g, 0.65 mol, 1 equiv.) was added under stirring (*CAUTION*: exothermic reaction!). The reaction mixture was heated up to reflux, and then refluxed for the next 16 hours. The solvent was removed under reduced pressure. Saturated sodium hydrogen carbonate solution (150 mL) and hexane (300 mL) were added to the residue, and the mixture was stirred for 3 hours. The organic fraction was separated and washed with 10% hydrochloric acid solution, followed by washing with pure water until neutral reaction of the aqueous phase (twice). The organic phase was dried over sodium sulphate and concentrated in vacuum. The crude material was purified by distillation at 1 Torr (120 °C fraction) to yield compound **8** (55 g, 0.21 mol, 33%) as a colorless liquid, diastereomeric ratio = 52:48 (gas chromatography), 56:44 (^{1}H NMR).

^{1}H NMR ($CDCl_3$, 600 MHz), δ, two diastereomers 56:44: 3.67 (m, minor) and 3.52 (m, major, 1H, CHCO), 3.60 (m, 2H, CH_2Cl), 3.17 (ddd, J = 17.3, 11.4, 2.1 Hz, minor) and 3.11 (ddd, J = 17.3, 9.6, 1.4 Hz, major, 1H, C*H*HCO), 2.98 (m, major) and 2.95 (m, minor, 1H, CH*H*CO), 2.19 (m, 1H, C*H*HCH_2Cl), 2.02 (m, major) and 1.92 (m, minor, 1H, CH*H*CH_2Cl), 1.42 (m, 1H, CHB), 1.27 (s, major), 1.252 and 1.249 (two s, minor, 12H, CH_3 in Bpin). $^{13}C\{^1H\}$ NMR ($CDCl_3$, 151 MHz), δ, two diastereomers: 209.9 (minor) and 208.5 (major, C=O), 83.90 (minor) and 83.87 (major, C-O), 60.4 (major) and 59.2 (major, *C*HC=O), 46.8 (major) and 46.7 (minor, *C*H_2C=O), 32.6 (major) and 31.5 (minor, *C*H_2CH_2Cl), 24.94 and 24.88 (two, minor), 24.76 and 24.73 (two, major, CH_3 in Bpin), 13.6 (broad) and 10.9 (broad, CH-B). HRMS: calculated m/z for $[M+H]^+$ $C_{12}H_{21}BClO_3^+$ 259.1267, found 259.1262.

2-Methyl-3-(4,4,5,5-tetramethyl-1,3,2-dioxaborolan-2-yl)cyclobutanone (5)

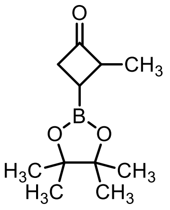

Obtained using **general procedure B**, yield 29 g, 40%, yellow liquid, b.p. 70 °C at 1 Torr, diastereomeric ratio = 61:39 (gas chromatography), 61:39 (^{1}H NMR).

^{1}H NMR ($CDCl_3$, 400 MHz), δ, two diastereomers 61:39: 3.55 (m, minor) and 3.34 (m, major, 1H), 3.13-2.97 (m, 2H), 2.00 (m, minor) and 1.35 (m, major, 1H), 1.29 (s, major) and 1.28 (s, minor, 12H), 1.21 (two d, major and minor, *J* = 7.4 Hz, 3H). ^{13}C{^{1}H} NMR ($CDCl_3$, 126 MHz), δ: 211.7 (minor) and 210.0 (major), 83.3 (minor) and 83.2 (major), 57.2 (major) and 56.3 (minor), 46.2 (major) and 45.9 (minor), 24.5 (minor), 24.4 (minor), 24.23 (major), 24.19 (major), 13.8 (major) and 12.4 (minor). ^{13}C{^{1}H} NMR ($CDCl_3$, 126 MHz), δ, two diastereomers: 211.7 (minor C=O) and 210.0 (major C=O), 83.3 (minor C-O) and 83.2 (major C-O), 57.2 (major CH) and 56.3 (minor CH), 46.2 (major CH_2) and 45.9 (minor CH_2), 24.5 and 24.4 (two minor CH_3 from Bpin), 24.22 and 24.19 (two major CH_3 from Bpin), 13.8 (major CH_3) and 12.4 (minor CH_3), 15.1-10.5 (broad, CH-B). HRMS: calculated m/z for $[M+H]^+$ $C_{11}H_{20}BO_3^+$ 211.1501, found 211.1500.

2,2-Dimethyl-3-(4,4,5,5-tetramethyl-1,3,2-dioxaborolan-2-yl)cyclobutanone (6)

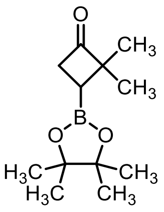

Obtained using **general procedure B**, yield 28 g, 40%, yellow crystalline solid, b.p. 75 °C at 1 Torr, m.p. too low for analysis (melts around room temperature).

^{1}H NMR ($CDCl_3$, 400 MHz), δ: 3.18 (dd, *J* = 17.8, 8.5 Hz, 1H), 3.05 (dd, *J* = 17.8, 10.4 Hz, 1H), 1.58 (dd, *J* = 10.3, 8.7 Hz, 1H), 1.28 (s, 12Hz), 1.26 (s, 3H), 1.21 (s, 3H). ^{1}H{^{13}C} NMR ($CDCl_3$, 151 MHz), δ: 215.3, 83.7, 62.8, 44.1, 24.98, 24.95, 24.2, 21.1, 20.8 (broad, CH-B). HRMS: calculated m/z for $[M+H]^+$ $C_{12}H_{22}BO_3^+$ 225.1657, found 225.1655, for $[M+NH_4]^+$ $C_{12}H_{25}BNO_3^+$ 242.1922, found 242.1919.

2-Cyclopropyl-3-(4,4,5,5-tetramethyl-1,3,2-dioxaborolan-2-yl)cyclobutanone (7)

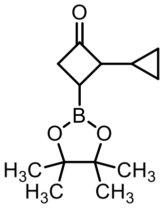

Obtained using **general procedure B**, yield 50 g, 41%, yellow liquid, b.p. 105 °C at 1 Torr, diastereomeric ratio = 59:41 (gas chromatography), 67:33 (^{1}H NMR).

^{1}H NMR ($CDCl_3$, 400 MHz), δ, two diastereomers 67:33: 3.05-2.86 (m, 3H), 1.99 (q, *J* = 10.0 Hz, minor) and 1.46 (q, *J* = 9.5 Hz, major, 1H), 1.27 (s, minor) and 1.26 (s, major, 12H), 1.01 (m, minor) and 0.94 (m, major, 1H), 0.55 (m, 1H), 0.46 (m, 1H), 0.25 (m, 2H). ^{13}C{^{1}H} NMR ($CDCl_3$, 126 MHz), δ, two diastereomers: 210.4 (minor) and 209.1 (major), 83.7, 67.0, 46.4 (minor) and 46.0 (major), 25.0 and 24.9 (two minor), 24.71 and 24.65 (two major), 12.4 (broad, CH-B), 10.0 (major) and 9.9 (minor), 4.7 (minor) and 3.5 (major), 3.0 (minor) and 2.0 (major). HRMS: calculated m/z for $[M+NH_4]^+$ $C_{13}H_{25}BNO_3^+$ 254.1922, found 254.1922.

3-(4,4,5,5-Tetramethyl-1,3,2-dioxaborolan-2-yl)spiro[3.3]heptan-1-one (9)

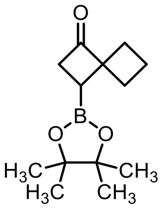

Obtained using **general procedure B**, yield 5 g, 42%, white oil, b.p. 115 °C at 1 Torr.

^{1}H NMR ($CDCl_3$, 500 MHz), δ: 2.98 (dd, *J* = 17.4, 10.4 Hz, 1H), 2.94 (dd, *J* = 17.4, 8.1 Hz, 1H), 2.37 (ddd, *J* = 15.3, 8.3, 3.8 Hz, 1H), 2.25 (ddd, *J* = 15.3, 8.5, 4.0 Hz, 1H), 2.14 (m, 2H), 1.98 (dquint., *J* = 11.0, 8.6 Hz, 1H), 1.79 (m, 1H), 1.69 (dd, *J* = 9.7, 8.6 Hz, 1H), 1.26 and 1.25 (two s, 9H). ^{13}C{^{1}H} NMR ($CDCl_3$, 151 MHz), δ: 214.0, 83.7, 66.5, 44.5, 31.6, 28.9, 25.0, 24.9, 19.6 (broad, CH-B), 16.8. HRMS: calculated m/z for $[M+H]^+$ $C_{13}H_{22}BO_3^+$ 237.1657, found 237.1660.

6,6-Difluoro-3-(4,4,5,5-tetramethyl-1,3,2-dioxaborolan-2-yl)spiro[3.3]heptan-1-one (10)

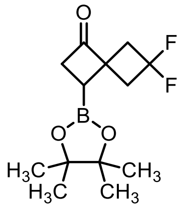

Obtained using **general procedure B**, yield 30 g, 35%, white oil, b.p. 115 °C at 1 Torr.

^{1}H NMR ($CDCl_3$, 500 MHz), δ: 3.15 (dd, *J* = 17.7, 10.7 Hz, 1H), 3.08 (dd, *J* = 17.7, 8.0 Hz, 1H), 3.03 (m, 1H), 2.87 (t, *J* = 12.3 Hz, 2H), 2.64 (m, 1H), 1.93 (dd, *J* = 10.4, 8.5 Hz, 1H), 1.28 (s, 12H). ^{13}C{^{1}H} NMR ($CDCl_3$, 151 MHz), δ: 209.8 (s), 117.7 (dd, *J* = 282, 276 Hz), 84.1 (s), 53.4 (dd, *J* = 9, 7 Hz), 46.5 (s), 44.3 (t, *J* = 24 Hz), 41.6 (t, *J* = 24 Hz), 24.94 (s), 24.88 (s), the CH-B resonance was not observed. ^{19}F NMR ($CDCl_3$, 376 MHz), δ: −91.2 (d, *J* = 191 Hz, 1F), −92.4 (d, *J* = 191 Hz, 1F). HRMS: calculated m/z for $[M+NH_4]^+$ $C_{13}H_{23}BF_2NO_3^+$ 290.1734, found 290.1738.

3-(4,4,5,5-Tetramethyl-1,3,2-dioxaborolan-2-yl)spiro[3.4]octan-1-one (11)

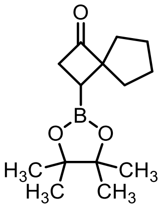

Obtained using **general procedure B**, yield 25 g, 45%, yellow liquid, b.p. 110 °C at 1 Torr.

^{1}H NMR ($CDCl_3$, 500 MHz), δ: 3.05 (dd, *J* = 17.4, 8.4 Hz, 1H), 3.00 (dd, *J* = 17.4, 10.2 Hz, 1H), 2.05 (m, 1H), 1.87 (m, 1H), 1.75-1.64 (m, 7H), 1.26 (s, 12H). ^{1}H{^{13}C} NMR ($CDCl_3$, 126 MHz), δ: 215.2, 83.2, 72.1, 44.1, 36.2, 32.8, 24.9, 24.5, 24.44, 24.41, 19.1 (broad, CH-B). HRMS: calculated m/z for $[M+H]^+$ $C_{14}H_{24}BO_3^+$ 251.1814, found 251.1811.

3-(4,4,5,5-Tetramethyl-1,3,2-dioxaborolan-2-yl)-6-oxaspiro[3.4]octan-1-one (12)

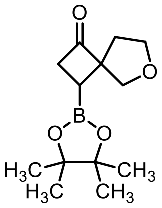

Obtained using **general procedure B**, yield 27 g, 30%, yellow liquid, b.p. 115 °C at 1 Torr, diastereomeric ratio = 64:36 (gas chromatography), 63:37 (^{1}H NMR).

^{1}H NMR ($CDCl_3$, 400 MHz), δ, two diastereomers 63:37: 4.08 (d, *J* = 9.0 Hz, minor), 3.93-3.84 (m, major and minor) and 3.76 (d, *J* = 9.0 Hz, minor, together 4H), 3.09 (m, 2H), 2.37 (ddd, *J* = 12.7, 7.1, 6.2 Hz, major) and 2.19 (dt, *J* = 12.6, 6.5 Hz, minor, 1H), 2.11 (t, *J* = 6.8 Hz, minor) and 2.04 (dt, *J* = 12.6, 7.1 Hz, major, 1H), 1.85 (m, 1H), 1.28 (s, 12H). ^{1}H{^{13}C} NMR ($CDCl_3$, 126 MHz), δ, two diastereomers: 211.4 (minor) and 210.8 (major), 83.48 (major) and 83.45 (minor), 74.4 (minor) and 71.6 (major), 71.1 (major) and 70.6 (minor), 68.2 (minor) and 67.4 (major), 45.4 (major) and 45.2 (minor), 36.3 (major) and 33.1 (minor), 24.45 and 24.42 (two, minor), 24.39 and 24.37 (two, major), 17.9 (broad, CH-B). HRMS: calculated m/z for $[M+H]^+$ $C_{13}H_{22}BO_4^+$ 253.1606, found 253.1603.

3-(4,4,5,5-Tetramethyl-1,3,2-dioxaborolan-2-yl)spiro[3.5]nonan-1-one (13)

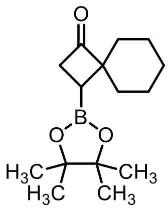

Obtained using **general procedure B**, yield 24 g, 50%, yellow oil, b.p. 120 °C at 1 Torr.

^{1}H NMR ($CDCl_3$, 500 MHz), δ: 3.00 (d, *J* = 9.2 Hz, 2H), 1.77 (m, 2H), 1.70-1.43 (m, 8H), 1.35 (m, 1H), 1.26 (s, 12H). ^{1}H{^{13}C} NMR ($CDCl_3$, 126 MHz), δ: 214.9, 83.1, 67.8, 42.6, 34.3, 30.0, 25.0, 24.5, 24.4, 22.5, 22.4, 19.3 (broad, CH-B). HRMS: calculated m/z for $[M+H]^+$ $C_{15}H_{26}BO_3^+$ 265.1970, found 265.1963, for $[M+Na]^+$ $C_{15}H_{25}BNaO_3^+$ 287.1789, found 287.1786.

3-(4,4,5,5-Tetramethyl-1,3,2-dioxaborolan-2-yl)-7-oxaspiro[3.5]nonan-1-one (14)

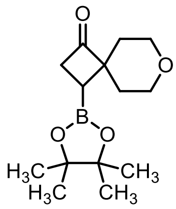

Obtained using **general procedure B**, the crude material was soaked in pentane and filtered, delivering the final product, no distillation was applied, yield 17 g, 15%, yellow crystalline solid, m.p. 81 °C.

^{1}H NMR ($CDCl_3$, 400 MHz), δ: 3.83 (ddd, *J* = 11.5, 9.3, 3.1 Hz, 1H), 3.76 (m, 3H), 3.11 (dd, *J* = 17.8, 8.6 Hz, 1H), 3.05 (dd, *J* = 17.7, 10.3 Hz, 1H), 1.87 (m, 2H), 1.80 (m, 2H), 1.60 (t, *J* = 9.3 Hz, 1H), 1.28 (s, 12H). ^{1}H{^{13}C} NMR ($CDCl_3$, 151 MHz), δ: 213.0, 83.3, 64.5, 64.3, 64.2, 42.9, 33.9, 30.5, 24.5, 19.2 (broad, CH-B). HRMS: calculated m/z for $[M+H]^+$ $C_{14}H_{24}BO_4^+$ 267.1763, found 267.1759, for $[M+Na]^+$ $C_{14}H_{23}BNaO_4^+$ 289.1582, found 289.1578.

7,7-Difluoro-3-(4,4,5,5-tetramethyl-1,3,2-dioxaborolan-2-yl)spiro[3.5]nonan-1-one (15)

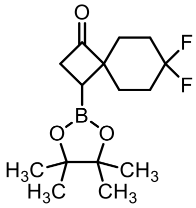

Obtained using **general procedure B**, the crude material was soaked in pentane and filtered, delivering the final product, no distillation was applied, yield 15 g, 30%, yellow crystalline solid, m.p. 80 °C.

^{1}H NMR ($CDCl_3$, 500 MHz), δ: 3.08 (m, 2H), 2.40-1.84 (m, 6H), 1.84 (m, 2H), 1.59 (t, *J* = 9.3 Hz, 1H), 1.26 (s, 12H). ^{1}H{^{13}C} NMR ($CDCl_3$, 151 MHz), δ: 213.4 (s), 122.8 (t, *J* = 241 Hz), 83.9 (s), 65.4 (s), 43.5 (s), 31.0 (t, *J* = 24 Hz), 30.8 (t, *J* = 24 Hz), 27.1 (t, *J* = 5 Hz), 24.95 (s), 24.92 (s), 18.9 (broad s, CH-B). ^{19}F NMR ($CDCl_3$, 376 MHz), δ: −95.97 (d, *J* = 240 Hz, 1F), −100.74 (d, *J* = 240 Hz, 1F). HRMS: calculated m/z for $[M+H]^+$ $C_{15}H_{24}BF_2O_3^+$ 301.1782, found 301.1777.

3-(4,4,5,5-Tetramethyl-1,3,2-dioxaborolan-2-yl)spiro[3.6]decan-1-one (16)

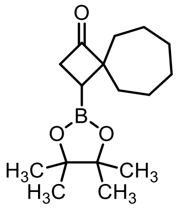

Obtained using **general procedure B**, yield 24 g, 50%, yellow crystalline solid, b.p. 125 °C at 1 Torr, m.p. 53 °C.

^{1}H NMR ($CDCl_3$, 500 MHz), δ: 3.06 (dd, *J* = 17.5, 8.4 Hz, 1H), 2.99 (dd, *J* = 17.4, 10.4 Hz, 1H), 1.89, (m, 2H), 1.74 (m, 2H), 1.64-1.48 (m, 9H), 1.26 (s, 12H). ^{1}H{^{13}C} NMR ($CDCl_3$, 151 MHz), δ: 215.7, 83.6, 71.3, 43.3, 36.6, 33.3, 29.2, 29.1, 24.99, 24.96, 23.9, 23.4, 21.2 (broad, CH-B). HRMS: calculated m/z for $[M+H]^+$ $C_{16}H_{28}BO_3^+$ 279.2127, found 279.2123.

3-Methyl-3-(4,4,5,5-tetramethyl-1,3,2-dioxaborolan-2-yl)cyclobutanone (17)

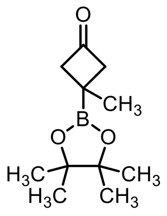

Obtained using **general procedure B**, yield 7 g, 15%, yellow crystalline solid, b.p. 75 °C at 1 Torr, m.p. too low for analysis (melts around room temperature).

^{1}H NMR ($CDCl_3$, 400 MHz), δ: 3.26 (d, *J* = 17.6 Hz, 2H), 2.66 (d, *J* = 18.0 Hz, 2H), 1.35 (s, 3H), 1.28 (s, 12H). ^{13}C{^{1}H} NMR ($CDCl_3$, 126 MHz), δ: 207.8, 83.3, 56.2, 24.1, 23.3, the C-B resonance was not observed. HRMS: calculated m/z for $[M+H]^+$ $C_{11}H_{20}BO_3^+$ 211.1501, found 211.1496, for $[M+NH_4]^+$ $C_{11}H_{23}BNO_3^+$ 228.1766, found 228.1763.

2,3-Dimethyl-3-(4,4,5,5-tetramethyl-1,3,2-dioxaborolan-2-yl)cyclobutanone (18)

Obtained using **general procedure B**, yield 33 g, 36%, white crystalline solid, b.p. 70 °C at 1 Torr, m.p. too low for analysis (melts around room temperature), diastereomeric ratio = 69:31 (gas chromatography), 70:30 (^{1}H NMR).

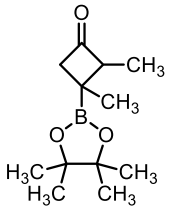

^1H NMR ($CDCl_3$, 500 MHz), δ (two diastereomers, 70:30), major: 3.41 (m, 1H), 3.25 (dd, *J* = 16.3, 1.6 Hz, 1H), 2.38 (dd, *J* = 16.2, 1.9 Hz, 1H), 1.25 (s, 12H), 1.08 (s, 3H), 1.01 (d, *J* = 7.3 Hz, 3H); minor: 3.16 (dd, *J* = 17.0, 2.4 Hz, 1H), 2.92 (m, 1H), 2.61 (dd, *J* = 16.9, 2.8 Hz, 1H), 1.37 (s, 3H), 1.24 (s, 12H), 1.15 (d, *J* = 7.5 Hz, 3H), $^{13}C\{^1H\}$ NMR ($CDCl_3$, 151 MHz), δ, two diastereomers: 211.0 (minor) and 209.9 (major), 83.8 (minor) and 83.7 (major), 65.0 (minor) and 59.3 (major), 54.7 (major) and 54.1 (minor), 24.99 (minor) and 16.8 (major), 24.94 and 24.87 (two, minor), 24.66 and 24.64 (two, major), 12.1 (minor) and 8.12 (major), the C-B resonance was not observed. HRMS: calculated m/z for $[M+H]^+$ $C_{12}H_{22}BO_3^+$ 225.1657, found 225.1654, for $[M+NH_4]^+$ $C_{12}H_{25}BNO_3^+$ 242.1922, found 242.1918.

2,2,3-Trimethyl-3-(4,4,5,5-tetramethyl-1,3,2-dioxaborolan-2-yl)cyclobutanone (19)

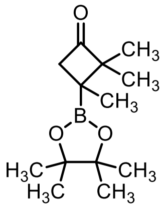

Obtained using **general procedure B**, yield 30 g, 45%, yellow crystalline solid, b.p. 85 °C at 1 Torr, m.p. 44 °C.

^1H NMR ($CDCl_3$, 400 MHz), δ: 3.44 (d, *J* = 17.1 Hz, 1H), 2.46 (d, *J* = 17.1 Hz, 1H), 1.28 (s, 12H), 1.24 (s, 3H), 1.16 (s, 3H), 1.08 (s, 3H). $^{13}C\{^1H\}$ NMR ($CDCl_3$, 151 MHz), δ: 215.2, 83.8, 63.4, 52.7, 25.0, 24.8, 23.2, 22.1 (broad, C-B), 19.4, 17.4. HRMS: calculated m/z for $[M+H]^+$ $C_{13}H_{24}BO_3^+$ 239.1814, found 239.1809.

3-Methyl-3-(4,4,5,5-tetramethyl-1,3,2-dioxaborolan-2-yl)spiro[3.3]heptan-1-one (20)

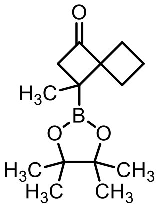

Obtained using **general procedure B**, yield 5.0 g, 30%, yellow crystalline powder, b.p. 127 °C at 1 Torr, m.p. 65 °C.

^1H NMR ($CDCl_3$, 500 MHz), δ: 3.18 (d, *J* = 17.0 Hz, 1H), 2.44 (d, *J* = 17.0 Hz, 1H), 2.20 (m, 1H), 2.12-1.96 (m, 4H), 1.72 (m, 1H), 1.25 and 1.23 (two s, 12H), 1.14 (s, 3H). $^{13}C\{^1H\}$ NMR ($CDCl_3$, 151 MHz), δ: 214.5, 83.7, 68.8, 52.8, 29.3, 25.8, 25.1, 24.8, 20.9 (broad, C-B), 19.1, 16.6. HRMS: calculated m/z for $[M+H]^+$ $C_{14}H_{24}BO_3^+$ 251.1814, found 251.1816.

3-Phenyl-3-(4,4,5,5-tetramethyl-1,3,2-dioxaborolan-2-yl)cyclobutanone (21)

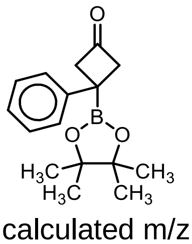

Obtained using **general procedure B**, the product was purified by column chromatography, eluent hexane – ethyl acetate 10:1 (R_f = 0.4), yield 7 g, 20%, yellow crystalline solid, m.p. 84 °C.

^1H NMR ($CDCl_3$, 400 MHz), δ: 7.35 (t, *J* = 7.5 Hz, 2H), 7.24 (m, 3H), 3.59 (m, 2H), 3.38 (m, 2H), 1.20 (s, 12H). $^{13}C\{^1H\}$ NMR ($CDCl_3$, 126 MHz), δ: 206.0, 144.9, 127.9, 126.0, 125.1, 83.8, 55.7, 23.9, the C-B resonance was not observed. HRMS: calculated m/z for $[M+NH_4]^+$ $C_{16}H_{25}BNO_3^+$ 290.1922, found 290.1916.

3-Phenyl-3-(4,4,5,5-tetramethyl-1,3,2-dioxaborolan-2-yl)spiro[3.3]heptan-1-one (22)

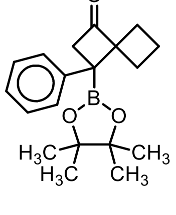

Obtained using **general procedure B**, the product was purified by column chromatography, eluent hexane – ethyl acetate 10:1 (R_f = 0.3), yield 2.0 g, 35%, yellow powder, m.p. 79 °C.

^1H NMR ($CDCl_3$, 500 MHz), δ: 3.18 (d, *J* = 17.0 Hz, 1H), 2.44 (d, *J* = 17.0 Hz, 1H), 2.20 (m, 1H), 2.12-1.96 (m, 4H), 1.72 (m, 1H), 1.25 and 1.23 (two s, 12H), 1.14 (s, 3H). $^{13}C\{^1H\}$ NMR ($CDCl_3$, 151 MHz), δ: 214.5, 83.7, 68.8, 52.8, 29.3, 25.8, 25.1,

24.8, 20.9 (broad, C-B), 19.1, 16.6. HRMS: calculated m/z for $[M+H]^+$ $C_{14}H_{24}BO_3^+$ 251.1814, found 251.1816.

(*2r*,*3r*)-2-(4-Bromophenyl)-3-(4,4,5,5-tetramethyl-1,3,2-dioxaborolan-2-yl)cyclobutanone (25)

Obtained using **general procedure B**. From 51 g of vinyl-Bpin an impure product (22 g, circa 70% purity by ^{1}H NMR; 19% yield) was obtained when using standard work up. NMR analysis of the crude product (picture below) showed presence of two diastereomers with d.r. = 7.0:1.

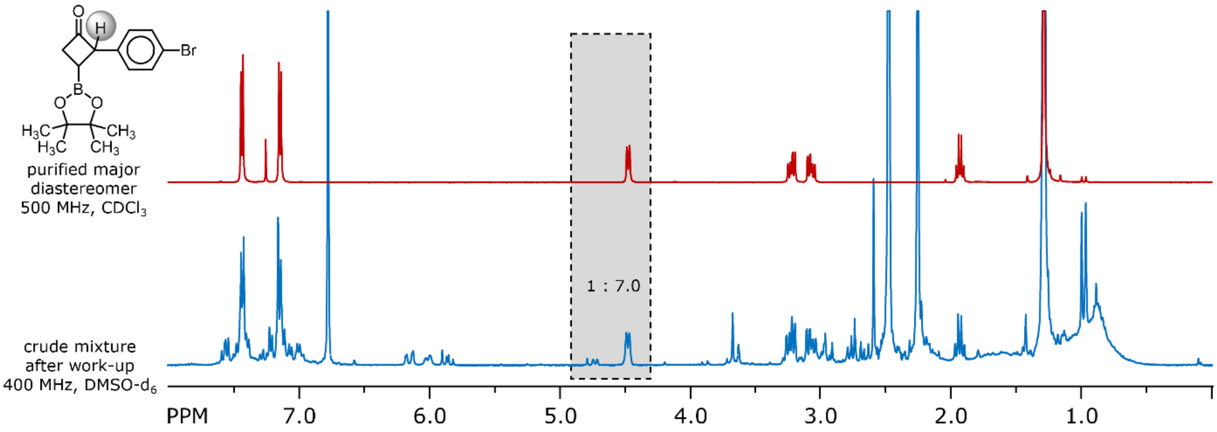

The product was purified by column chromatography producing single major diastereomer in 3.0 g amount, 3% yield, eluent hexane – ethyl acetate 4:1 (R_f = 0.3), yellow powder, m.p. 106 °C.

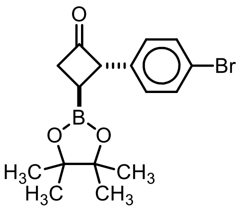

^{1}H NMR ($CDCl_3$, 500 MHz), δ: 7.44 (d, *J* = 8.4 Hz, 2H), 7.15 (d, *J* = 8.4 Hz, 2H), 4.48 (d, *J* = 9.6 Hz, 1H), 3.22 (dd, *J* = 17.0, 9.5 Hz, 1H), 3.07 (ddd, *J* = 17.0, 9.7, 2.3 Hz, 1H), 1.93 (q, *J* = 9.6 Hz, 1H), 1.29 (s, 12H). ^{13}C{^{1}H} NMR ($CDCl_3$, 126 MHz), δ: 205.0, 135.2, 131.1, 128.1, 120.3, 83.6, 65.3, 46.3, 24.2, 14.0 (broad, CH-B). HRMS: calculated m/z for $[M+H]^+$ $C_{16}H_{21}BBrO_3^+$ 351.0762 (^{79}Br) and 353.0742 (^{81}Br), found 351.0760 and 353.0743.

(*2r*,*3r*)-3-(4,4,5,5-Tetramethyl-1,3,2-dioxaborolan-2-yl)-2-(thiophen-3-yl)cyclobutanone (26)

Obtained using **general procedure B**. From 20.3 g of vinyl-Bpin an impure product (12 g, ca. 50% purity by ^{1}H NMR; 33% yield) was obtained when using the standard work up. NMR analysis of the crude product (picture below) showed presence of two diastereomers with d.r. = 2.3:1.

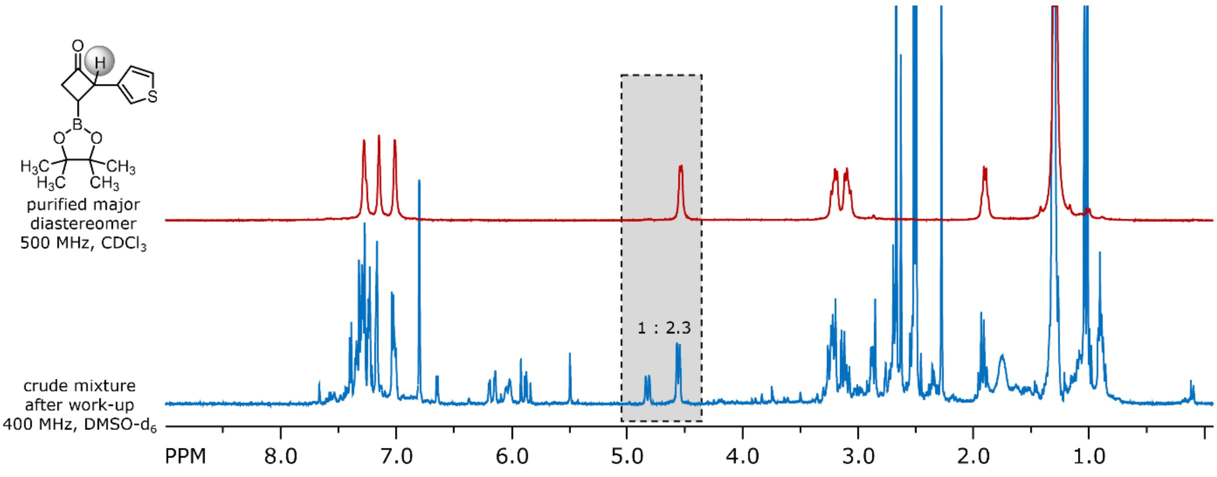

The product was purified by column chromatography producing single major diastereomer in 500 mg amount, 1% yield, eluent hexane – ethyl acetate 4:1 (R_f = 0.3), brown powder, m.p. 66 °C.

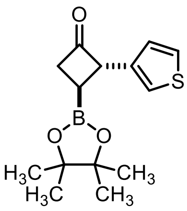

^{1}H NMR ($CDCl_3$, 500 MHz), δ: 7.28 (s, 1H), 7.15 (s, 1H), 7.01 (s, 1H), 4.53 (d, *J* = 7.8 Hz, 1H), 3.20 (m, 1H), 3.08 (m, 1H), 1.90 (q, *J* = 8.8 Hz, 1H), 1.29 (s, 12H). ^{13}C{^{1}H} NMR ($CDCl_3$, 126 MHz), δ: 205.2, 136.6, 126.1, 125.4, 120.2, 83.4, 62.0, 46.4, 24.27, 24.24, 14.4 (broad, CH-B). HRMS: calculated m/z for $[M+H]^+$ $C_{14}H_{20}BO_3S^+$ 279.1221, found 279.1222.

3-((4,4,5,5-Tetramethyl-1,3,2-dioxaborolan-2-yl)methyl)cyclobutanone (28)

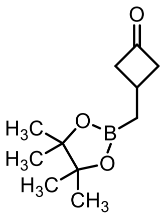

Obtained using **general procedure B**, yield 25 g, 33%, yellow liquid, b.p. 65 °C at 1 Torr.

^{1}H NMR ($CDCl_3$, 400 MHz), δ: 3.20 (m, 2H), 2.73 (m, 2H), 2.62 (m, 1H), 1.25 (s, 12H), 1.19 (m, 2H). ^{13}C{^{1}H} NMR ($CDCl_3$, 126 MHz), δ: 208.6, 82.7, 54.0, 24.3, 19.5, 18.1 (broad, CH-B). HRMS: calculated m/z for $[M+H]^+$ $C_{11}H_{20}BO_3^+$ 211.1501, found 211.1498.

2,2-Dimethyl-3-((4,4,5,5-tetramethyl-1,3,2-dioxaborolan-2-yl)methyl)cyclobutanone (29)

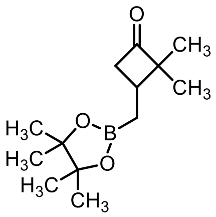

Obtained using **general procedure B**, yield 50 g, 50%, colorless liquid, b.p. 80 °C at 1 Torr.

^{1}H NMR ($CDCl_3$, 400 MHz), δ: 3.18 (dd, *J* = 17.6, 9.1 Hz, 1H), 2.67 (dd, *J* = 17.6, 7.5 Hz, 1H), 2.27 (m, 1H), 1.26 (s, 12H), 1.19 (s, 3H), 1.06 (s, 3H), 1.02 (m, 2H). ^{13}C{^{1}H} NMR ($CDCl_3$, 126 MHz), δ: 215.5, 82.7, 60.7, 49.9, 31.8, 24.30, 24.28, 22.7, 16.9, 12.3 (broad, CH-B). HRMS: calculated m/z for $[M+H]^+$ $C_{13}H_{24}BO_3^+$ 239.1814, found 239.1810, for $[M+NH_4]^+$ $C_{13}H_{27}BNO_3^+$ 256.2079, found 256.2075.

3-((4,4,5,5-Tetramethyl-1,3,2-dioxaborolan-2-yl)methyl)spiro[3.3]heptan-1-one (30)

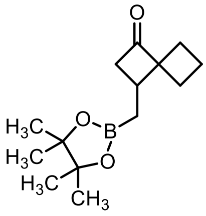

Obtained using **general procedure B**, yield 20 g, 40%, brownish liquid, b.p. 125 °C at 1 Torr.

^{1}H NMR ($CDCl_3$, 500 MHz), δ: 3.07 (dd, *J* = 17.4, 9.0 Hz, 1H), 2.49 (dd, *J* = 17.4, 6.7 Hz, 1H), 2.34 (ddd, *J* = 15.5, 8.3, 7.3 Hz, 1H), 2.28 (m, 1H), 2.08 (m, 3H), 1.98 (m, 1H), 1.76 (m, 1H), 1.245 and 1.242 (two s, 9H), 1.06 (dd, *J* = 15.8, 7.2 Hz, 1H), 0.92 (dd, *J* = 15.9, 8.3 Hz, 1H). ^{13}C{^{1}H} NMR ($CDCl_3$, 151 MHz), δ: 214.7, 83.3, 66.4, 50.5, 30.6, 29.8, 24.9, 24.81, 24.78, 16.4, 13.9 (broad, CH_2-B). HRMS: calculated m/z for $[M+H]^+$ $C_{14}H_{24}BO_3^+$ 251.1814, found 251.1817, for $[M+Na]^+$ $C_{14}H_{23}BNaO_3^+$ 273.1633, found 273.1636.

6,6-Difluoro-3-((4,4,5,5-tetramethyl-1,3,2-dioxaborolan-2-yl)methyl)spiro[3.3]heptan-1-one (31)

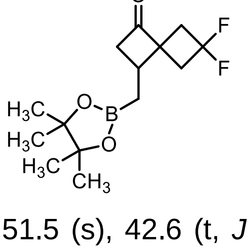

Obtained using **general procedure B**, yield 20 g, 40%, yellow solid that melts around room temperature, b.p. 135 °C at 1 Torr.

^{1}H NMR ($CDCl_3$, 500 MHz), δ: 3.23 (dd, *J* = 17.2, 8.7 Hz, 1H), 2.92 (q, *J* = 12.0 Hz, 1H), 2.72-2.55 (m, 5H), 1.25 and 1.24 (two s, 12H), 1.11 (dd, *J* = 16.2, 7.0 Hz, 1H), 1.03 (dd, *J* = 16.0, 7.9 Hz, 1H). ^{13}C{^{1}H} NMR ($CDCl_3$, 126 MHz), δ: 210.3 (d, *J* = 3 Hz), 117.3 (dd, *J* = 280, 278 Hz), 83.1 (s), 53.3 (t, *J* = 8 Hz), 51.5 (s), 42.6 (t, *J* = 24 Hz), 37.7 (t, *J* = 24 Hz), 30.1 (d, *J* = 3 Hz), 24.4 and 24.2 (two s), 13.8

(broad, CH_2-B). $^{19}F\{^1H\}$ NMR ($CDCl_3$, 376 MHz), δ: −89.6 (d, *J* = 191 Hz, 1F), −92.9 (d, *J* = 191 Hz, 1F). HRMS: calculated m/z for $[M+H]^+$ $C_{14}H_{22}BF_2O_3^+$ 287.1625, found 287.1629.

Failed reactions

Attempts towards compound 4

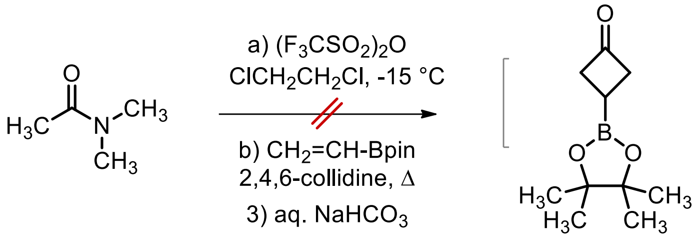

4

The reaction was attempted using the original conditions of **general procedure B** and with the following variations:

(a) Heating temperature was reduced from refluxing dichloroethane (83 °C) to 60 °C.

(b) The number of *N*,*N*-dimethylacetamide equivalents was increased from 1.2 equiv. to 3 equiv., 4 equiv. and 5 equiv.

Crude mixture after the work-up was analyzed by ^{1}H NMR revealing the absence of the target product, presence of starting vinyl pinacol boronate and a complex mixture from the reacted keteniminium salts.

Failed reactions with full conversion of the starting material

Several other substrates were attempted in the reaction following original conditions of **general procedure B**. In all cases, NMR analysis of the crude reaction product revealed a complex mixture with the absence of the starting vinylboronates. The product was not observed neither.

Full alkene conversion, no desired product formed:

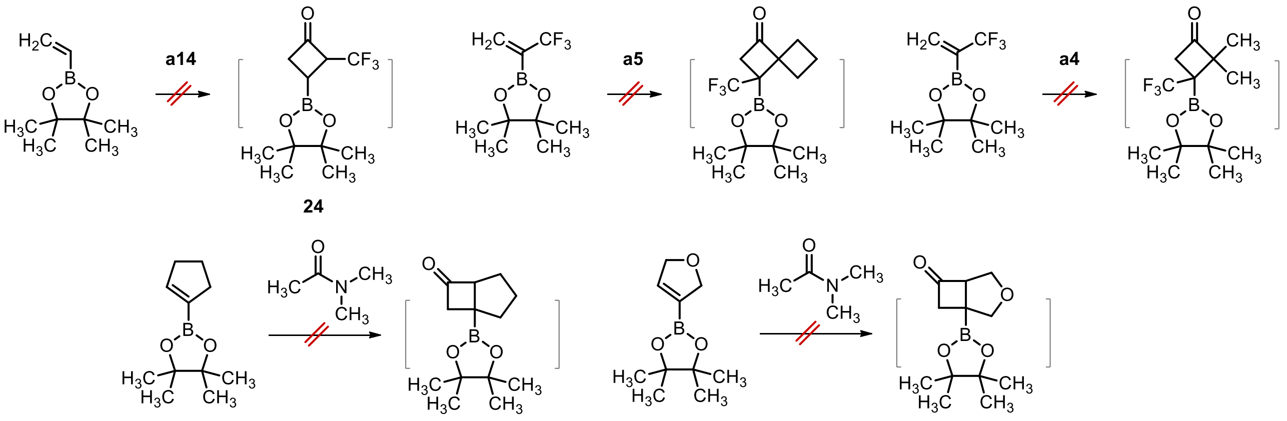

Failed reactions without conversion of the starting material

Several substrates were attempted in the reaction following original conditions of **general procedure B**. In these cases, NMR analysis of the crude reaction product revealed unreacted starting vinylboronates (vinylstannane). The product could not be isolated.

No conversion of the alkene:

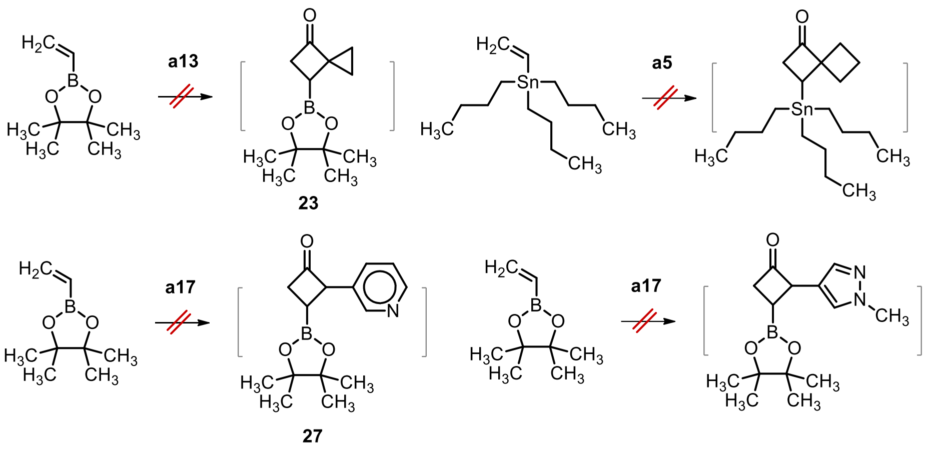

Low yield

(*2r*,*3s*)-2-Methyl-3-phenyl-3-(4,4,5,5-tetramethyl-1,3,2-dioxaborolan-2-yl)cyclobutanone was obtained using **general procedure B**. Analysis of the crude mixture revealed unreacted starting alkene and some amount of the desired product. The product was purified by column chromatography, eluent hexane – ethyl acetate 10:1 (R_f = 0.3), yield 0.85 g, 3.4%, beige crystalline solid, m.p. 71 °C, single diastereomer by gas chromatography, ^{1}H and ^{13}C{^{1}H} NMR. The obtained ^{1}H and ^{13}C NMR spectra agreed with the literature data reported by Clement et al.[S10] and Cui et al.[S11]

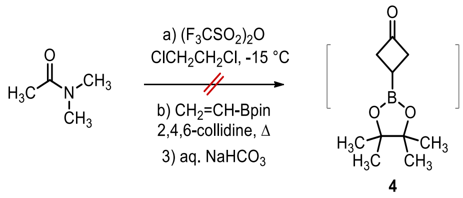

^{1}H NMR ($CDCl_3$, 500 MHz), δ: 7.32 (t, *J* = 7.5 Hz, 2H), 7.23-7.18 (m, 3H), 3.58 (m 1H), 3.52 (dd, *J* = 16.6, 2.1 Hz, 1H), 3.22 (dd, *J* = 16.7, 1.8 Hz, 1H). 1.41 (d, *J* = 7.4 Hz, 3H), 1.18 (s, 6H), 1.15 (s, 6H). ^{13}C{^{1}H} NMR ($CDCl_3$, 151 MHz), δ: 209.0, 147.2, 128.3, 126.1, 125.5, 84.2, 64.1, 53.5, 24.72, 24.65, 13.0, 31.0 (broad, C-B). HRMS: calculated m/z for $[M+H]^+$ $C_{17}H_{24}BO_3^+$ 287.1814, found 287.1812.

3. Derivative compounds

Spiro[3.3]heptan-2-one (33)

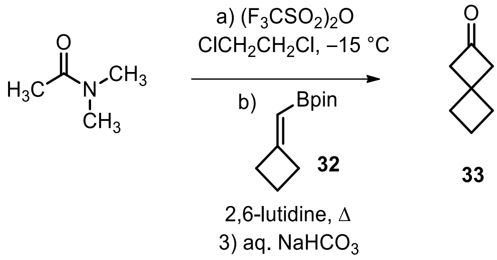

A solution of *N*,*N*-dimethylacetamide (5.38 g, 0.062 mol, 1.17 equiv.) in 1,2-dichloroethane (200 mL) was cooled down to −15 °C. Triflic anhydride (12.1 mL, 0.072 mol, 1.35 equiv.) was added dropwise within 10 minutes, and the reaction mixture was stirred for additional 15 minutes while the temperature was kept below 0 °C. A mixture of compound **32** (10 g, 0.053 mol, 1 equiv.) and 2,6-lutidine (8.7 g, 0.072 mol, 1.35 equiv.) in 1,2-dichloroethane (25 mL) was added quickly (*CAUTION*: exothermic reaction!). The reaction mixture was heated up to reflux, and it was refluxed for the next 16 hours. The solvent was removed under reduced pressure. water (50 mL), hexane (50 mL) and sodium bicarbonate were added to the remaining mixture. The mixture was stirred for 2 hours. The organic layer was separated and washed with 36% hydrochloric acid, followed by washing with water until pH of the aqueous phases was close to neutral. Organic phase was dried over sodium sulphate, solvent was removed under reduced pressure. The title compound was purified by distillation at 20 Torr (70 °C fraction). Compound **33** (2.3 g, 0.021 mol, yield 39%) was obtained as a colorless liquid.

^{1}H NMR ($CDCl_3$, 400 MHz), δ: 3.07 (s, 4H), 2.23 (t, *J* = 7.4 Hz, 4H), 1.97 (quint., *J* = 7.4 Hz, 2H). ^{13}C{^{1}H} NMR ($CDCl_3$, 151 MHz), δ: 208.2, 58.8, 34.1, 33.0, 16.6. HRMS: calculated m/z for $[M+H]^+$ $C_7H_{11}O^+$ 111.0805, found 111.0804.

6-(4,4,5,5-Tetramethyl-1,3,2-dioxaborolan-2-yl)spiro[2.3]hexan-4-one (23)

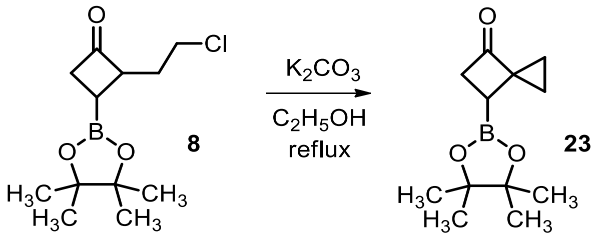

Potassium carbonate (44 g, 1.5 equiv.) was added to a solution of compound **8** (55 g, 0.21 mol, 1 equiv.) in anhydrous ethanol (400 mL). The mixture was refluxed for 16 hours. The solvent was removed under reduced pressure, the residue was mixed with pentane (300 mL) and filtered. The solvent was removed under reduced pressure. The crude material was purified by distillation at 1 Torr (100 °C fraction) to yield compound **23** (40 g, 0.18 mol, 86% yield) as white crystalline solid, m.p. 60 °C.

^{1}H NMR (DMSO-d_6, 500 MHz), δ: 3.29 (d, *J* = 10.0 Hz, 1H), 3.15 (dd, *J* = 17.4, 10.0 Hz, 1H), 2.88 (dd, *J* = 17.4, 6.2 Hz, 1H), 1.98 (dd, *J* = 9.9, 6.4 Hz, 1H), 1.90 (s, 12H), 1.10 (m, 3H). ^{13}C{^{1}H} NMR ($CDCl_3$, 126 MHz), δ: 215.2, 83.1, 45.5, 42.3, 24.37, 24.36, 15.7, 15.0, 14.7 (broad, CH-B). HRMS: calculated m/z for $[M+H]^+$ $C_{12}H_{20}BO_3^+$ 223.1501, found 223.1497.

2,2-Dimethyl-3-(4,4,5,5-tetramethyl-1,3,2-dioxaborolan-2-yl)cyclobutanaminium chloride (37×HCl)

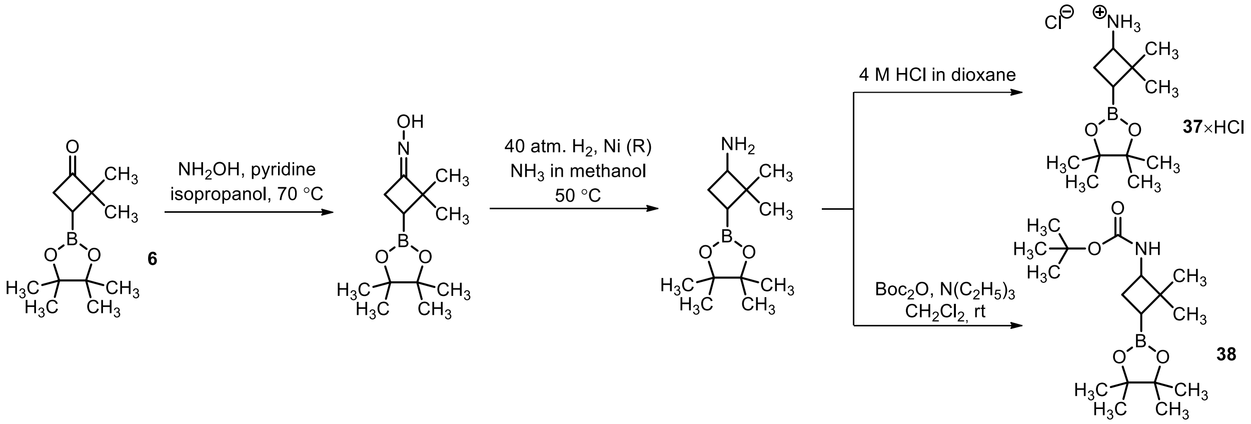

Pyridine (63.6 g, 0.804 mol, 2.2 equiv.) was added to a solution of compound **6** (82 g, 0.366 mol, 1 equiv.) in isopropanol (1 L). Hydroxylamine hydrochloride (55.9 g, 0.805 mol, 2.2 equiv.) was added, and the mixture was heated at 70 °C for 2 hours. The mixture was cooled down to 25 °C and concentrated in vacuum. Methyl *tert*-butyl ether (1 L) was added, the organic phase was washed with water (3×500 mL) and brine (1×200 mL), and dried under sodium sulphate. The solvent was removed under reduced pressure to give the oxime as yellow oil (81.2 g, 0.337 mol), which was used without purification. A high-pressure reactor was charged with the oxime, ammonia solution in methanol (800 mL) and Raney nickel (15 g) as suspension in methanol. The mixture was hydrogenated under hydrogen (40 atm) at 50 °C for 20 hours. After cooling down, the mixture was filtered from the catalyst, the filtrate was concentrated under reduced pressure to give amine as an oil (64.7 g, 0.287 mol). A fraction of this substance (30 g, 0.133 mol, 46% of the amine) was mixed with 4 M hydrochloric acid in dioxane (800 mL). The mixture was concentrated in vacuum giving **37**×HCl as white solid (34.5 g, 0.132 mol, 78% yield from **6**), m.p. 242 °C.

^{1}H NMR (DMSO-d$_6$, 500 MHz), δ, two diastereomers ≈ 1:1: 8.24 (broad s, 3H), 3.31 (t, *J* = 8.5 Hz) and 3.23 (t, *J* = 7.8 Hz, 1H), 2.05-1.89 (m, 2H), 1.41 (dd, *J* = 8.9, 6.4 Hz) and 1.31 (dd, *J* = 11.4, 8.6 Hz, 1H), 1.188 (s), 1.176 (s), 1.164 (s) and 1.159 (s, 12H), 1.09 (s), 1.08 (s), 1.07 (s) and 1.04 (s, 6H). ^{13}C{^{1}H} NMR (DMSO-d$_6$, 151 MHz), δ, two diastereomers: 83.6 and 83.5, 53.0 and 52.9, 42.2 and 40.8, 29.8 and 27.2, 25.4, 25.27, 25.24 and 25.1, 24.9 (broad) and 24.4 (broad), 24.1, 24.0, 23.4 and 19.4. HRMS: calculated m/z for $[M+H]^+$ $C_{12}H_{25}BNO_2^+$ 226.1973, found 226.1973.

***Tert*-butyl (2,2-dimethyl-3-(4,4,5,5-tetramethyl-1,3,2-dioxaborolan-2-yl)cyclobutyl)carbamate (38)**

A fraction of the amine from the previous step (20 g, 0.089 mol) was mixed with dichloromethane (600 mL). Triethylamine (9.88 g, 0.098 mol, 1.1 equiv.) was added followed by a dropwise addition of di-*tert*-butyl dicarbonate (21.3 g, 0.098 mol, 1.1 equiv.). The mixture was stirred at ambient temperature for 16 hours, then some dichloromethane was added and the mixture was washed with brine and dried over anhydrous sodium sulphate. Removal of the solvent under reduced pressure gave target compound **38** as white crystalline solid (23.4 g, 0.072 mol, 73% yield) that melted around room temperature, diastereomeric ratio = 55:45 (gas chromatography), 51:49 (^{1}H NMR).

^{1}H NMR (DMSO-d$_6$, 500 MHz), δ, two diastereomers 51:49, major rotamer: 6.86 (d, *J* = 8.2 Hz) and 6.80 (d, *J* = 7.5 Hz, 1H), 3.70 (q, *J* = 8.5 Hz) and 3.64 (q, *J* = 8.5 Hz, 1H), 1.95-1.74 (m, 2H), 1.35 (s, 9H), 1.19 (s), 1.17 (s), 1.150 (s) and 1.147 (s, 12H), 1.02 (s), 0.99 (s), 0.95 (s) and 0.85 (s, 6H). ^{13}C{^{1}H} NMR (DMSO-d$_6$, 151 MHz), δ, two diastereomers: 155.5, 83.3 and 83.2, 77.8 and 77.7, 54.3 and 53.0, 44.8 and 43.6, 28.7, 30.7 and 27.8, 25.29, 25.27, 25.17 and 25.14, 24.85, 24.78 and 24.45, 24.3 (broad) and 23.4 (broad), 19.7. HRMS: calculated m/z for $[M+Na]^+$ $C_{17}H_{32}BNNaO_4^+$ 348.2317, found 348.2315.

3-(4,4,5,5-Tetramethyl-1,3,2-dioxaborolan-2-yl)spiro[3.3]heptan-1-aminium trifluoroacetate (39)

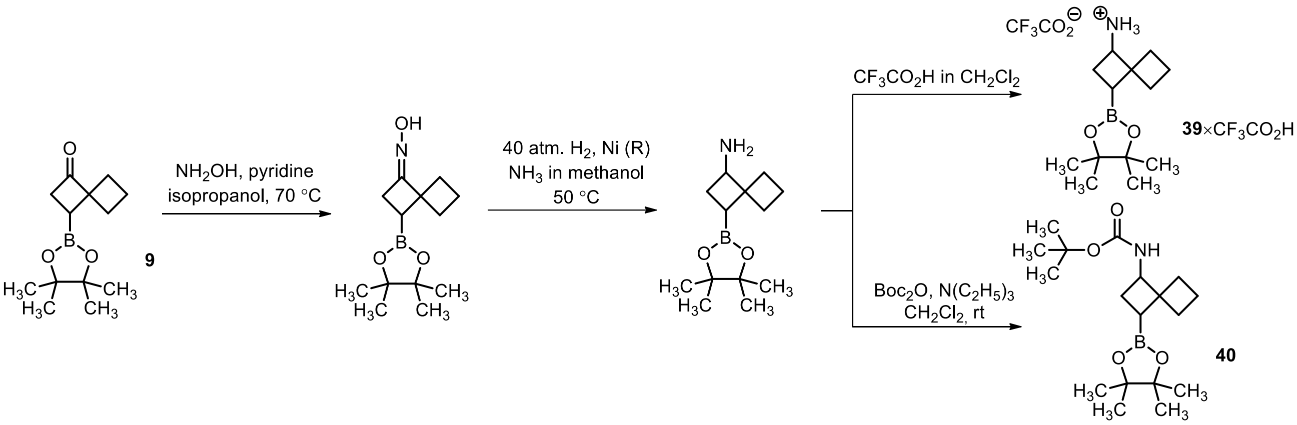

Pyridine (37.22 g, 0.471 mol, 2.2 equiv.) was added to a solution of compound **9** (50.5 g, 0.213 mol, 1 equiv.) in isopropanol (0.8 L). Hydroxylamine hydrochloride (32.7 g, 0.470 mol, 2.2 equiv.) was added, and the mixture was heated at 70 °C for 2 hours. The mixture was cooled down to 25 °C and concentrated in vacuum. Methyl *tert*-butyl ether (0.8 L) was added, the organic phase was washed with water (3×300 mL) and brine (1×200 mL), and dried under sodium sulphate. The solvent was removed under reduced pressure to give the oxime as yellow oil (48.9 g, 0.193. mol), which was used without purification. A high-pressure reactor was charged with the oxime, ammonia solution in methanol (800 mL) and Raney nickel (10 g) as suspension in methanol. The mixture was hydrogenated under hydrogen (40 atm) at 50 °C for 20 hours. After cooling down, the mixture was filtered from the catalyst, the filtrate was concentrated under reduced pressure to give amine as an oil (38.7 g, 0.163 mol). Trifluoroacetic acid (13.2 g, 0.116 mol, 1.1 equiv.) was added dropwise to a stirred solution of the amine (25 g, 0.105 mol, 65% of the amine from the former step) in dichloromethane (50 mL). The mixture was concentrated in vacuum giving **39**×CF_3CO_2H as beige crystalline powder (36.3 g, 0.103 mol, 74% yield from **9**), m.p. 164-188 °C.

^{1}H NMR (DMSO-d$_6$, 500 MHz), δ, two diastereomers ≈ 4:1: 8.16 (s, minor) and 8.07 (s, major, 3H), 3.38 (m, 1H), 2.18-2.07 (m, 2H), 2.02-1.65 (m, 6H), 1.47 (dd, *J* = 11.5, 8.2 Hz, 1H), 1.206 (s) and 1.198 (s, two major), 1.189 (s) and 1.185 (s, two minor, 12H). ^{13}C{^{1}H} NMR (DMSO-d$_6$, 151 MHz), δ, two diastereomers: 158.6 (q, *J* = 31 Hz), 117.6 (q, *J* = 299 Hz), 83.72 (s, major) and 83.68 (s, minor), 51.9 (s, minor) and 51.5 (s, major), 49.1 (s, major) and 47.7 (s, minor), 32.7 (s) and 26.0 (s, two major), 31.2 (s) and 30.3 (s, two minor), 25.3 (s) and 25.06 (s, two major), 25.2 (s) and 25.13 (s, two minor), 24.5 (s, major) and 24.0 (s, minor), 24.2 (broad), 16.2 (s, major) and 16.1 (s, minor). HRMS: calculated m/z for [M+H]+ $C_{13}H_{25}BNO_2^+$ 238.1973, found 238.1981.

***Tert*-butyl (3-(4,4,5,5-tetramethyl-1,3,2-dioxaborolan-2-yl)spiro[3.3]heptan-1-yl)carbamate (40)**

A fraction of the amine from the previous step (15 g, 0.063 mol, 1 equiv.) was mixed with dichloromethane (300 mL). Triethylamine (7.04 g, 0.070 mol, 1.1 equiv.) was added followed by a dropwise addition of di-*tert*-butyl dicarbonate (15.2 g, 0.070 mol, 1.1 equiv.). The mixture was stirred at ambient temperature for 16 hours, then some dichloromethane was added and the mixture was washed with brine and dried over anhydrous sodium sulphate. Removal of the solvent under reduced pressure gave target compound **40** as white crystalline solid (17.9 g, 0.053 mol, 84% yield) m.p. 79-93 °C, diastereomeric ratio = 74:26 (gas chromatography), 77:23 (^{1}H NMR).

^{1}H NMR (DMSO-d$_6$, 500 MHz), δ, two diastereomers 77:23, major rotamer: 7.08 (d, *J* = 8.6 Hz, minor) and 6.97 (d, *J* = 7.9 Hz, major, 1H), 3.89 (q, *J* = 8.5 Hz, minor) and 3.71 (q, *J* = 8.3 Hz,

major, 1H), 2.06-1.53 (series of multiplets, 8H), 1.373 (s, minor) and 1.366 (s, major, 9H), 1.183 (s, minor) and 1.179 (s, major, 12H). $^{13}C\{^1H\}$ NMR (DMSO-d_6, 151 MHz), δ, two diastereomers: 155.6 (major) and 155.5 (minor), 83.30 (major) and 83.26 (minor), 77.90 (minor) and 77.84 (major), 53.5 (minor) and 52.3 (major), 33.4, 28.7, 25.6 and 25.5 (two minor), 25.3 and 25.1 (two major), 23.4 (broad), 16.7 (minor) and 16.6 (major). HRMS: calculated m/z for $[M+Na]^+$ $C_{18}H_{32}BNNaO_4^+$ 360.2317, found 360.2318.

Unsuccessful cyclization of 29 into bicyclo[1.1.1]pentane (41)

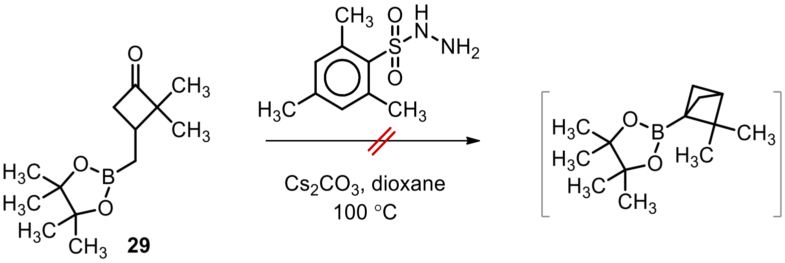

2,4,6-Trimethylbenzenesulfonohydrazide (16.5 g, 0.077 mol, 1.2 equiv.) was added to a solution of compound **29** (15.3 g, 0.064 mol, 1 equiv.) in anhydrous dioxane (100 mL). The mixture was stirred at the room temperature for 12 hours. Cesium carbonate (62.8 g, 0.19 mol, 3 equiv.) was added and the mixture was placed under an argon atmosphere and heated up to 100 °C under stirring for 12 hours. The mixture was cooled down, filtered, the liquid fraction was concentrated under reduced pressure. Resulting mixture was analyzed by ^1H NMR, gas chromatography and high performance liquid chromatography (mass detection) revealing that the starting material was consumed by the target product was absent in the mixture.

Potassium (2,2-dimethyl-3-oxocyclobutyl)trifluoroborate (6a)

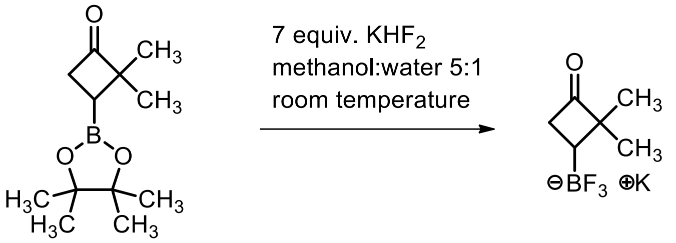

6 **6a**

Compound **6** (10 g, 44.6 mmol, 1 equiv.) was dissolved in a mixture of methanol (80 mL) and water (20 mL). Potassium bifluoride (24.4 g, 312 mmol, 7 equiv.) was added, and the mixture was stirred for 16 hours at room temperature. It was concentrated in vacuum, then acetonitrile (200 mL) was added, the mixture was filtered, and the filtrate was concentrated in vacuum giving compound **6a** as white powder (8.0 g, 39.2 mmol, 88% yield), m.p. non-informative. ^1H NMR (DMSO-d_6, 500 MHz), δ: 2.69 (dd, J = 16.6, 10.7 Hz, 1H), 2.53 (dd, J = 16.6, 7.6 Hz, 1H), 1.00 (s, 3H), 0.97 (s, 3H), 0.70 (m, 1H). $^{13}C\{^1H\}$ NMR (DMSO-d_6, 151 MHz), δ: 218.1, 59.4, 44.8, 27.2 (broad, CH-B), 26.2, 20.0. $^{19}F\{^1H\}$ NMR (DMSO-d_6, 376 MHz), δ: −139.1 (m). HRMS: calculate m/z for $[M]^-$ $C_6H_9BF_3O^-$ 165.0704, found 165.0703.

Potassium trifluoro(3-oxospiro[3.3]heptan-1-yl)borate (9a)

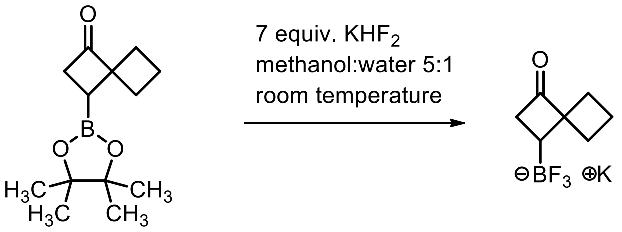

9 **9a**

Compound was obtained in analogous procedure starting from **9** (10 g, 42.4 mmol) producing **9a** as white powder (8.5 g, 39.3 mmol, 93% yield), m.p. 137-230 °C. ^{1}H NMR (D_2O, 500 MHz), δ: 2.83 (dd, *J* = 17.2, 11.6 Hz, 1H), 2.39 (dd, *J* = 17.2, 6.1 Hz, 1H), 2.16 (m, 1H), 2.08-1.92 (m, 3H), 1.75-1.60 (m, 2H), 1.11 (m, 1H). ^{13}C{^{1}H} NMR (D_2O, 126 MHz), δ: 225.1 (s), 64.4 (s), 43.0 (m), 32.1 (s), 26.9 (s), 23.7 (broad, CH-B), 16.3 (s). ^{19}F{^{1}H} NMR (D_2O, 376 MHz), δ: −140.7 (m). HRMS: calculated m/z for $[M]^-$ $C_7H_9BF_3O^-$ 177.0704, not found.

Potassium trifluoro(3-oxospiro[3.5]nonan-1-yl)borate (13a)

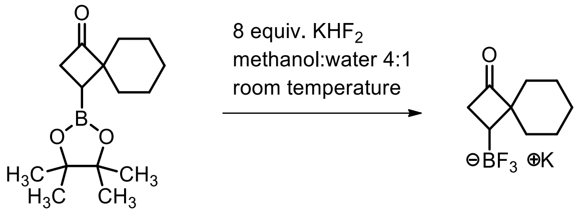

13 **13a**

Compound **13** (20 g, 0.076 mol, 1 equiv.) was dissolved in a mixture of methanol (100 mL) and water (25 mL). Potassium bifluoride (47.3 g, 0.61 mol, 8 equiv.) was added and the mixture was stirred for the next 12 hours. All solvents were removed under reduced pressure, acetonitrile was added to the remaining solid material, the mixture was shaken, filtered, and the remaining solid was discarded. The solvent was removed under reduced pressure giving compound **13a** as white powder (11.6 g, 0.048 mol, 63% yield), m.p. 174 °C.

^{1}H NMR (D_2O, 500 MHz), δ: 2.90 (dd, *J* = 17.4, 11.8 Hz, 1H), 2.46 (dd, *J* = 17.4, 6.8 Hz, 1H), 1.55 (m, 1H), 1.46-1.18 (m, 8H), 0.96 (m, 1H). ^{13}C{^{1}H} NMR (D_2O, 126 MHz), δ: 226.2 (s), 65.2 (s), 42.8 (s), 35.6 (s), 29.0 (s), 25.3 (s), 23.0 (s), 22.6 (s). ^{19}F{^{1}H} NMR (D_2O, 376 MHz), δ: −138.8 (m). HRMS: calculated m/z for $[M]^-$ $C_9H_{13}BF_3O^-$ 205.1017, not found.

3-(4,4,5,5-Tetramethyl-1,3,2-dioxaborolan-2-yl)spiro[3.5]nonan-1-ol (43)

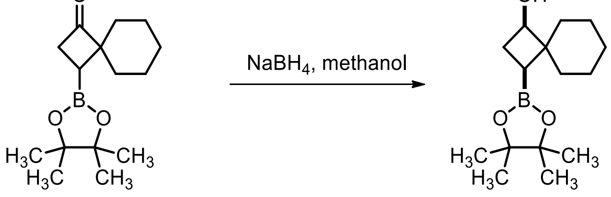

13 **43**

Compound **13** (43.7 g, 0.166 mol, 1 equiv.) was dissolved in anhydrous methanol (500 mL). Sodium borohydride (7.3 g, 0.193 mol, 1.16 equiv.) was added portionwise, and the mixture was stirred for the next 12 hours at the room temperature. The solvent was removed under reduced pressure, water and dichloromethane was added, organic phase was separated, and aqueous phase was additionally extracted by dichloromethane. Combined organic phases were dried over

sodium sulphate. Removing of the solvent under reduced pressure produced **43** as colorless oil (40 g, 0.150 mol, 90% yield),diastereomeric ratio = 84:16 (gas chromatography), 83:17 (^{1}H NMR).

^{1}H NMR ($CDCl_3$, 500 MHz), δ, two diastereomers 83:17: 4.02 (t, *J* = 8.0 Hz, minor) and 3.85 (t, *J* = 6.1 Hz, major, 1H), 2.36 (ddd, *J* = 11.9, 8.9, 7.2 Hz, major) and 2.24 (ddd, *J* = 10.7, 7.9, 3.6 Hz, minor, 1H), 1.91 (broad s, 1H), 1.72 (ddd, *J* = 11.9, 7.6, 5.3 Hz, 1H), 1.63 (m, 1H), 1.55-1.34 (m, 9H), 1.263 and 1.258 (two s, major), 1.25 and 1.24 (two s, minor, 12H), 1.16 (m, 1H). $^{13}C\{^1H\}$ NMR ($CDCl_3$, 151 MHz), δ, two diastereomers: 83.5 (major) and 83.1 (minor), 75.5 (major) and 73.7 (minor), 49.0 (minor) and 48.4 (major), 39.5 (major) and 36.1 (minor), 31.2 and 29.36 (two minor), 29.33 and 29.2 (two major), 26.2 (minor) and 26.1 (major), 25.03 and 24.92 (two major), 24.95 and 24.78 (two minor), 23.20 and 23.18 (two major), 23.13 and 22.5 (two minor), 22.6 (broad, CH-B). HRMS: calculated m/z for $[M+Na]^+$ $C_{15}H_{27}BNaO_3^+$ 289.1946, found 289.1942.

Spiro[3.5]nonane-1,3-diol (44)

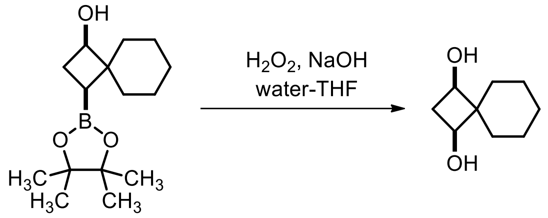

43 **44**

Compound **43** (40 g, 0.150 mol, 1 equiv.) was dissolved in anhydrous tetrahydrofuran. A mixture of sodium hydroxide (12 g, 0.3 mol, 2 equiv.) solution in water (600 mL) and 35% hydrogen peroxide (600 mL) was added. The mixture was stirred for the next 12 hours at the room temperature. The reaction mixture was extracted by methyl tert-butyl ether (300 mL), the organic phase was washed with water (2×100 mL) and dried over sodium sulphate. The solvent was removed under reduced pressure. Recrystallization from methyl tert-butyl ether produced compound **44** (15 g, 0.096 mol, 64%) as white crystalline solid, m.p. 74 °C, diastereomeric ratio = 77:23 (gas chromatography), 82:18 (^{1}H NMR).

^{1}H NMR (CD_3OD, 500 MHz), δ, two diastereomers 82:18: 3.94 (t, *J* = 6.4 Hz, minor) and 3.43 (t, *J* = 7.6 Hz, major, 2H), 2.57 (dt, *J* = 11.4, 7.2 Hz, major) and 2.11 (t, *J* = 6.5 Hz, minor, 1H), 1.80 (dt, *J* = 11.5, 8.3 Hz, major, 1H), 1.67-1.59 (m, 4H), 1.53-1.36 (m, 6H). $^{13}C\{^1H\}$ NMR (CD_3OD, 126 MHz), δ, major diastereomer: 67.9, 49.7, 37.0, 36.4, 25.5, 23.7, 22.6, 22.3. HRMS: calculated m/z for $[M+H]^+$ $C_9H_{17}O_2^+$ 157.1224, found 157.1221, for $[M+Na]^+$ $C_9H_{16}NaO_2^+$ 179.1043, found 179.1035.

4. Checkcif for compound X-ray crystal structures

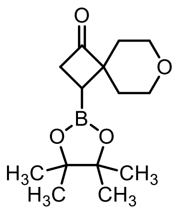

3-(4,4,5,5-Tetramethyl-1,3,2-dioxaborolan-2-yl)-7-oxaspiro[3.5]nonan-1-one (**14**)

Cambridge Crystallographic Data Centre (CCDC) deposition number: CCDC 2312008.

Bond precision:	C-C = 0.0114 A		Wavelength=0.71073
Cell:	a=10.847(4)	b=6.213(2)	c=11.060(3)
	alpha=90	beta=105.661(8)	gamma=90
Temperature:	273 K		

	Calculated	Reported
Volume	717.7(4)	717.7(4)
Space group	P 21	P 1 21 1
Hall group	P 2yb	P 2yb
Moiety formula	C14 H23 B O4	C14 H23 B O4
Sum formula	C14 H23 B O4	C14 H23 B O4
Mr	266.13	266.13
Dx,g cm-3	1.232	1.231
Z	2	2
Mu (mm-1)	0.087	0.087
F000	288.0	288.0
F000'	288.15	
h,k,lmax		12,7,13
Nref		2503
Tmin,Tmax	0.985,0.996	
Tmin'	0.985	

Correction method= Not given

Data completeness= Theta(max)= 24.989

R(reflections)= 0.0972(2183) wR2(reflections)= 0.2817(2503)

S = 1.177 Npar= 177

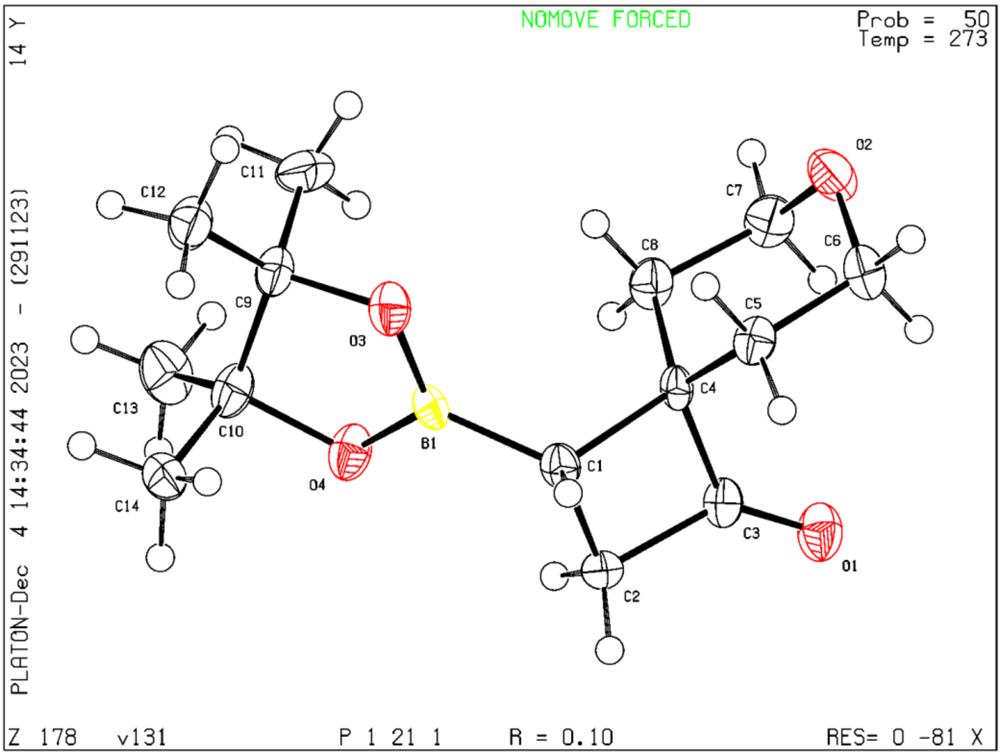

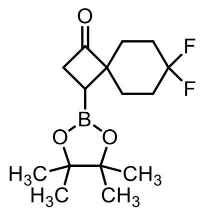

7,7-Difluoro-3-(4,4,5,5-tetramethyl-1,3,2-dioxaborolan-2-yl)spiro[3.5]nonan-1-one (**15**)

Cambridge Crystallographic Data Centre (CCDC) deposition number: CCDC 2312007.

Bond precision: C-C = 0.0059 A Wavelength=0.71073

Cell: a=13.3713(5) b=6.2669(3) c=18.6934(6)
alpha=90 beta=91.481(2) gamma=90

Temperature: 296 K

	Calculated	Reported
Volume	1565.92(11)	1565.92(11)
Space group	P 21/n	P 1 21/n 1
Hall group	-P 2yn	-P 2yn
Moiety formula	C15 H23 B F2 O3	C15 H23 B F2 O3
Sum formula	C15 H23 B F2 O3	C15 H23 B F2 O3
Mr	300.14	300.14
Dx,g cm-3	1.273	1.273
Z	4	4
Mu (mm-1)	0.101	0.101
F000	640.0	640.0
F000'	640.40	
h,k,lmax		15,7,22
Nref		2764
Tmin,Tmax	0.989,0.994	
Tmin'	0.980	

Correction method= Not given

Data completeness= Theta(max)= 24.993

R(reflections)= 0.0812(2063) wR2(reflections)= 0.2000(2764)

S = 1.159 Npar= 194

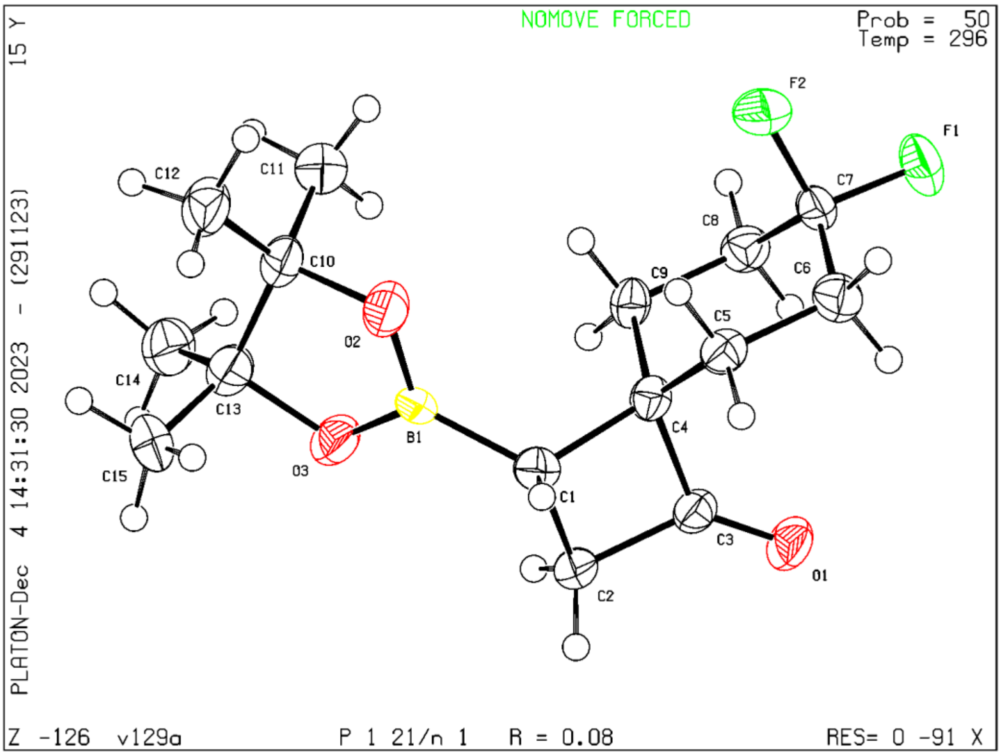

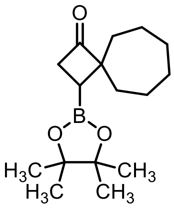

3-(4,4,5,5-Tetramethyl-1,3,2-dioxaborolan-2-yl)spiro[3.6]decan-1-one (**16**)

Cambridge Crystallographic Data Centre (CCDC) deposition number: CCDC 2312010.

Bond precision: C-C = 0.0026 A Wavelength=0.71073

Cell: a=6.7371(3) b=9.7392(5) c=13.4409(6)
alpha=102.908(3) beta=102.707(3) gamma=103.170(3)

Temperature: 296 K

	Calculated	Reported
Volume	802.21(7)	802.20(7)
Space group	P -1	P -1
Hall group	-P 1	-P 1
Moiety formula	C16 H27 B O3	C16 H27 B O3
Sum formula	C16 H27 B O3	C16 H27 B O3
Mr	278.19	278.18
Dx,g cm-3	1.152	1.152
Z	2	2
Mu (mm-1)	0.076	0.076
F000	304.0	304.0
F000'	304.14	
h,k,lmax		8,11,15
Nref		2822
Tmin,Tmax	0.991,0.992	
Tmin'	0.985	

Correction method= Not given

Data completeness= Theta(max)= 24.998

R(reflections)= 0.0431(2207) wR2(reflections)= 0.1055(2822)

S = 1.035 Npar= 185

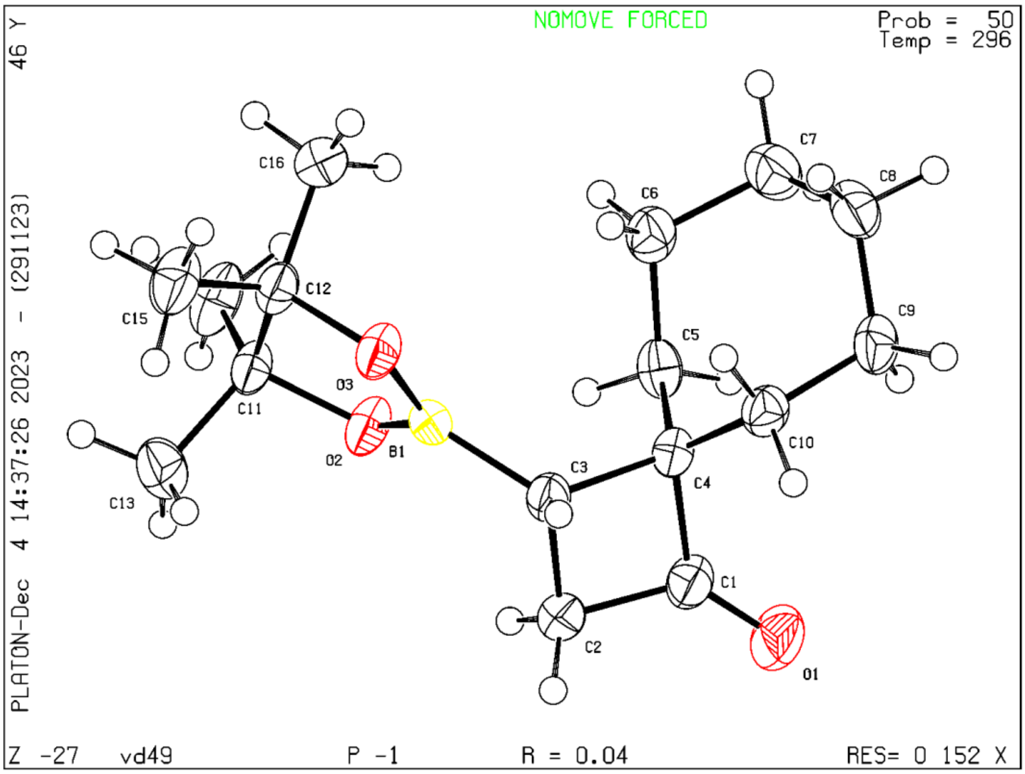

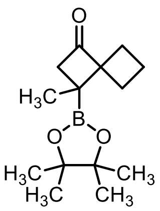

3-Methyl-3-(4,4,5,5-tetramethyl-1,3,2-dioxaborolan-2-yl)spiro[3.3]heptan-1-one (**20**)

Cambridge Crystallographic Data Centre (CCDC) deposition number: CCDC 2312009.

Bond precision:	C-C = 0.0068 A		Wavelength=0.71073
Cell:	a=9.489(2)	b=14.273(3)	c=11.565(3)
	alpha=90	beta=107.466(15)	gamma=90
Temperature:	296 K		

	Calculated	Reported
Volume	1494.1(6)	1494.2(6)
Space group	P 21/c	P 1 21/c 1
Hall group	-P 2ybc	-P 2ybc
Moiety formula	C14 H23 B O3	C14 H23 B O3
Sum formula	C14 H23 B O3	C14 H23 B O3
Mr	250.13	250.13
Dx,g cm-3	1.112	1.112
Z	4	4
Mu (mm-1)	0.075	0.075
F000	544.0	544.0
F000'	544.26	
h,k,lmax		11,16,13
Nref		2508
Tmin,Tmax	0.991,0.993	
Tmin'	0.985	

Correction method= Not given

Data completeness= Theta(max)= 24.989

R(reflections)= 0.0829(1110) wR2(reflections)= 0.2449(2508)

S = 0.883 Npar= 168

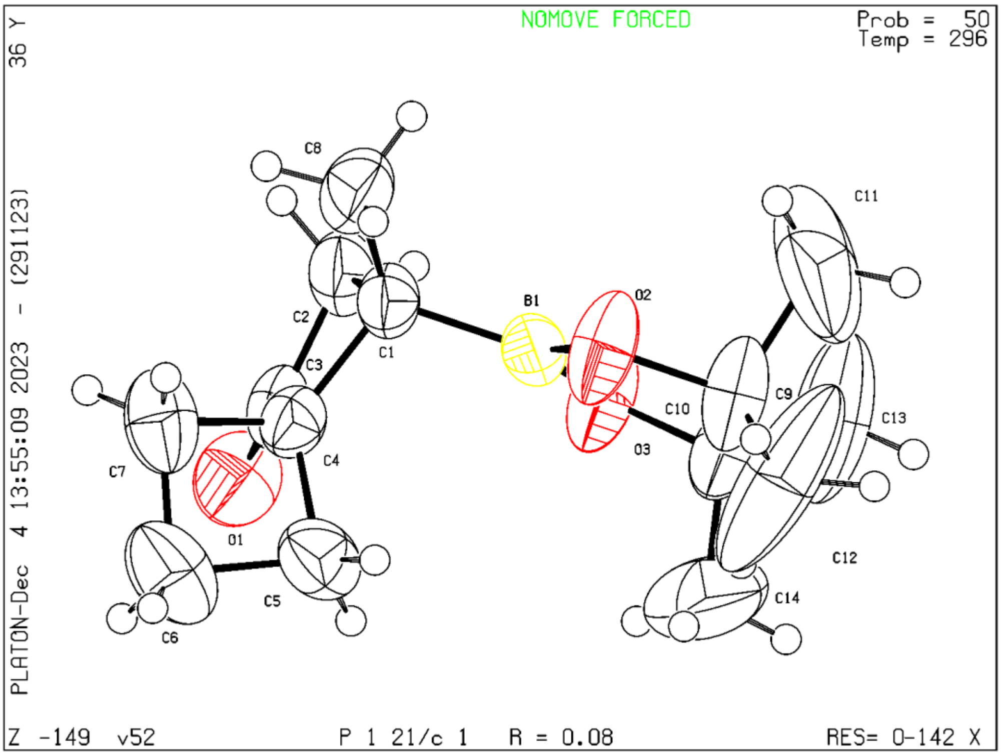

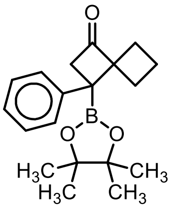

3-Phenyl-3-(4,4,5,5-tetramethyl-1,3,2-dioxaborolan-2-yl)spiro[3.3]heptan-1-one (**22**)

Cambridge Crystallographic Data Centre (CCDC) deposition number: CCDC 2312011.

Bond precision:		C-C = 0.0053 A		Wavelength=0.71073
Cell:	a=6.9091(9)	b=16.369(2)	c=15.729(2)	
	alpha=90	beta=92.089(10)	gamma=90	
Temperature:	296 K			

	Calculated	Reported
Volume	1777.7(4)	1777.7(4)
Space group	P 21/n	P 1 21/n 1
Hall group	-P 2yn	-P 2yn
Moiety formula	C19 H25 B O3	C19 H25 B O3
Sum formula	C19 H25 B O3	C19 H25 B O3
Mr	312.20	312.20
Dx,g cm-3	1.166	1.167
Z	4	4
Mu (mm-1)	0.076	0.076
F000	672.0	672.0
F000'	672.30	
h,k,lmax		8,19,18
Nref		3128
Tmin,Tmax	0.994,0.996	
Tmin'	0.986	

Correction method= Not given

Data completeness= Theta(max)= 24.999

R(reflections)= 0.0706(1504) wR2(reflections)= 0.1608(3128)

S = 1.003 Npar= 212

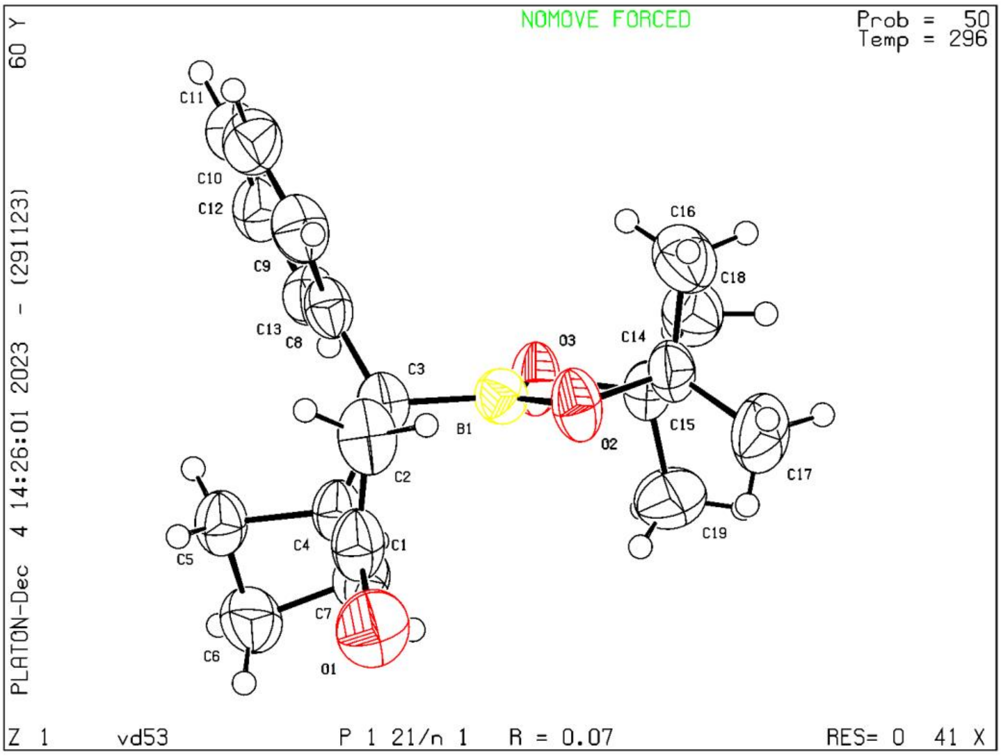

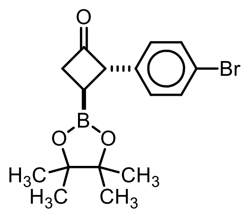

(2*r*,3*r*)-2-(4-Bromophenyl)-3-(4,4,5,5-tetramethyl-1,3,2-dioxaborolan-2-yl)cyclobutanone (**25**)

Cambridge Crystallographic Data Centre (CCDC) deposition number: CCDC 2321052.

Bond precision: C-C = 0.0086 A Wavelength=0.71073

Cell: a=6.1342(11) b=8.6448(14) c=16.040(3)
alpha=74.401(10) beta=86.768(16) gamma=89.349(12)

Temperature: 296 K

	Calculated	Reported
Volume	818.0(3)	817.9(2)
Space group	P -1	P -1
Hall group	-P 1	-P 1
Moiety formula	C16 H20 B Br O3	C16 H20 B Br O3
Sum formula	C16 H20 B Br O3	C16 H20 B Br O3
Mr	351.03	351.04
Dx,g cm-3	1.425	1.425
Z	2	2
Mu (mm-1)	2.518	2.519
F000	360.0	360.0
F000'	359.58	
h,k,lmax	7,10,19	7,10,19
Nref	2885	2874
Tmin,Tmax	0.724,0.817	0.513,0.745
Tmin'	0.662	

Correction method= # Reported T Limits: Tmin=0.513 Tmax=0.745 AbsCorr = MULTI-SCAN

Data completeness= 0.996 Theta(max)= 24.999

R(reflections)= 0.0768(1759) wR2(reflections)= 0.2234(2874)

S = 1.032 Npar= 194

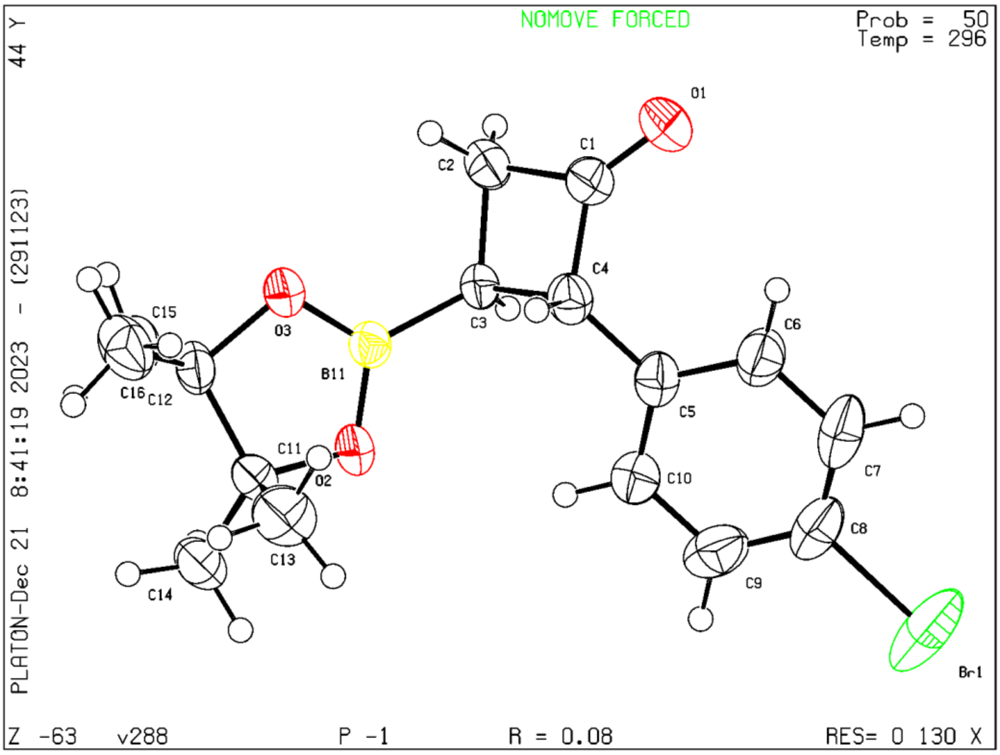

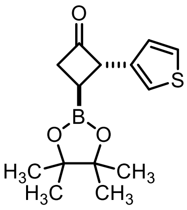

(2*r*,3*r*)-3-(4,4,5,5-Tetramethyl-1,3,2-dioxaborolan-2-yl)-2-(thiophen-3-yl)cyclobutanone (**26**)

Cambridge Crystallographic Data Centre (CCDC) deposition number: CCDC 2321392.

Bond precision: C-C = 0.0060 A Wavelength=0.71073

Cell: a=6.1465(9) b=8.6888(12) c=14.071(2)
alpha=102.121(9) beta=90.881(11) gamma=90.055(10)

Temperature: 273 K

	Calculated	Reported
Volume	734.63(18)	734.63(18)
Space group	P -1	P -1
Hall group	-P 1	-P 1
Moiety formula	C14 H19 B O3 S	C14 H19 B O3 S
Sum formula	C14 H19 B O3 S	C14 H19 B O3 S
Mr	278.16	278.16
Dx,g cm-3	1.258	1.258
Z	2	2
Mu (mm-1)	0.220	0.220
F000	296.0	296.0
F000'	296.38	
h,k,lmax	7,10,16	7,10,16
Nref	2590	2578
Tmin,Tmax	0.984,0.989	
Tmin'	0.974	

Correction method= Not given

Data completeness= 0.995 Theta(max)= 24.998

R(reflections)= 0.0706(1258) wR2(reflections)= 0.2018(2578)

S = 0.990 Npar= 189

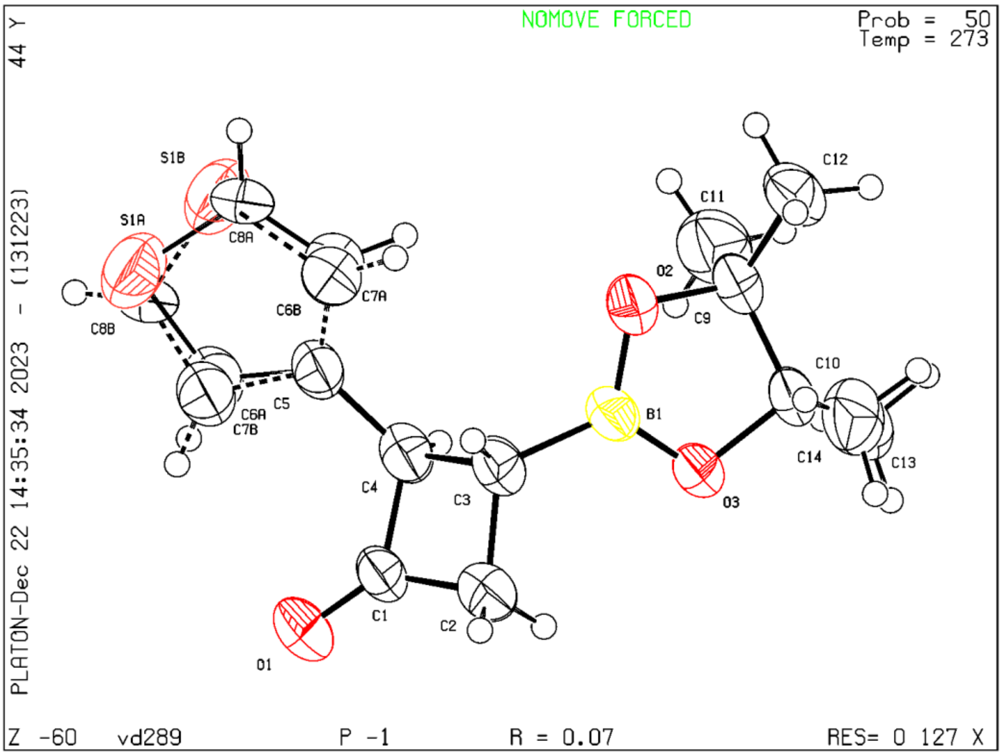

5. Copies of the NMR spectra

Starting materials

^{1}H NMR for amide ***a1***

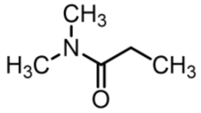

Amide **a1** ($CDCl_3$, 500 MHz)

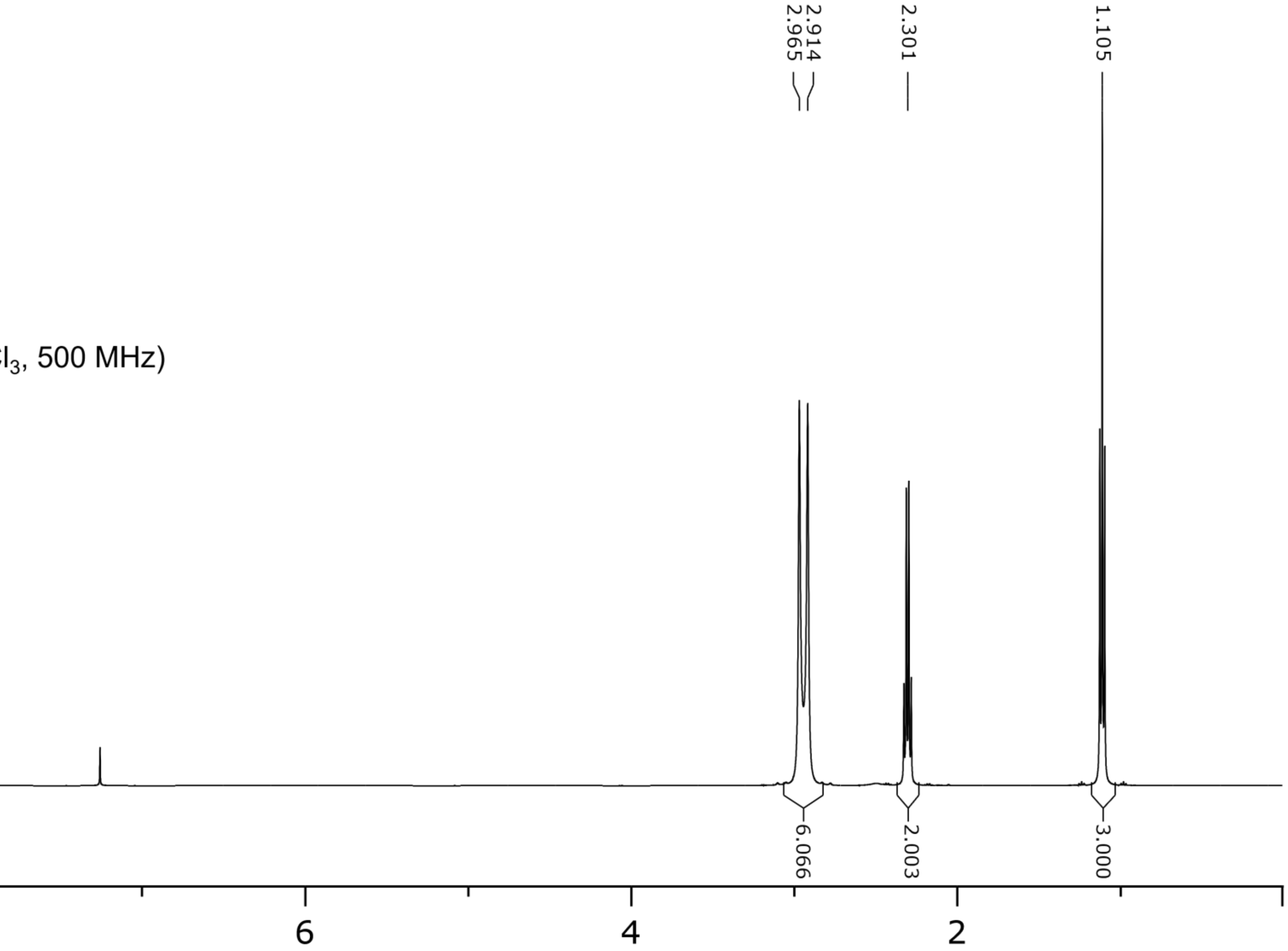

*$^{13}C\{^{1}H\}$ NMR for amide **a1***

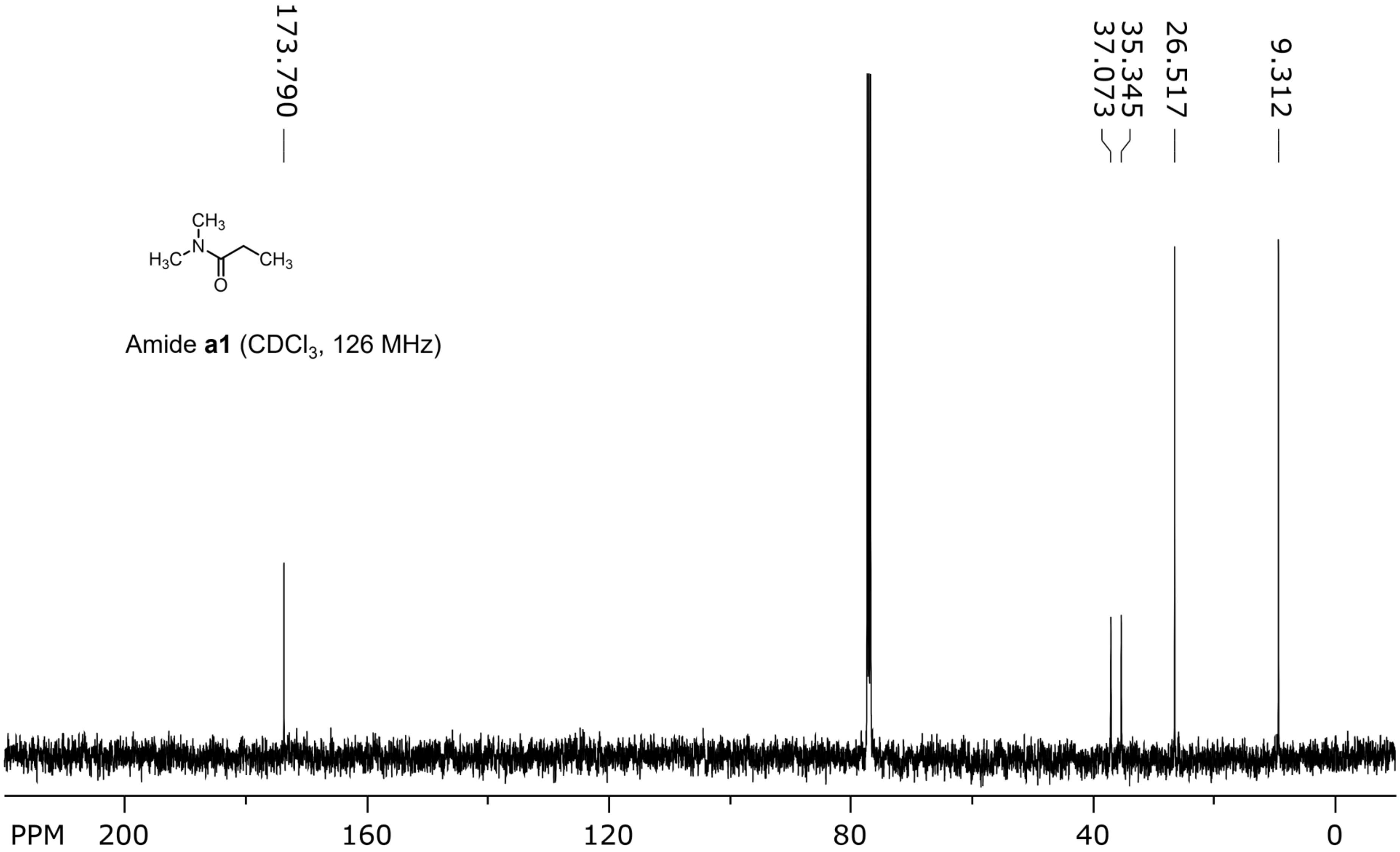

Amide **a1** ($CDCl_3$, 126 MHz)

^{1}H NMR for amide ***a2***

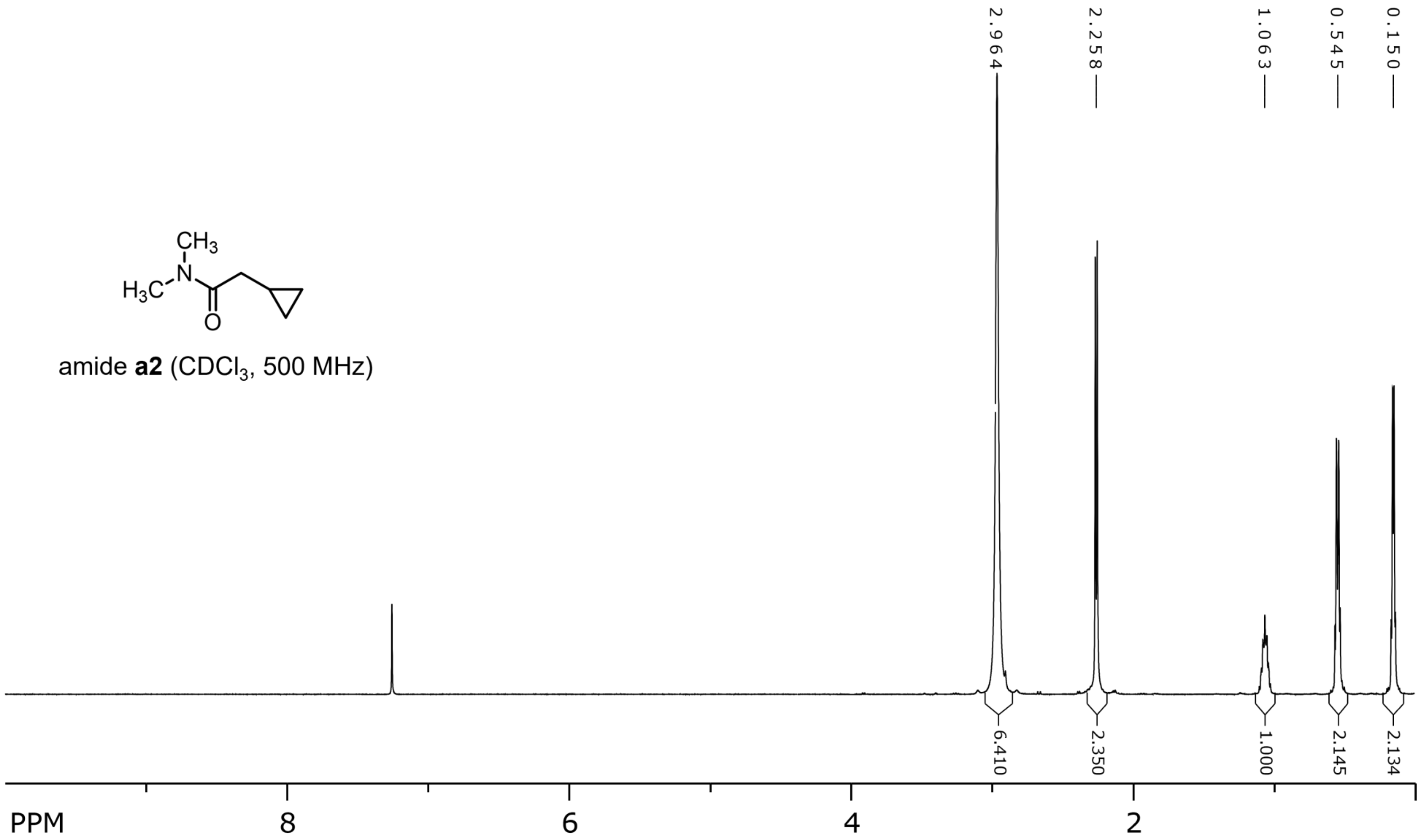

*^{13}C{^{1}H} NMR for amide **a2***

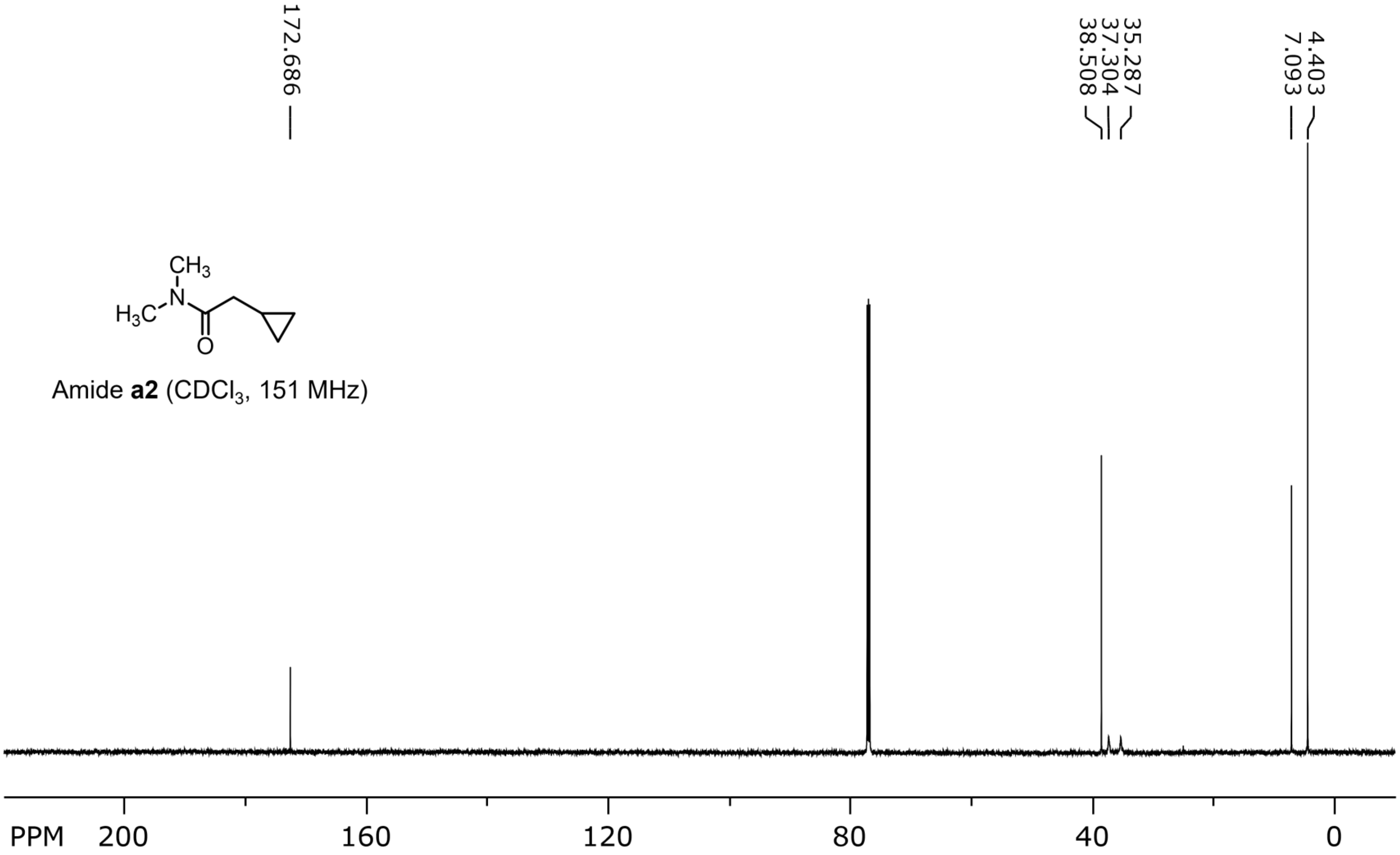

Amide **a2** ($CDCl_3$, 151 MHz)

[1]*H NMR for amide* ***a3***

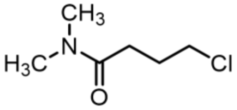

Amide **a3** (DMSO-d_6, 400 MHz)

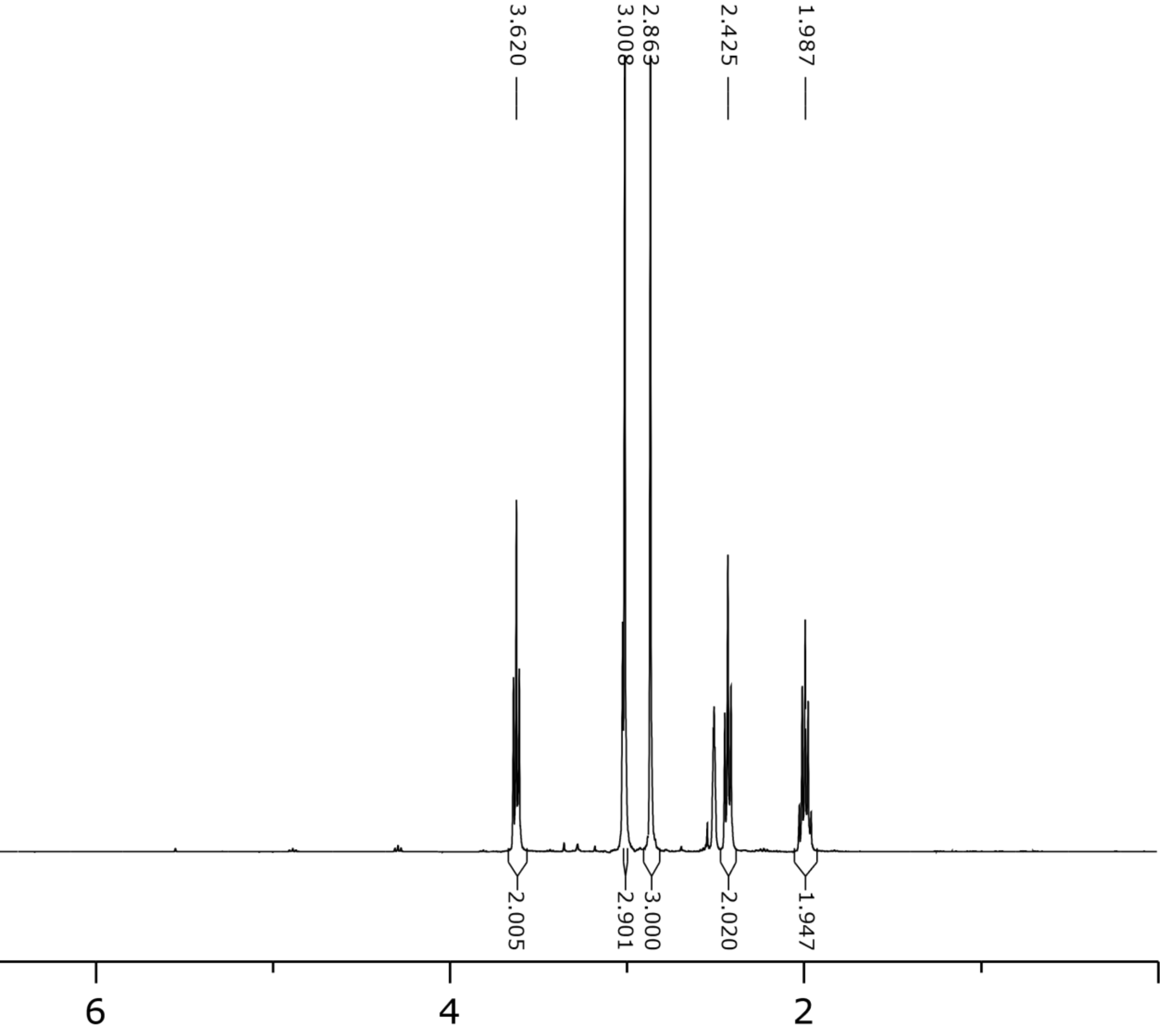

$^{13}C\{^1H\}$ *NMR for amide* ***a3***

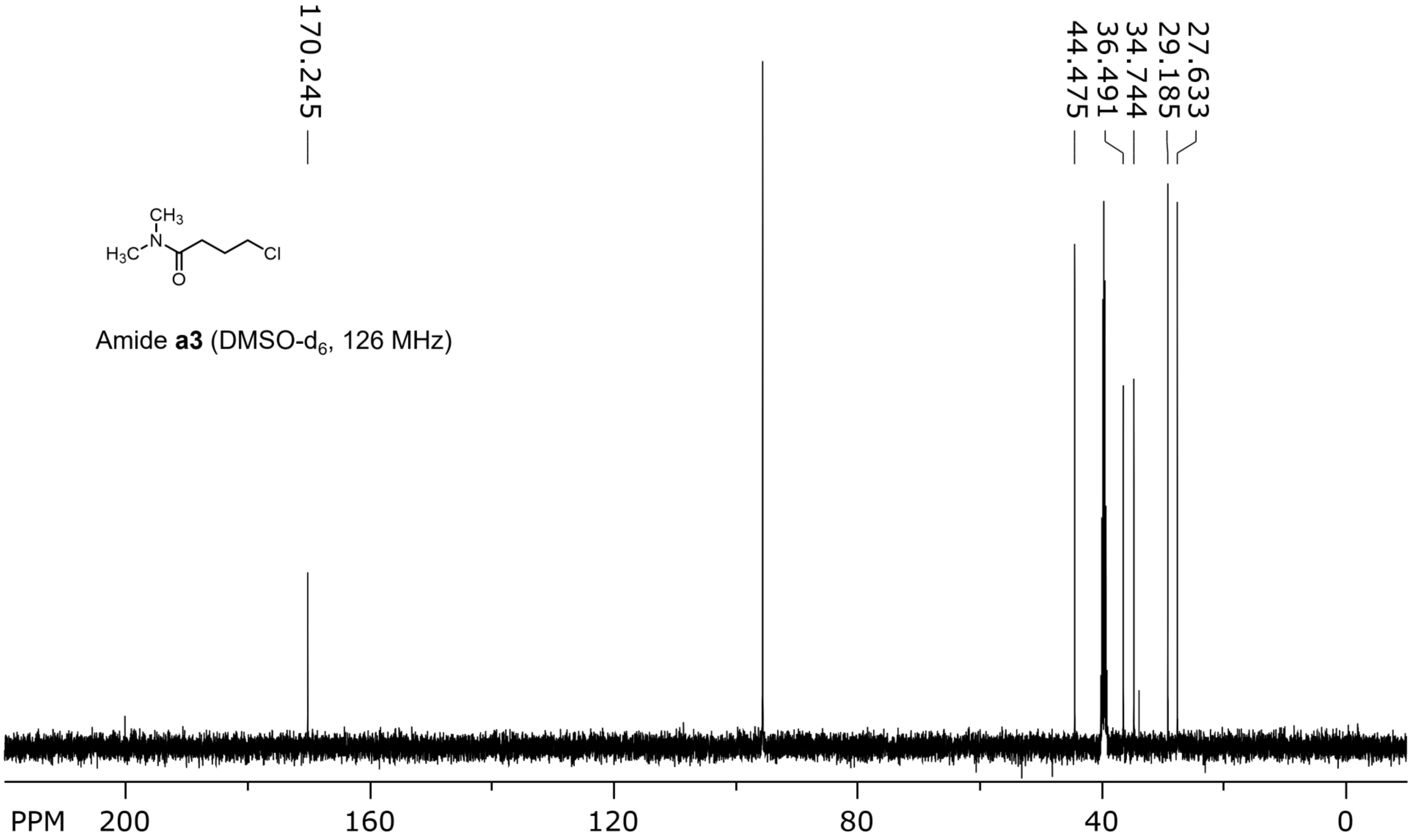

*^{1}H NMR for amide **a4***

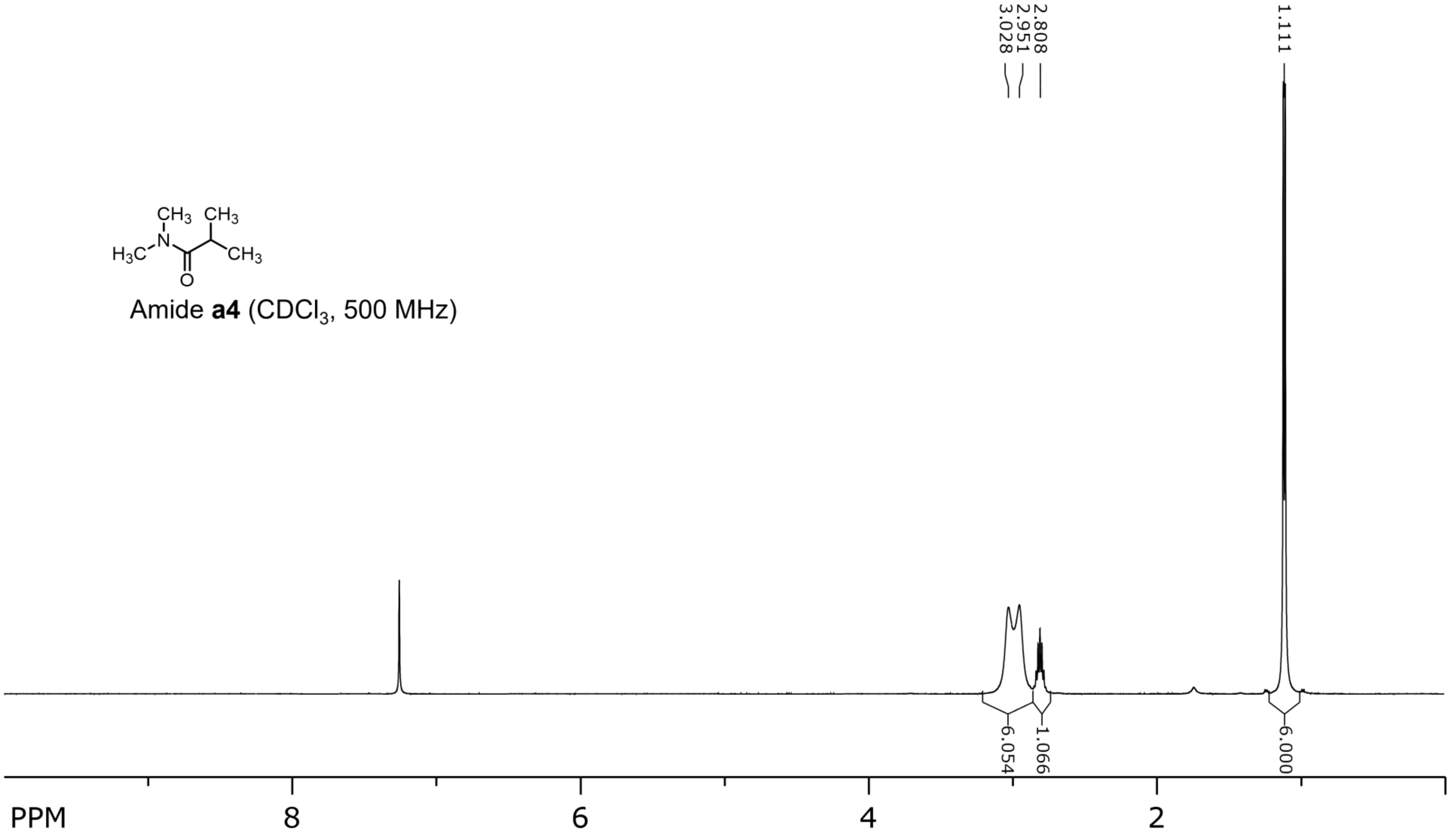

Amide **a4** ($CDCl_3$, 500 MHz)

$^{13}C\{^1H\}$ *NMR for amide* ***a4***

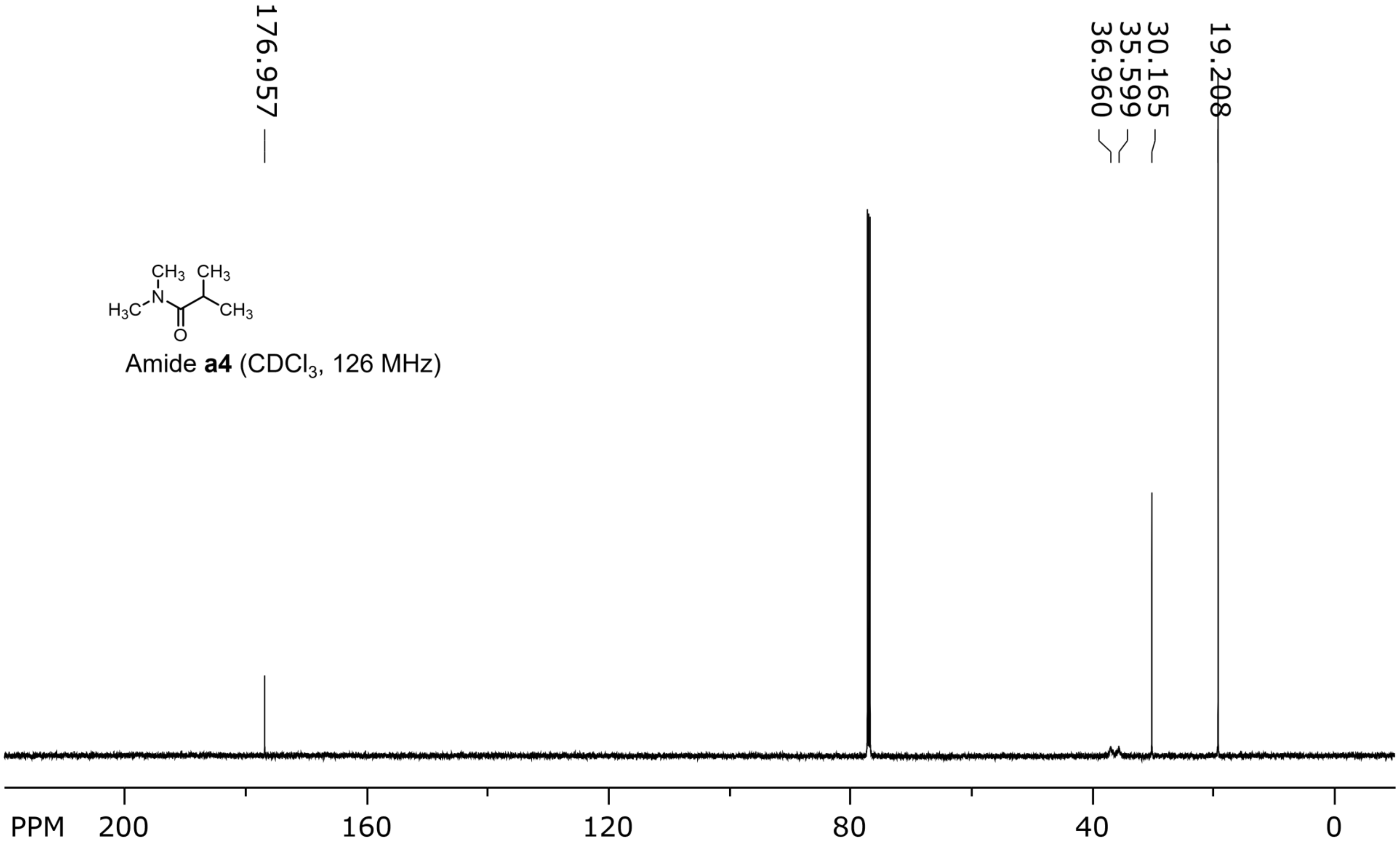

*^{1}H NMR for amide **a5***

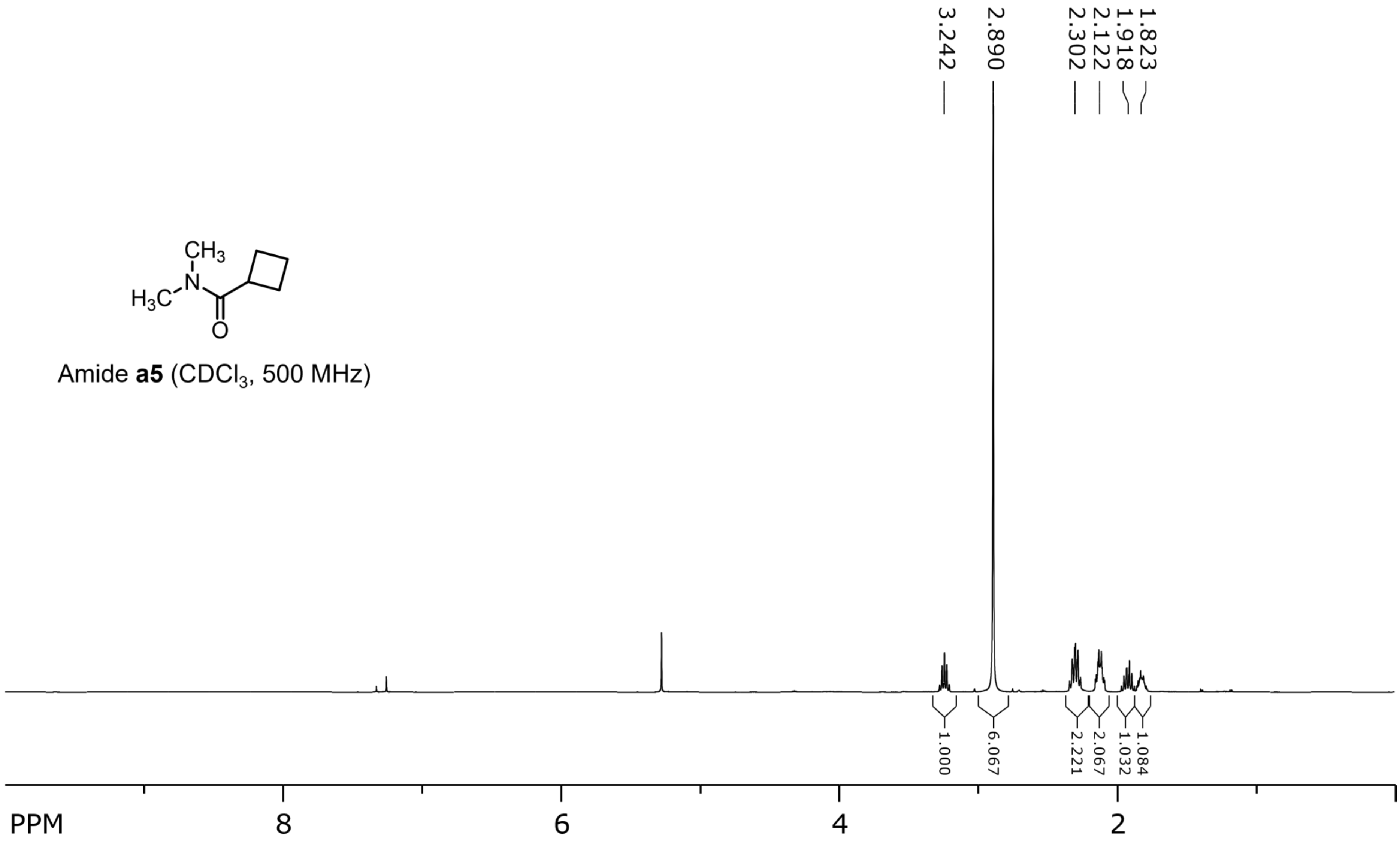

$^{13}C\{^{1}H\}$ NMR for amide ***a5***

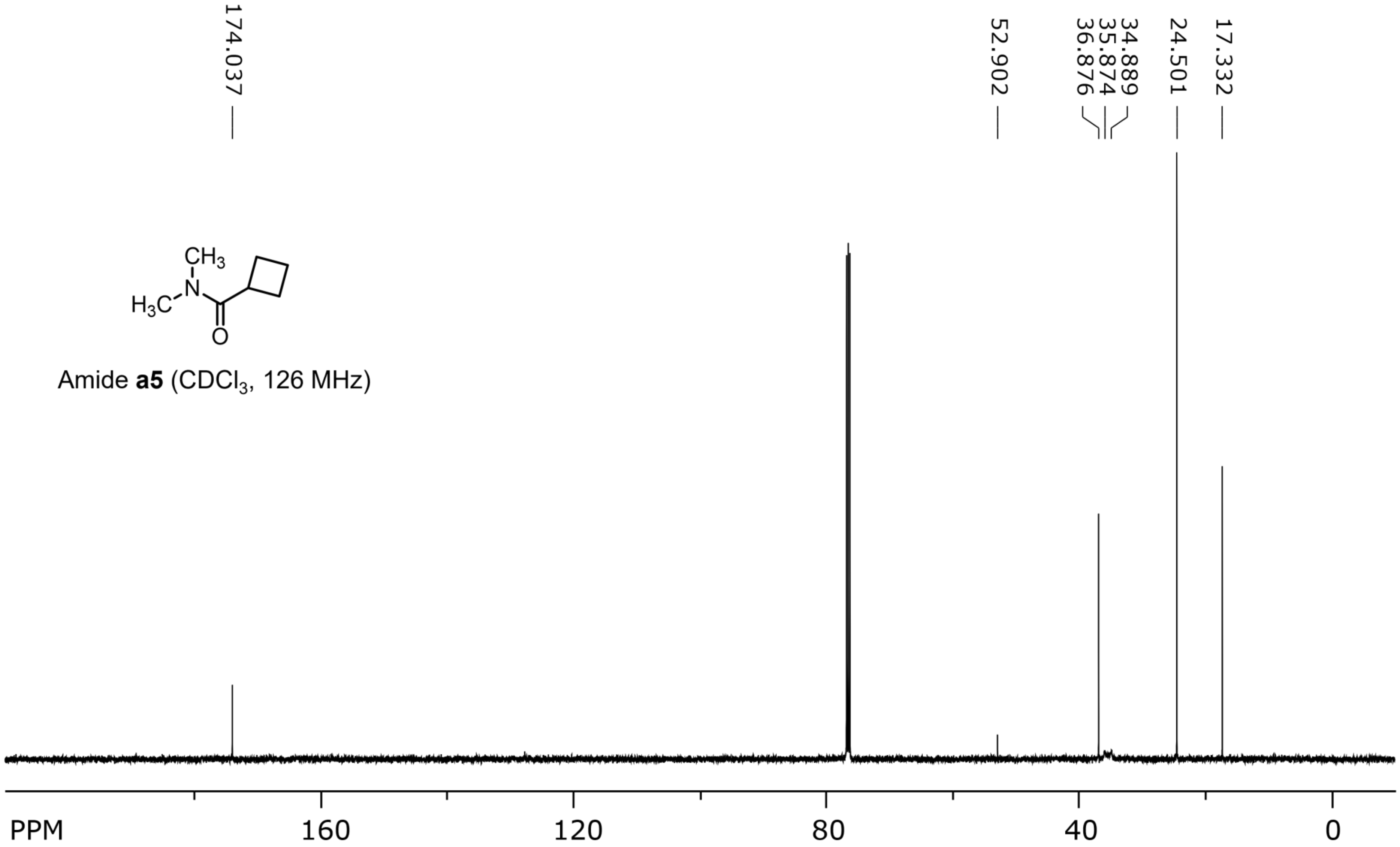

^{1}H NMR for amide ***a6***

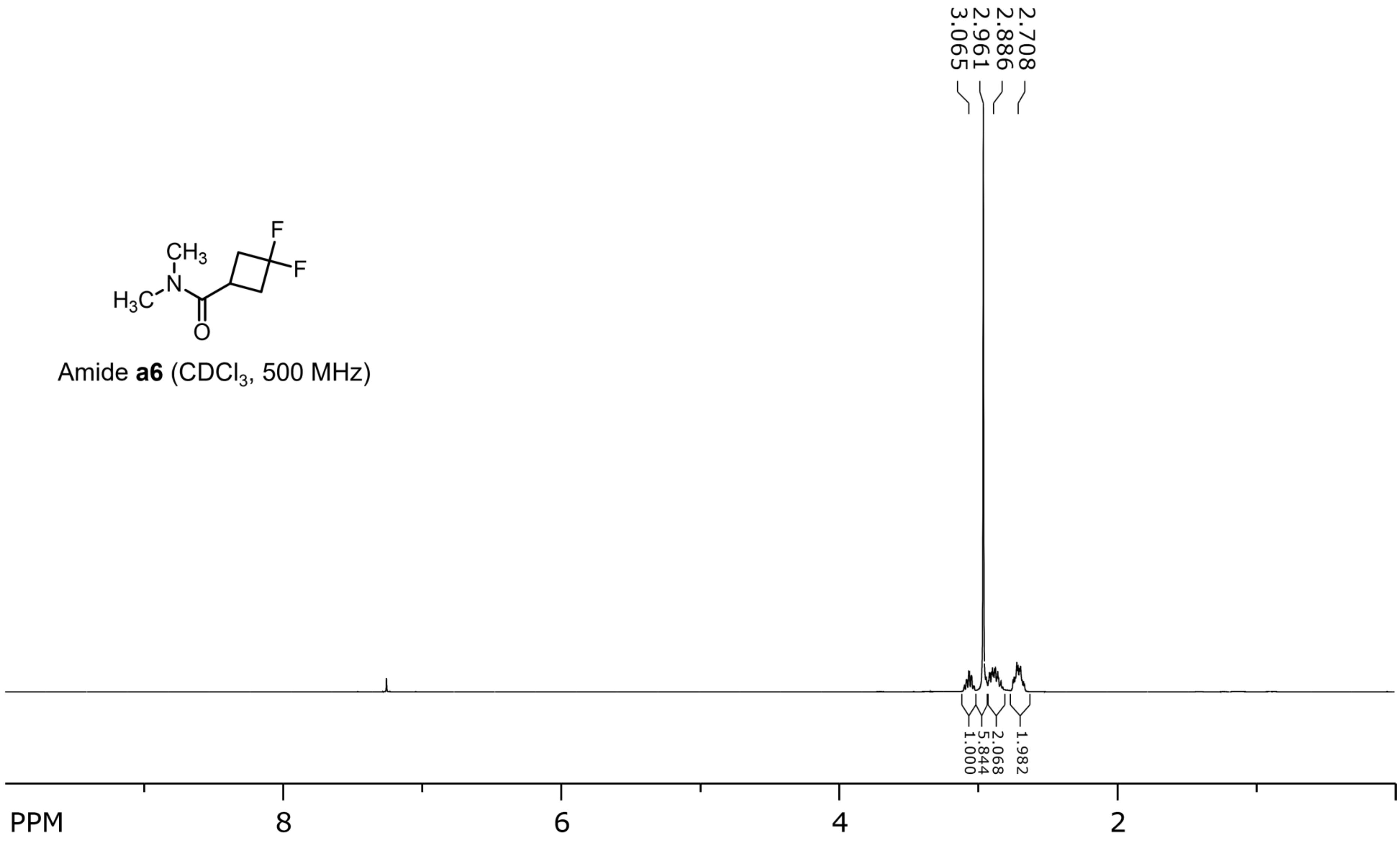

*^{13}C{^{1}H} NMR for amide **a6***

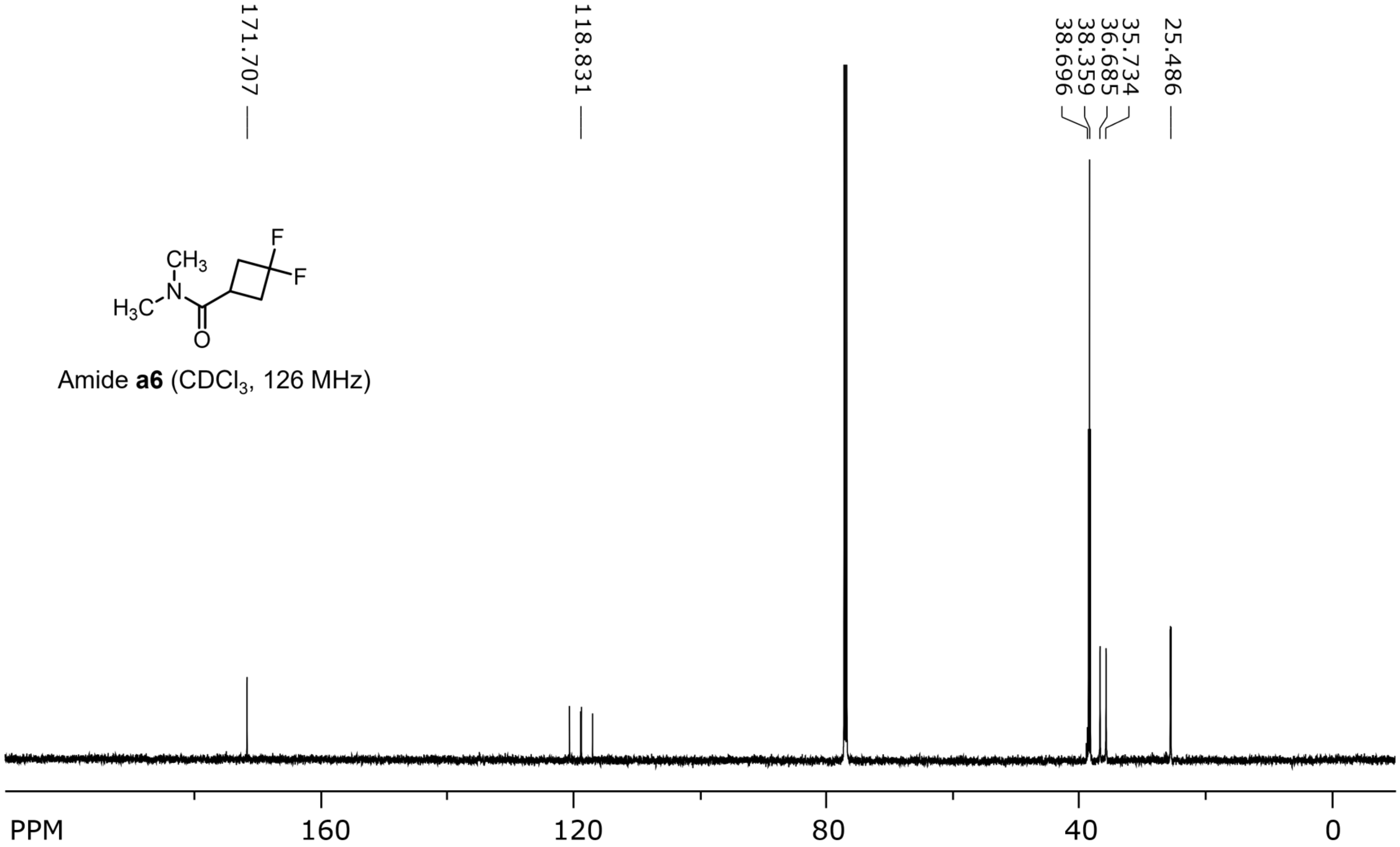

*$^{19}F\{^{1}H\}$ NMR for amide **a6***

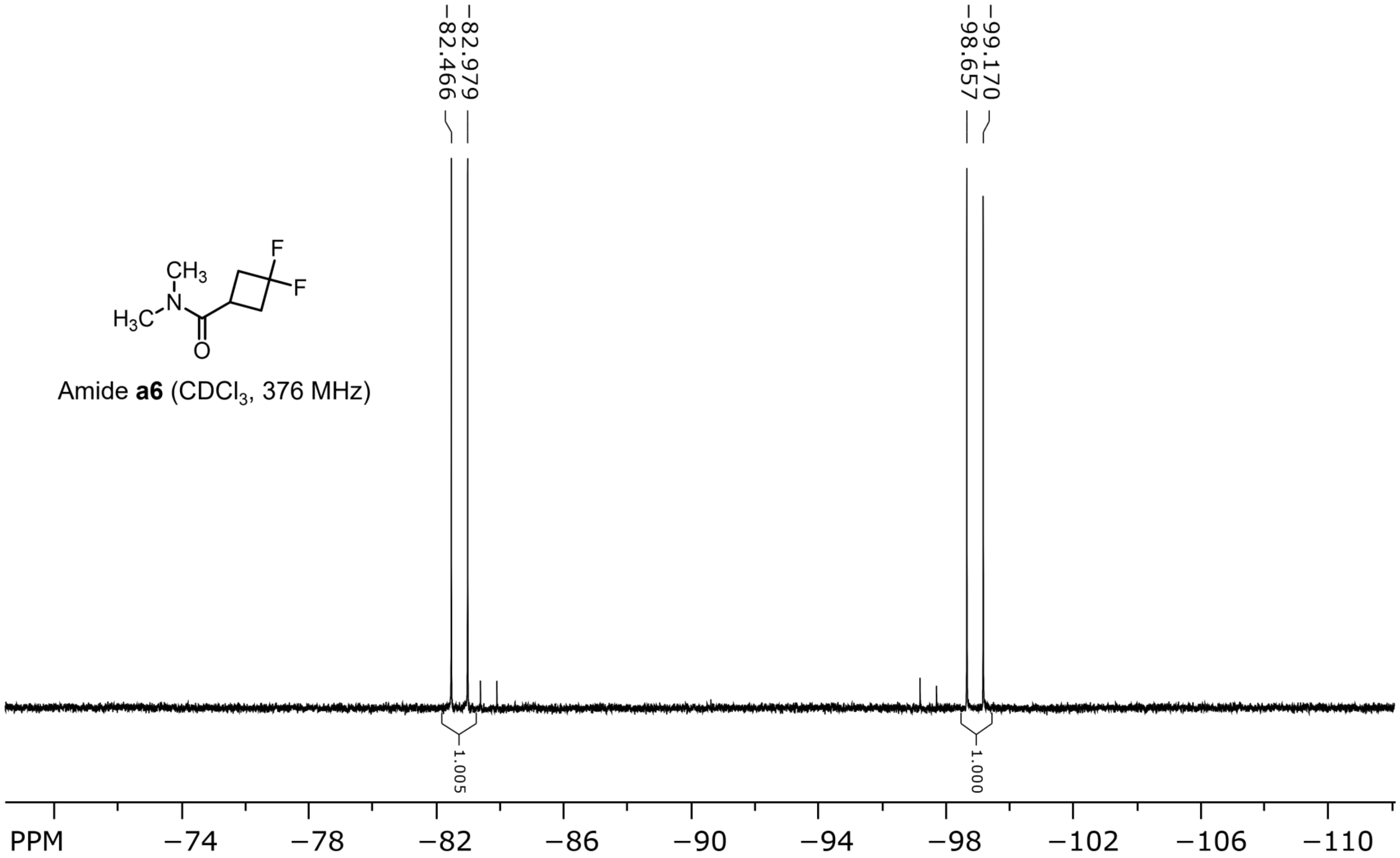

*^{1}H NMR for amide **a7***

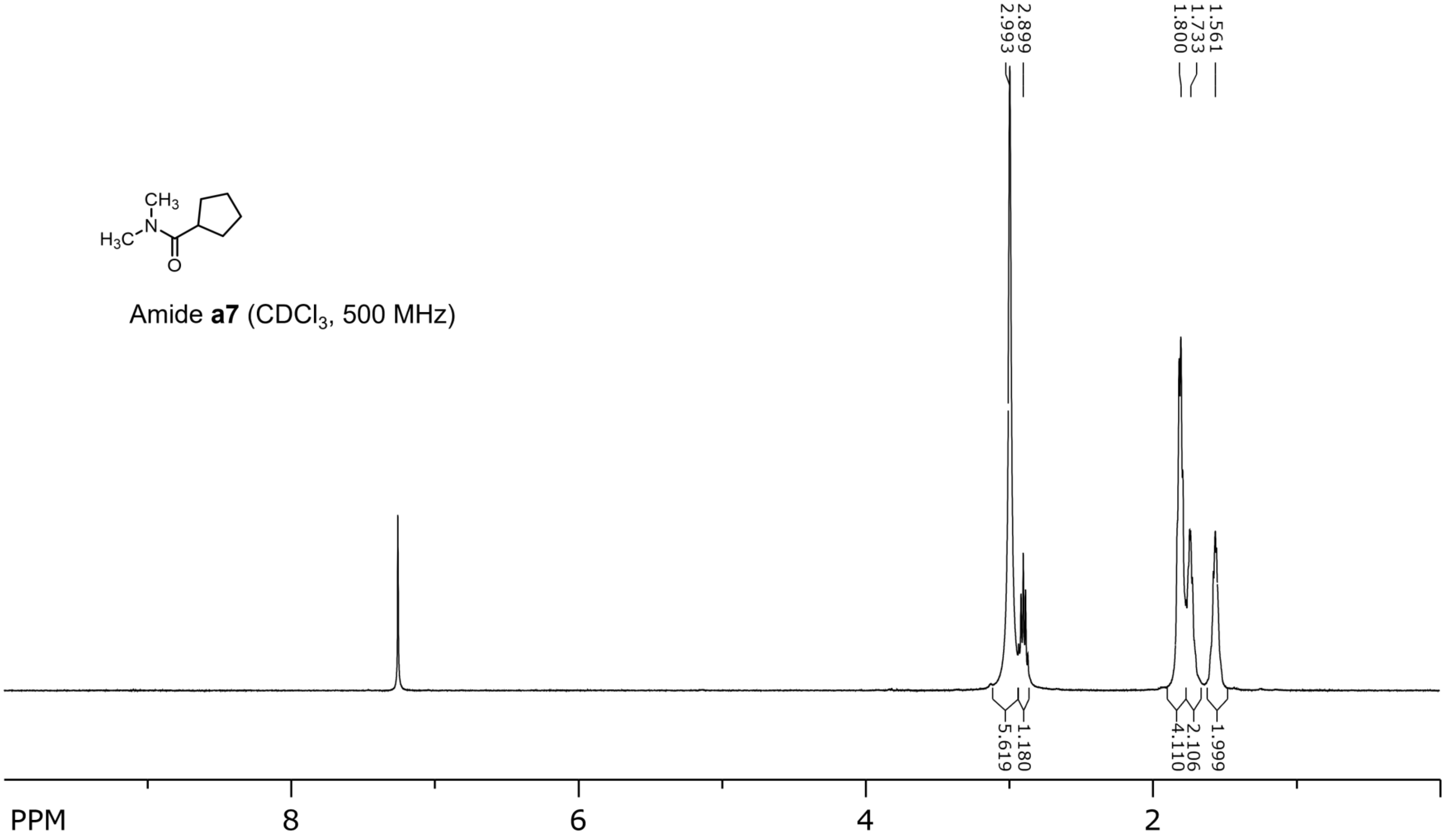

Amide **a7** ($CDCl_3$, 500 MHz)

$^{13}C\{^{1}H\}$ *NMR for amide* ***a7***

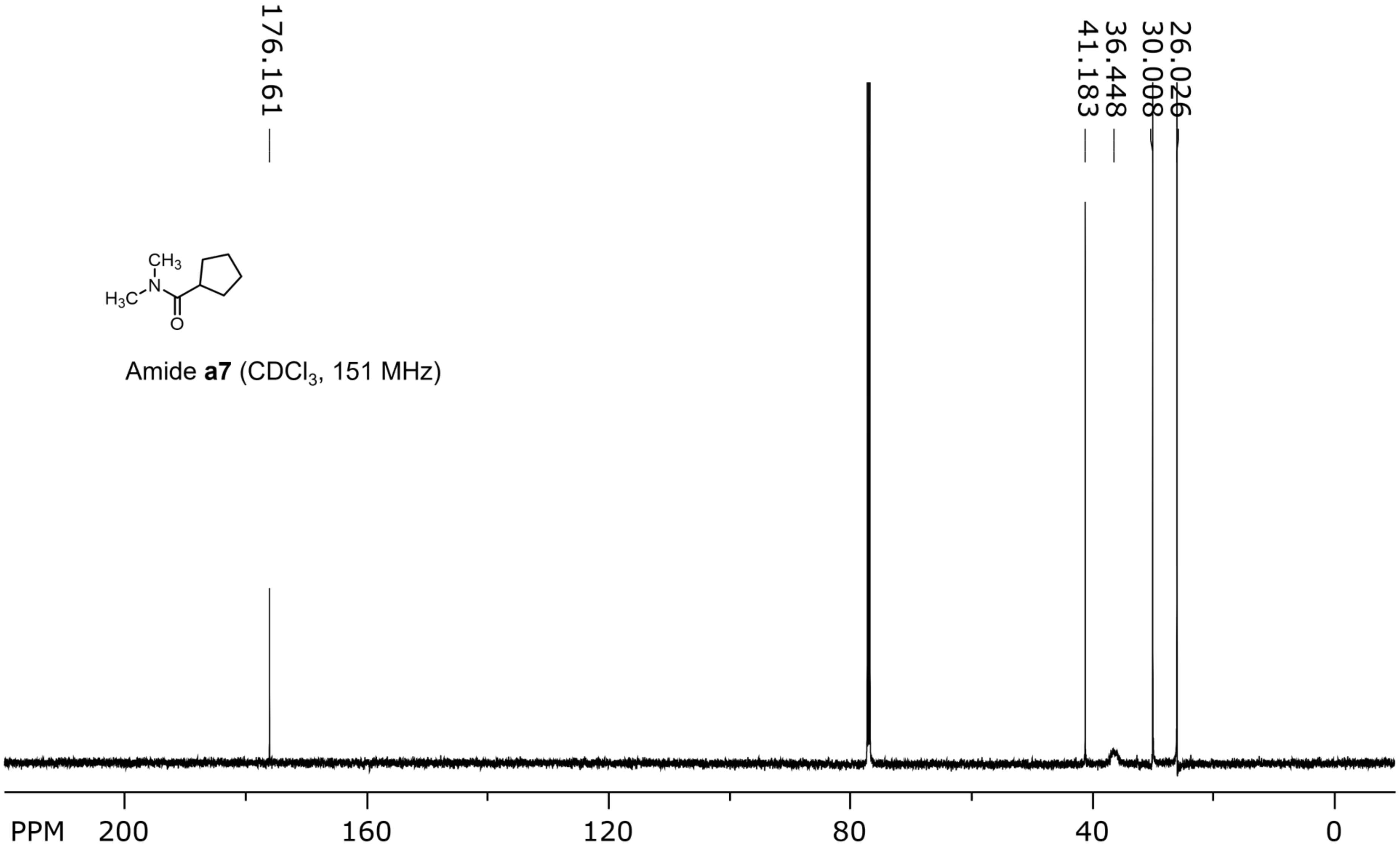

*^{1}H NMR for amide **a8***

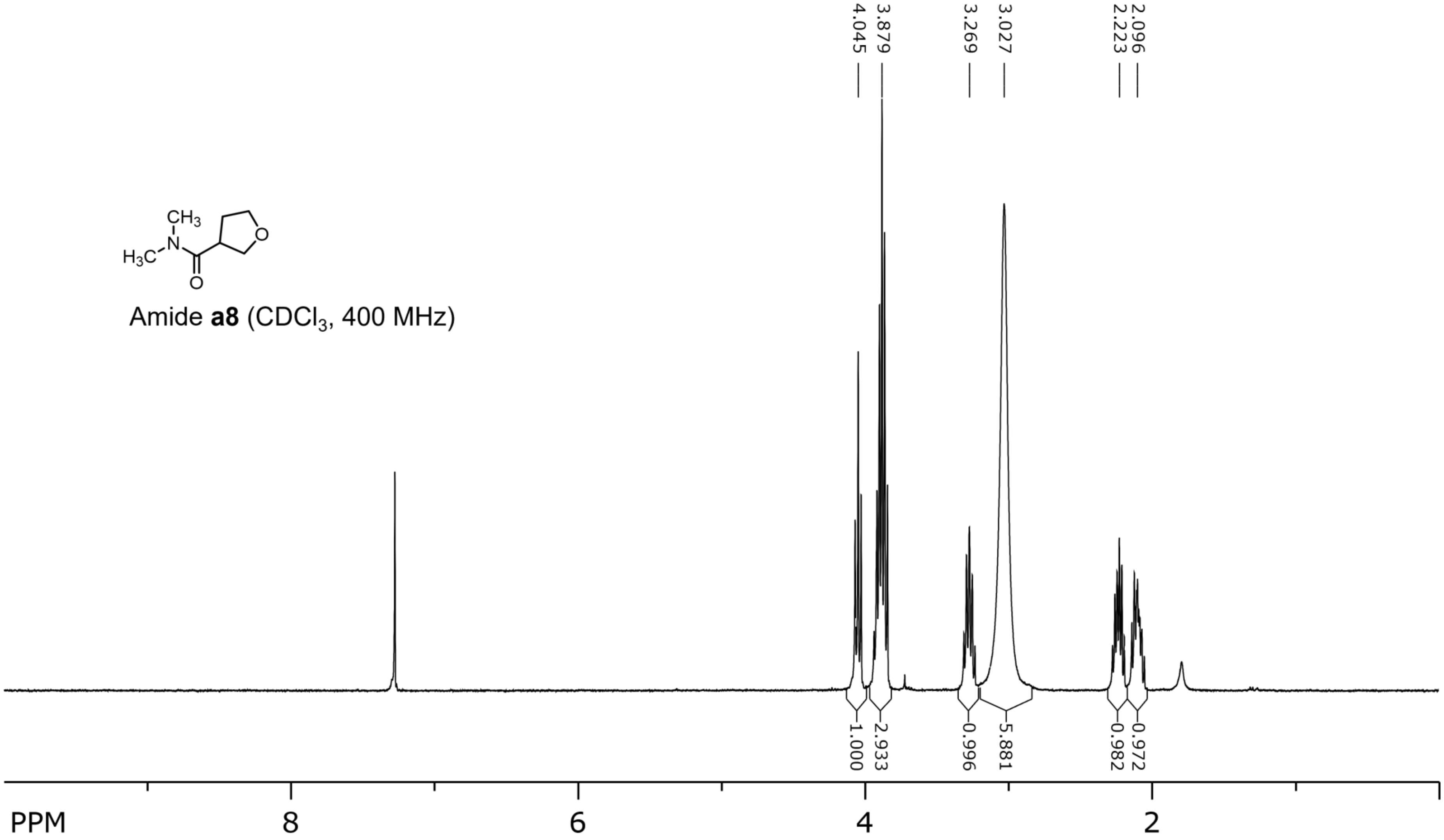

$^{13}C\{^1H\}$ *NMR for amide* ***a8***

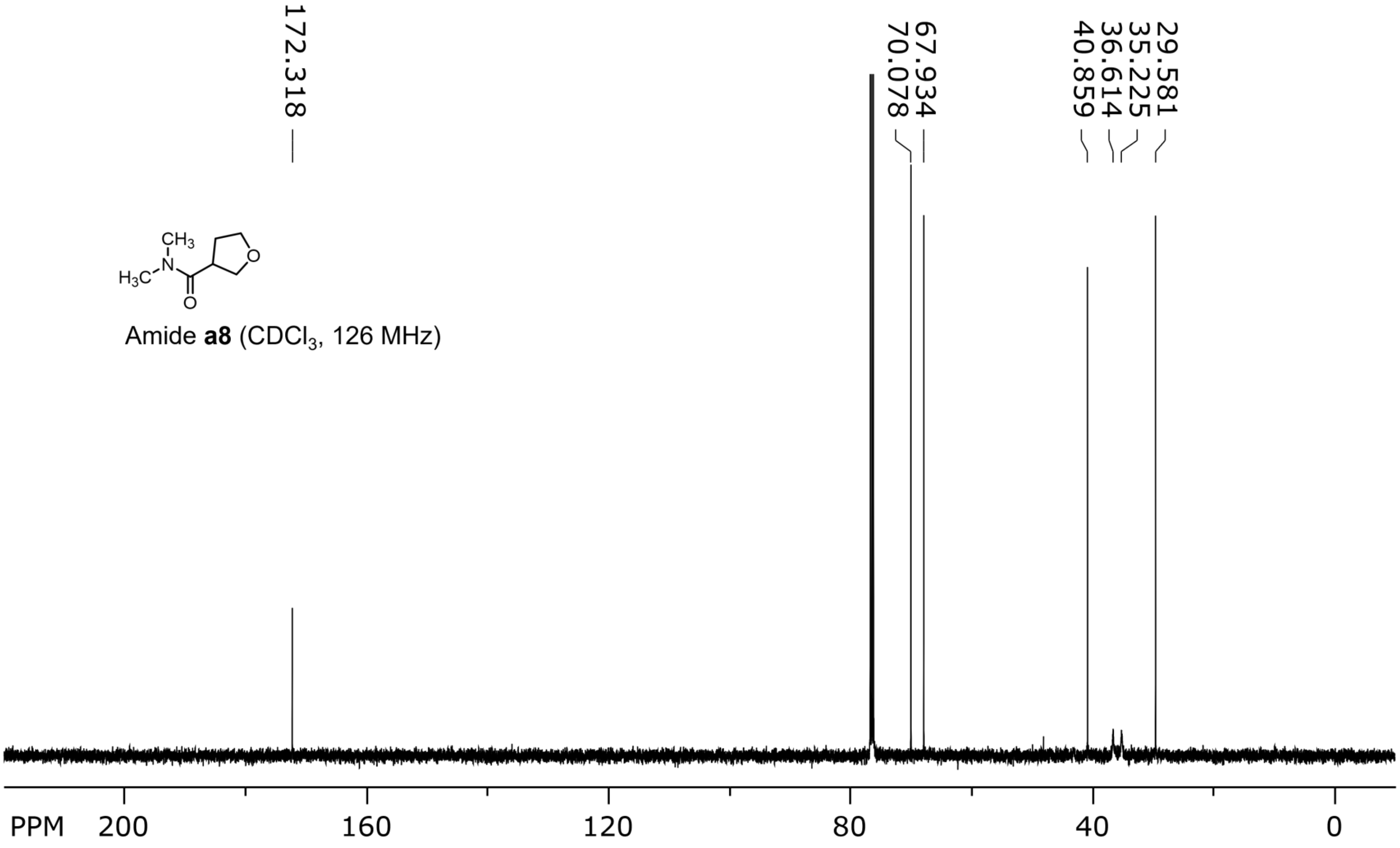

^{1}H NMR for amide ***a9***

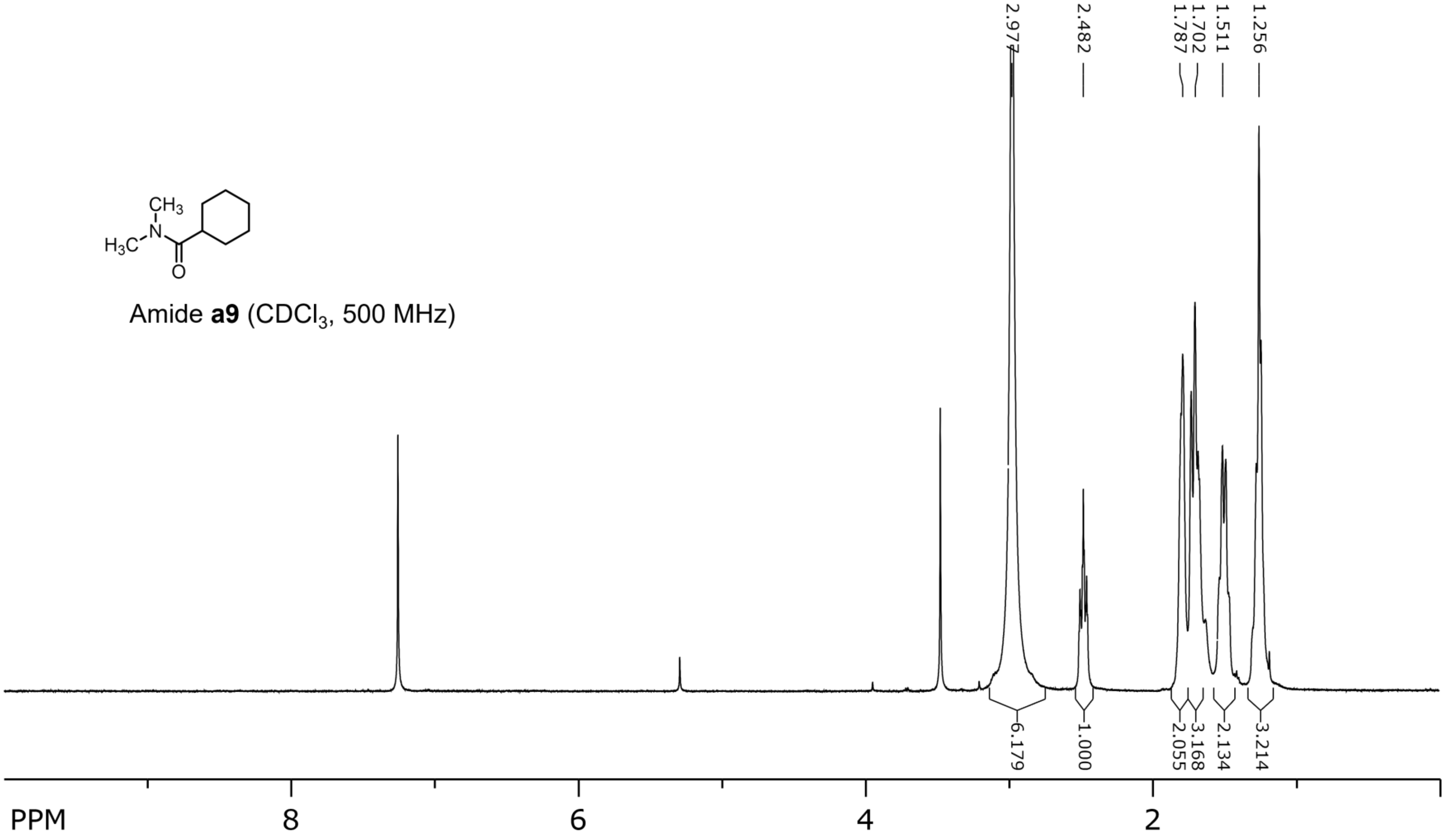

*$^{13}C\{^{1}H\}$ NMR for amide **a9***

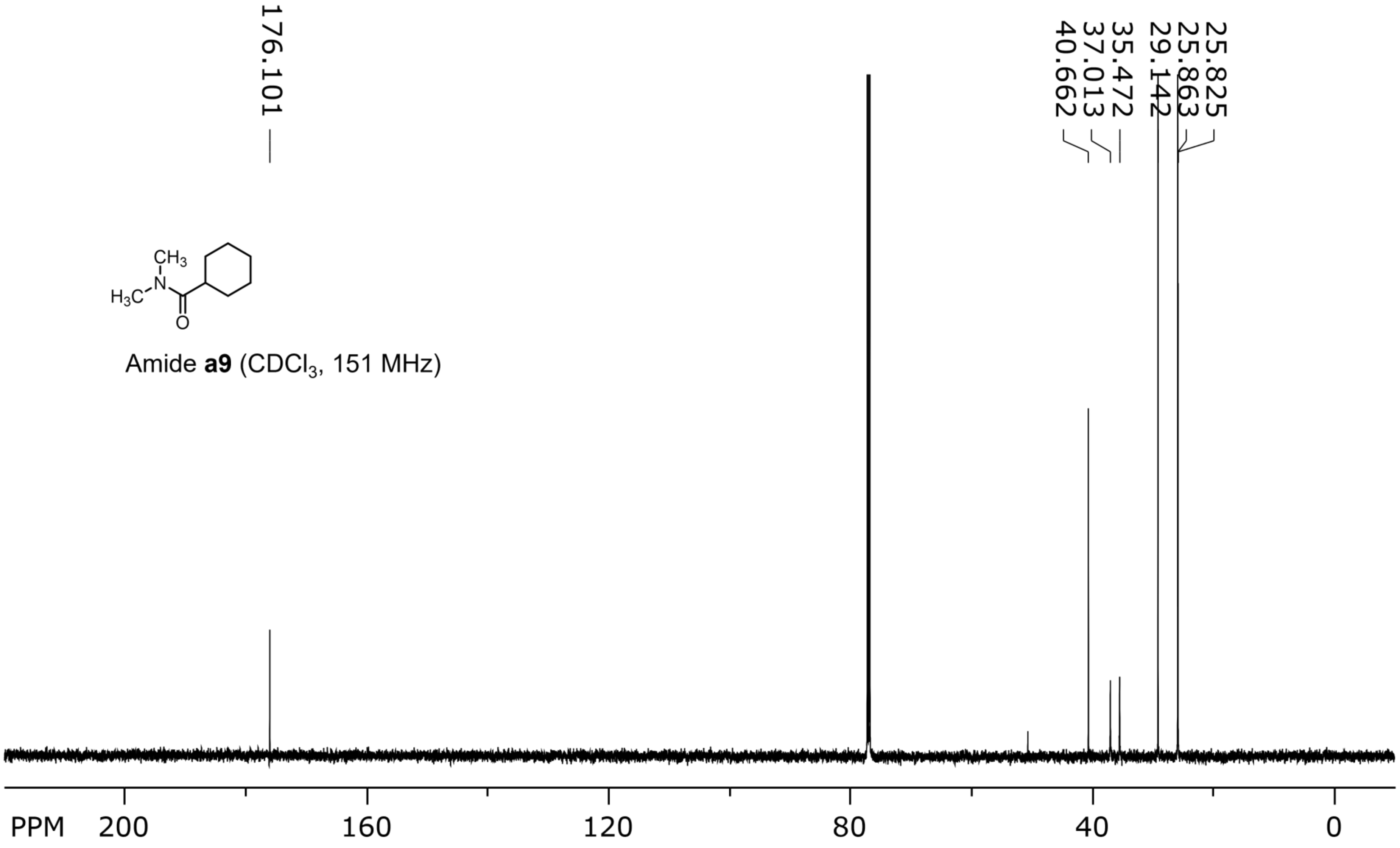

*^{1}H NMR for amide **a10***

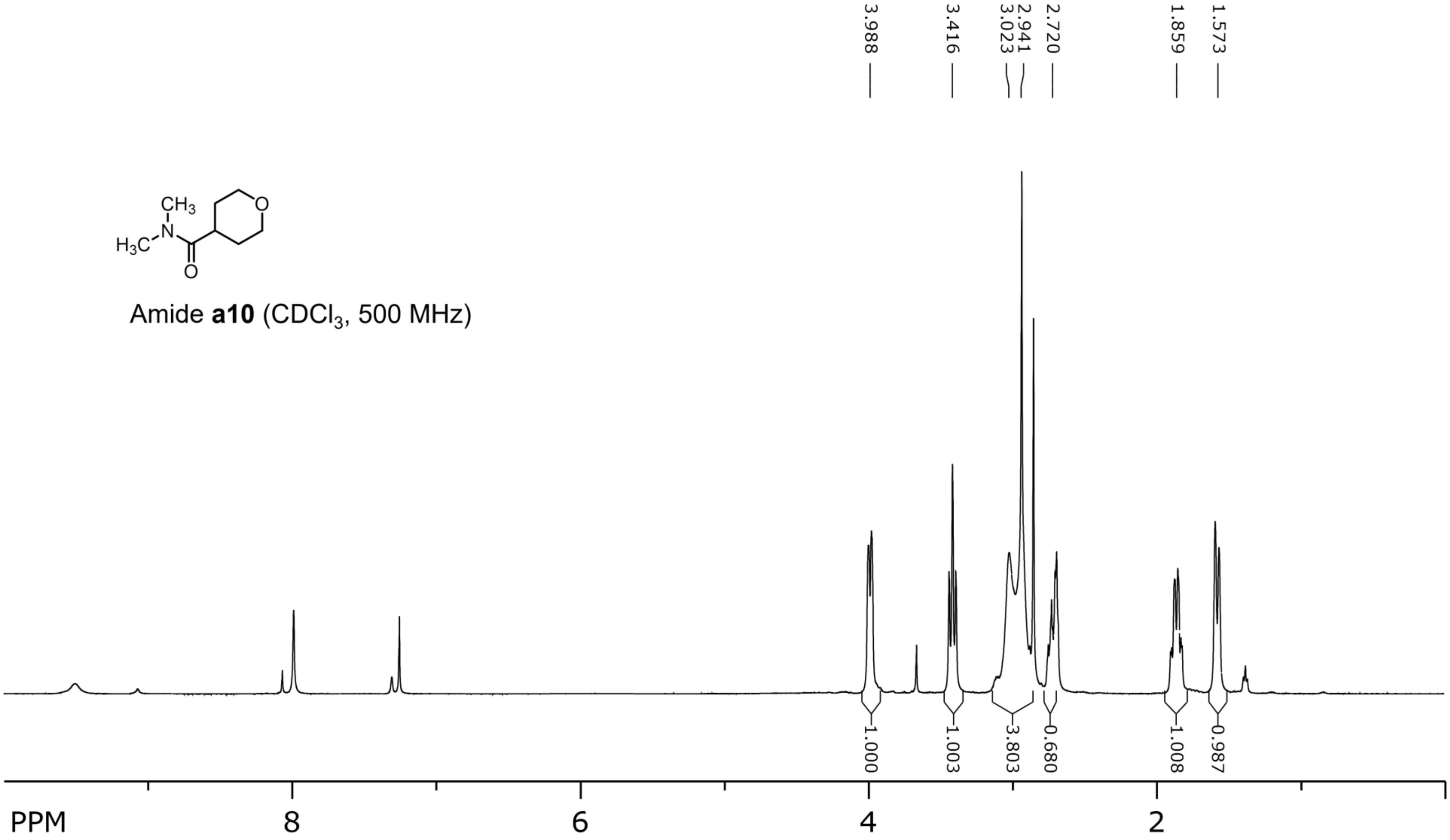

^{13}C{^{1}H} NMR for amide ***a10***

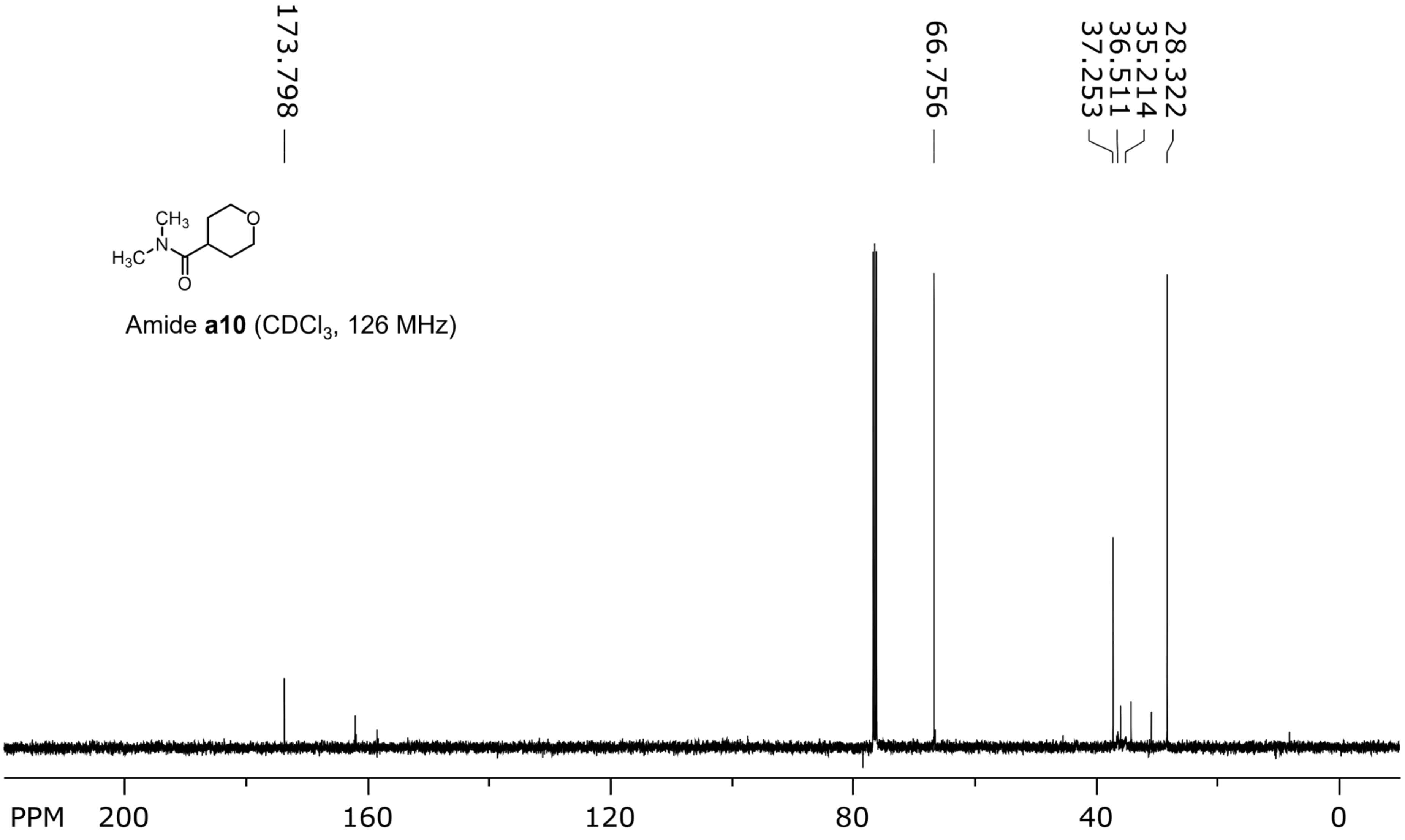

^{1}H NMR for amide ***a11***

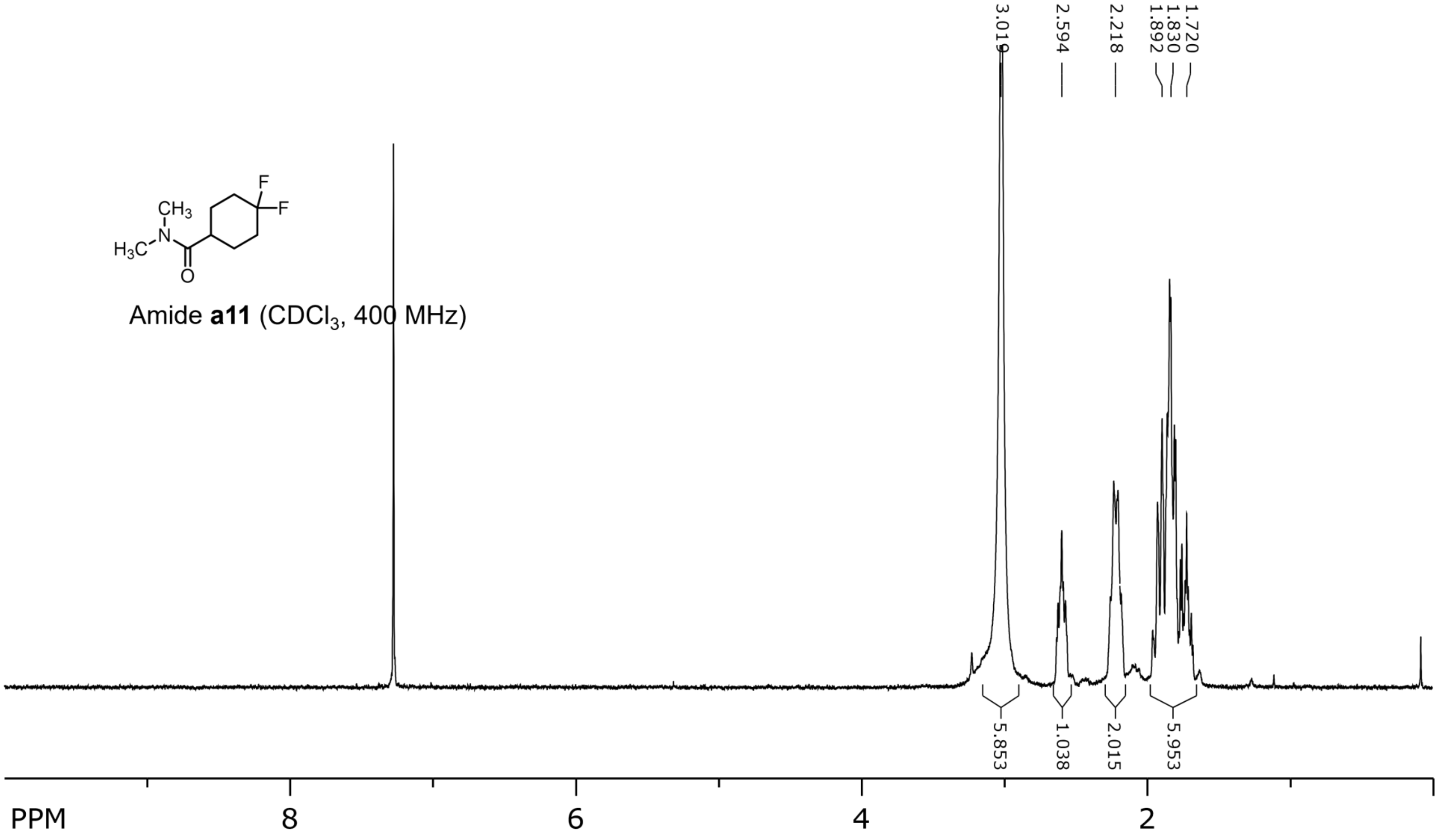

$^{13}C\{^{1}H\}$ *NMR for amide* ***a11***

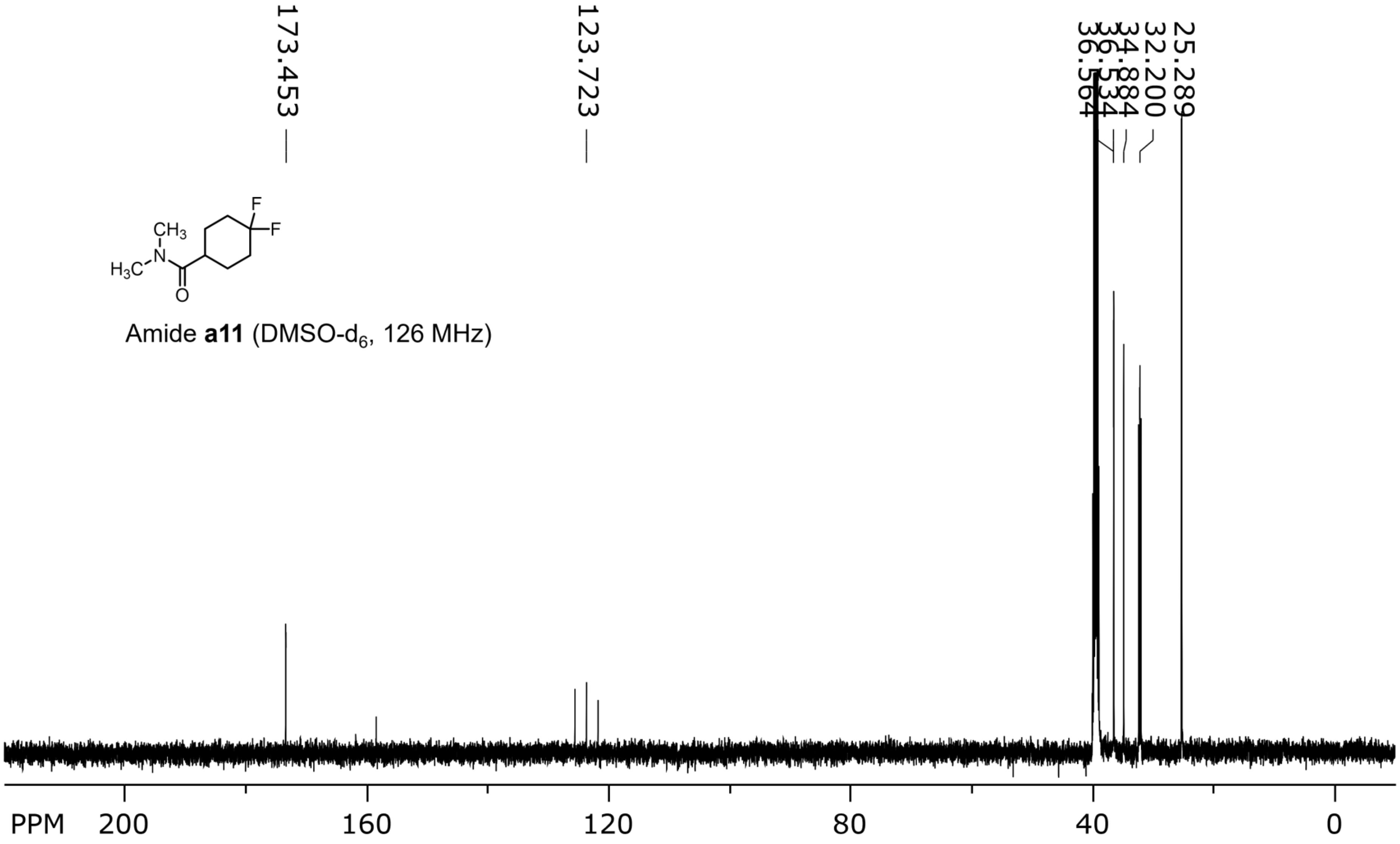

Amide **a11** (DMSO-d_6, 126 MHz)

*^{19}F{^{1}H} NMR for amide **a11***

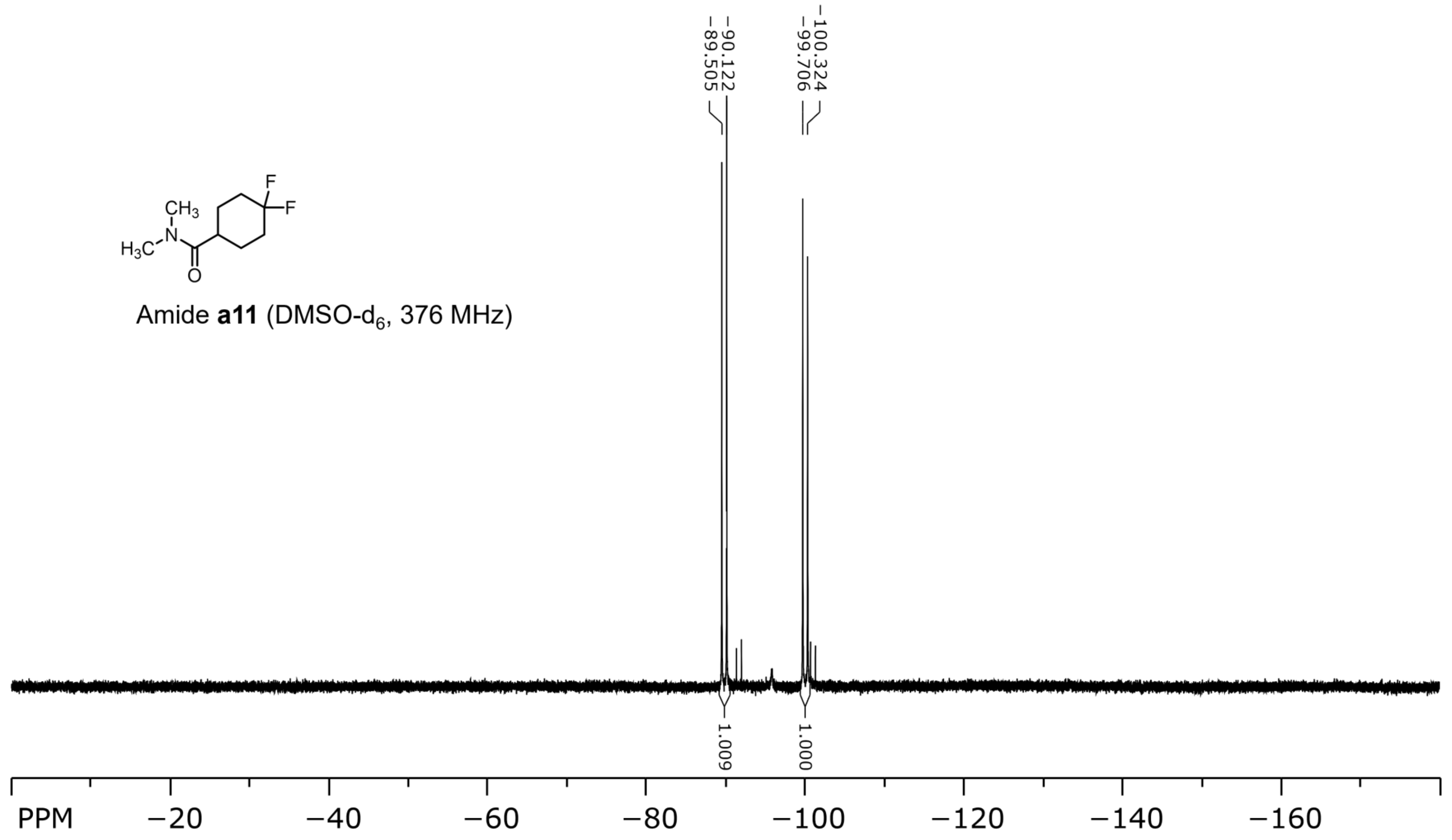

^{1}H NMR for amide ***a12***

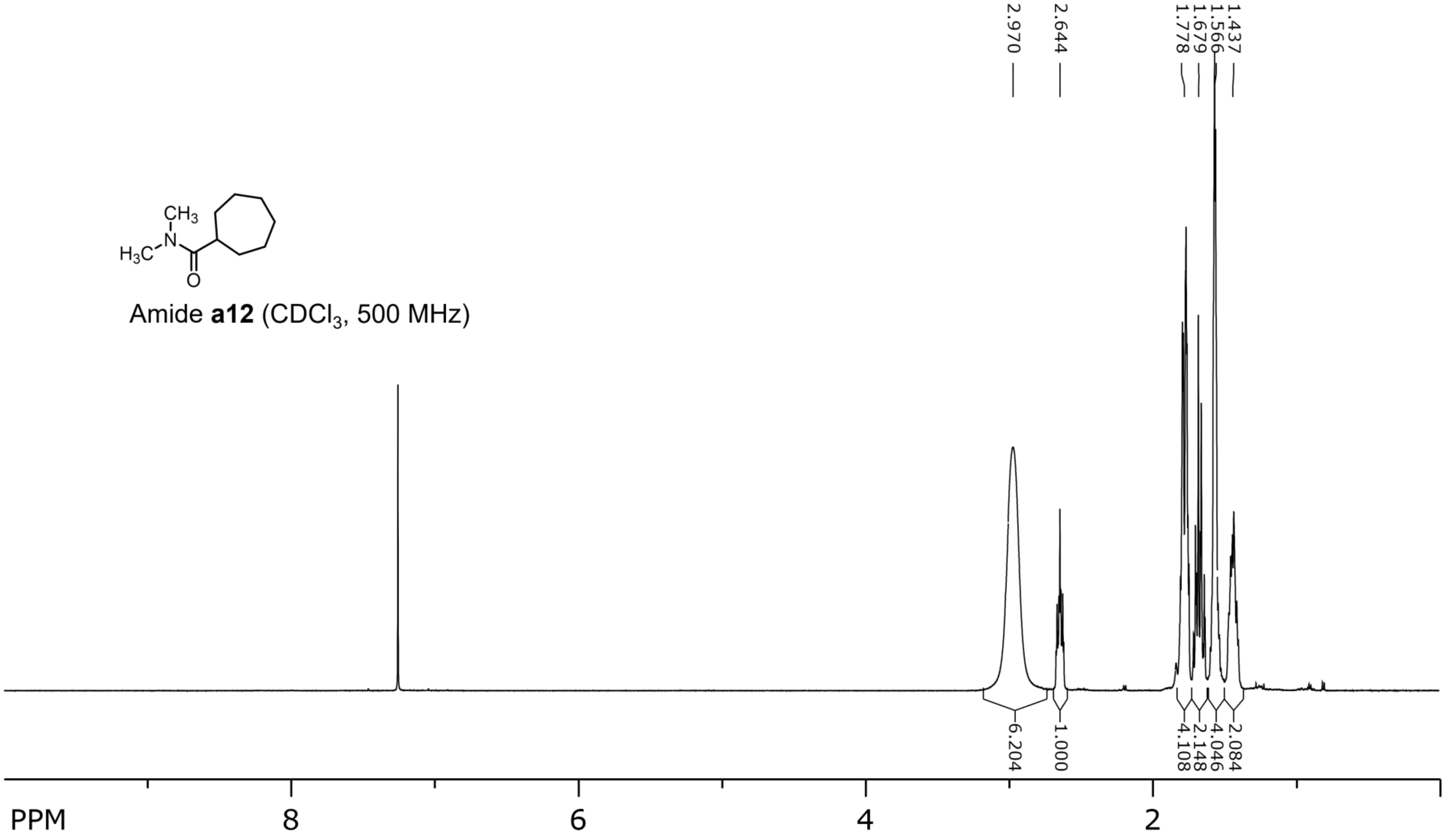

$^{13}C\{^{1}H\}$ *NMR for amide* ***a12***

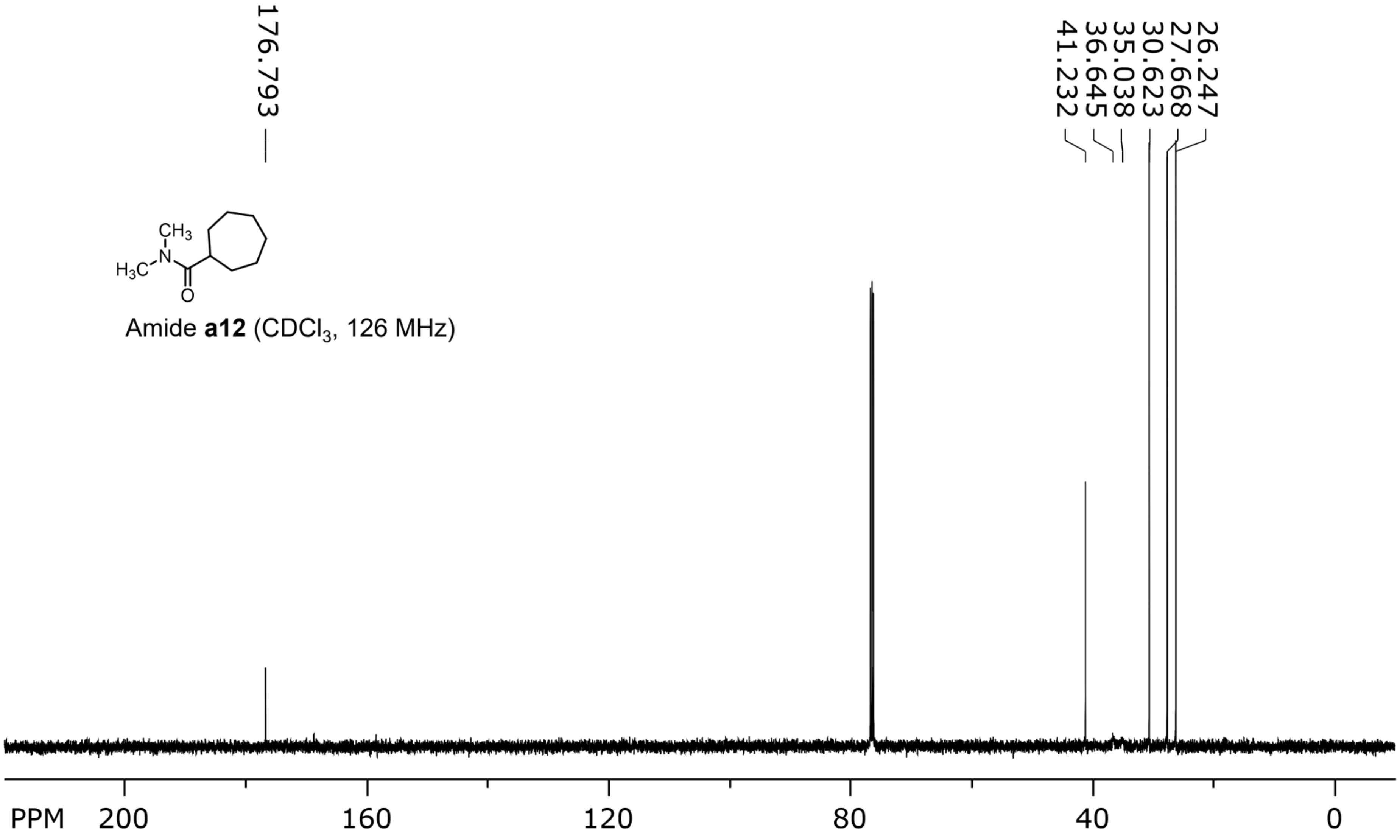

*^{1}H NMR for amide **a13***

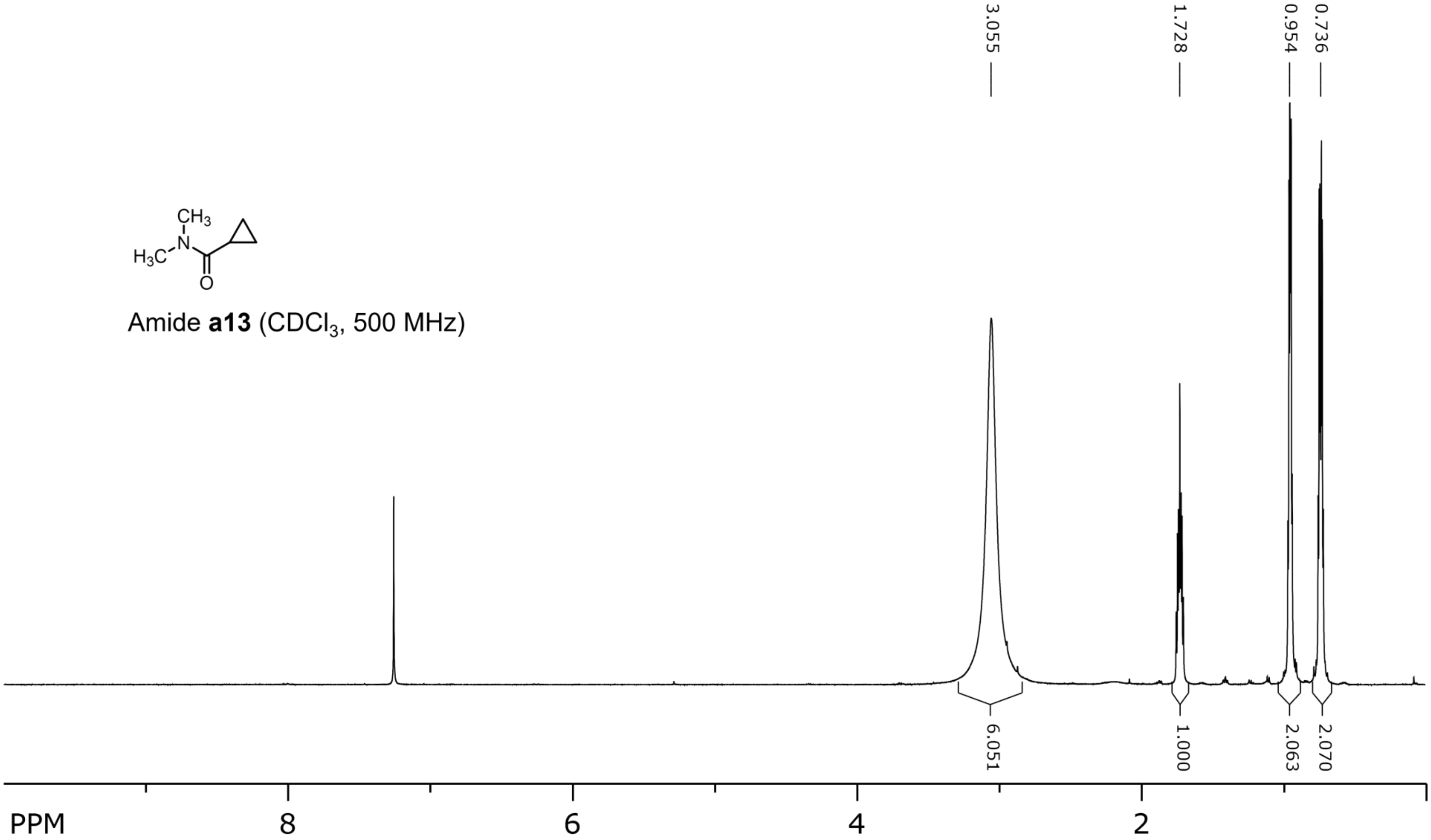

$^{13}C\{^1H\}$ *NMR for amide* ***a13***

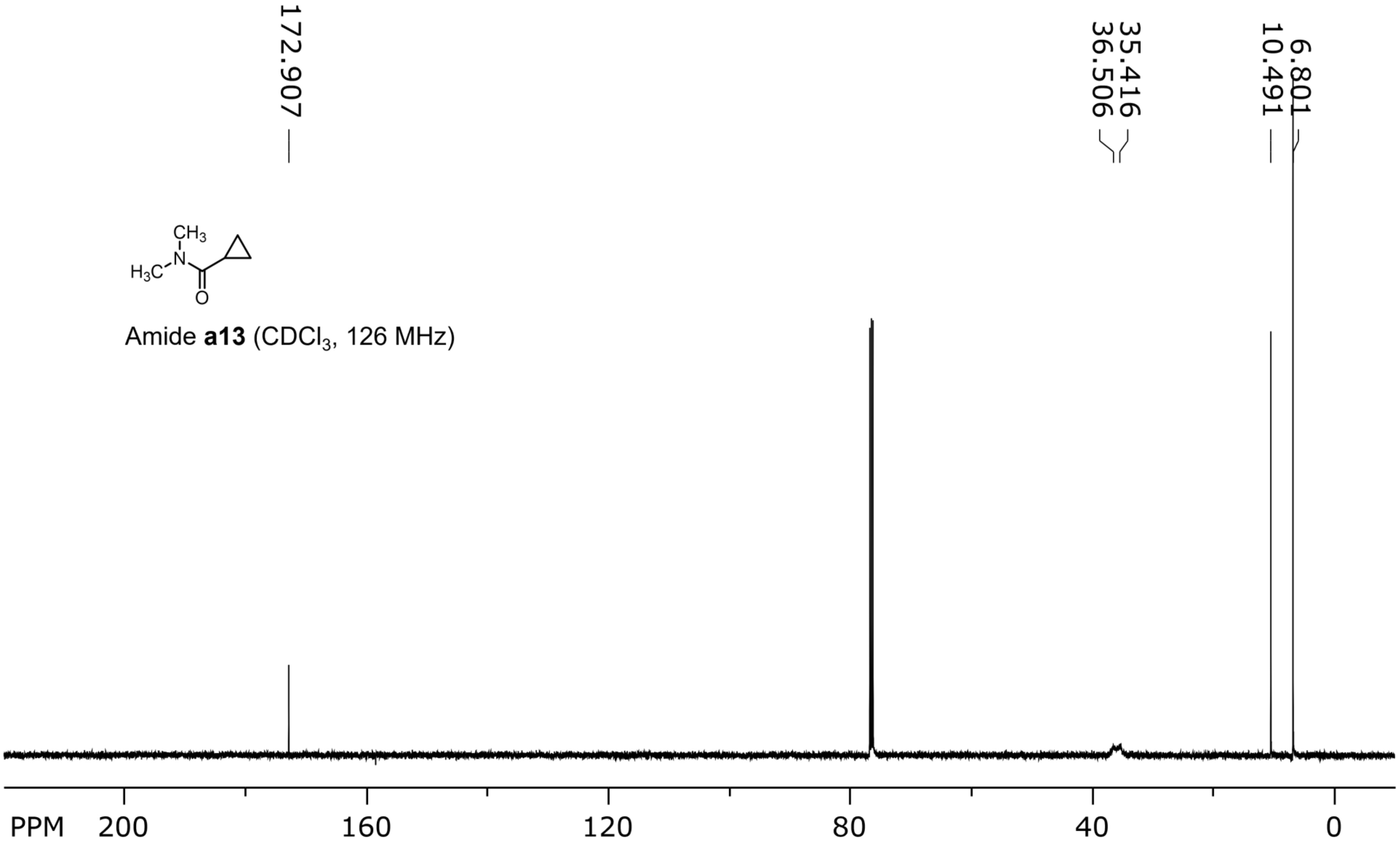

^{1}H NMR for amide ***a14***

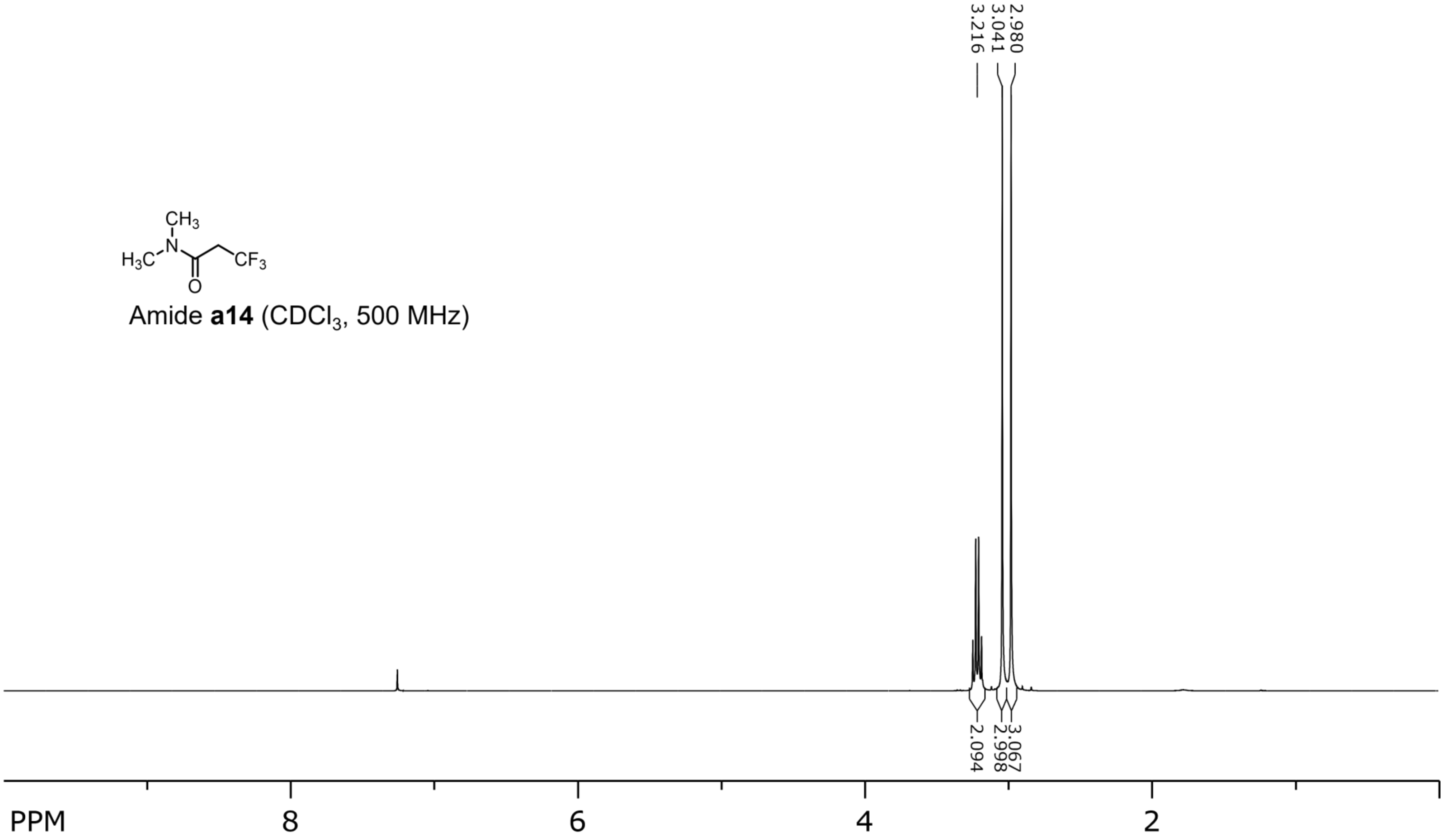

Amide **a14** ($CDCl_3$, 500 MHz)

*^{13}C{^{1}H} NMR for amide **a14***

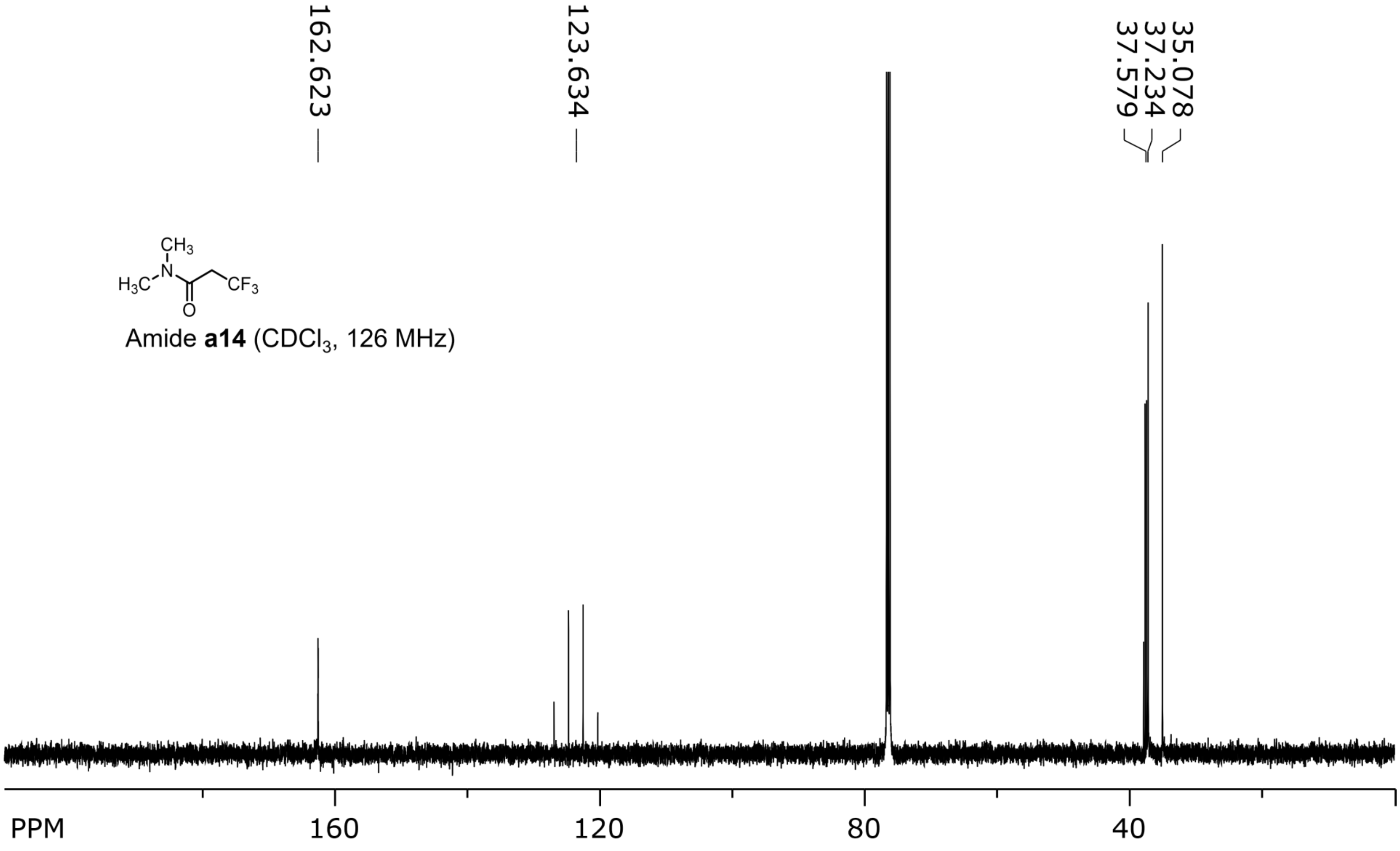

$^{19}F\{^{1}H\}$ *NMR for amide* ***a14***

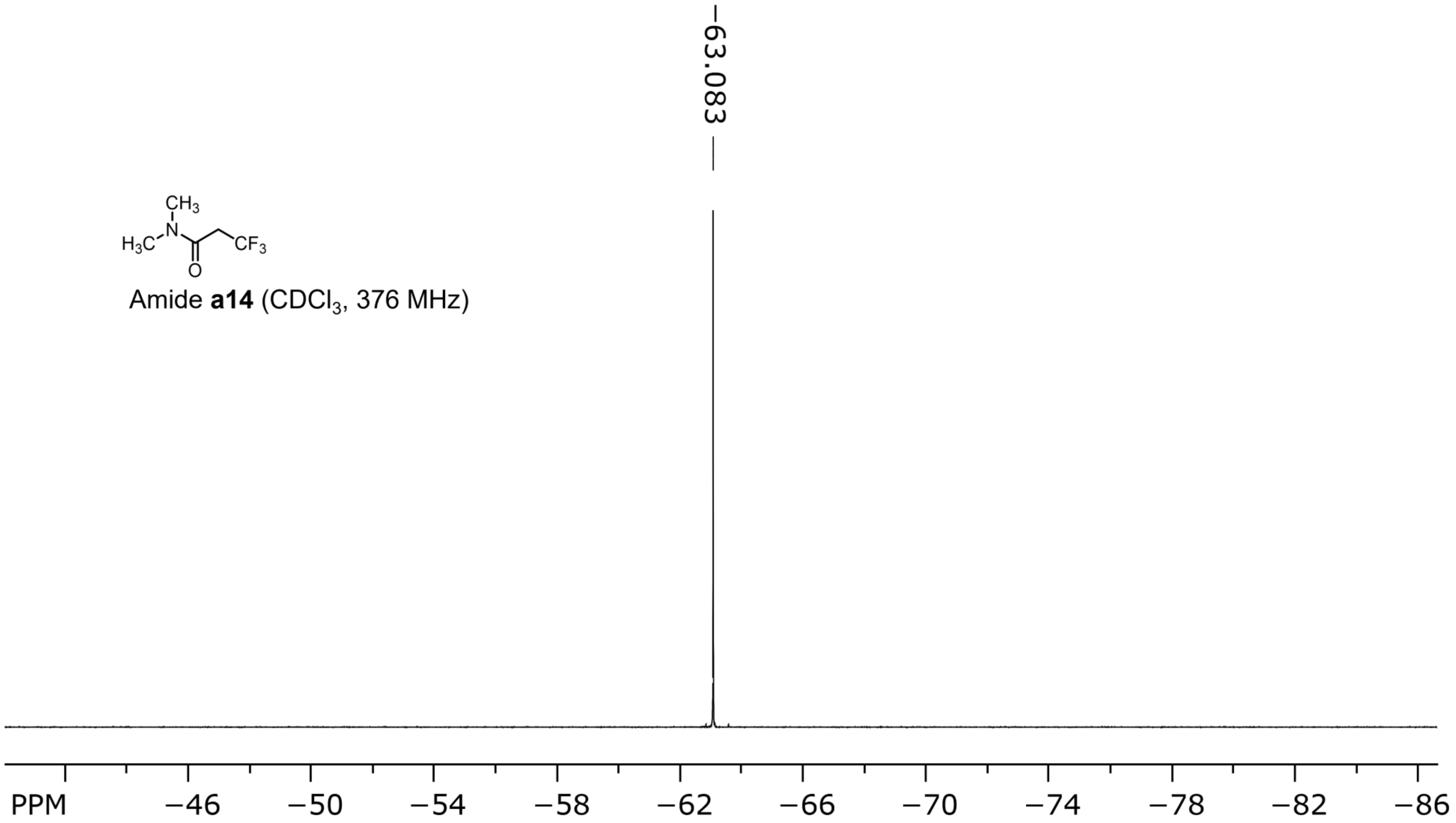

^{1}H *NMR for amide* ***a15***

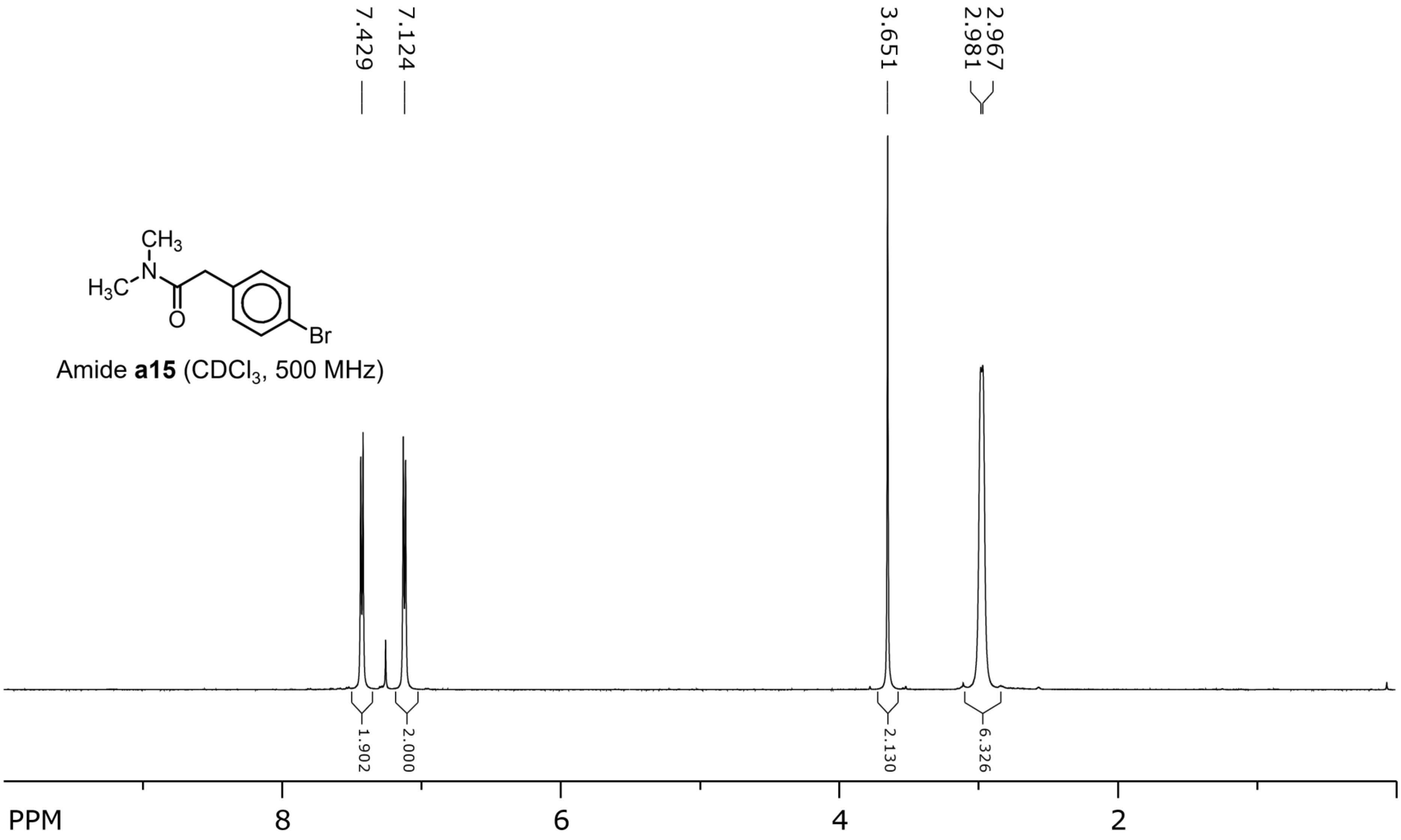

*$^{13}C\{^{1}H\}$ NMR for amide **a15***

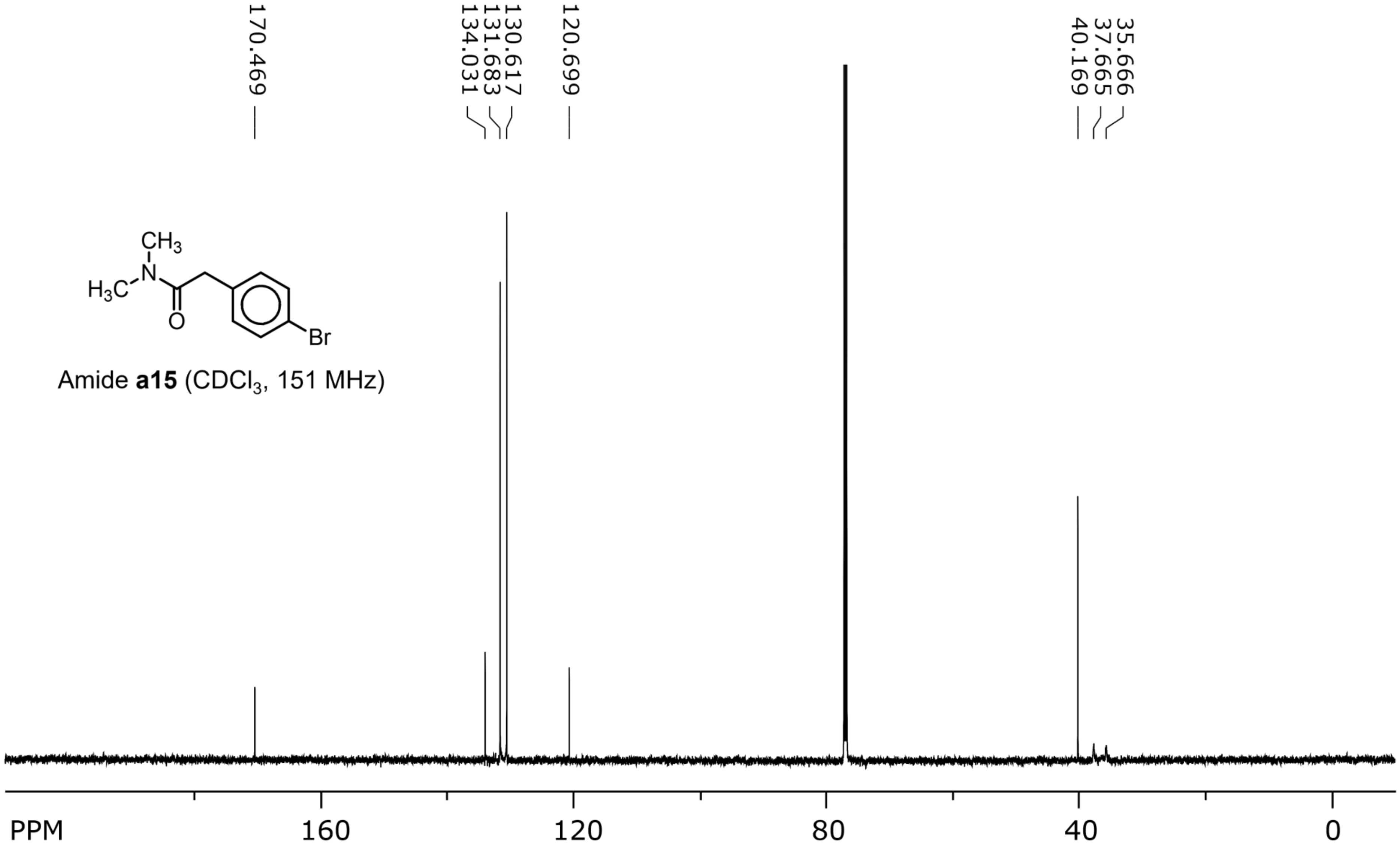

*^{1}H NMR for amide **a16***

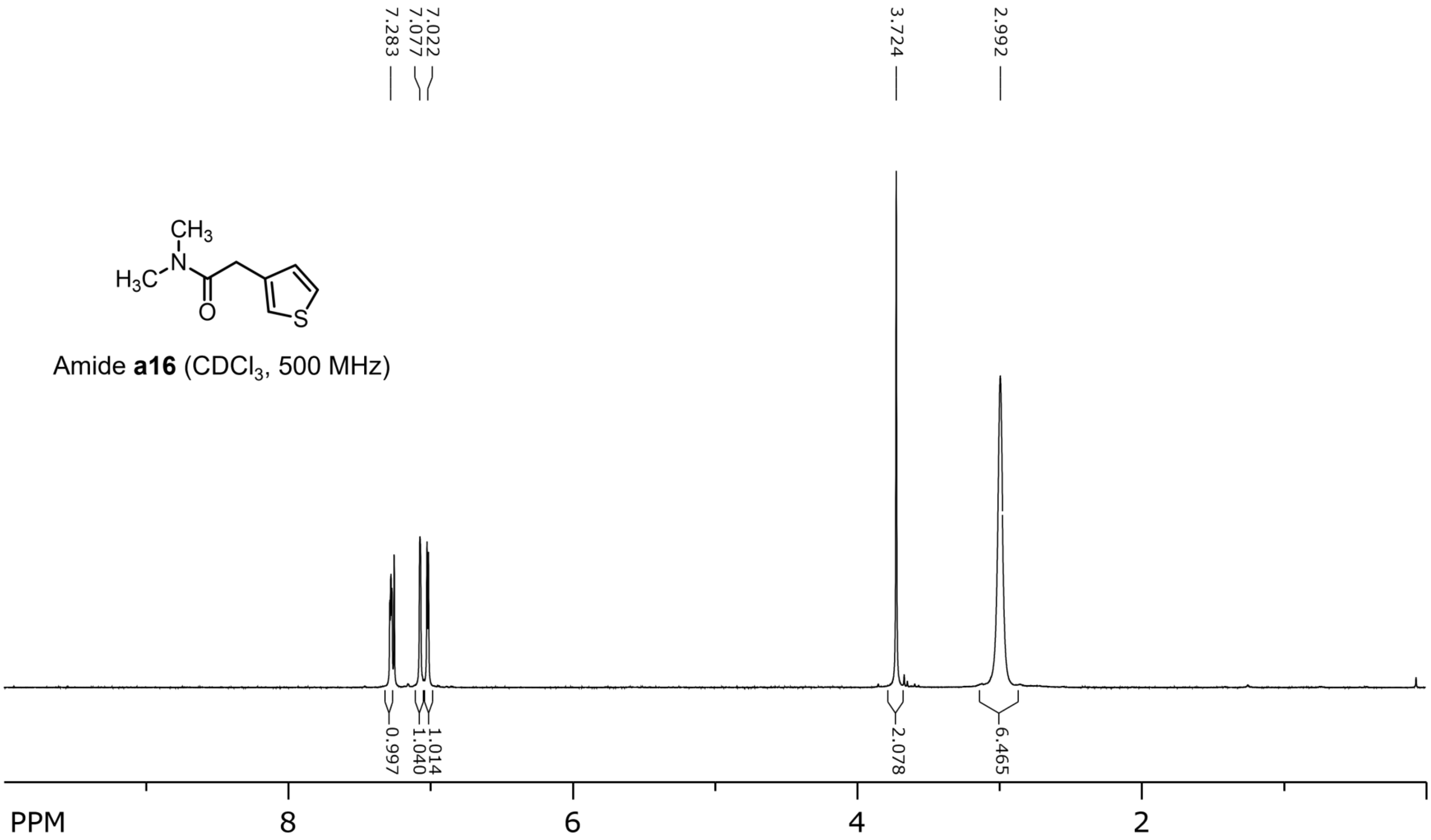

$^{13}C\{^{1}H\}$ NMR for amide ***a16***

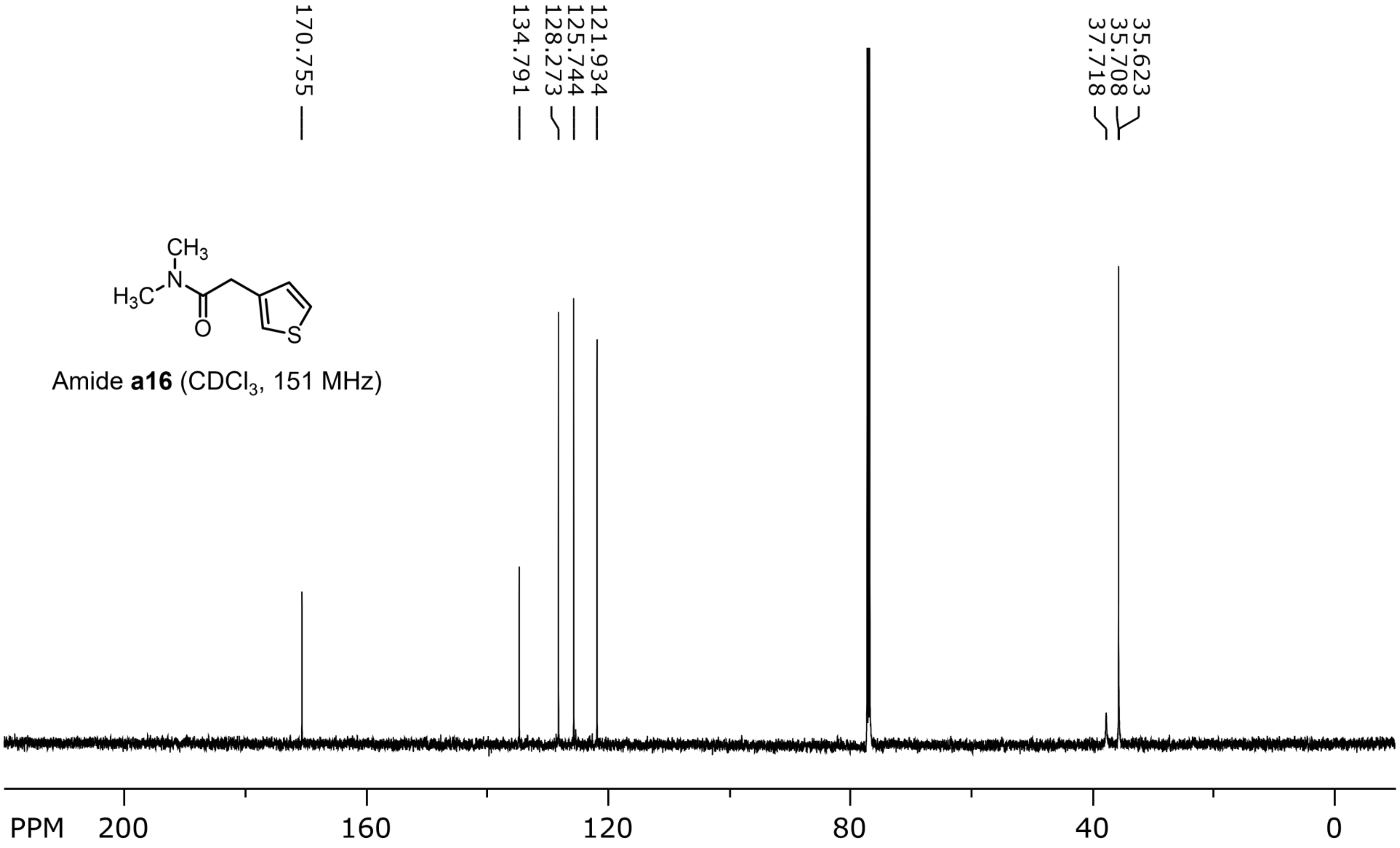

^{1}H NMR for amide ***a17***

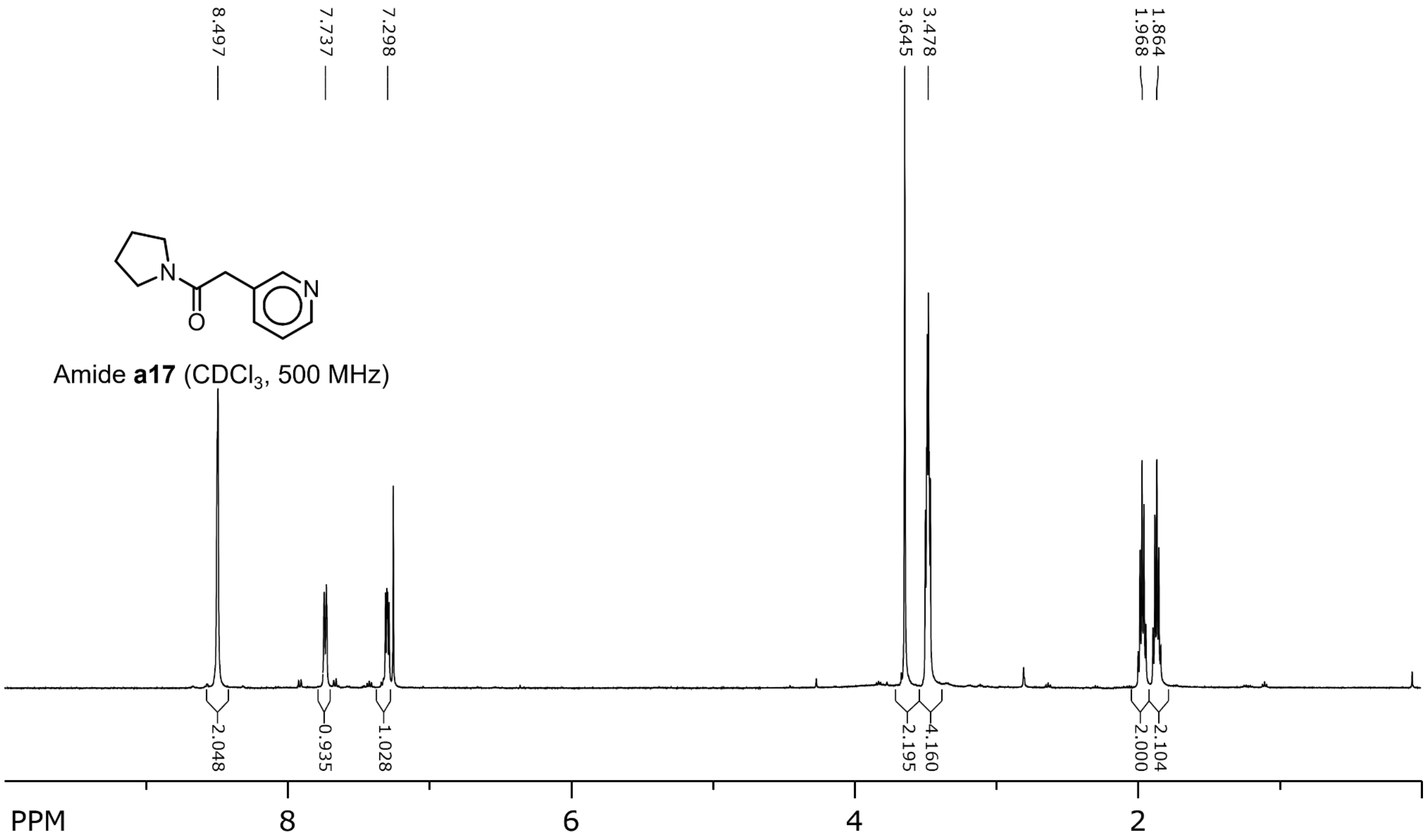

*$^{13}C\{^{1}H\}$ NMR for amide **a17***

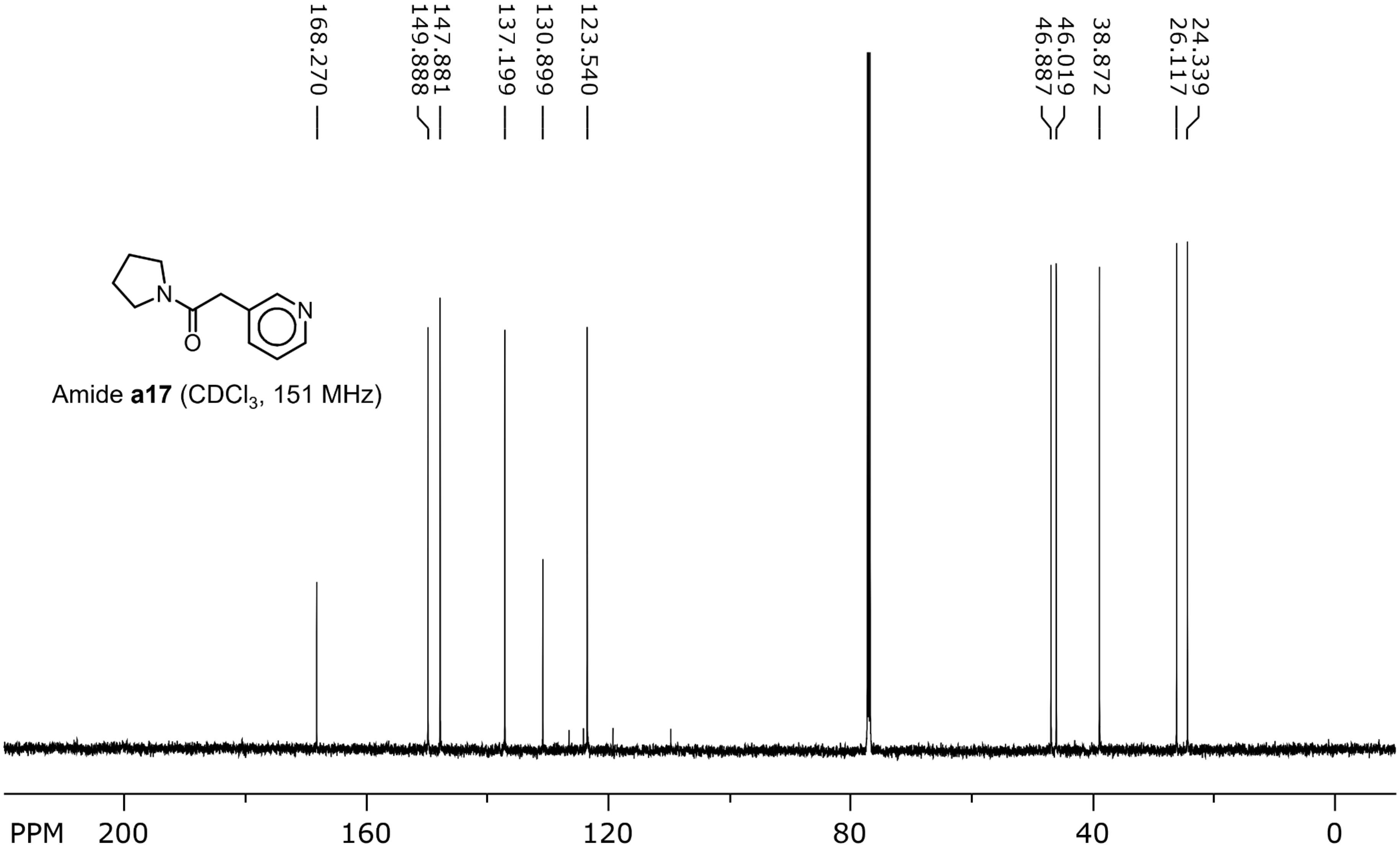

Amide **a17** ($CDCl_3$, 151 MHz)

^{1}H NMR for amide ***a18***

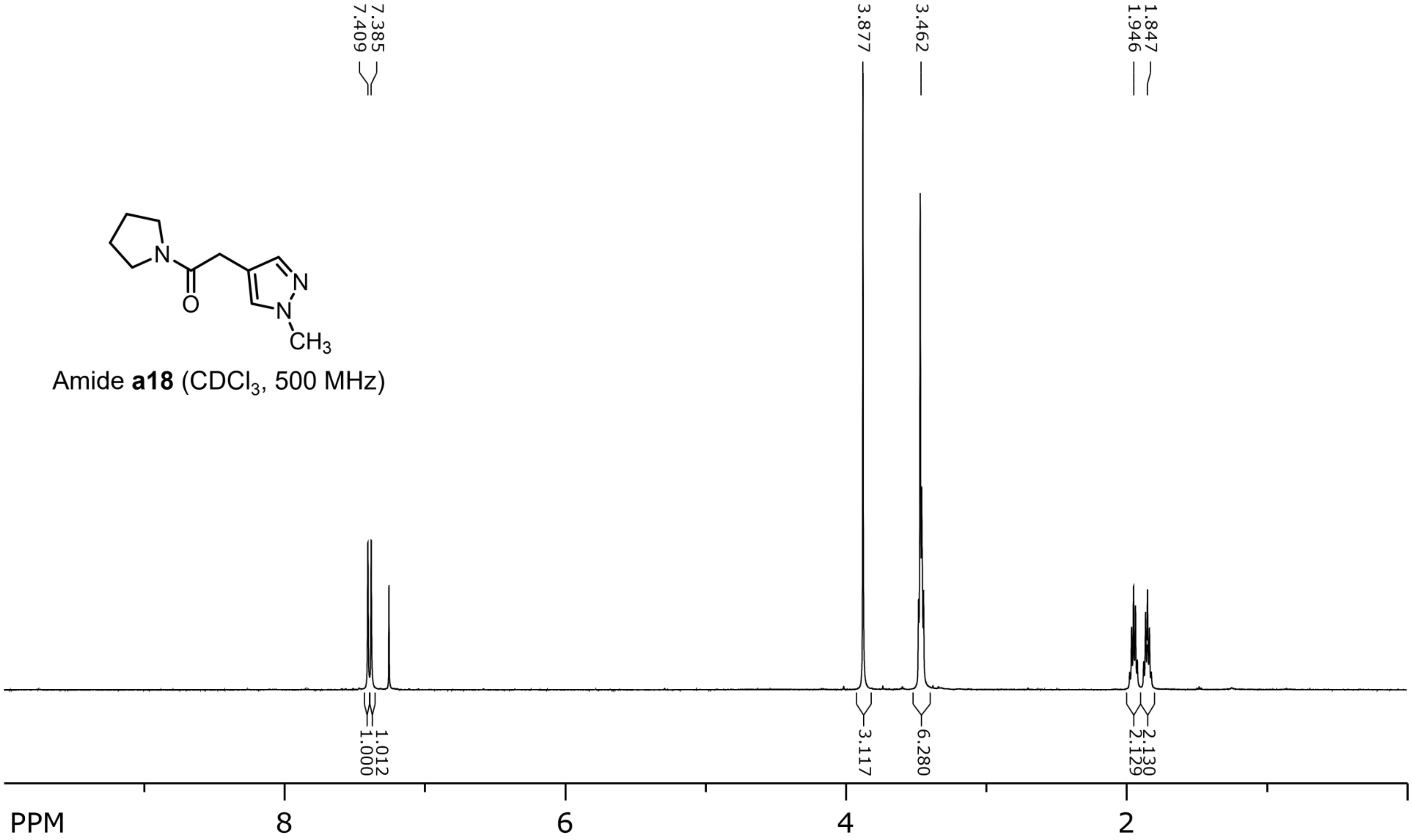

*$^{13}C\{^{1}H\}$ NMR for amide **a18***

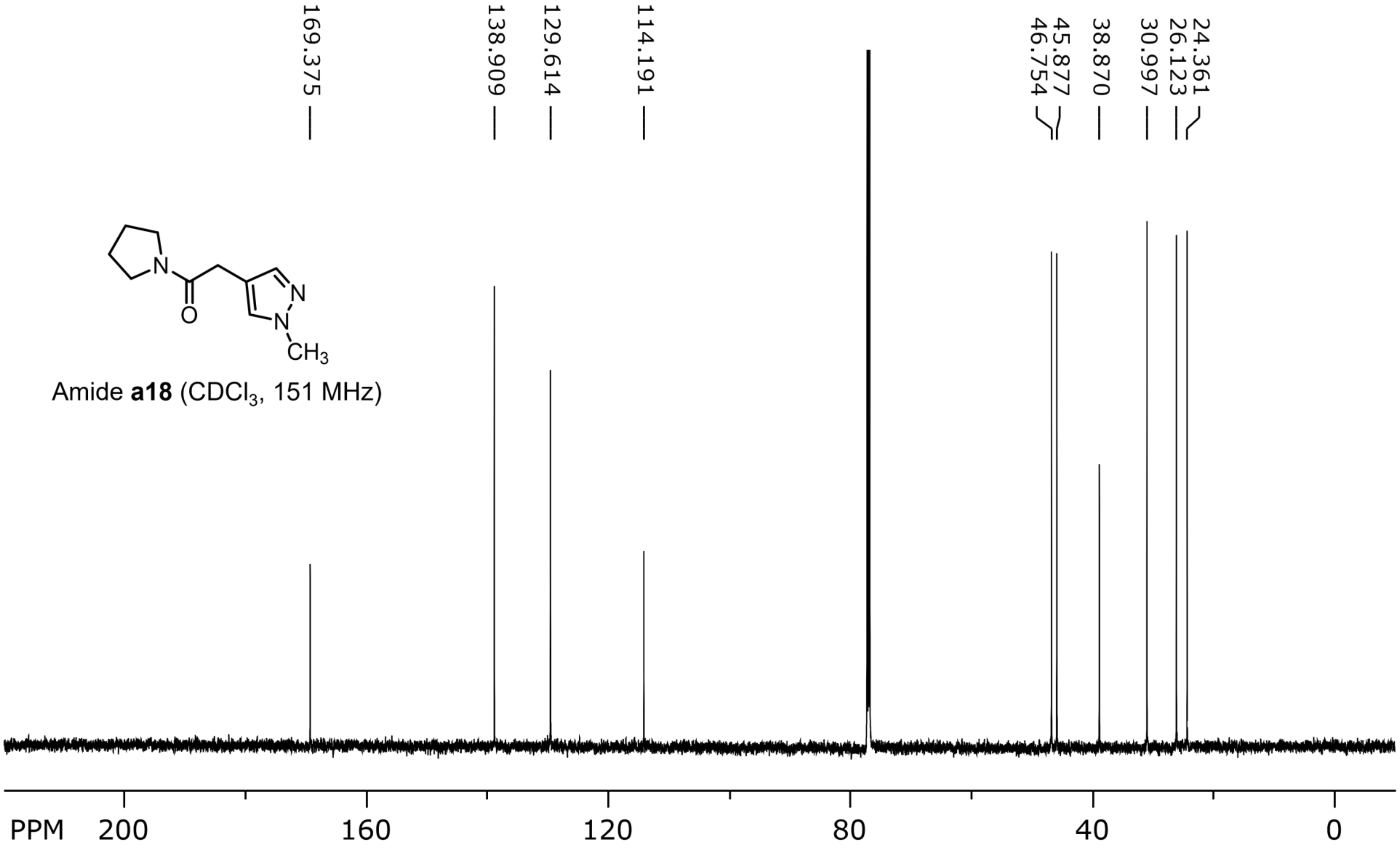

[2+2] cycloaddition products

*^{1}H NMR for compound **5***

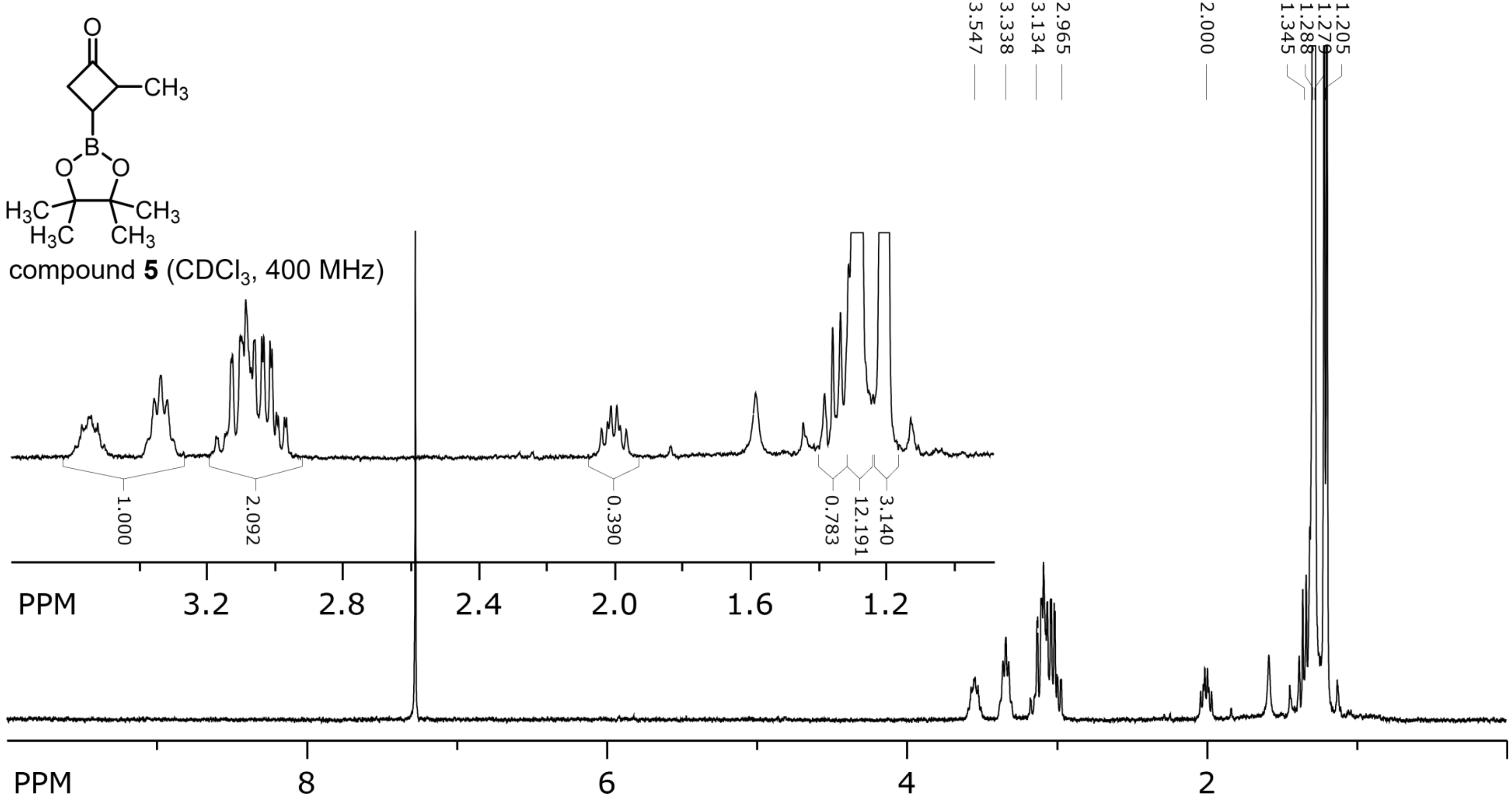

$^{13}C\{^{1}H\}$ *NMR for compound* ***5***

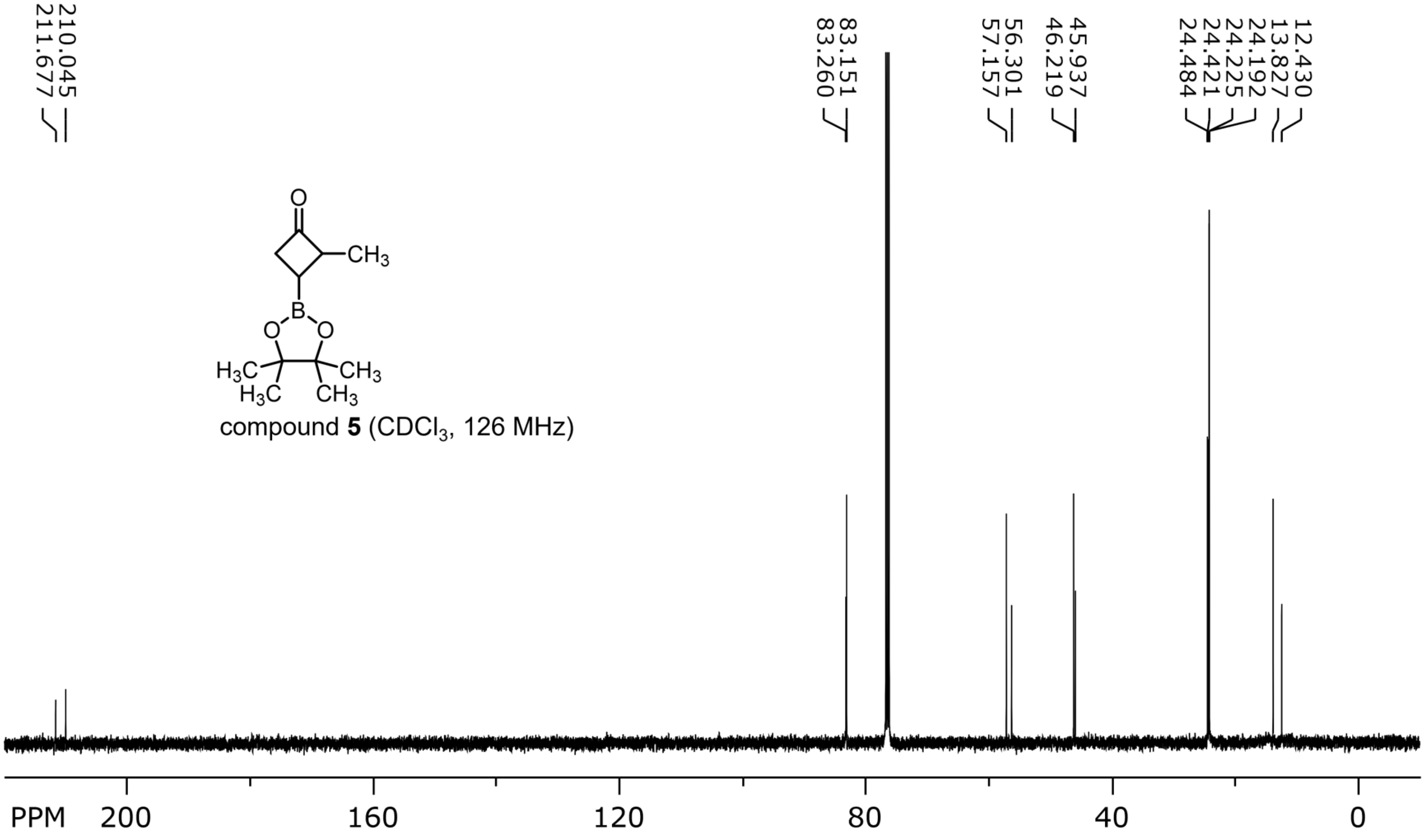

^{1}H NMR for compound ***6***

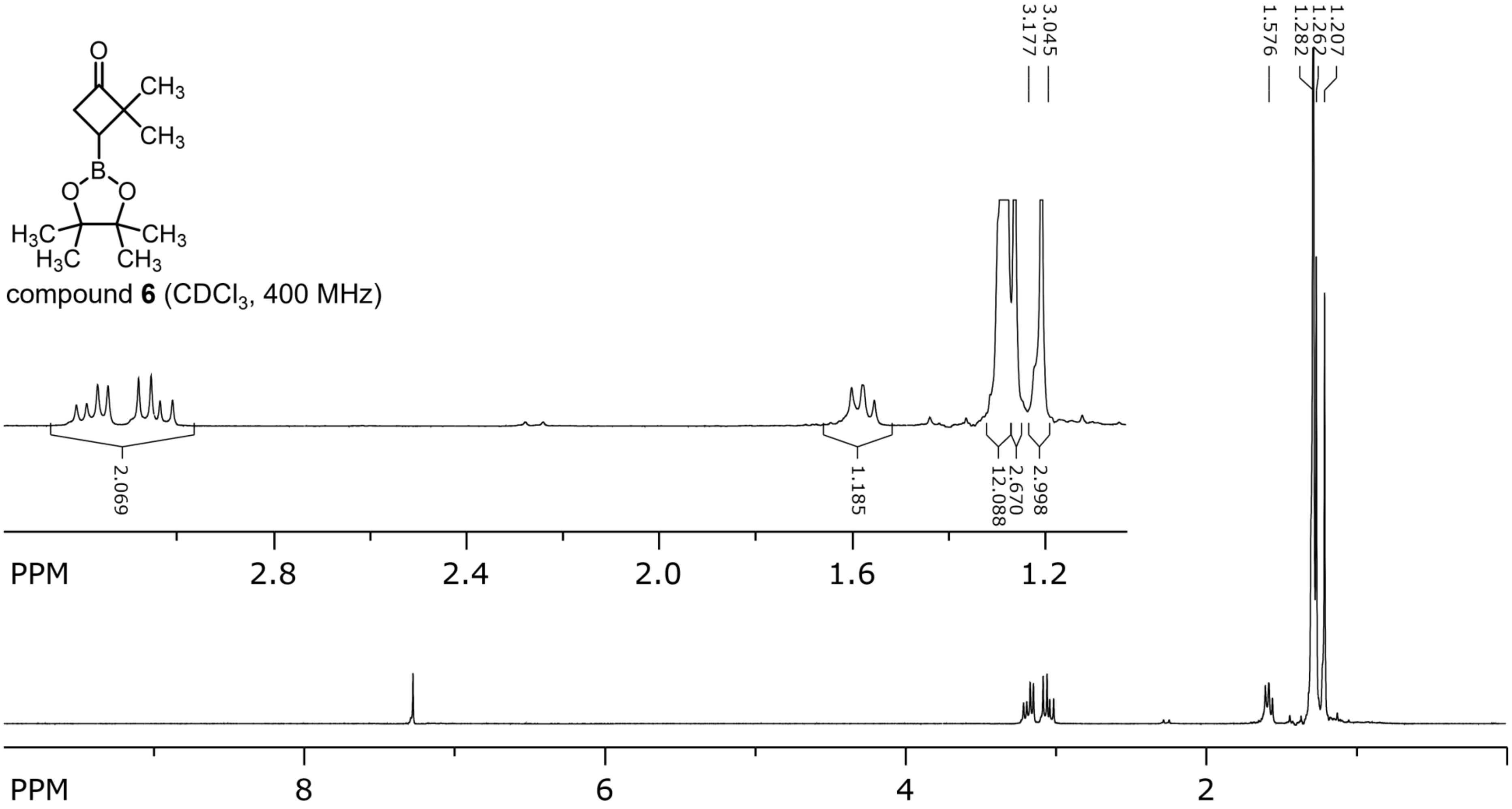

$^{13}C\{^{1}H\}$ *NMR for compound* ***6***

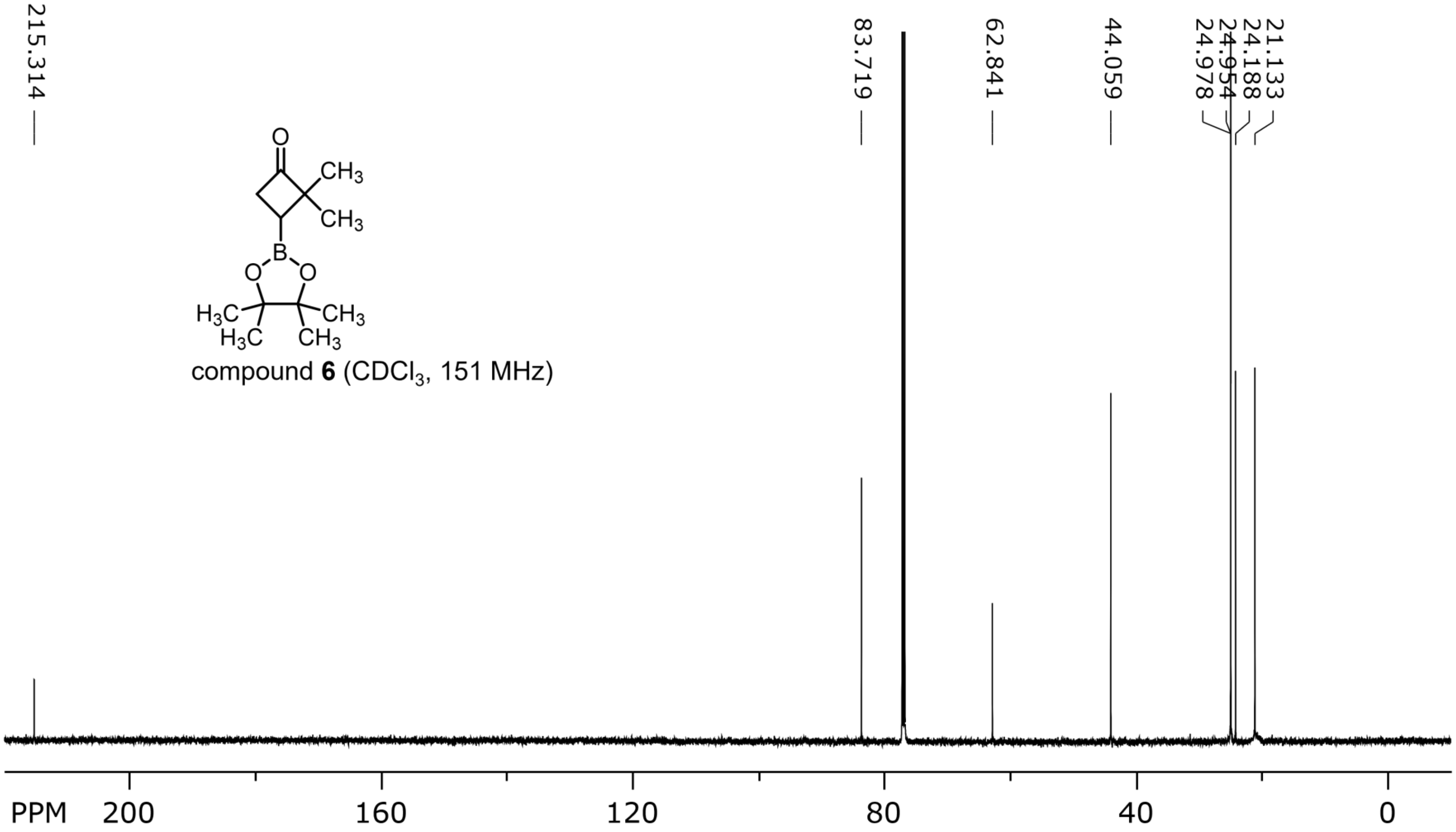

^{1}H NMR for compound **7**

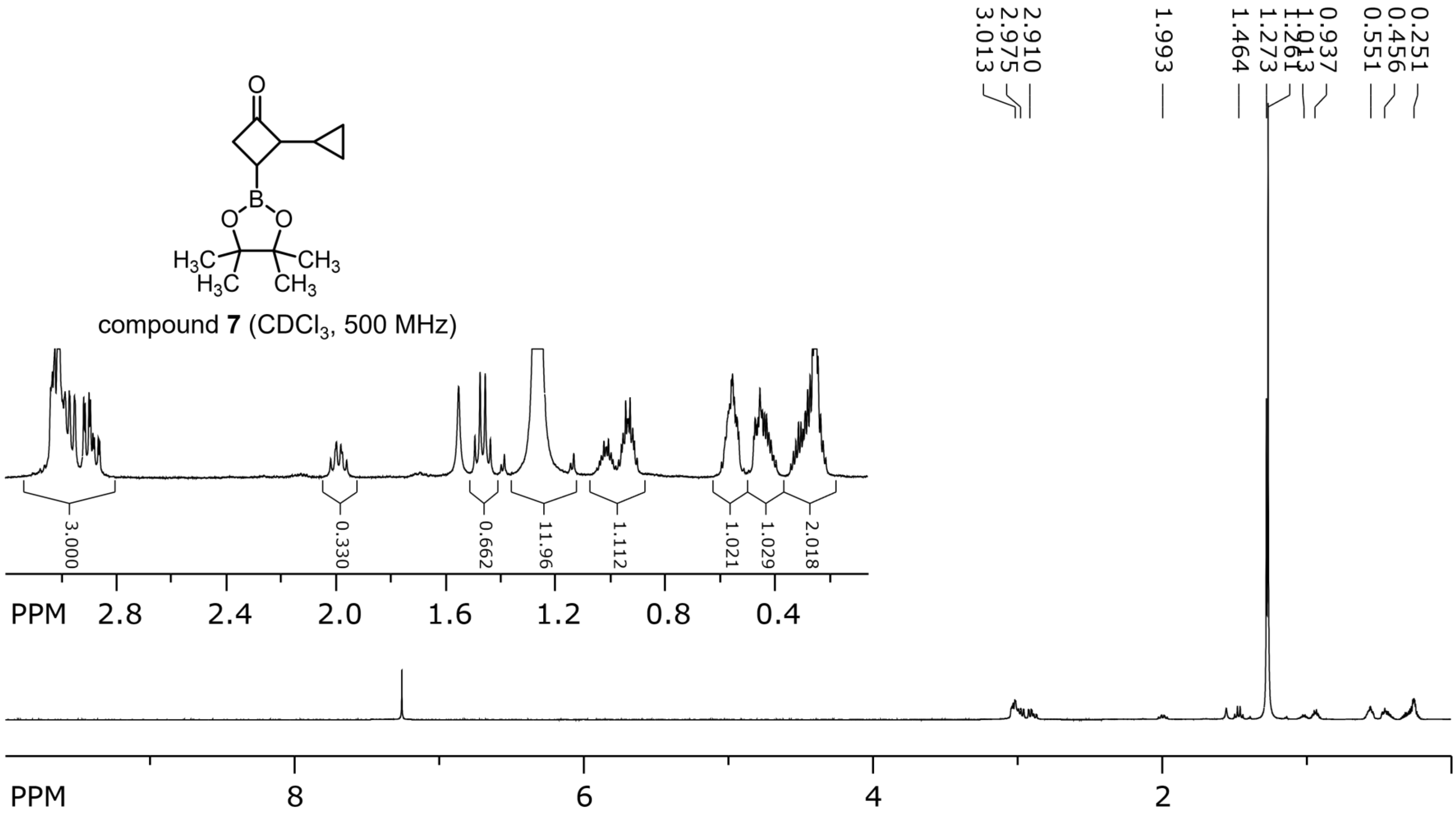

^{13}C{^{1}H} NMR for compound ***7***

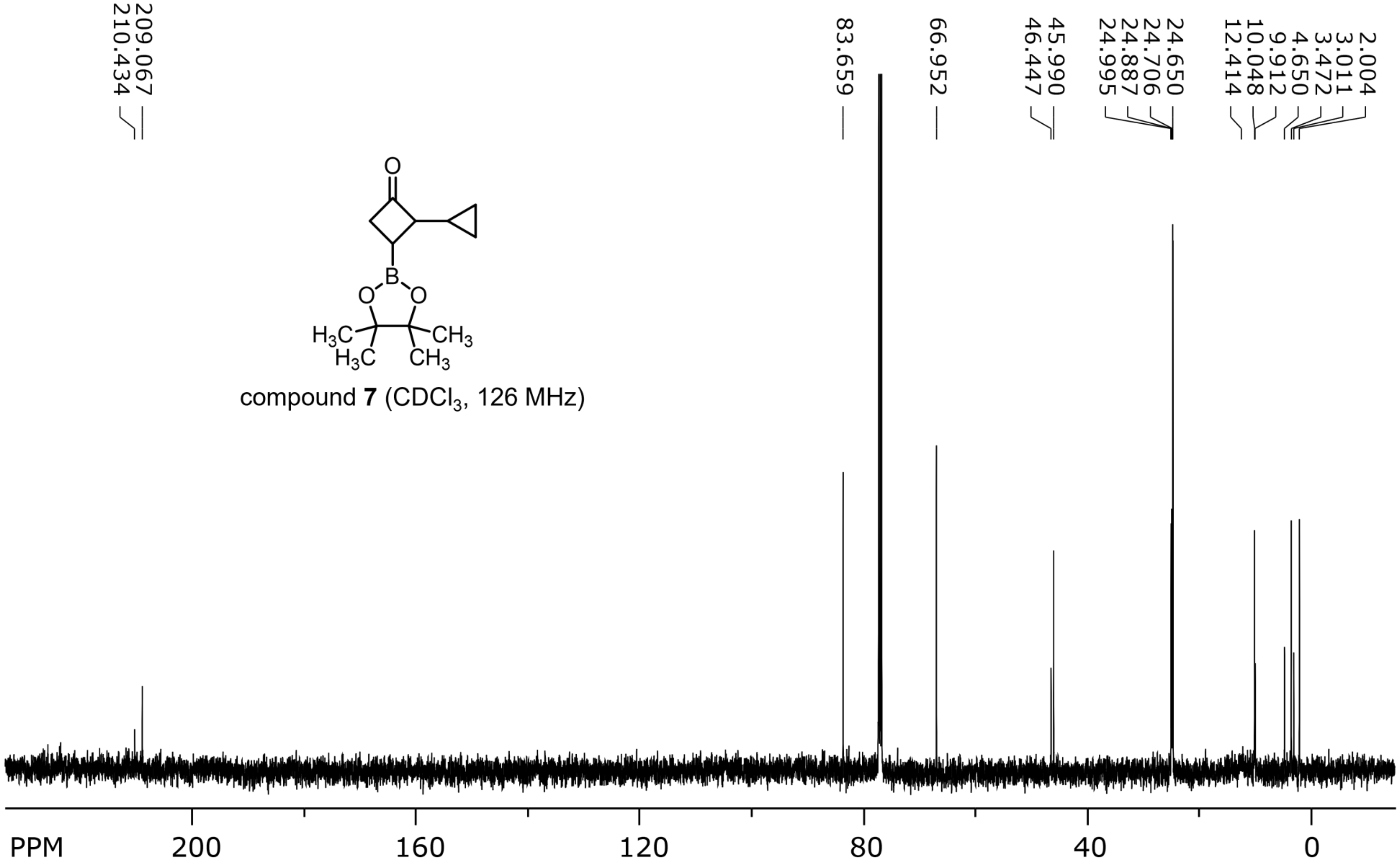

*^{1}H NMR for compound **8***

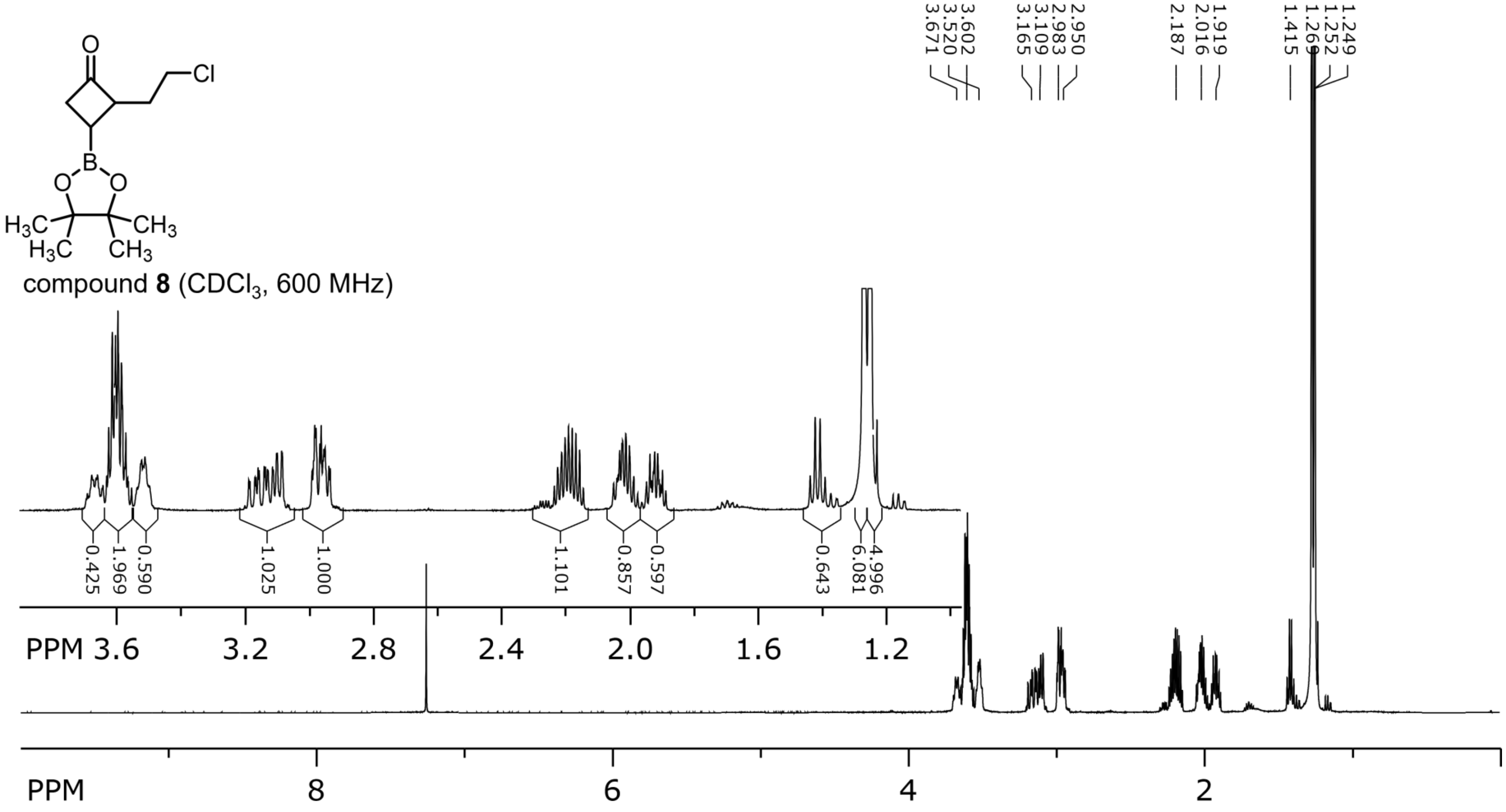

$^{13}C\{^{1}H\}$ *NMR for compound* ***8***

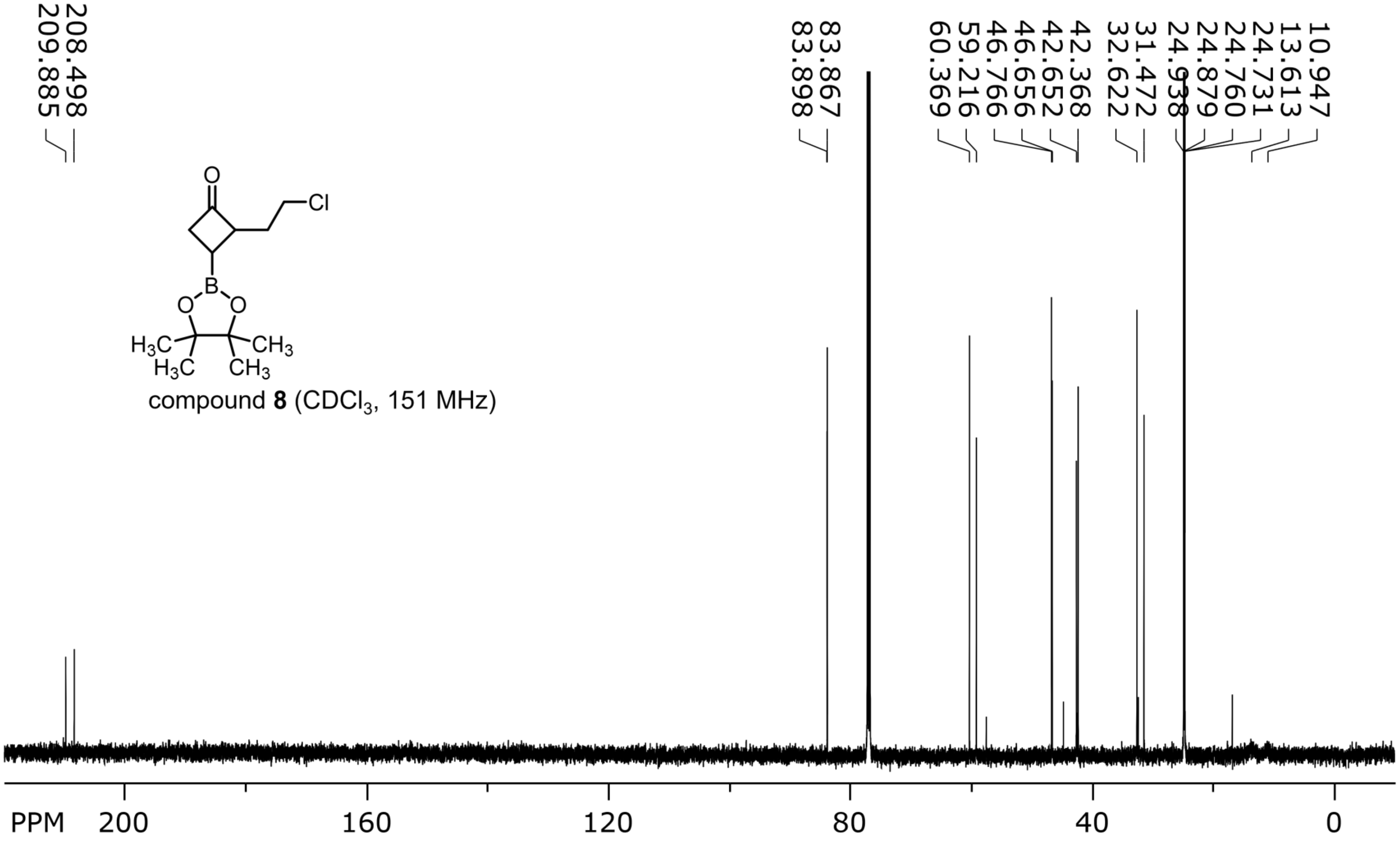

*$^1H\{^{13}C\}$ HSQC multiplicity edited for compound **8***

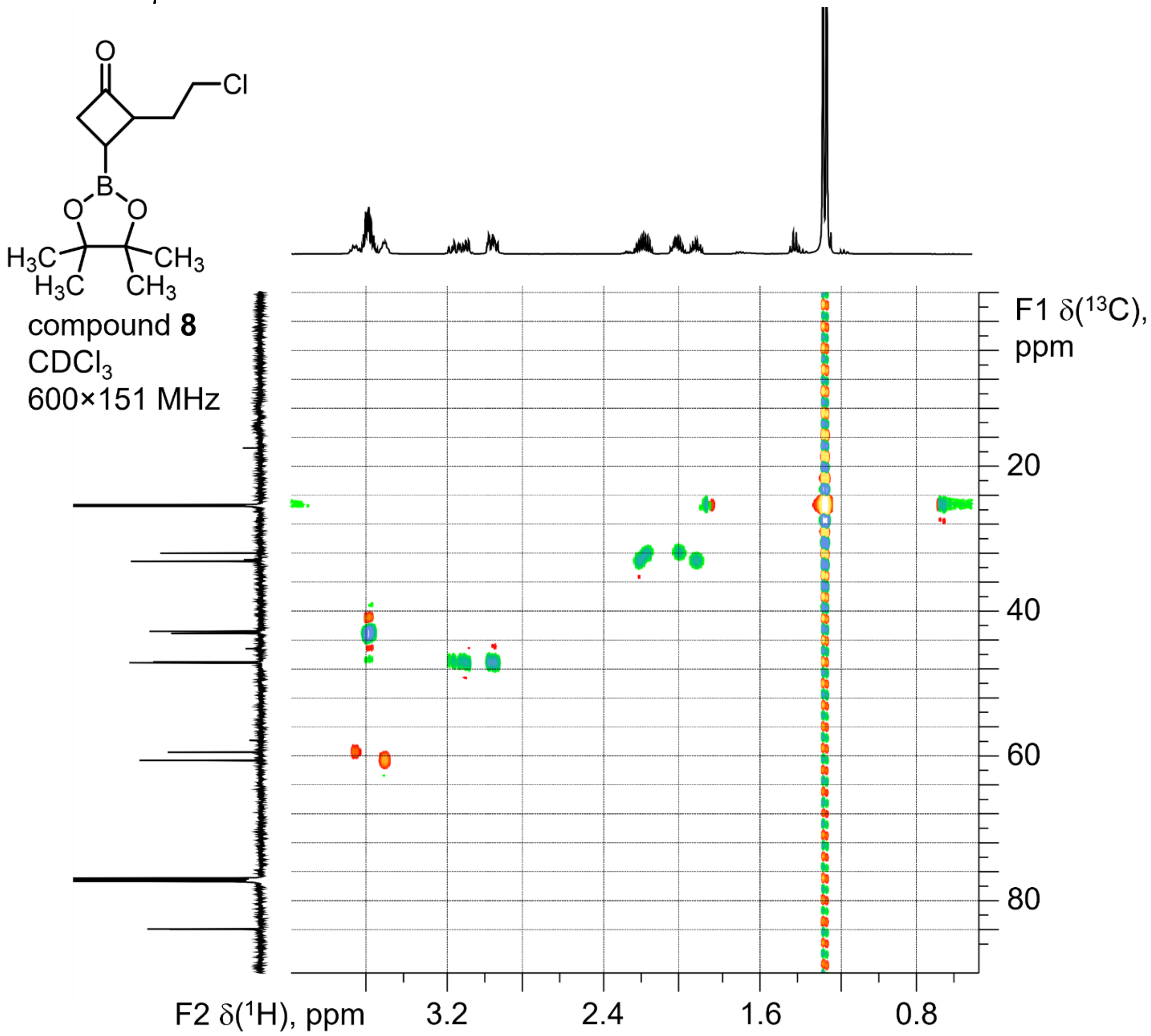

*^{1}H NMR for compound **9***

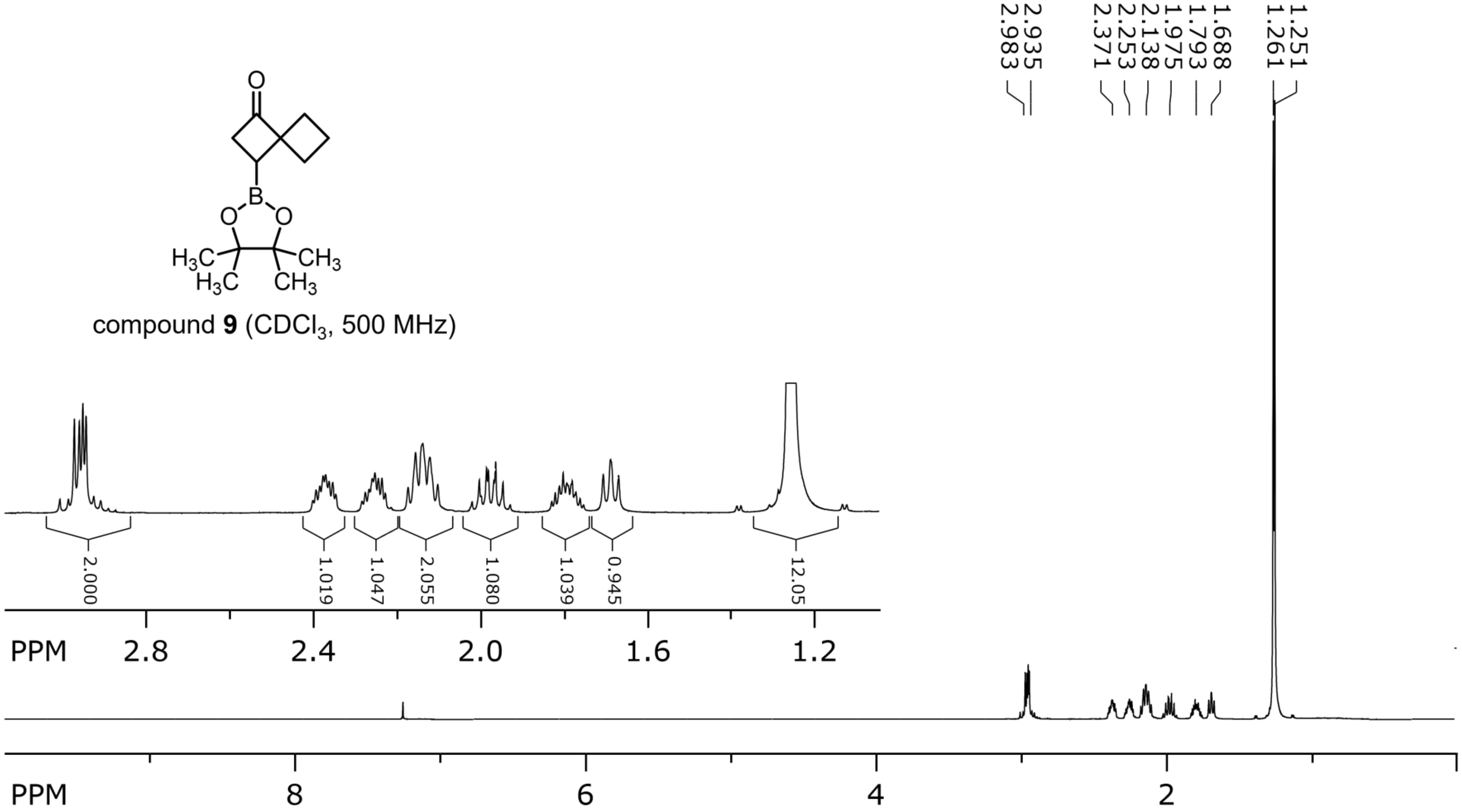

$^{13}C\{^{1}H\}$ *NMR for compound* ***9***

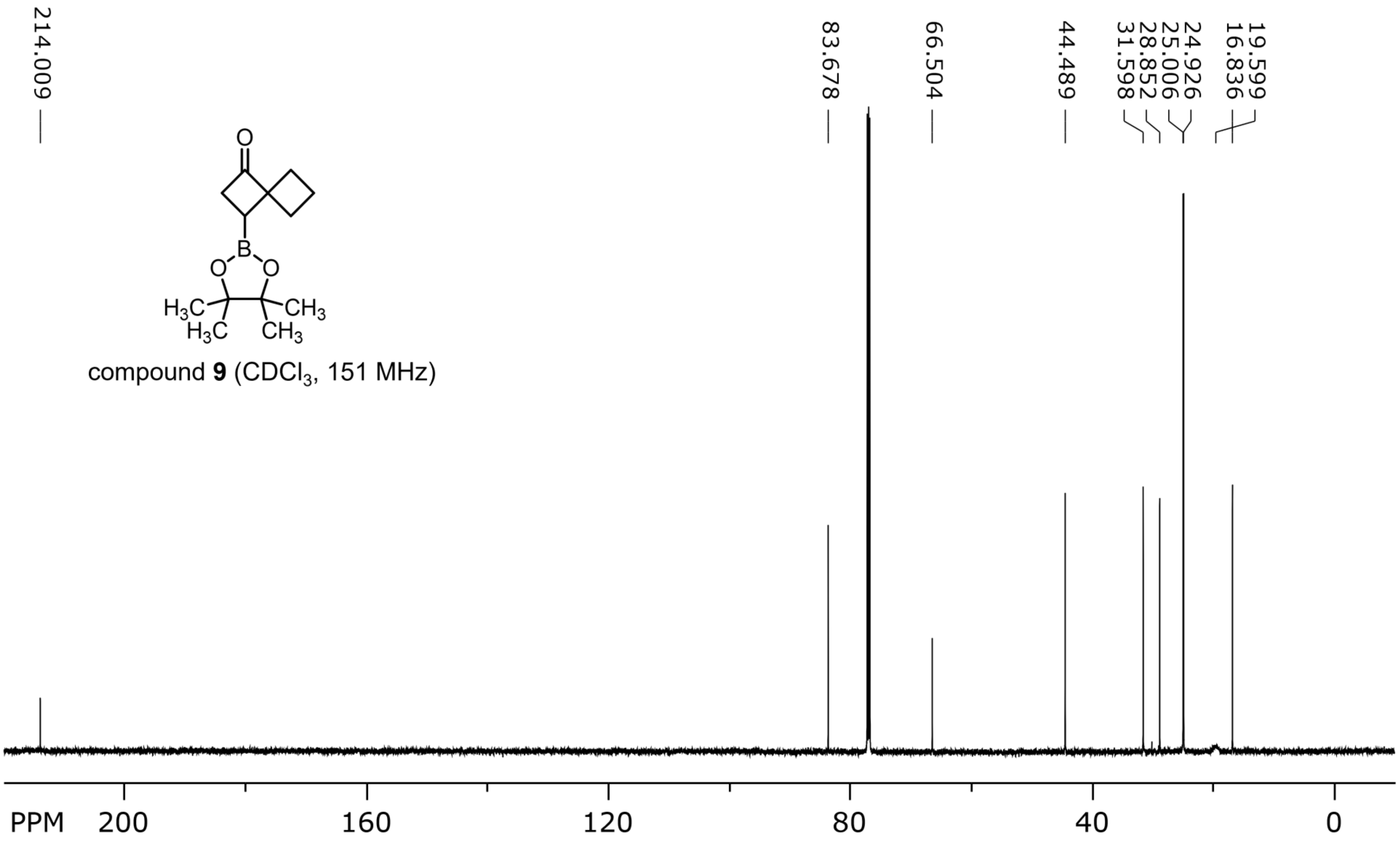

^{1}H NMR for compound ***10***

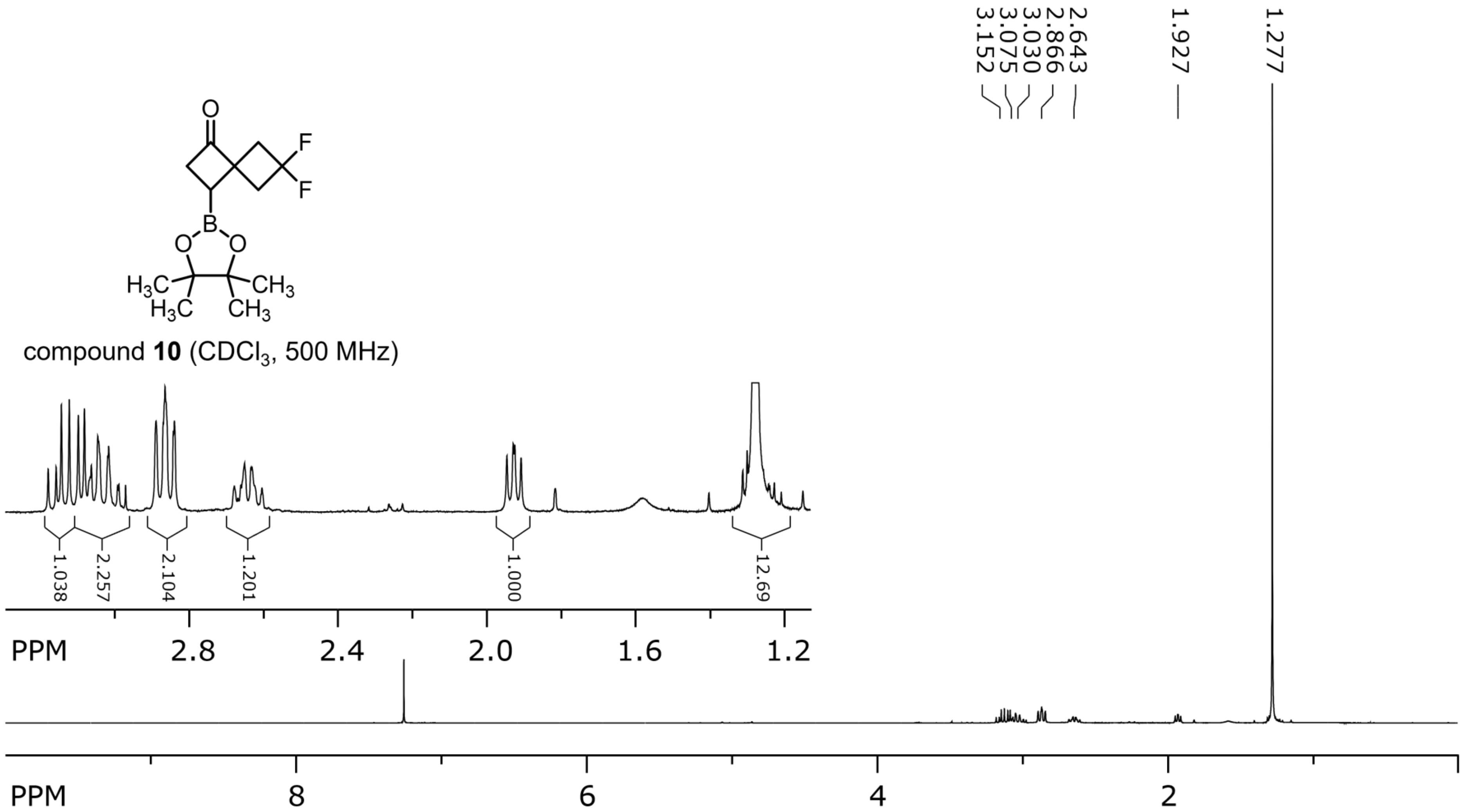

$^{13}C\{^{1}H\}$ *NMR for compound* ***10***

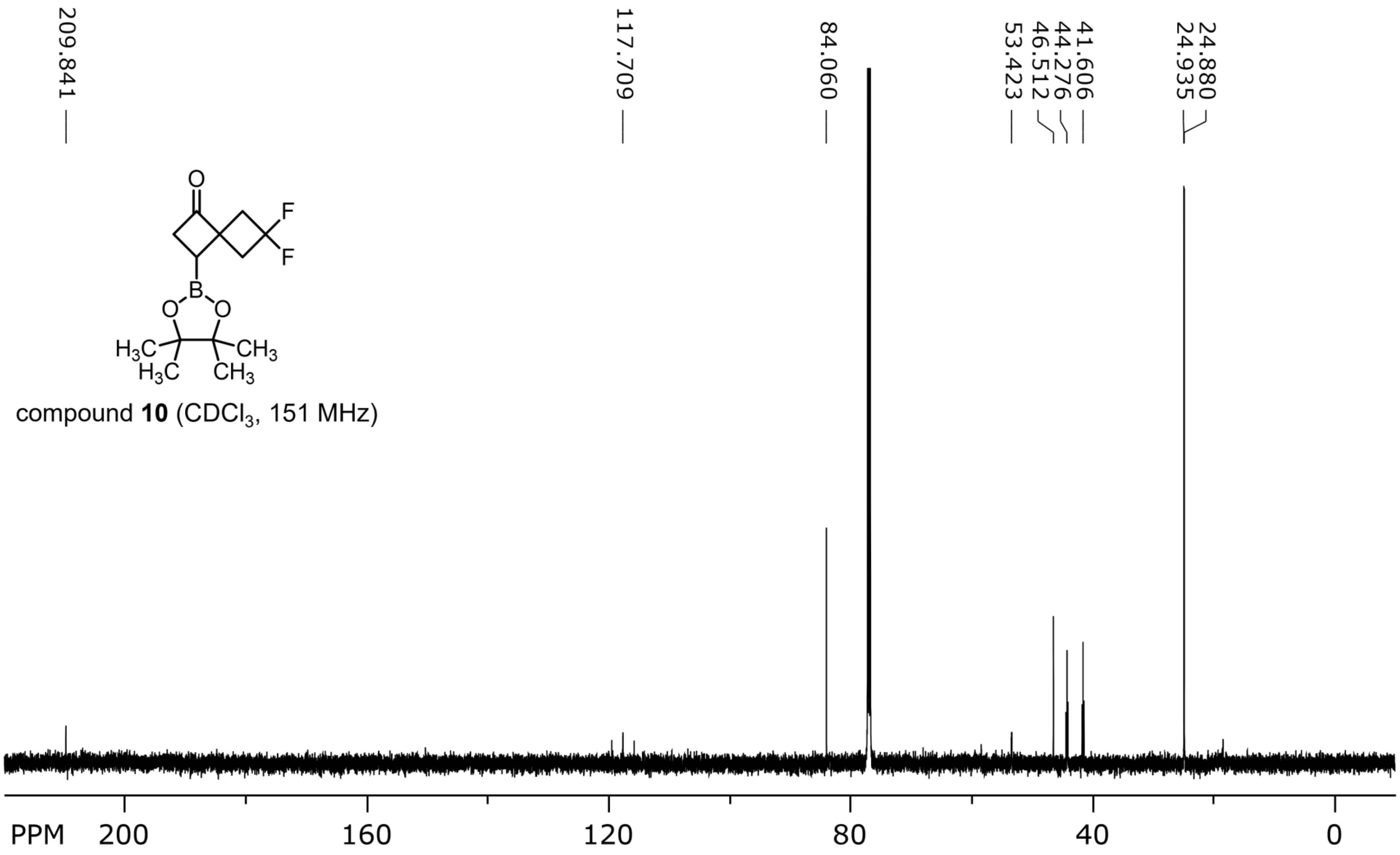

*^{19}F{^{1}H} NMR for compound **10***

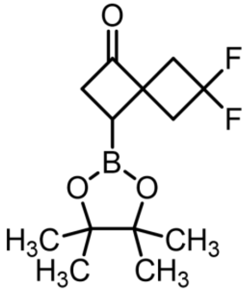

compound **10** ($CDCl_3$, 376 MHz)

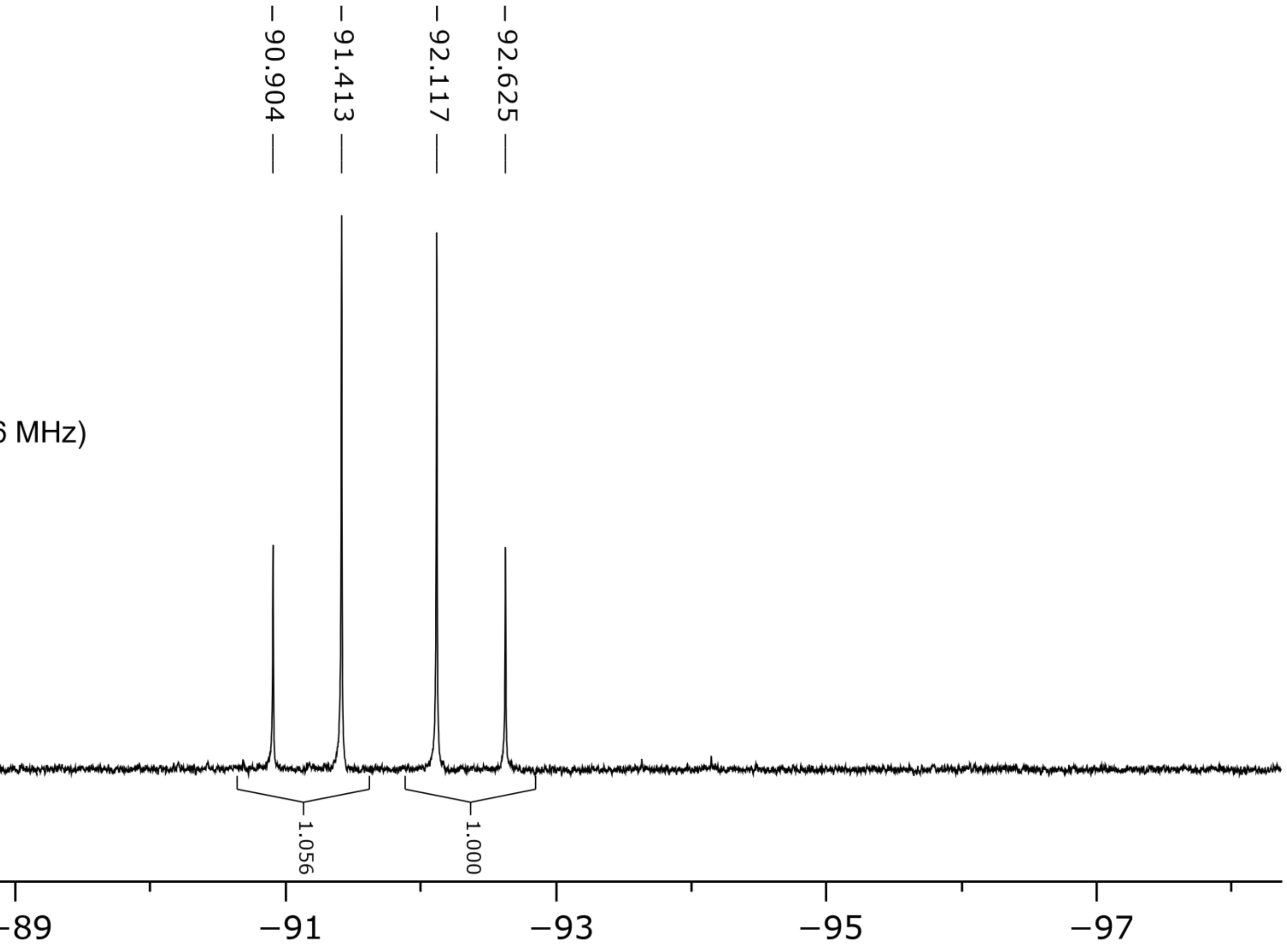

^{1}H NMR for compound **11**

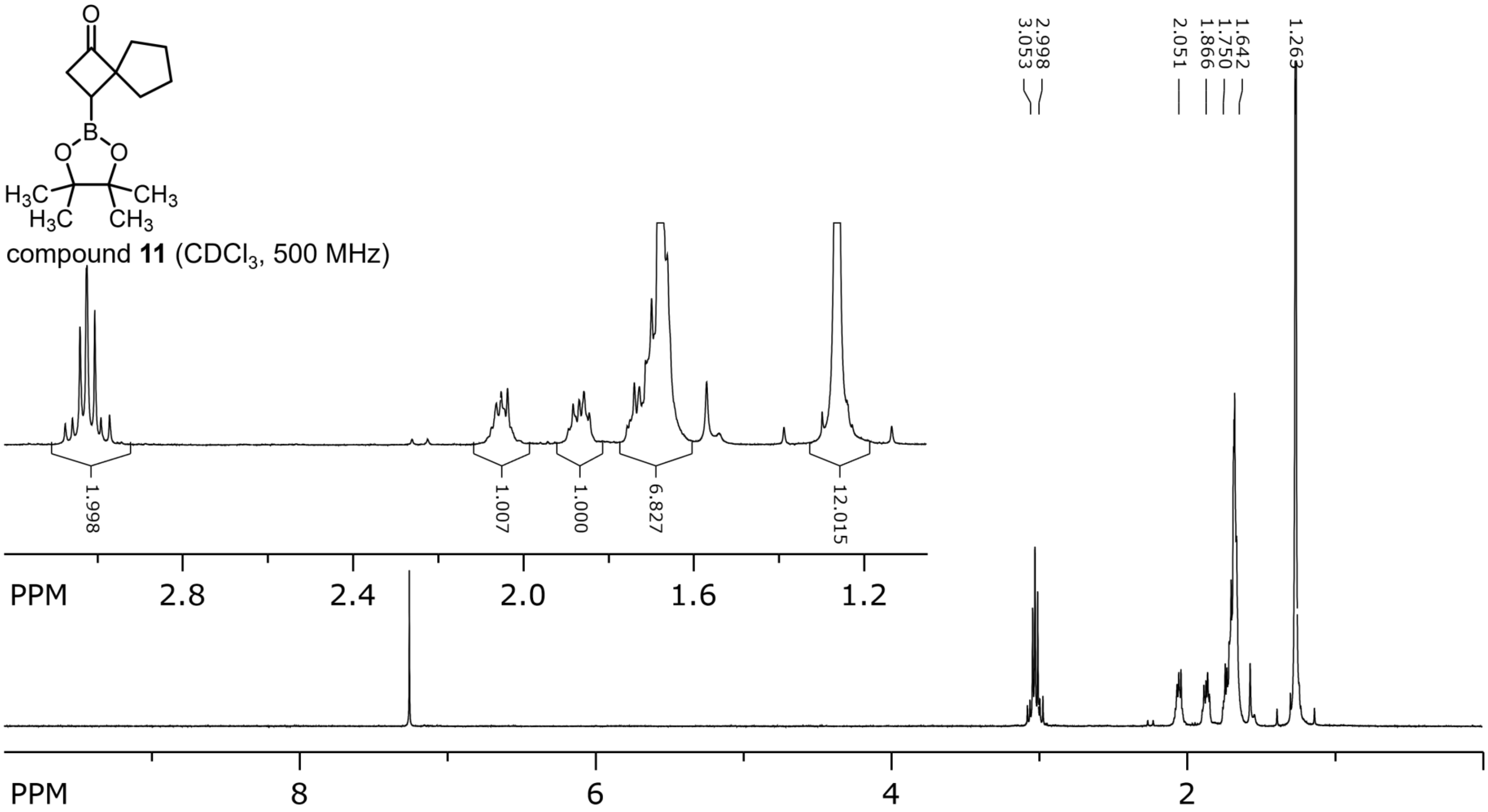

*$^{13}C\{^{1}H\}$ NMR for compound **11***

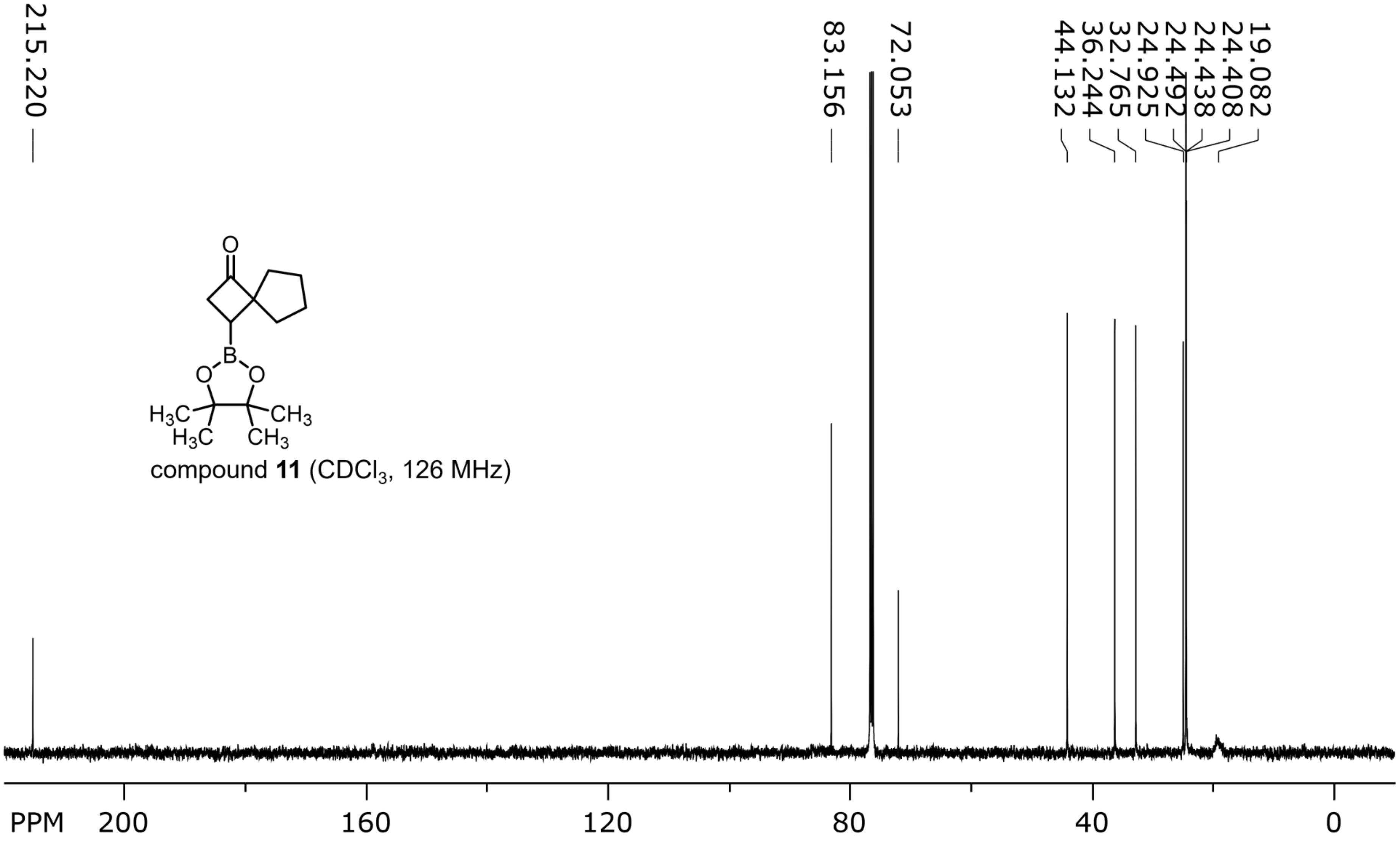

*^{1}H NMR for compound **12***

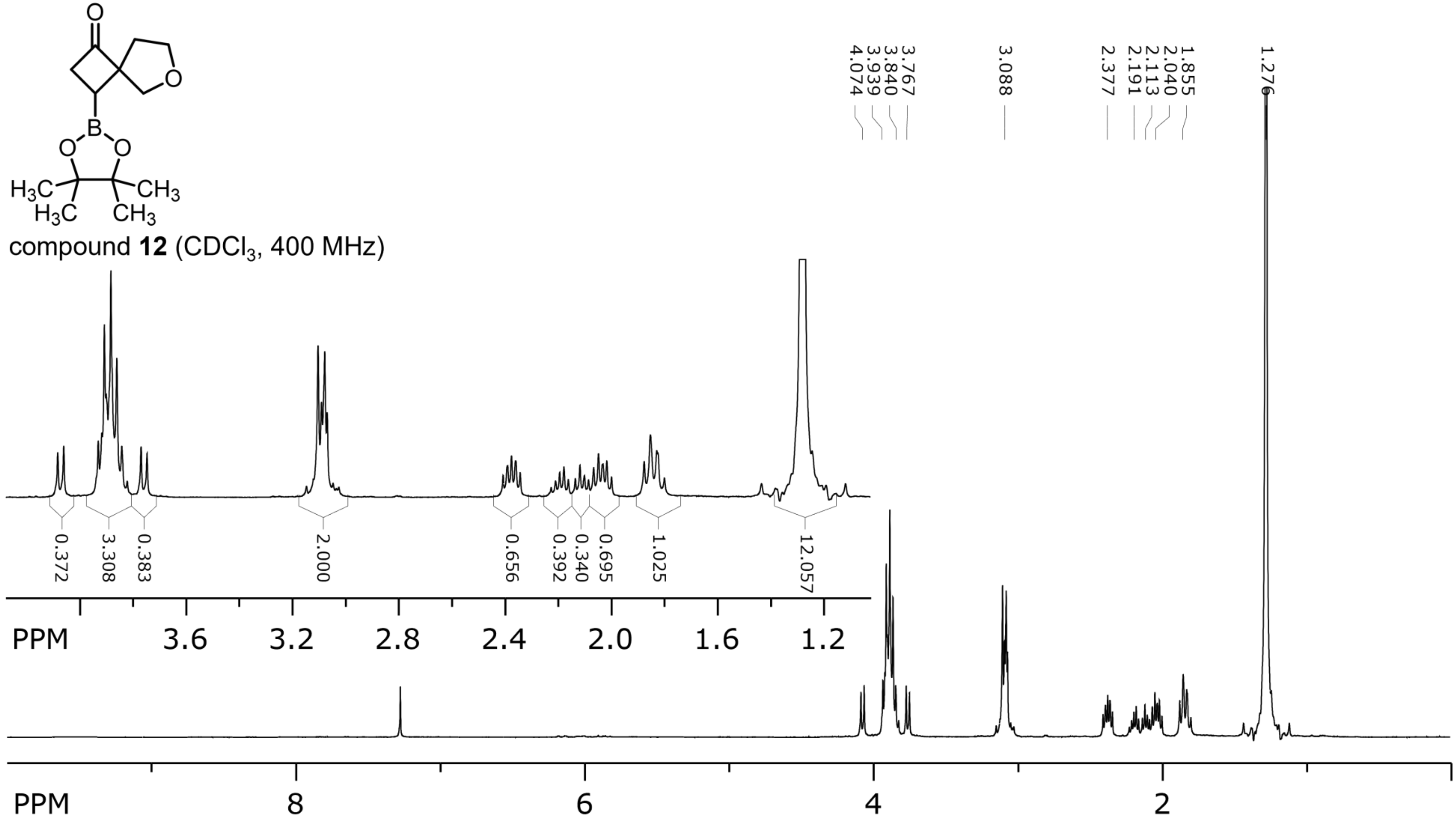

$^{13}C\{^{1}H\}$ *NMR for compound* ***12***

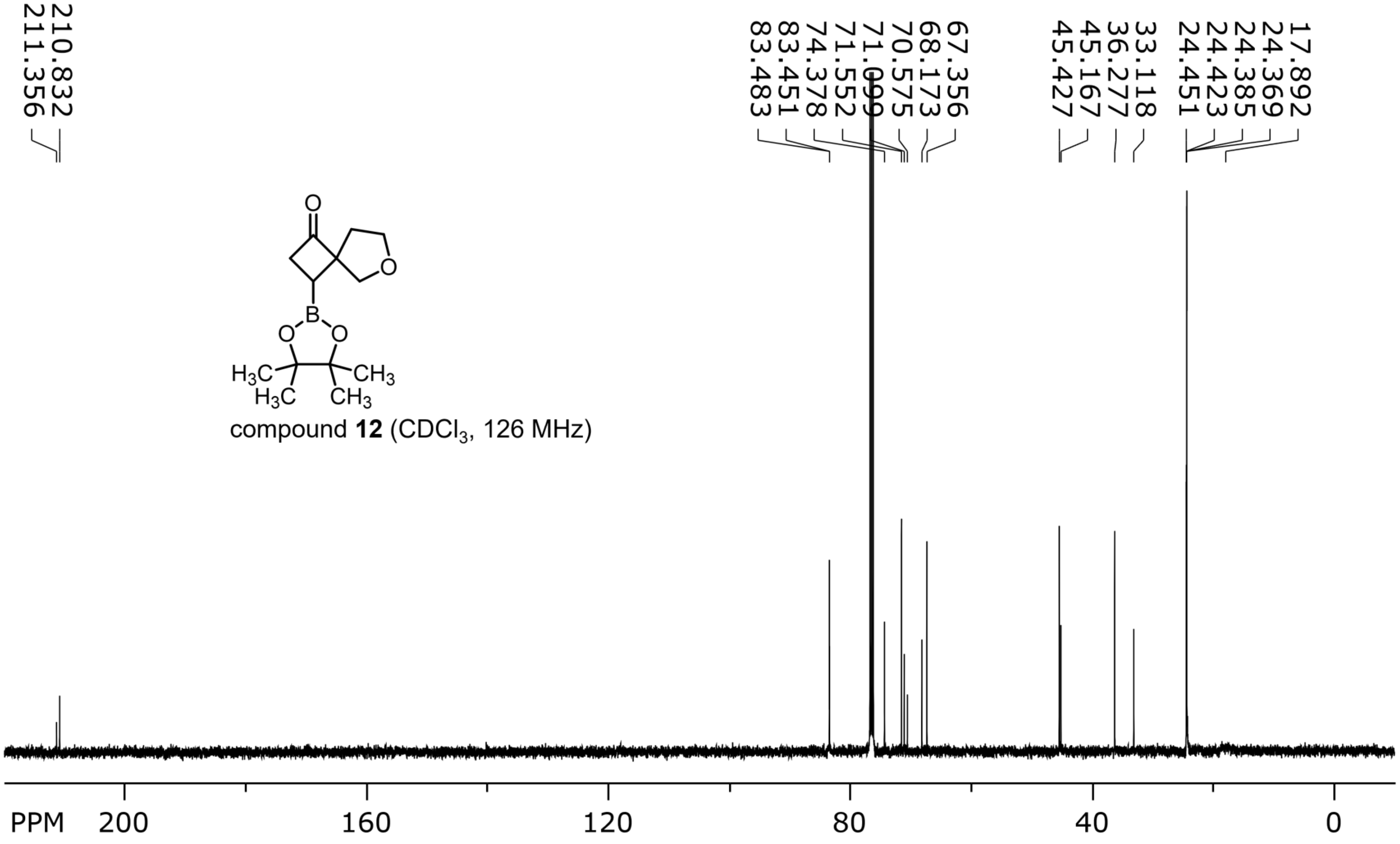

*^{1}H NMR for compound **13***

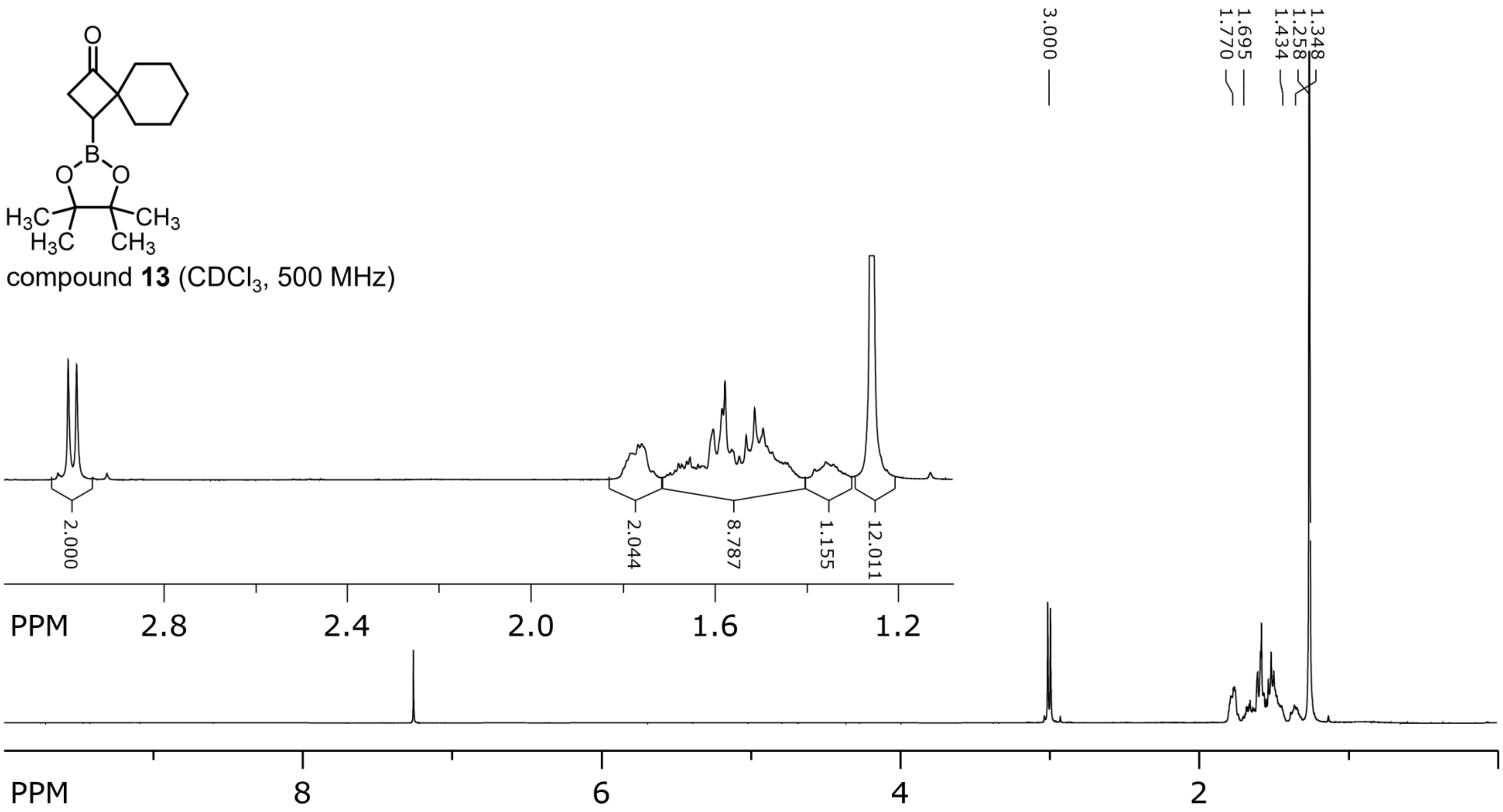

*$^{13}C\{^1H\}$ NMR for compound **13***

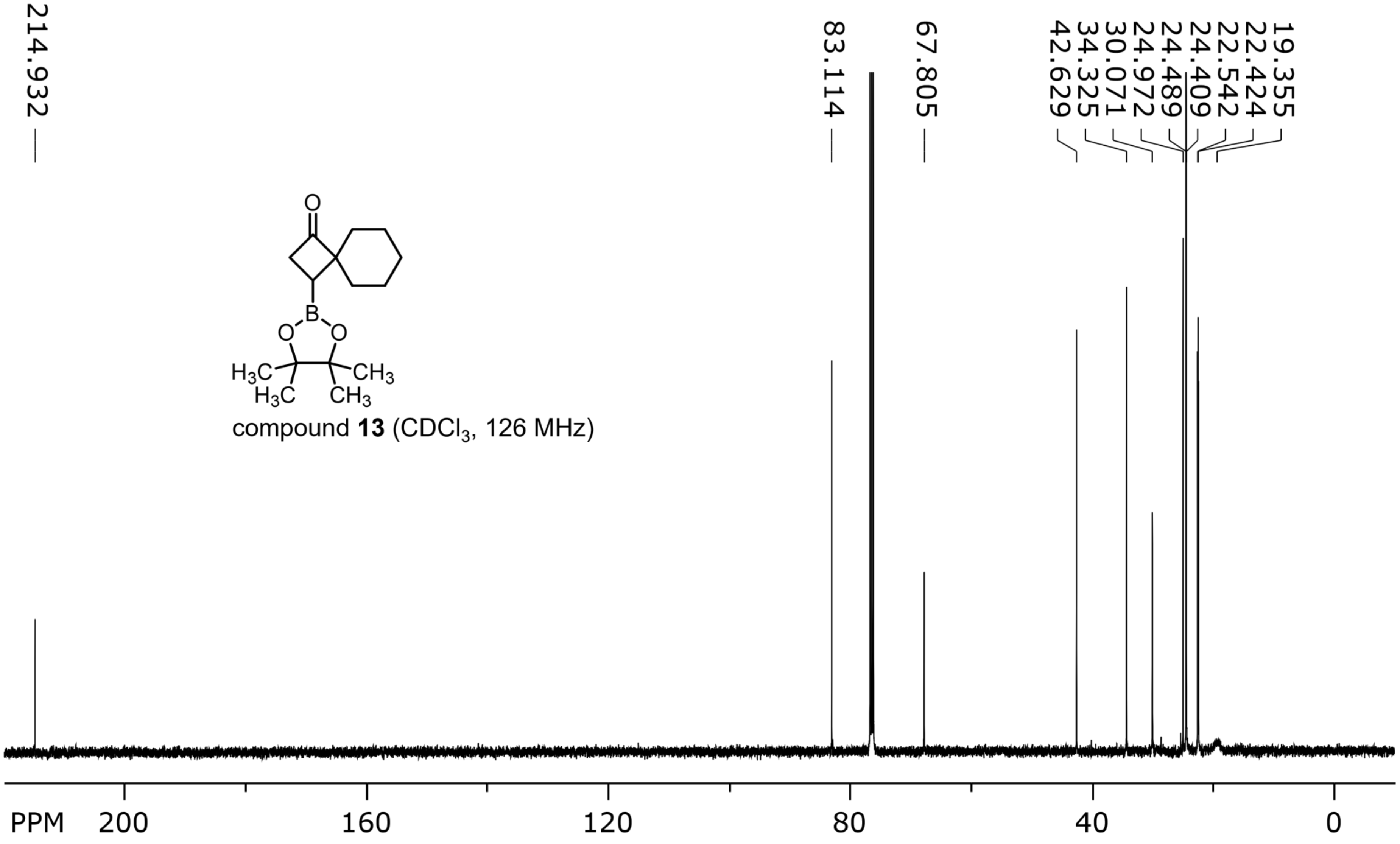

*^{1}H NMR for compound **14***

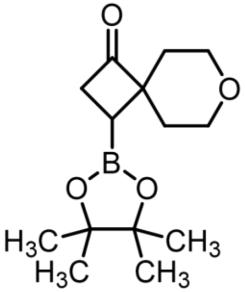

compound **14** ($CDCl_3$, 400 MHz)

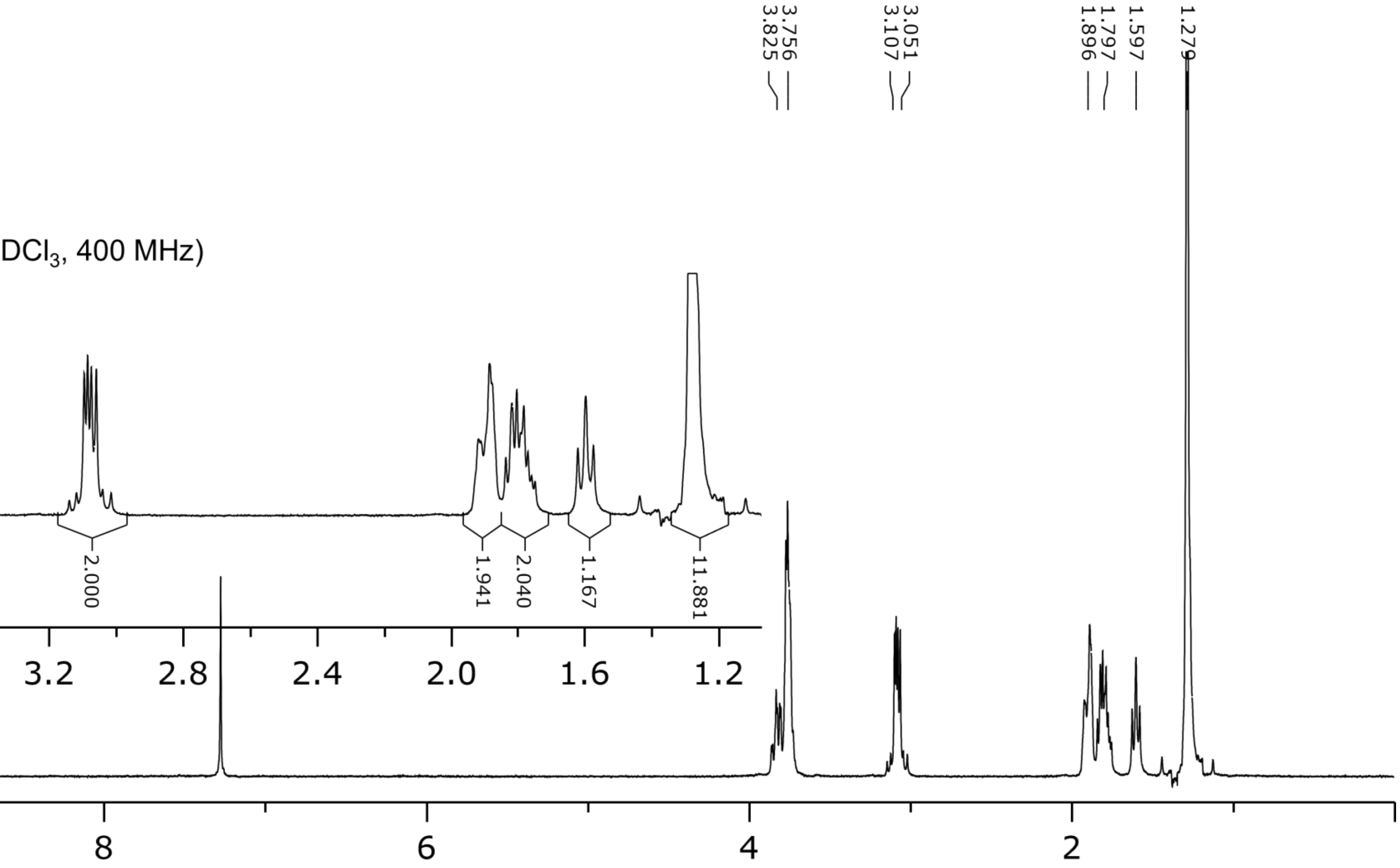

$^{13}C\{^{1}H\}$ NMR for compound ***14***

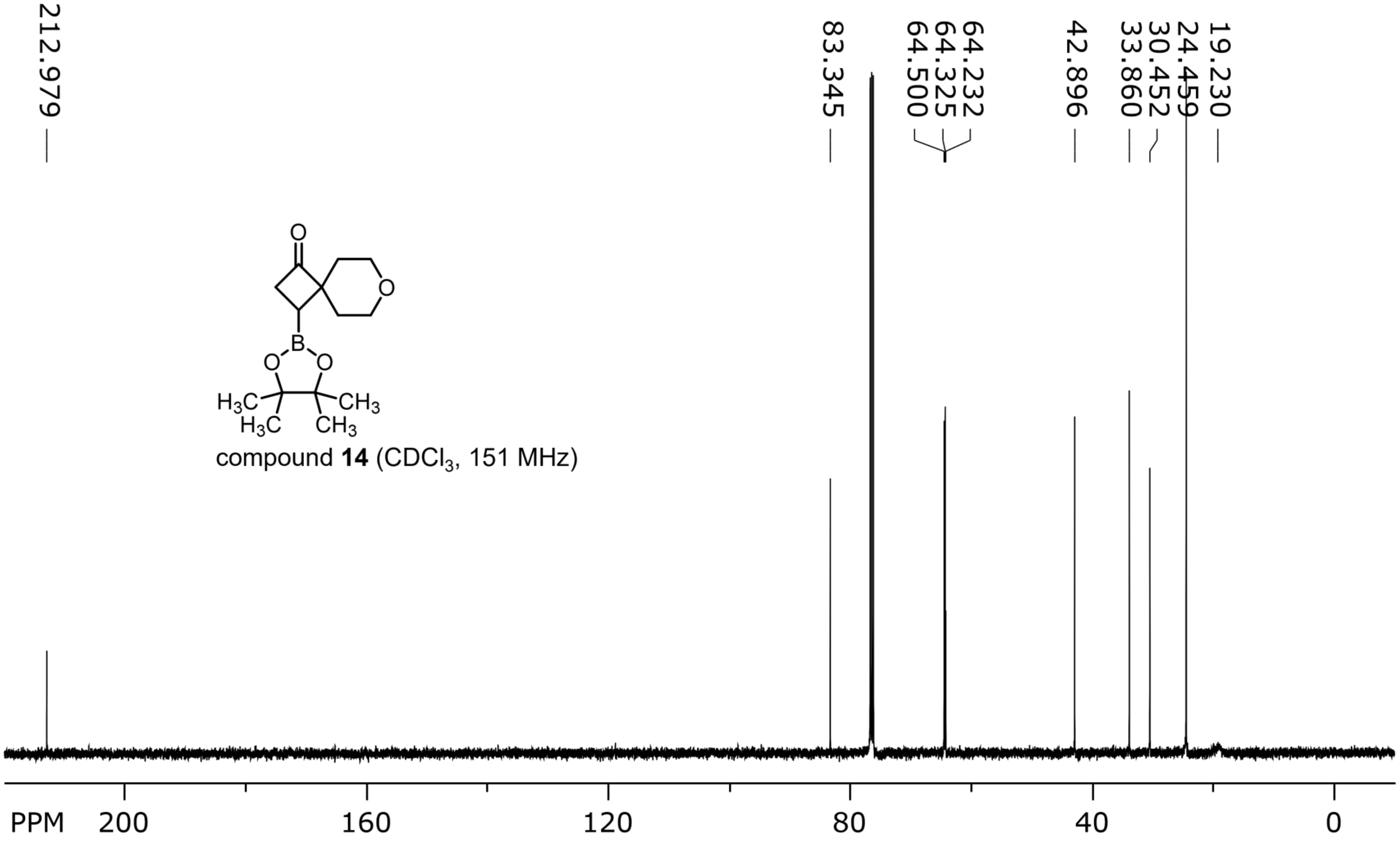

*^{1}H NMR for compound **15***

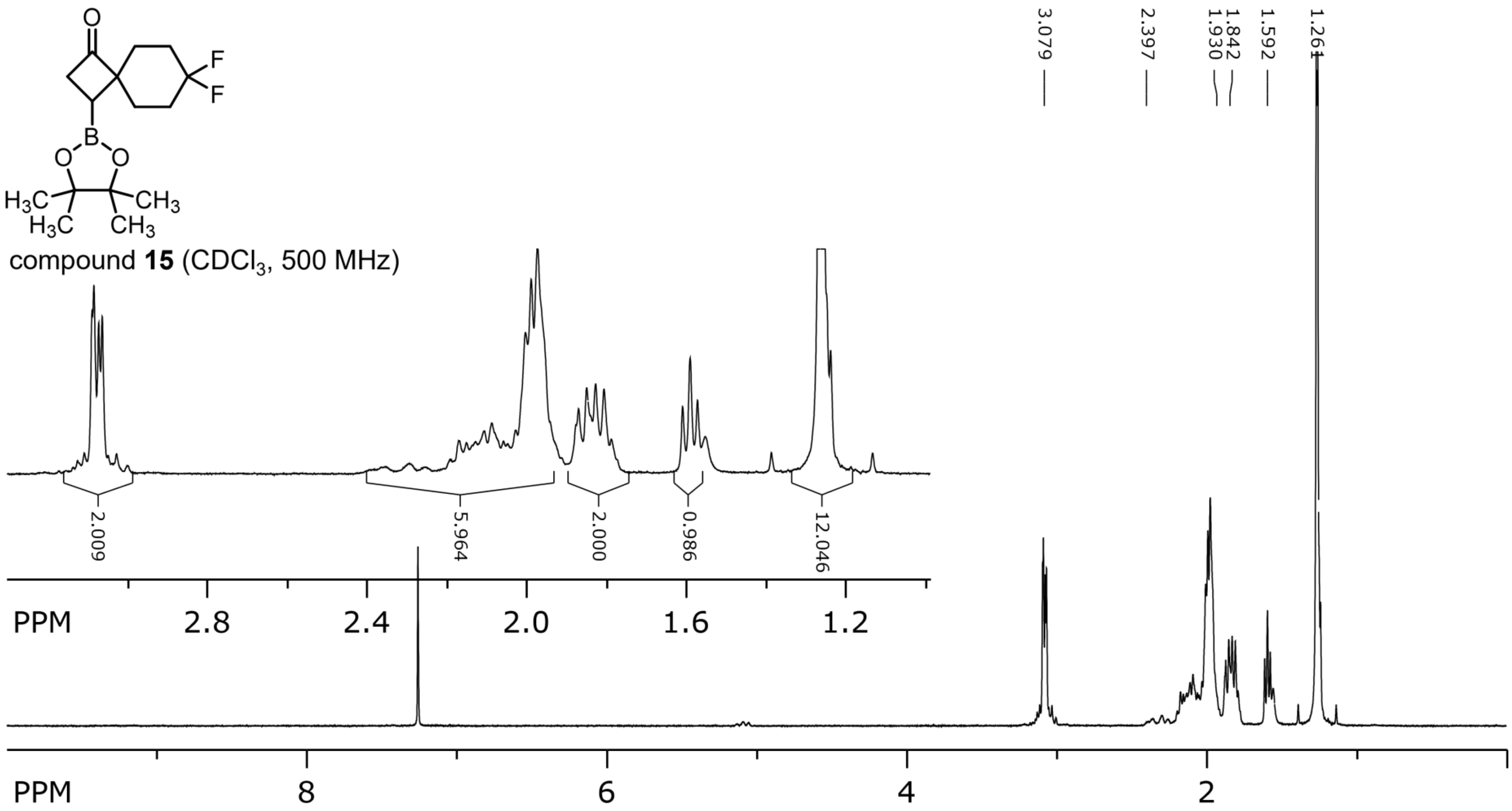

*^{13}C{^{1}H} NMR for compound **15***

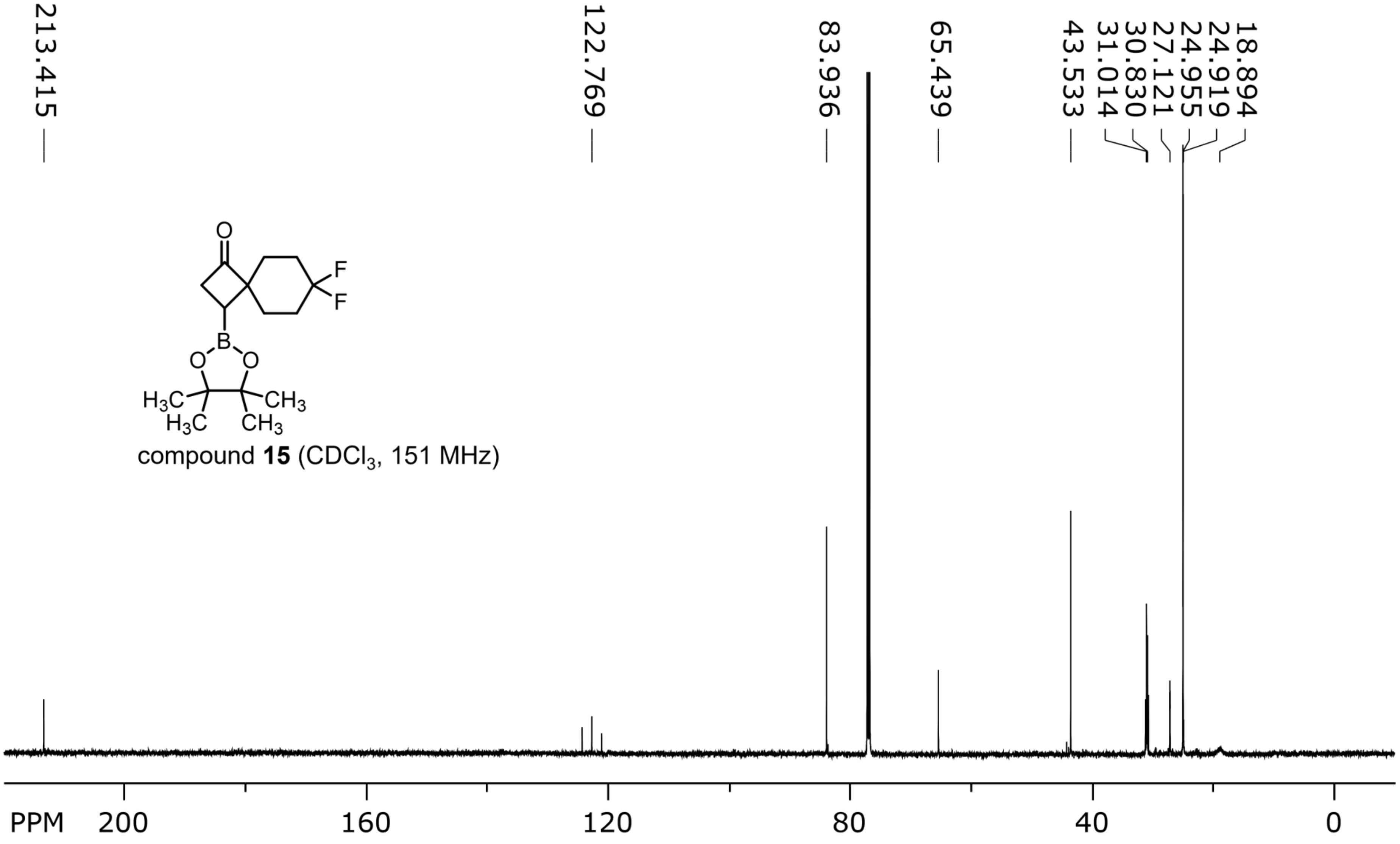

*^{19}F NMR for compound **15***

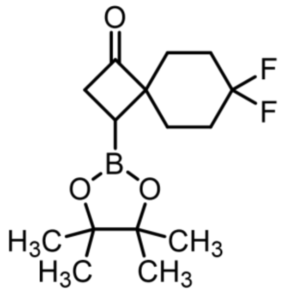

compound **15** ($CDCl_3$, 376 MHz)

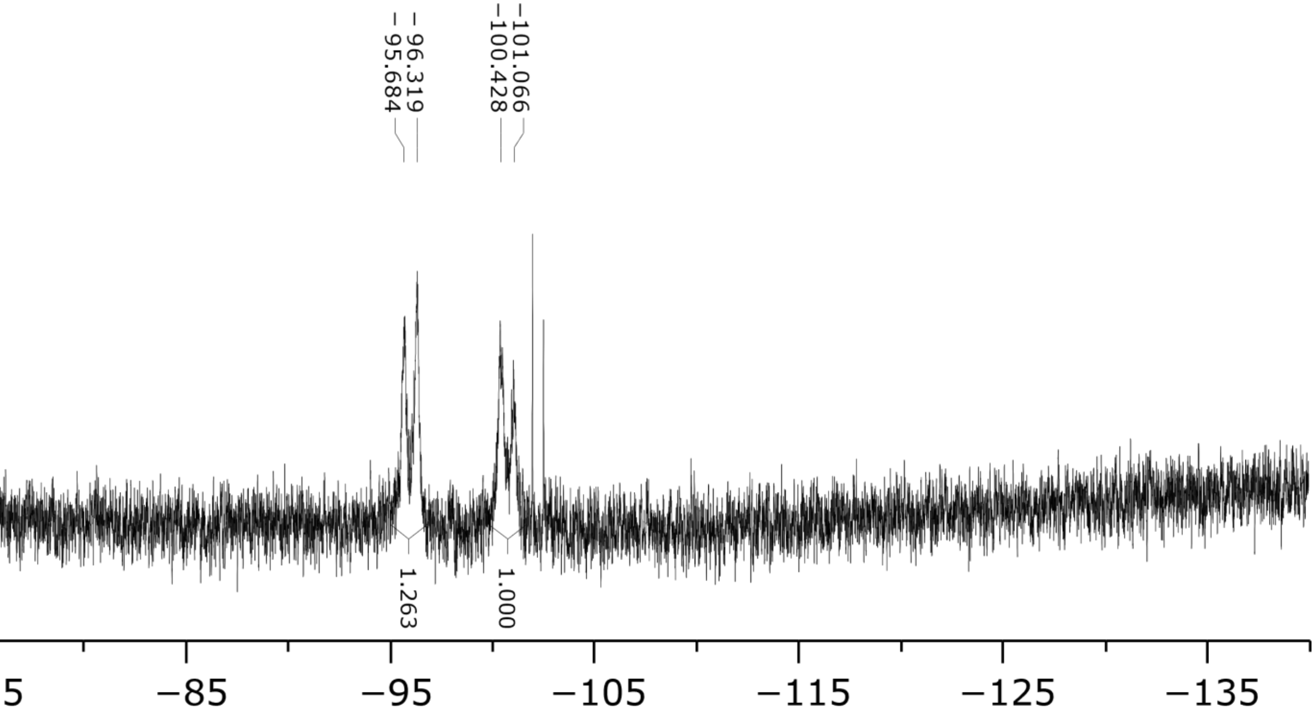

*^{1}H NMR for compound **16***

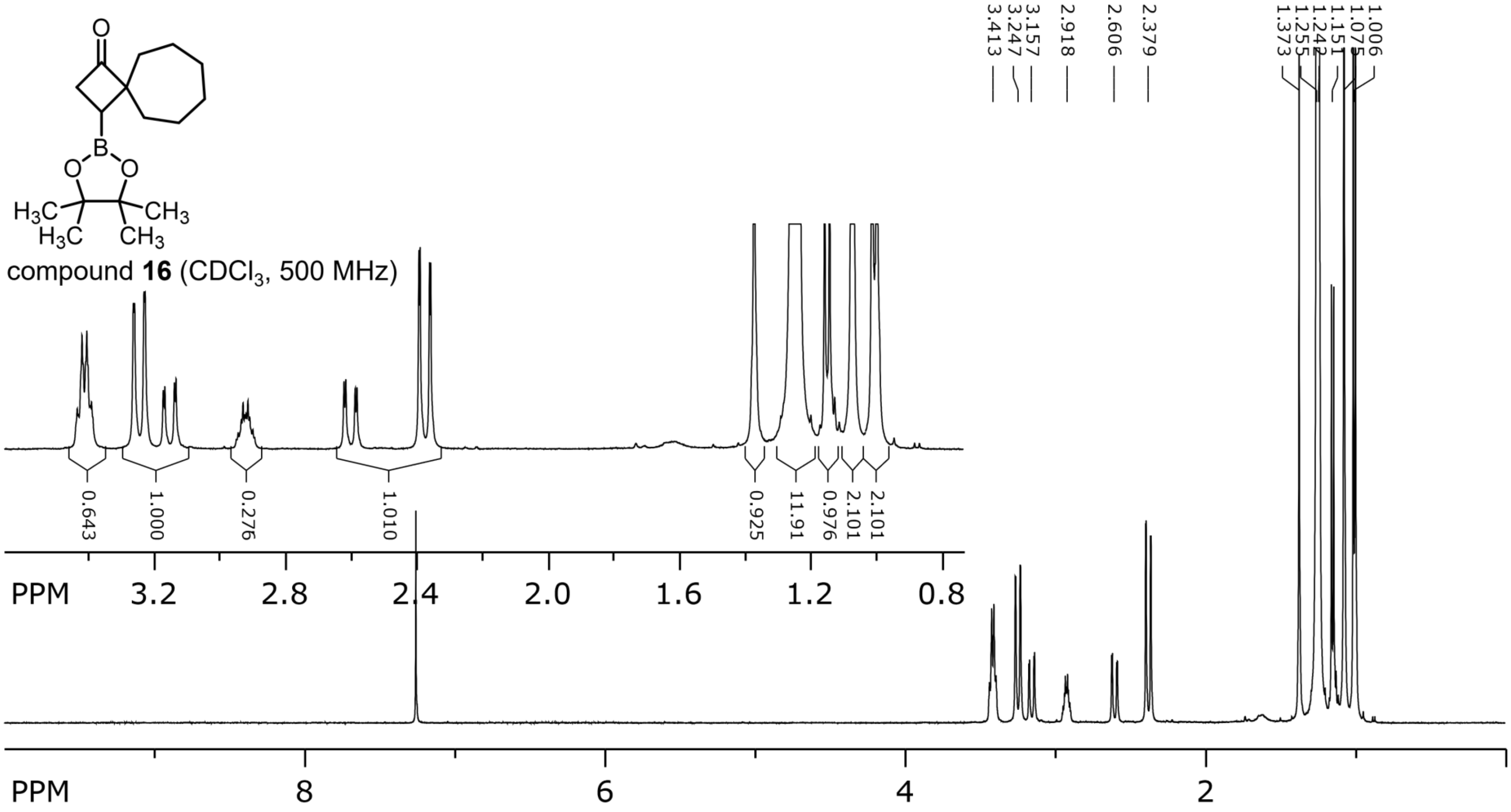

$^{13}C\{^1H\}$ *NMR for compound* ***16***

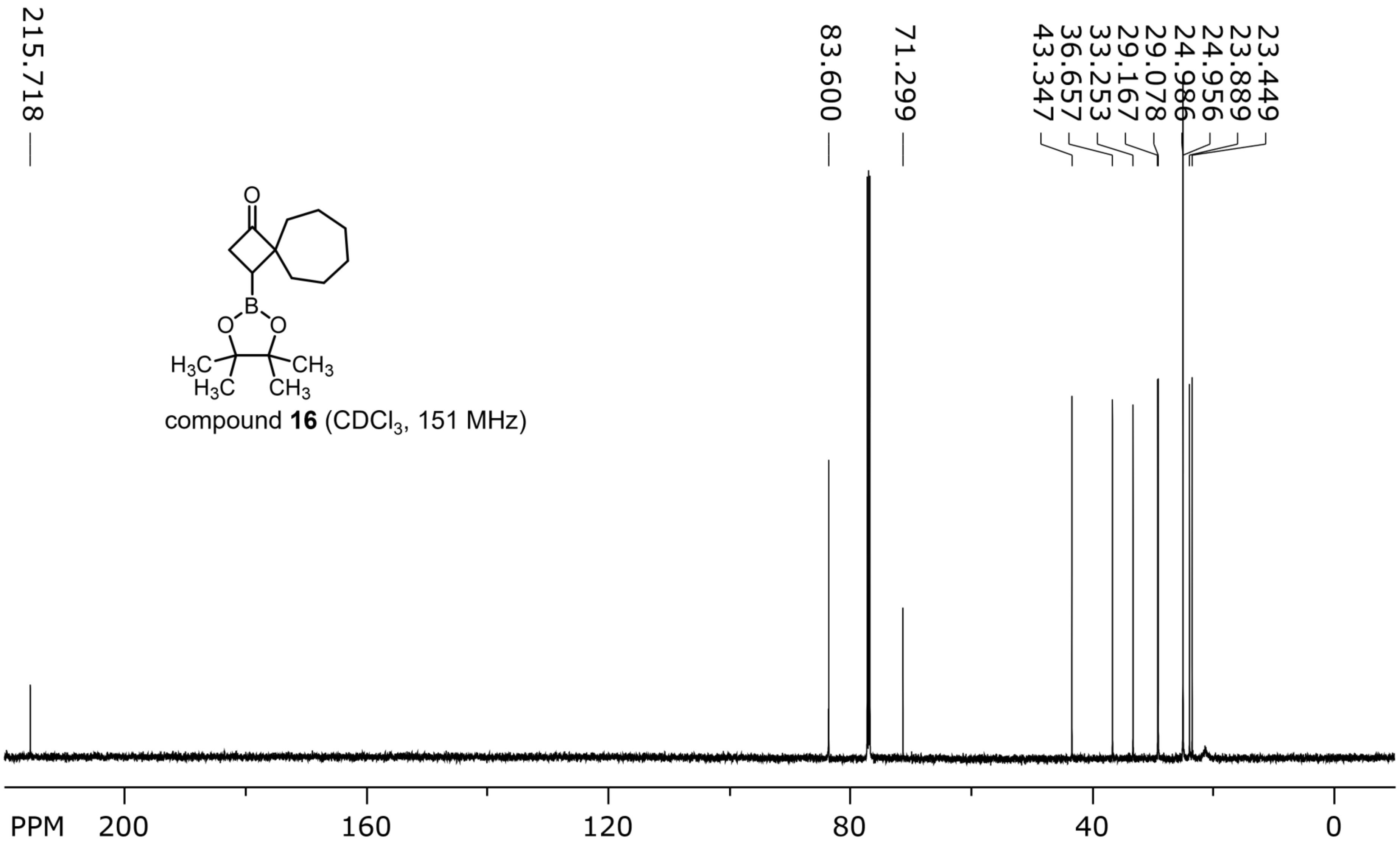

*^{1}H NMR for compound **17***

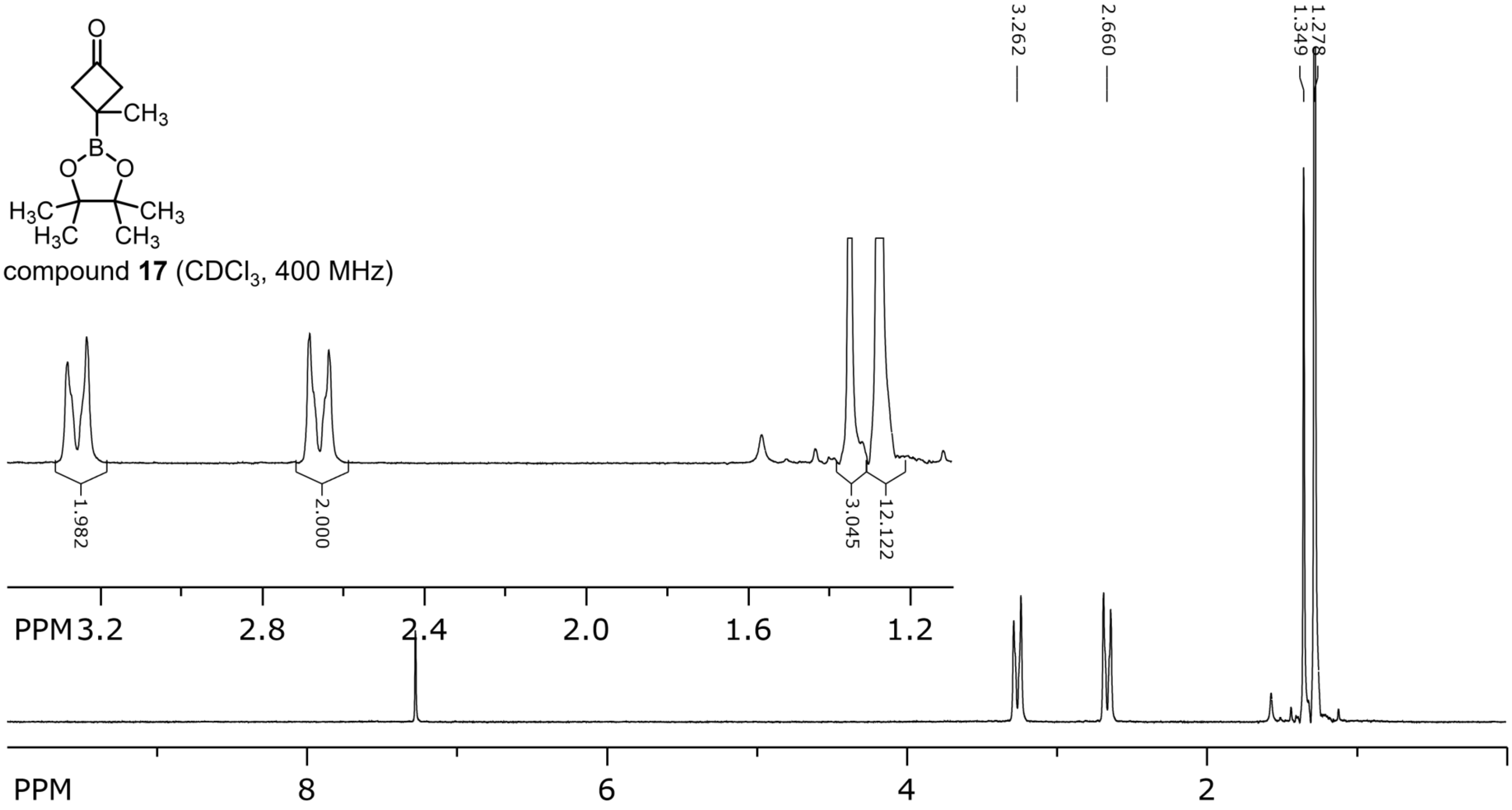

*^{13}C{^{1}H} NMR for compound **17***

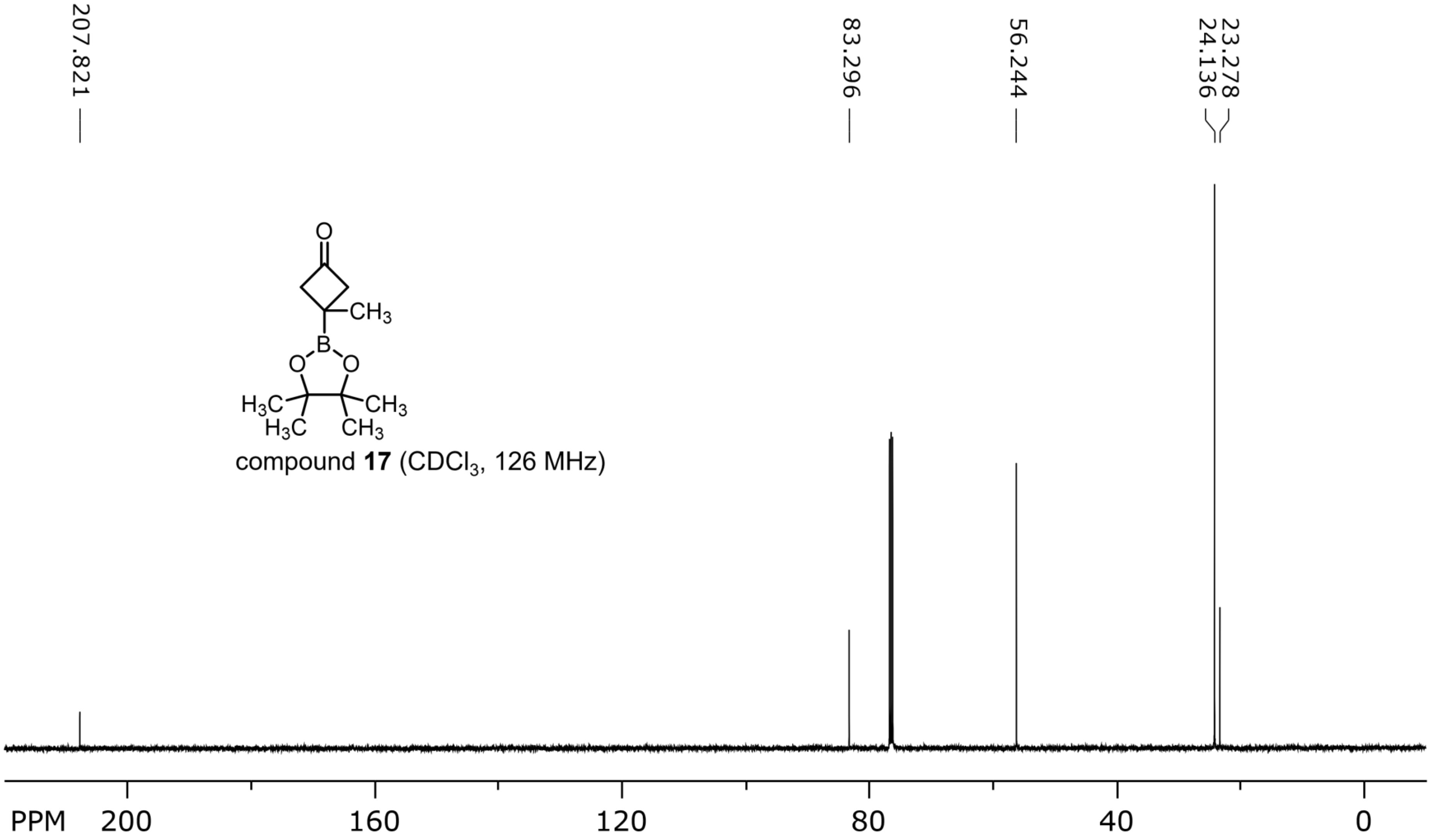

*^{1}H NMR for compound **18***

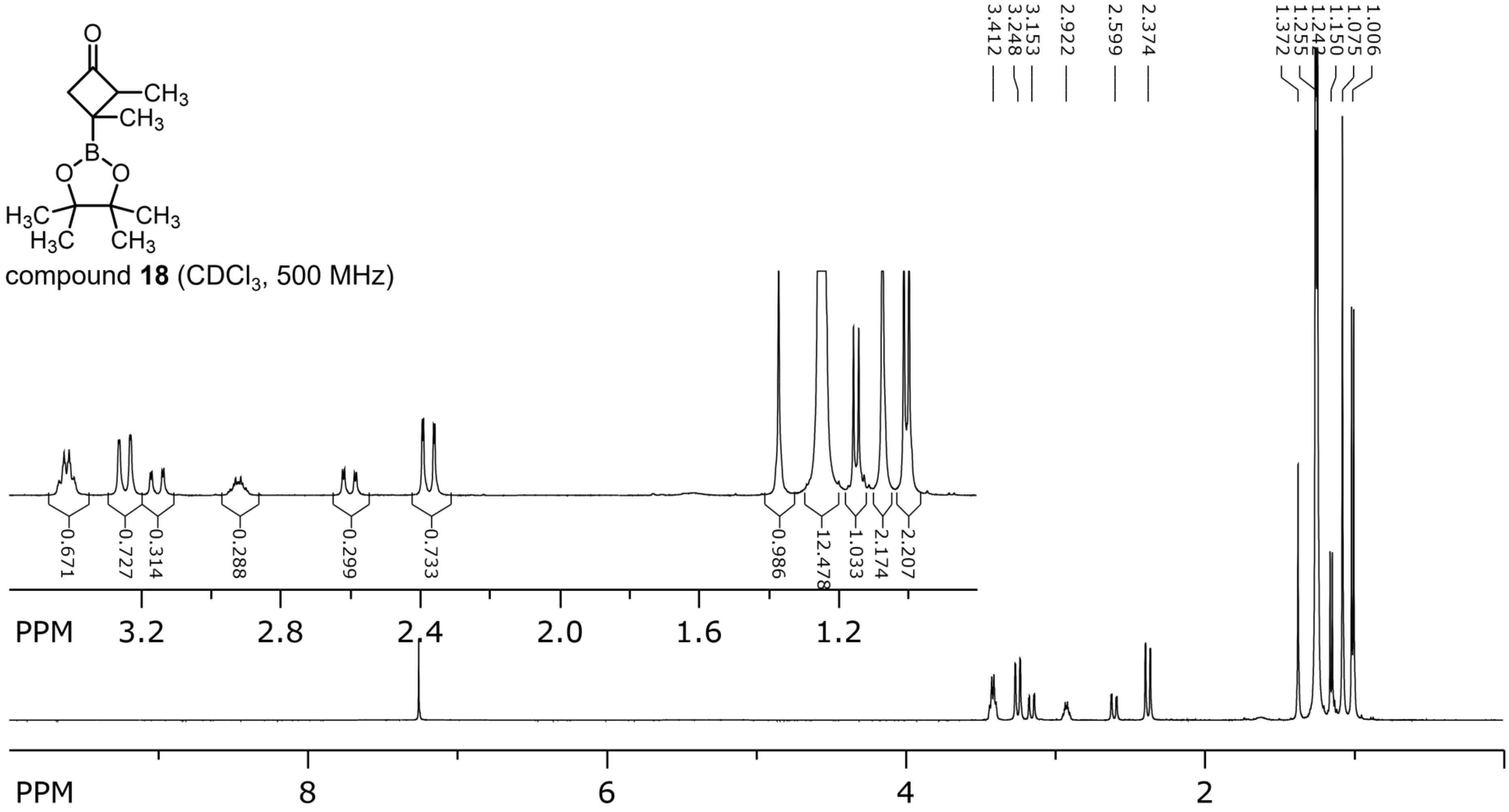

*$^{13}C\{^{1}H\}$ NMR for compound **18***

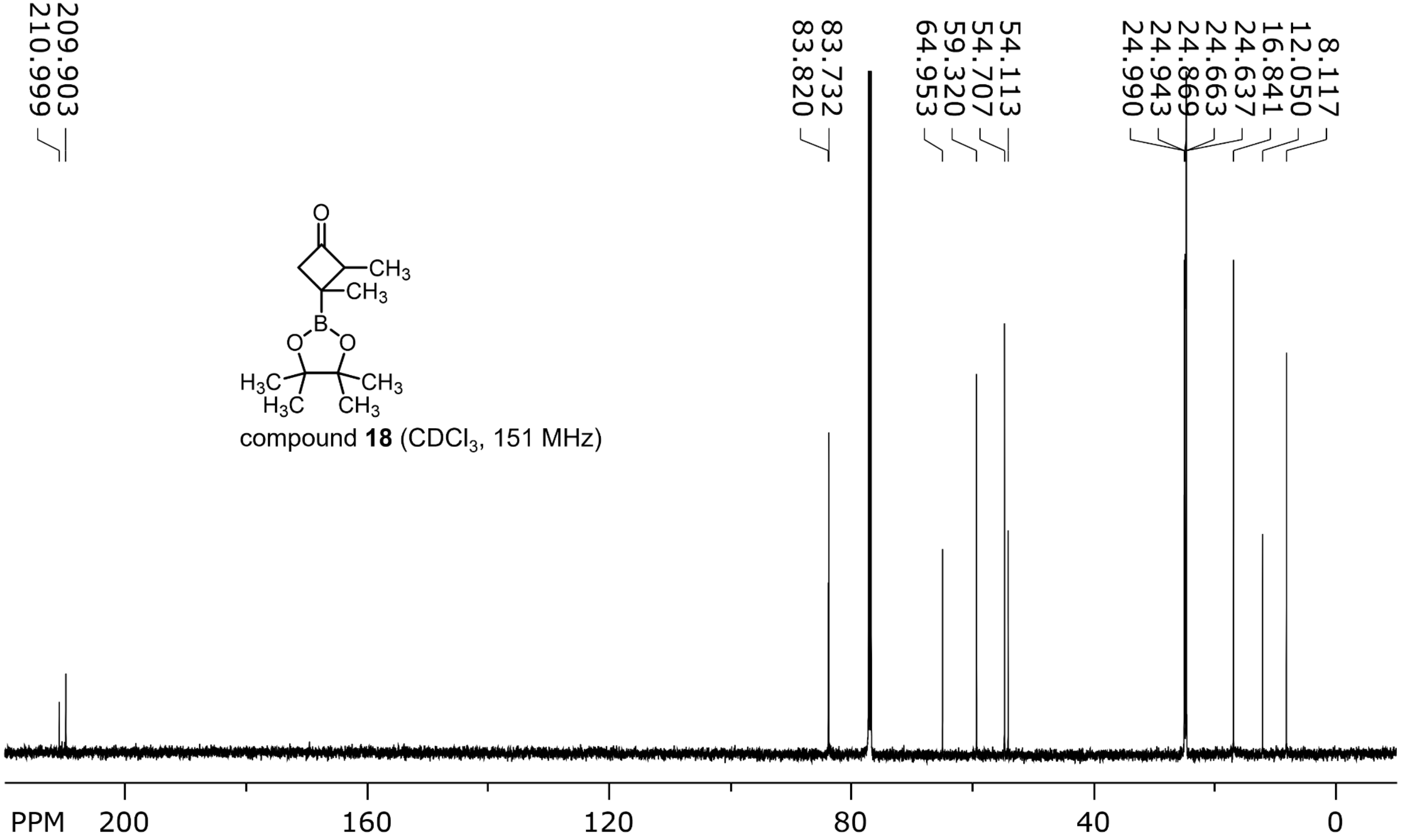

*^{1}H NMR for compound **19***

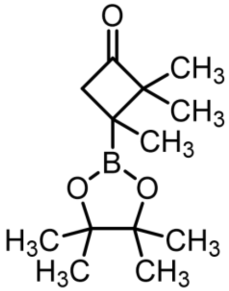

compound **19** ($CDCl_3$, 400 MHz)

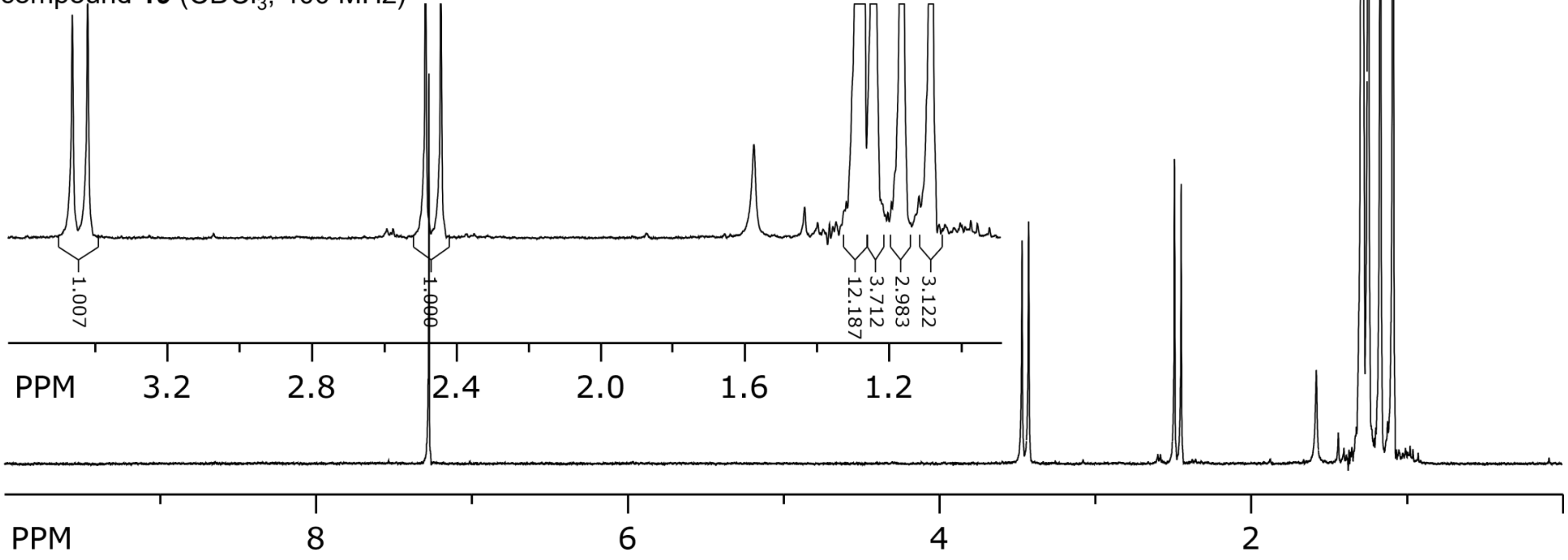

$^{13}C\{^1H\}$ NMR for compound ***19***

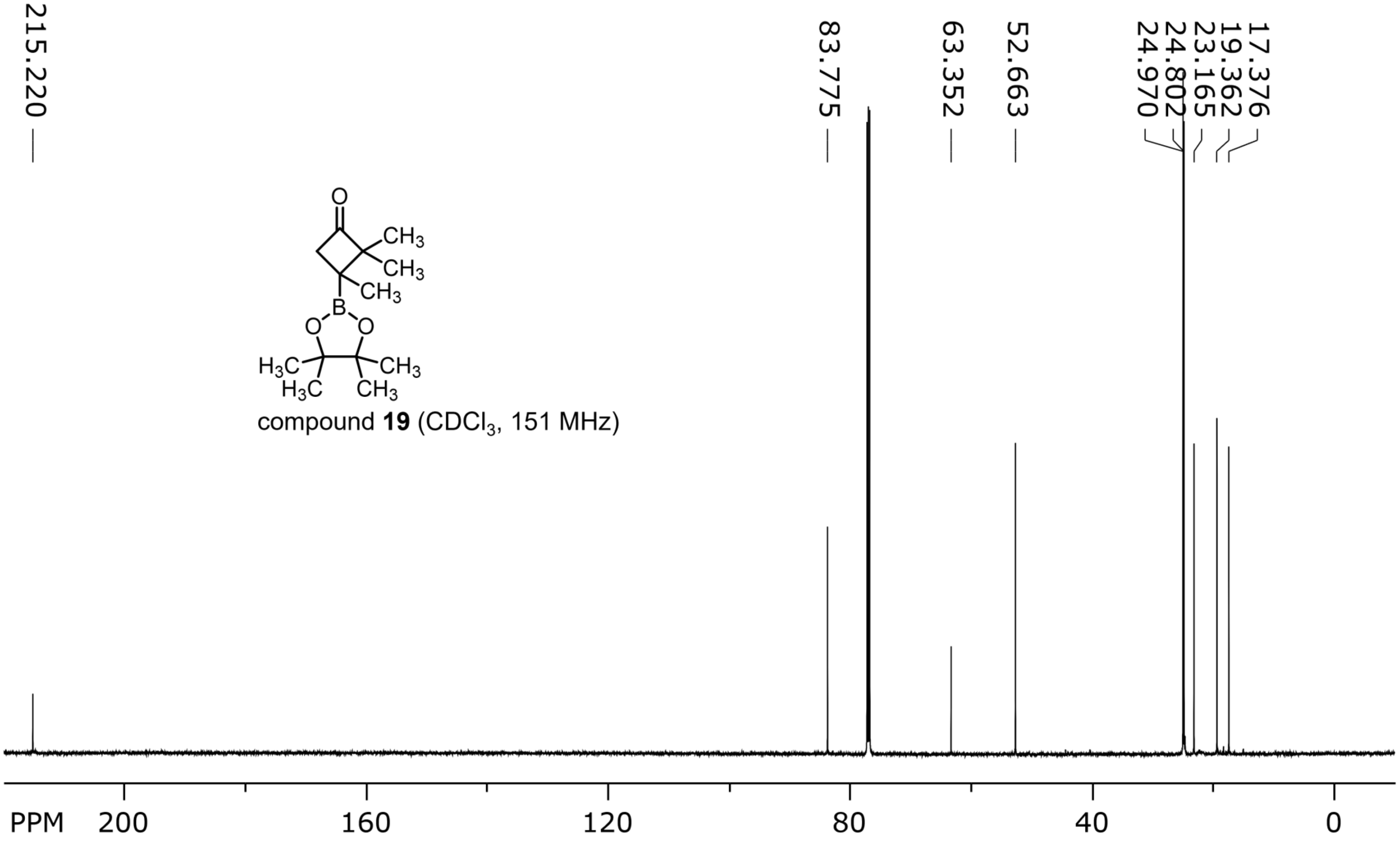

^{1}H NMR for compound ***20***

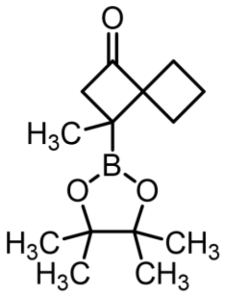

compound **20** ($CDCl_3$, 500 MHz)

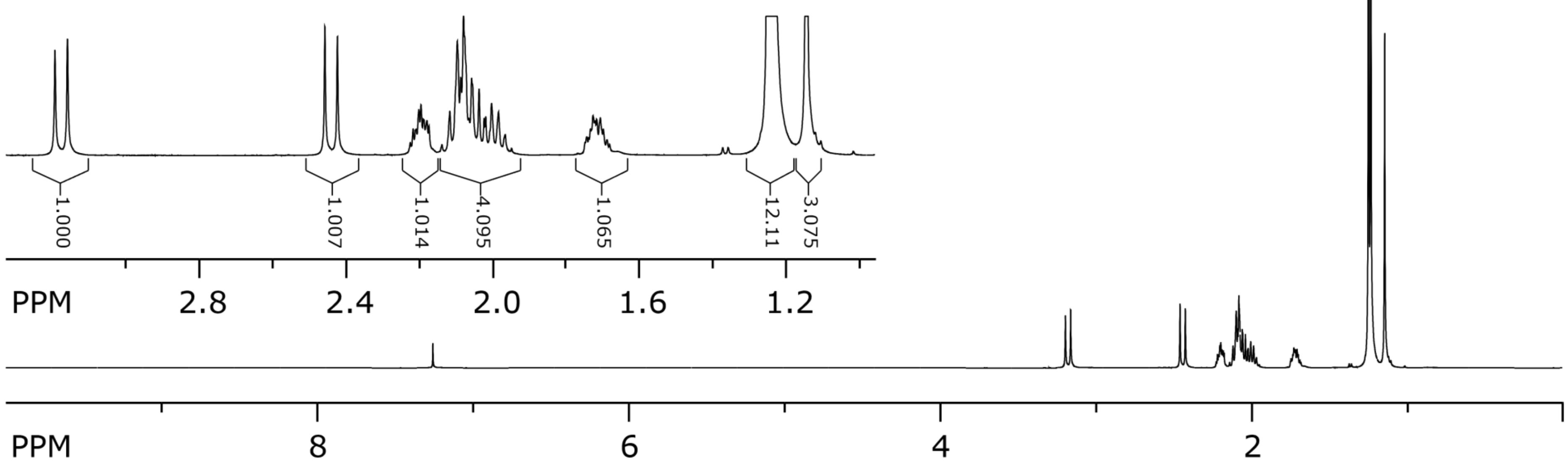

$^{13}C\{^1H\}$ *NMR for compound* ***20***

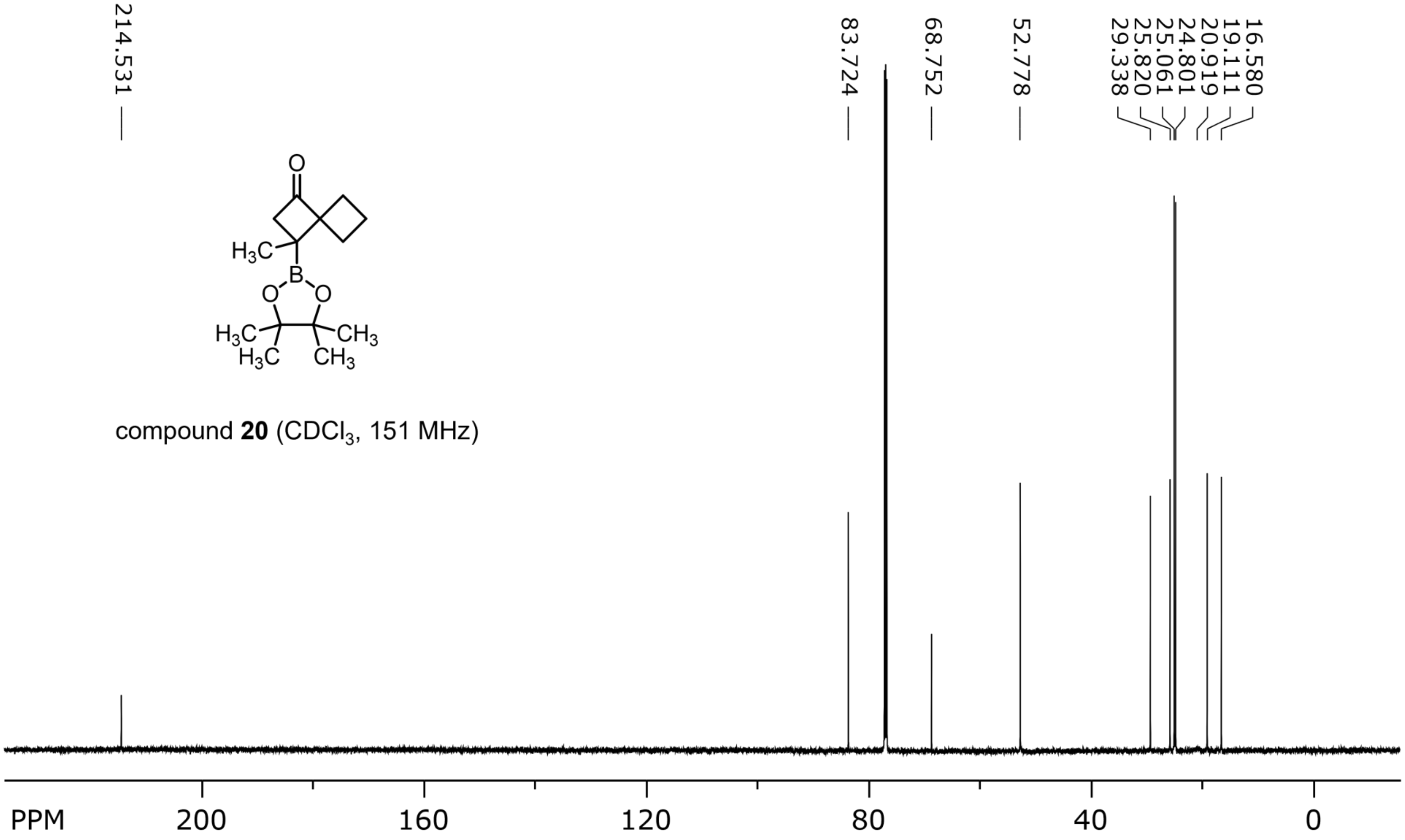

*^{1}H NMR for compound **21***

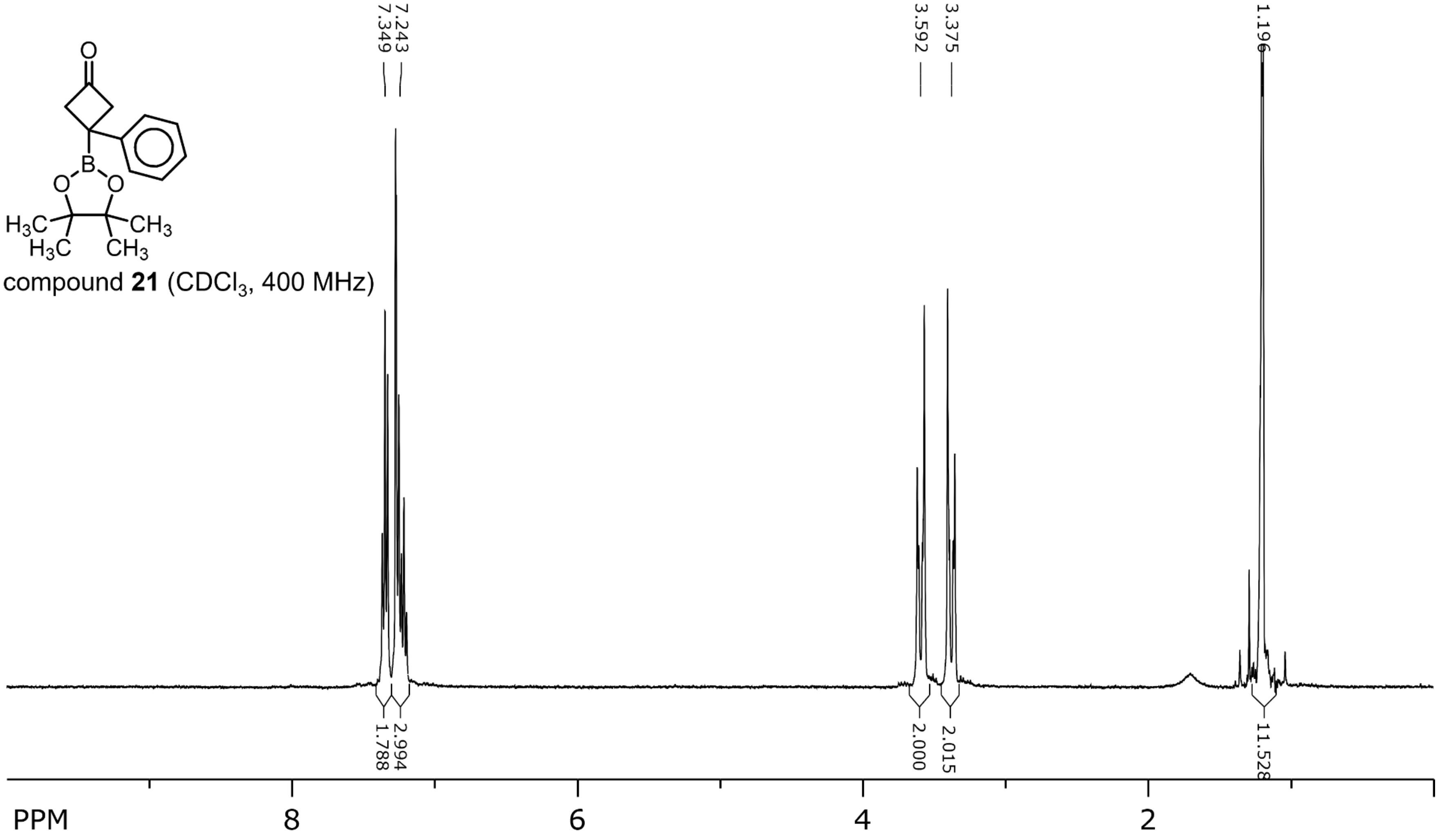

$^{13}C\{^1H\}$ *NMR for* compound ***21***

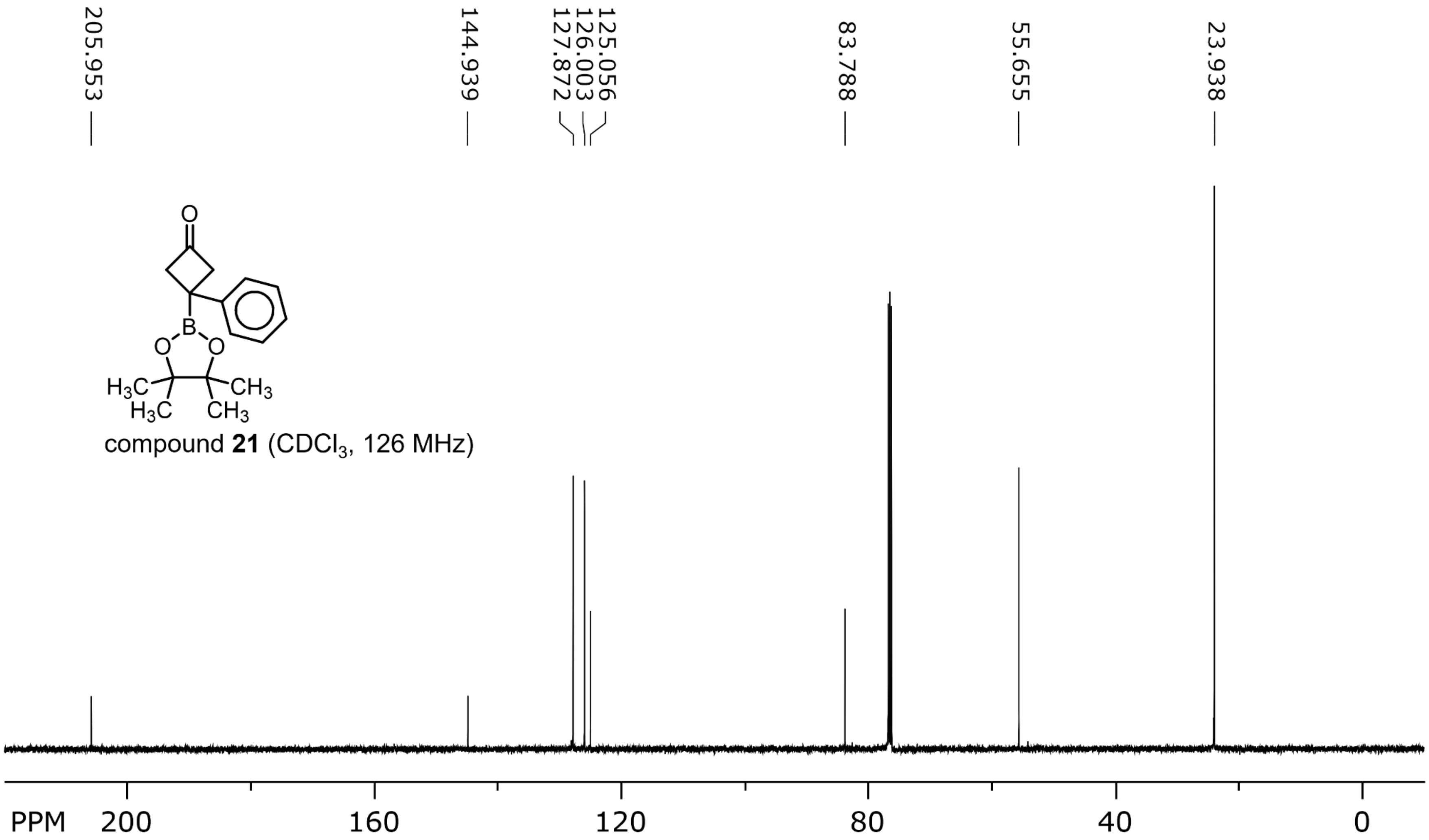

*^{1}H NMR for compound **22***

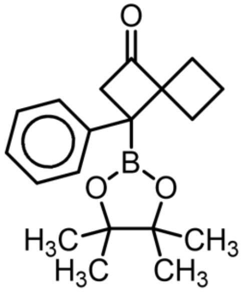

compound **22** ($CDCl_3$, 500 MHz)

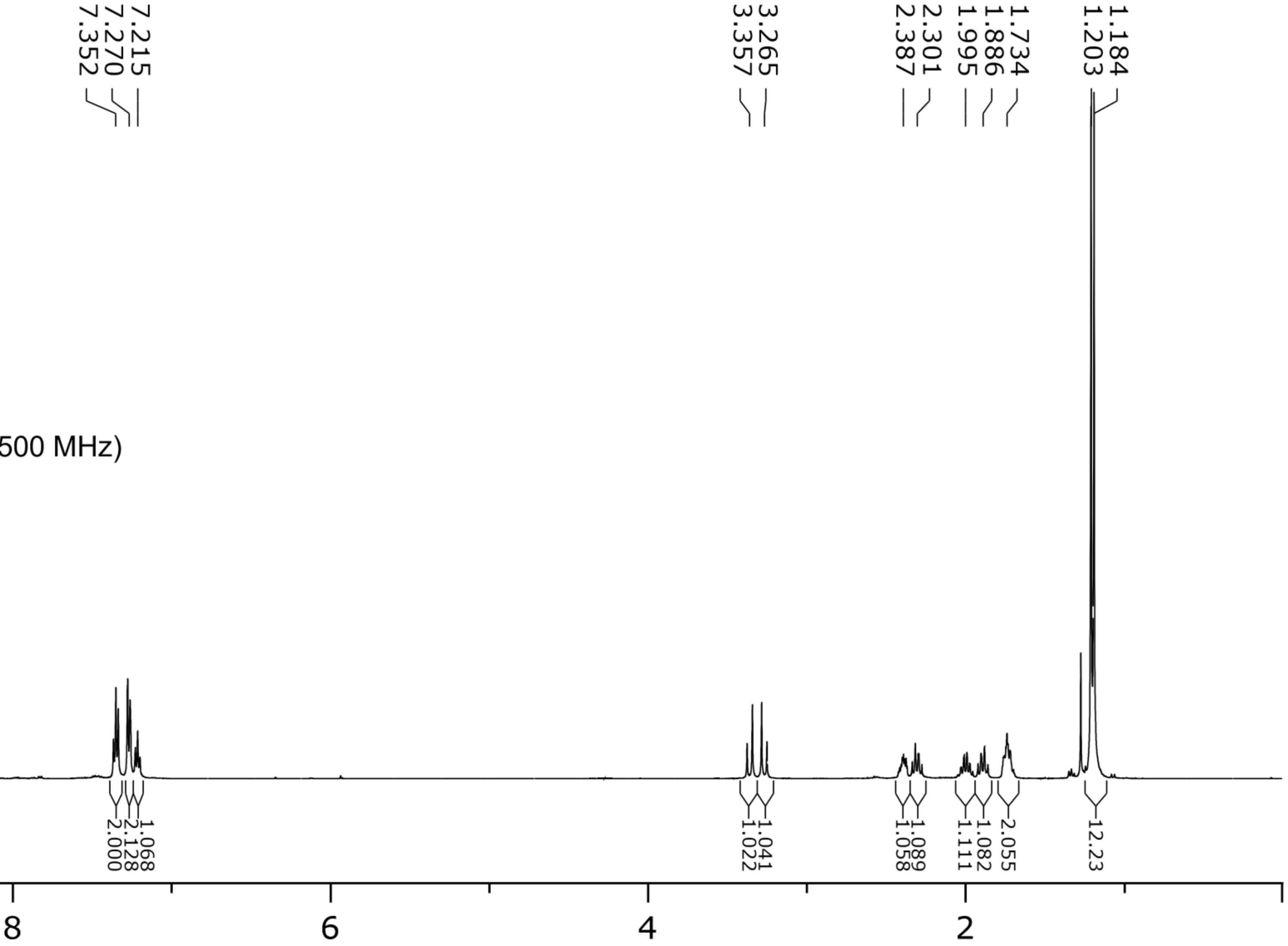

$^{13}C\{^1H\}$ *NMR for compound* ***22***

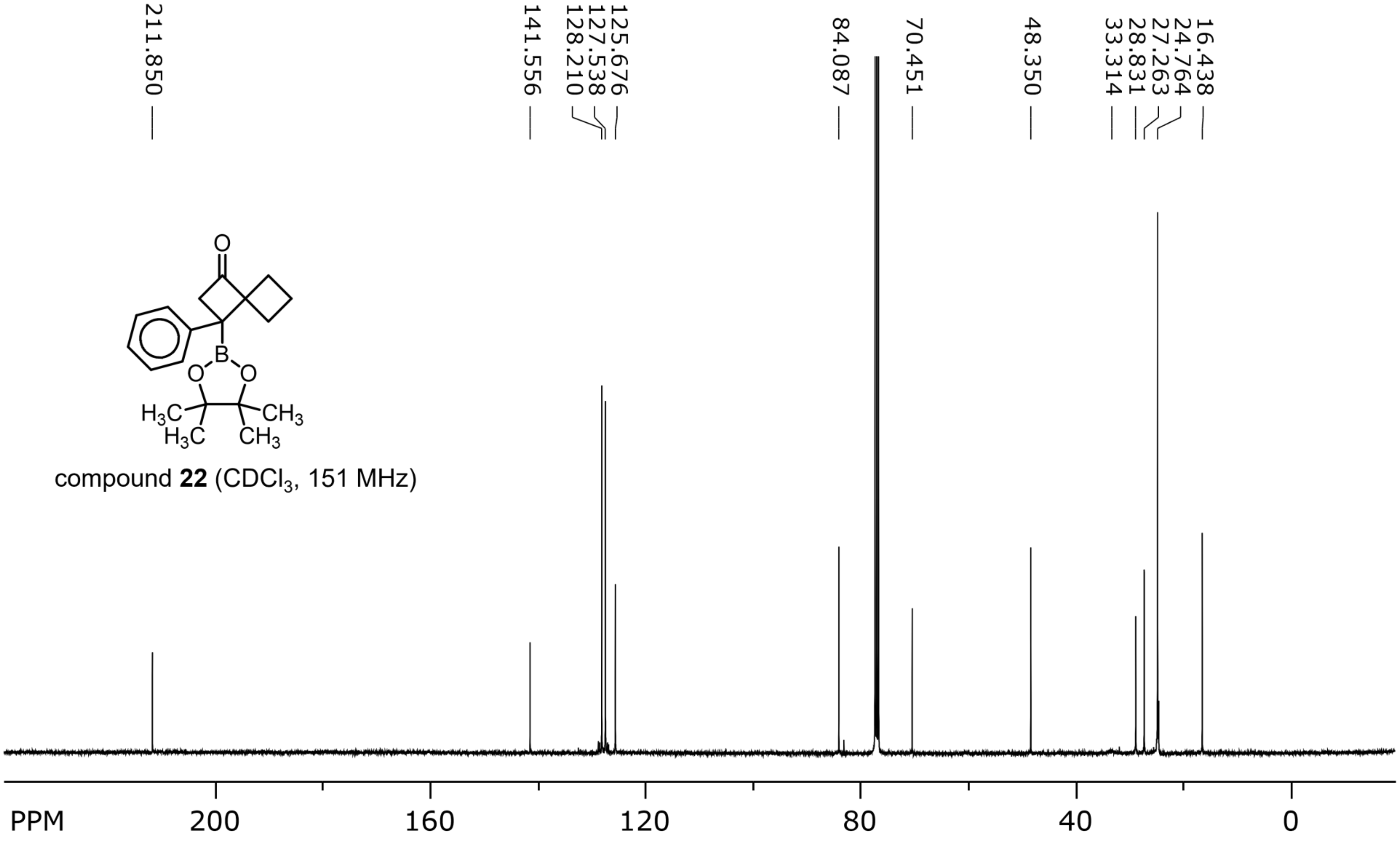

[1]*H NMR for compound* ***25***

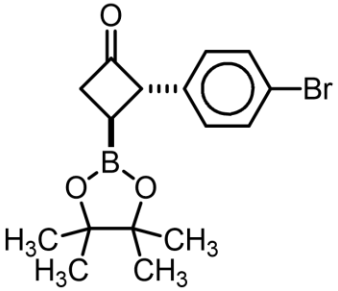

compound **25** ($CDCl_3$, 500 MHz)

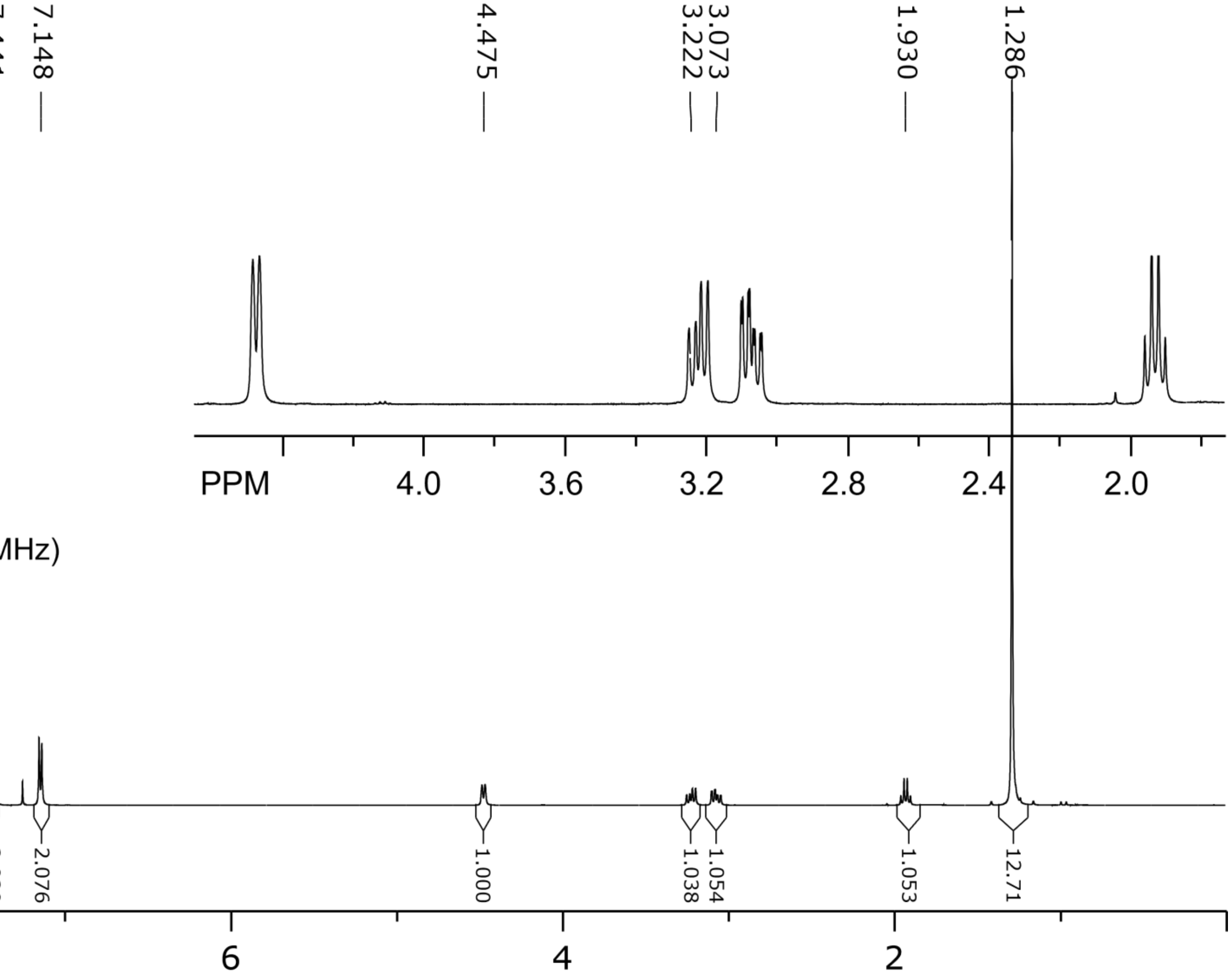

*^{13}C{^{1}H} NMR for compound **25***

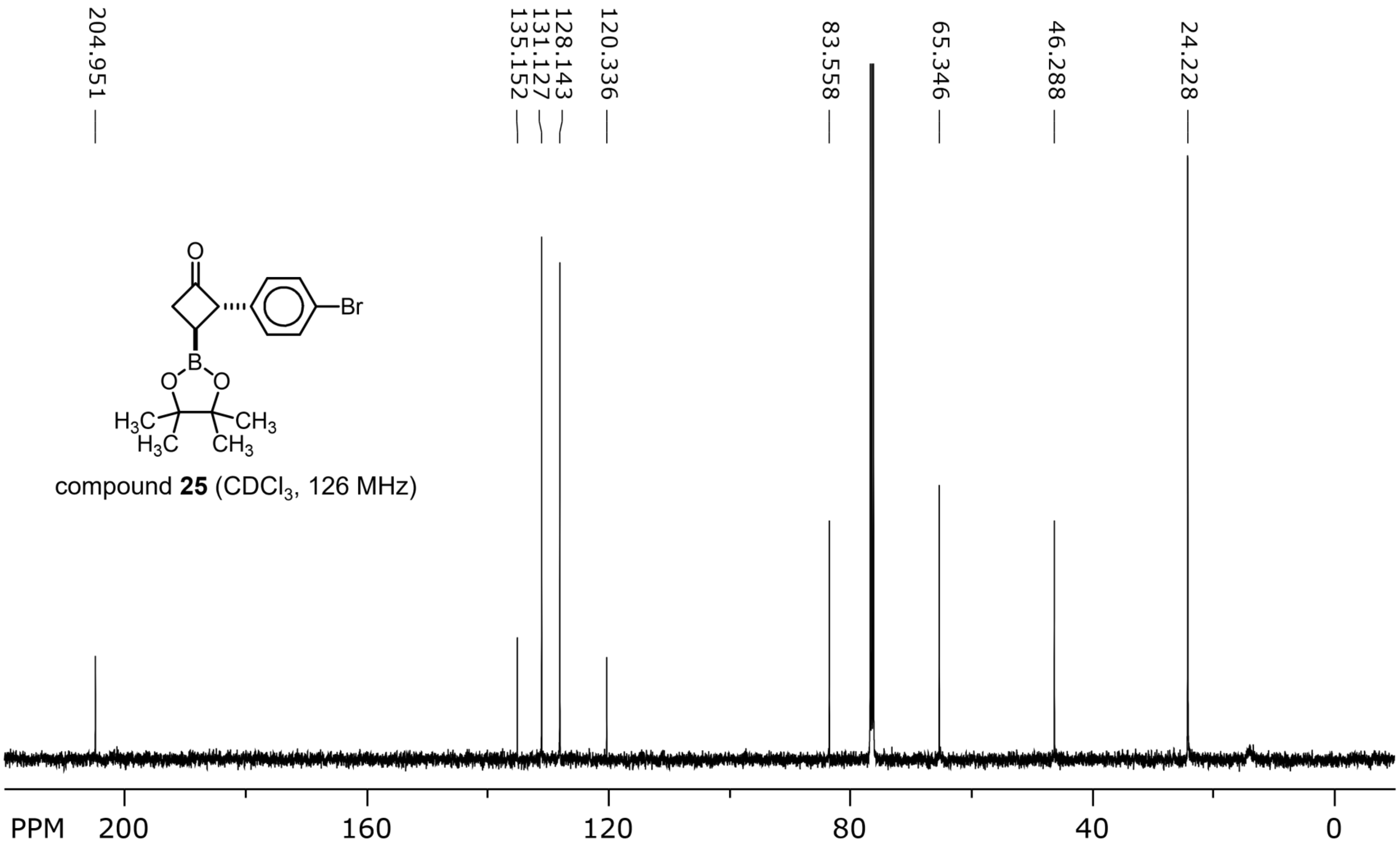

*^{1}H NMR for compound **26***

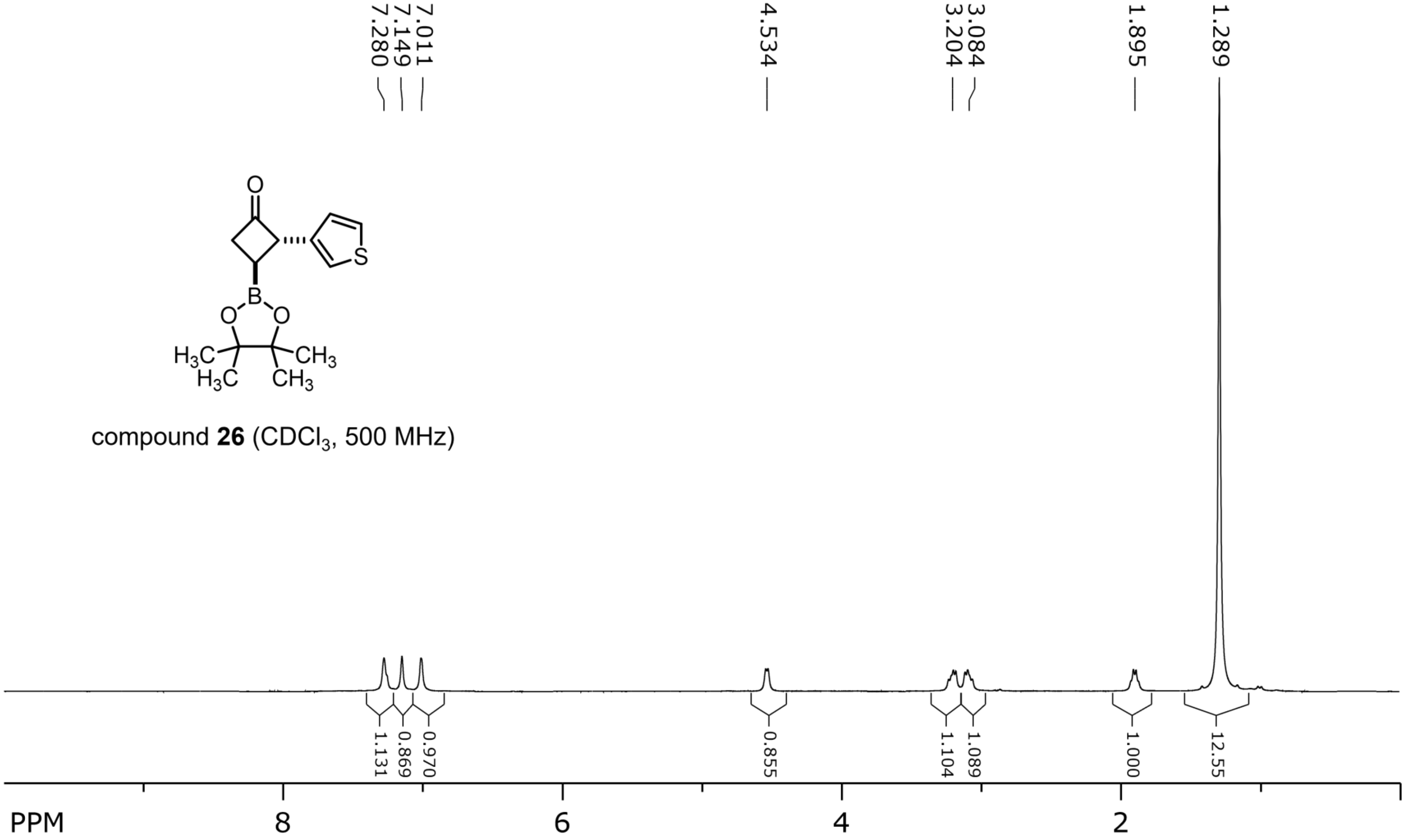

*^{13}C{^{1}H} NMR for compound **26***

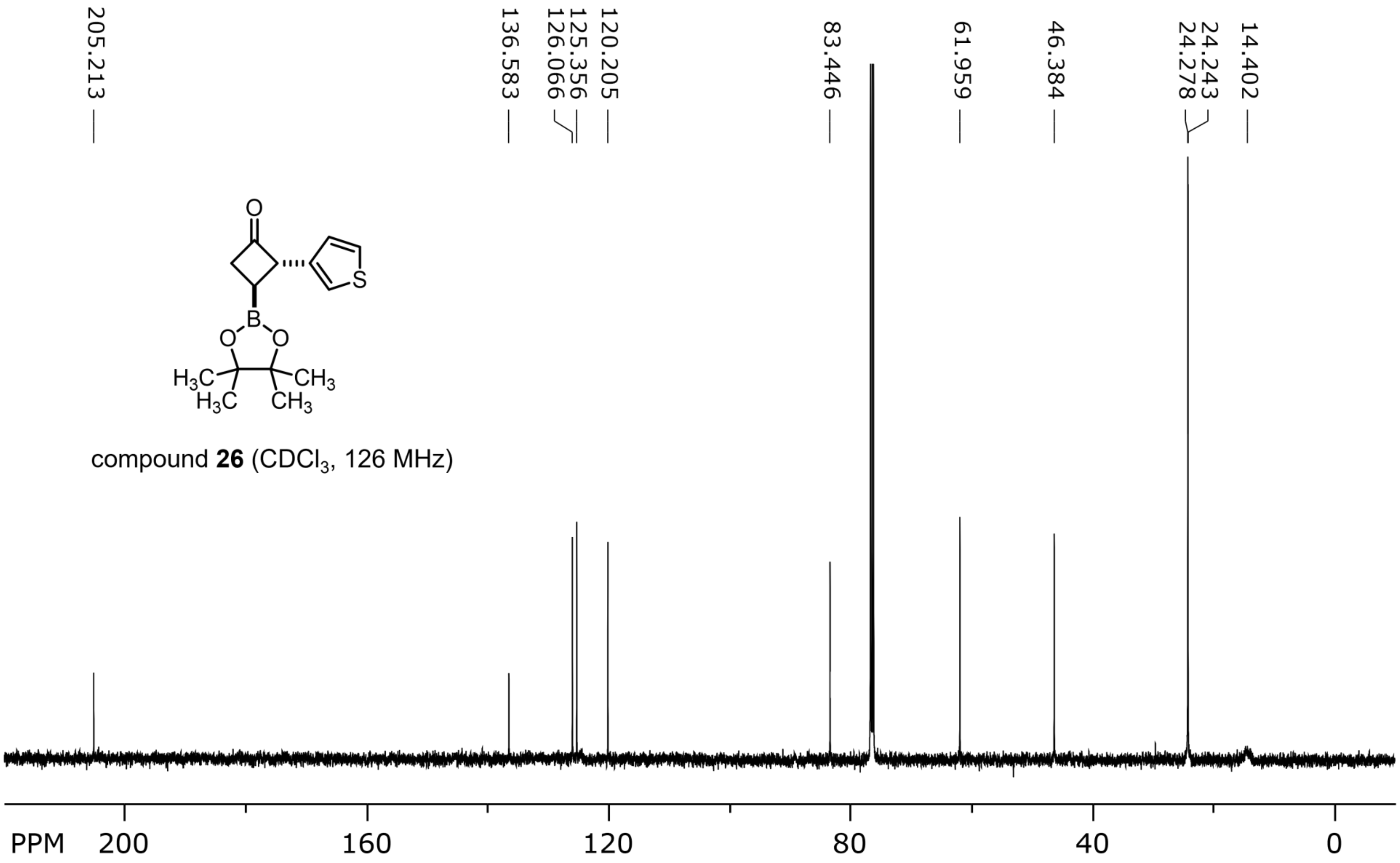

^{1}H NMR for compound ***28***

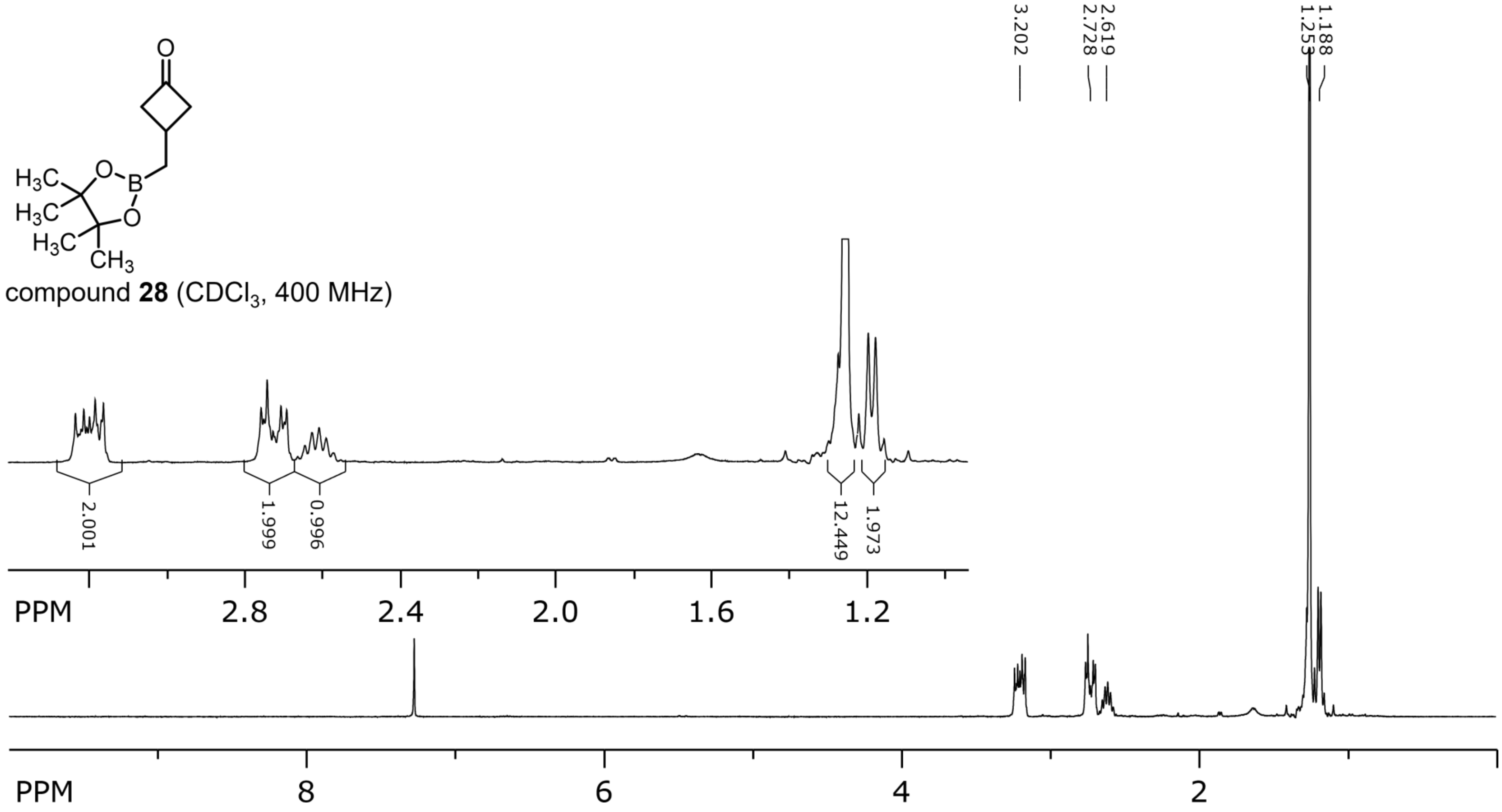

^{13}C{^{1}H} NMR for compound ***28***

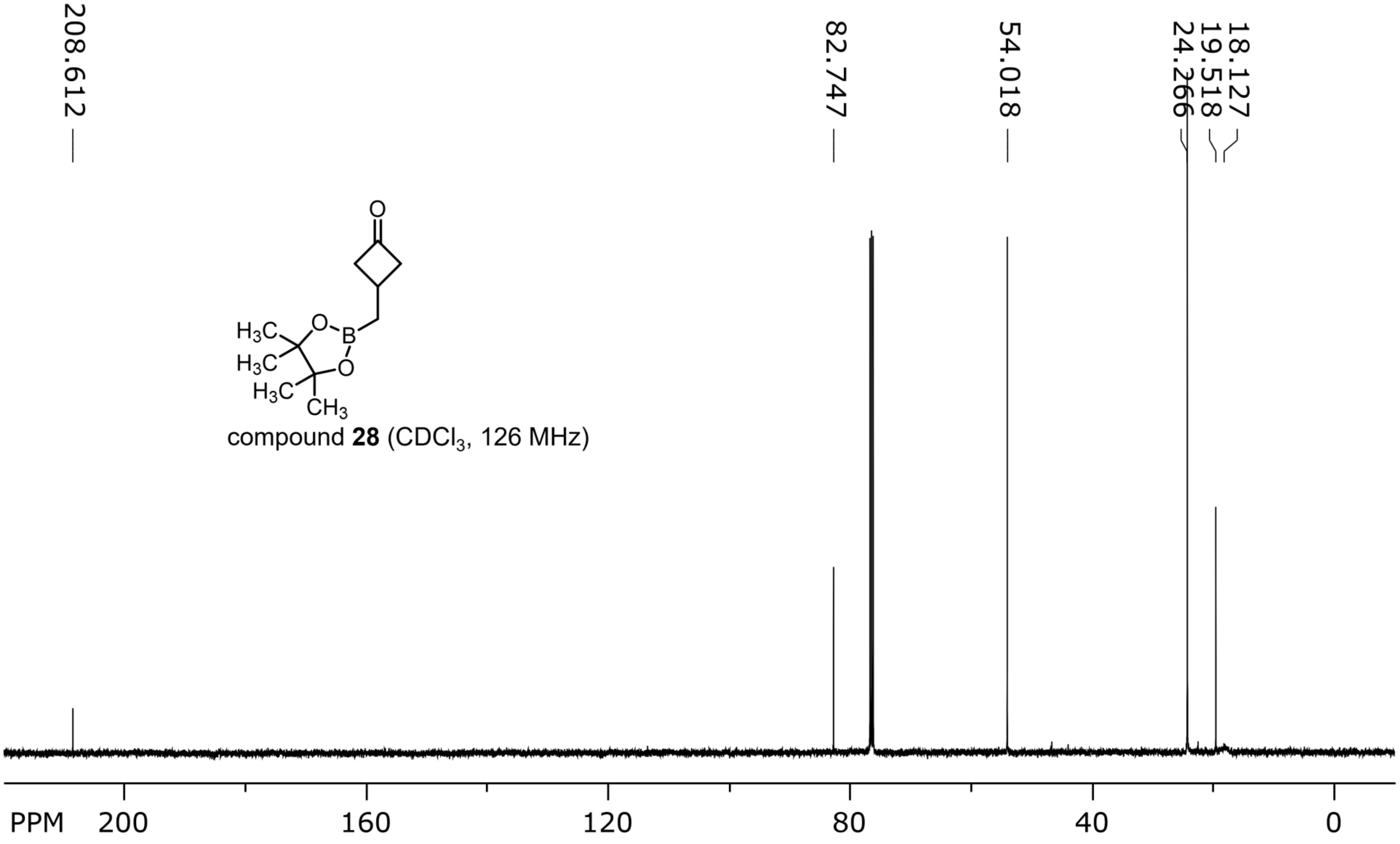

^{1}*H NMR for compound **29***

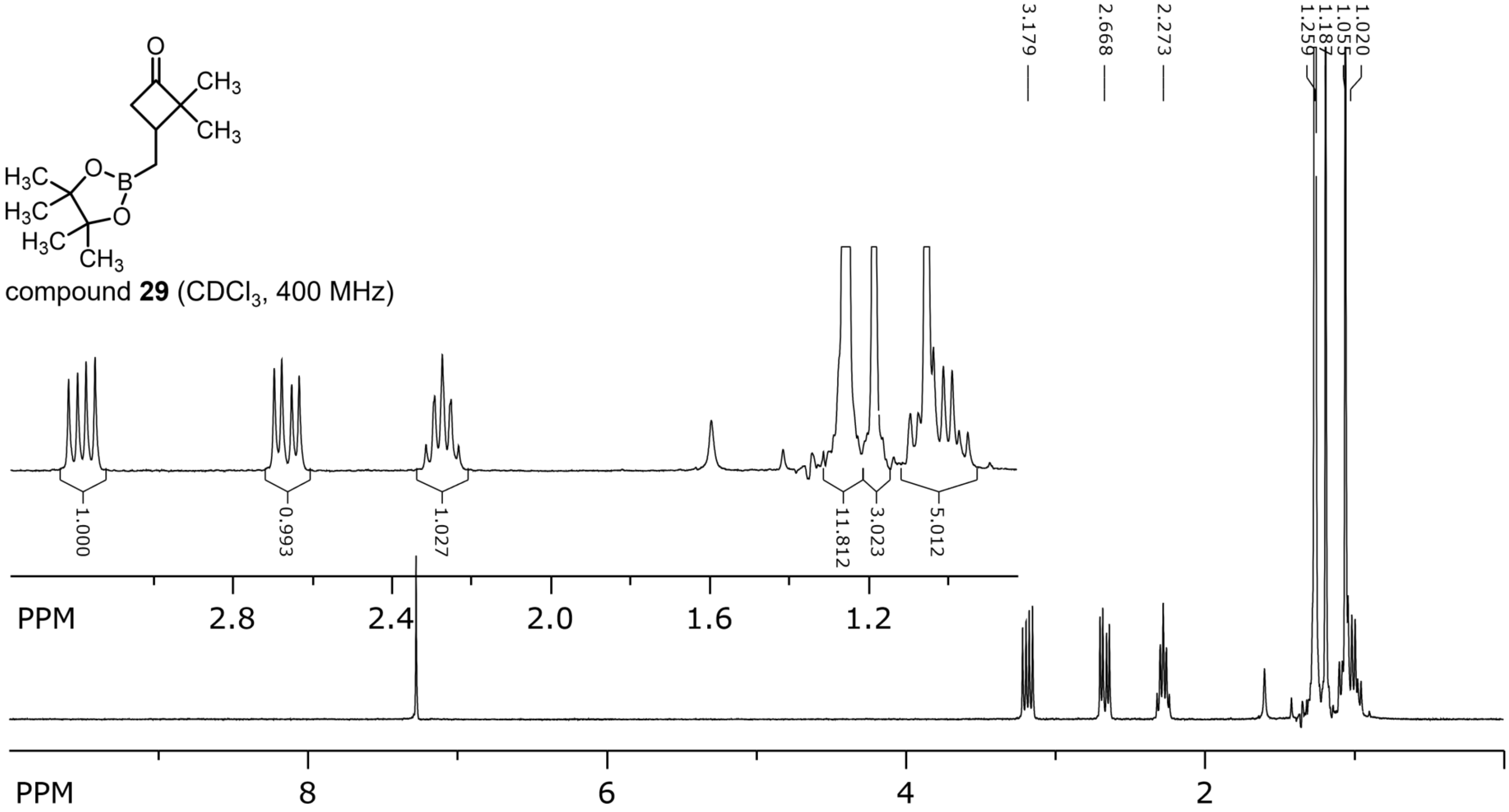

*^{13}C{^{1}H} NMR for compound **29***

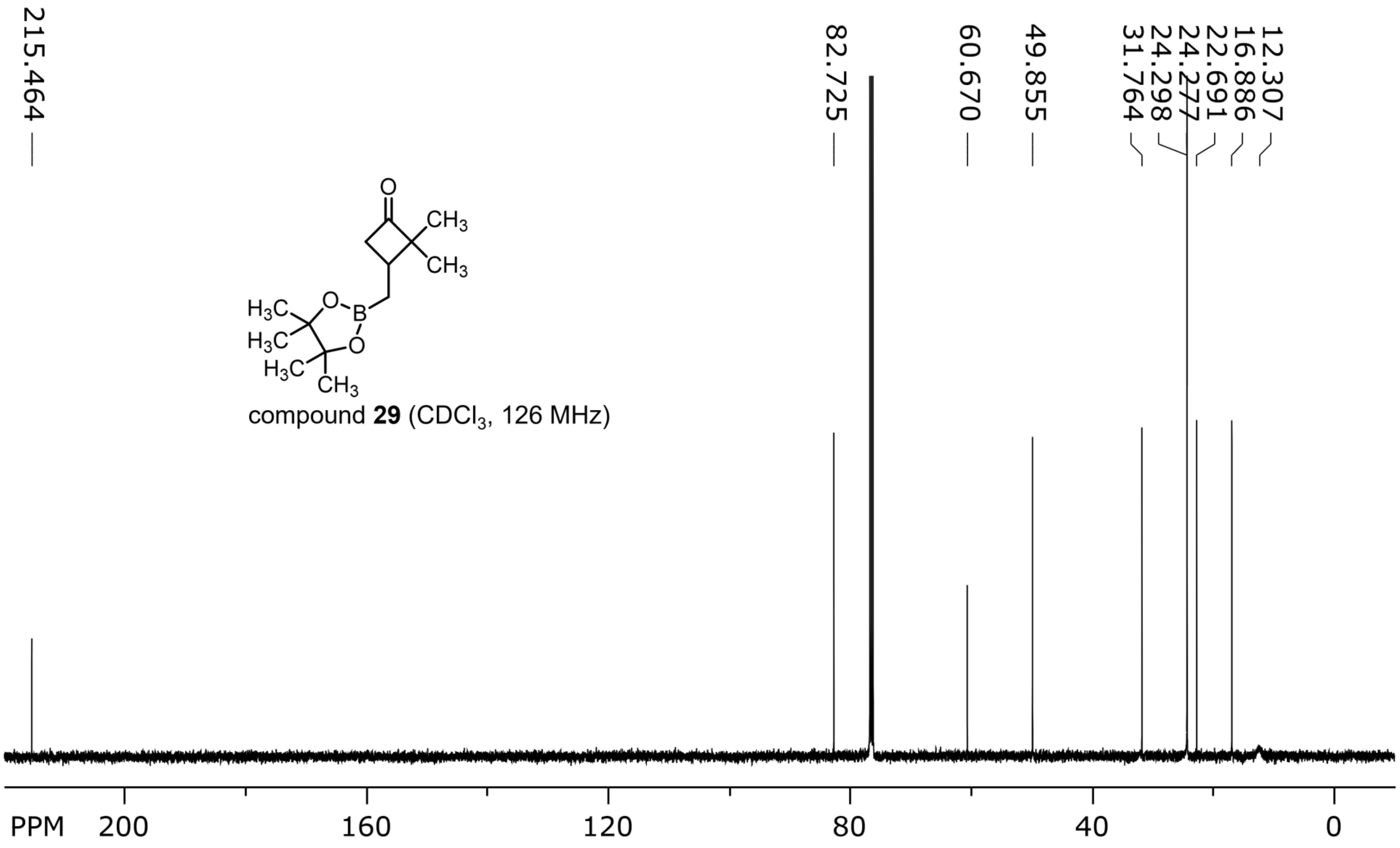

*^{1}H NMR for compound **30***

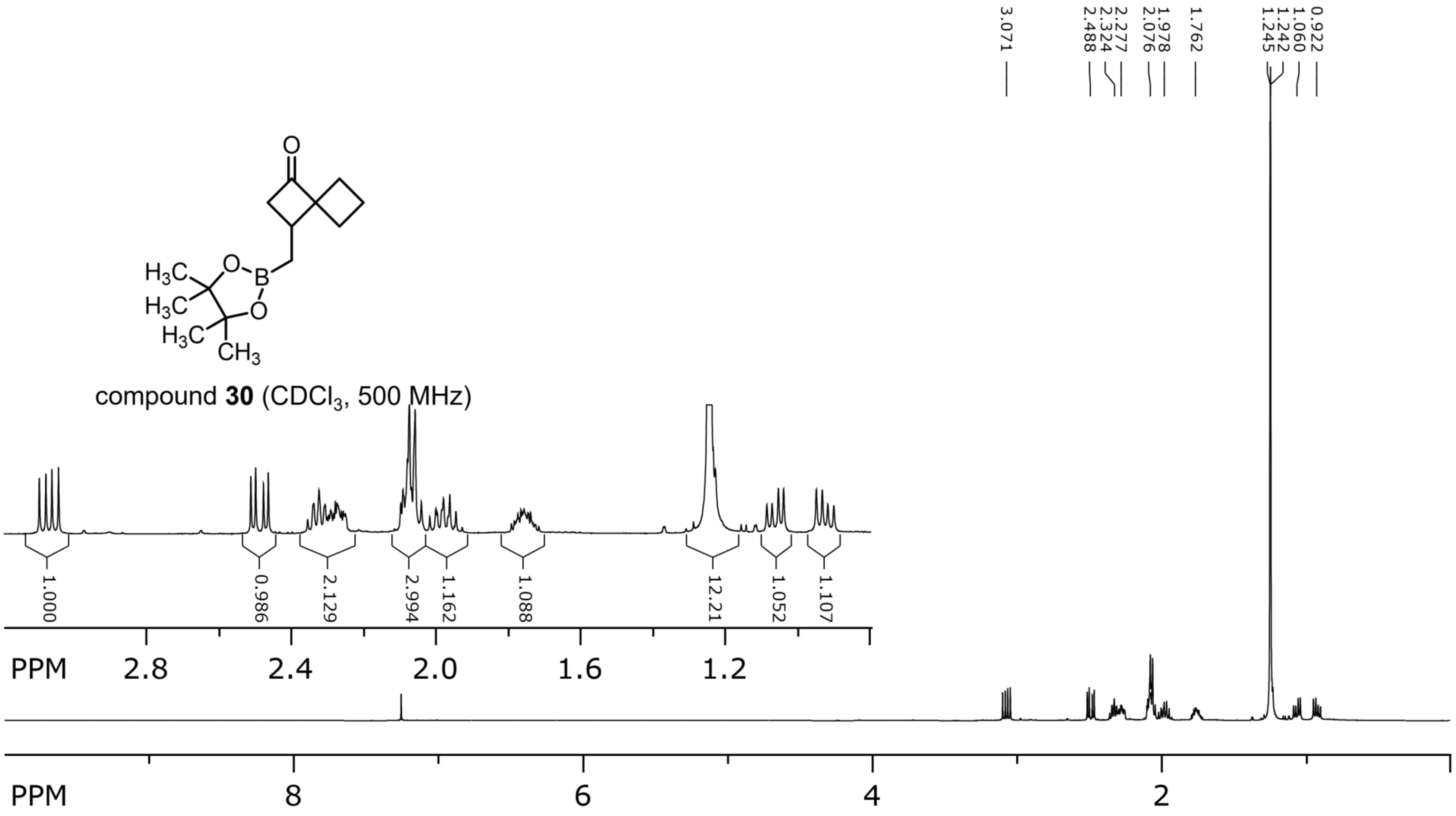

*$^{13}C\{^{1}H\}$ NMR for compound **30***

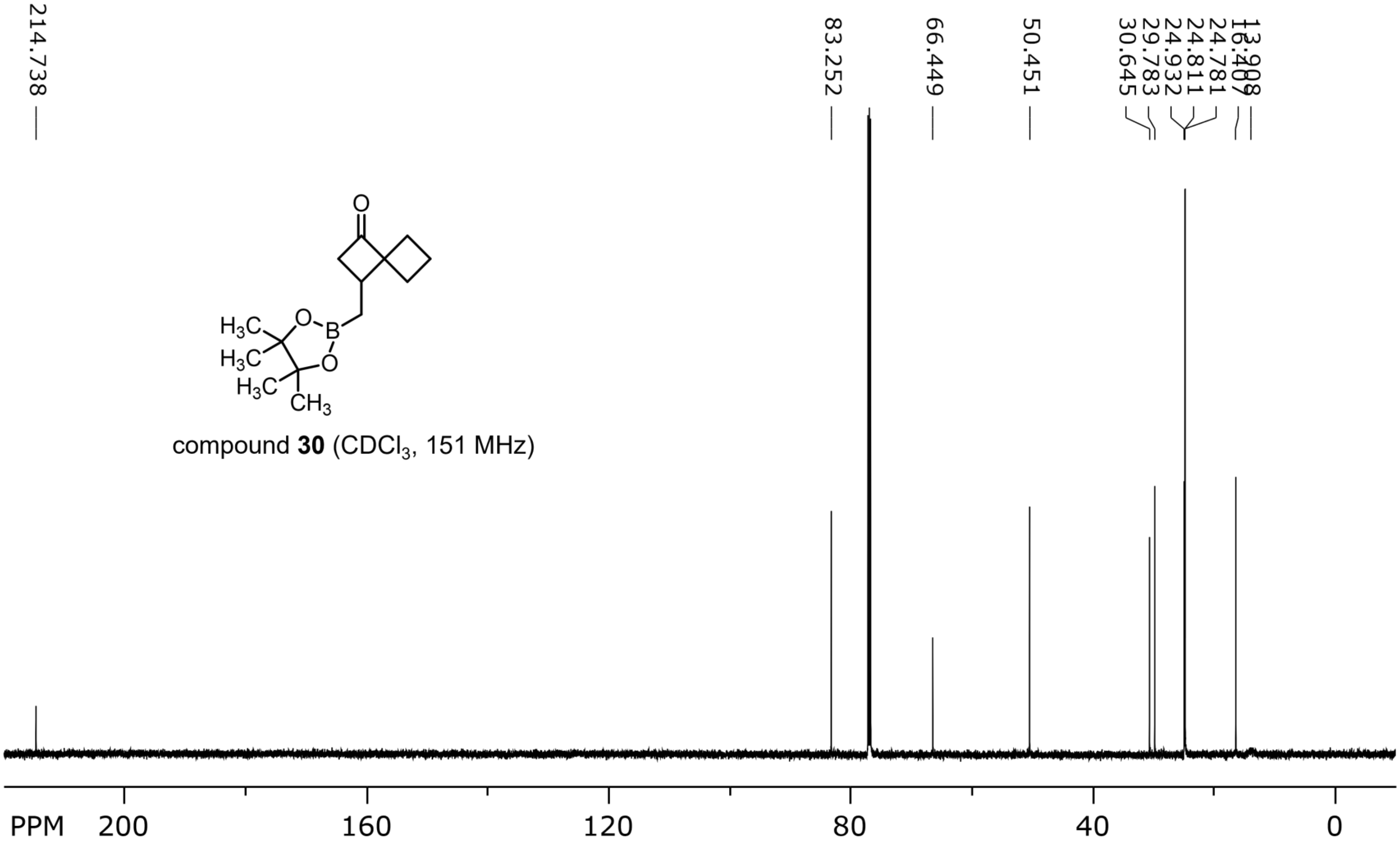

^{1}H NMR for compound ***31***

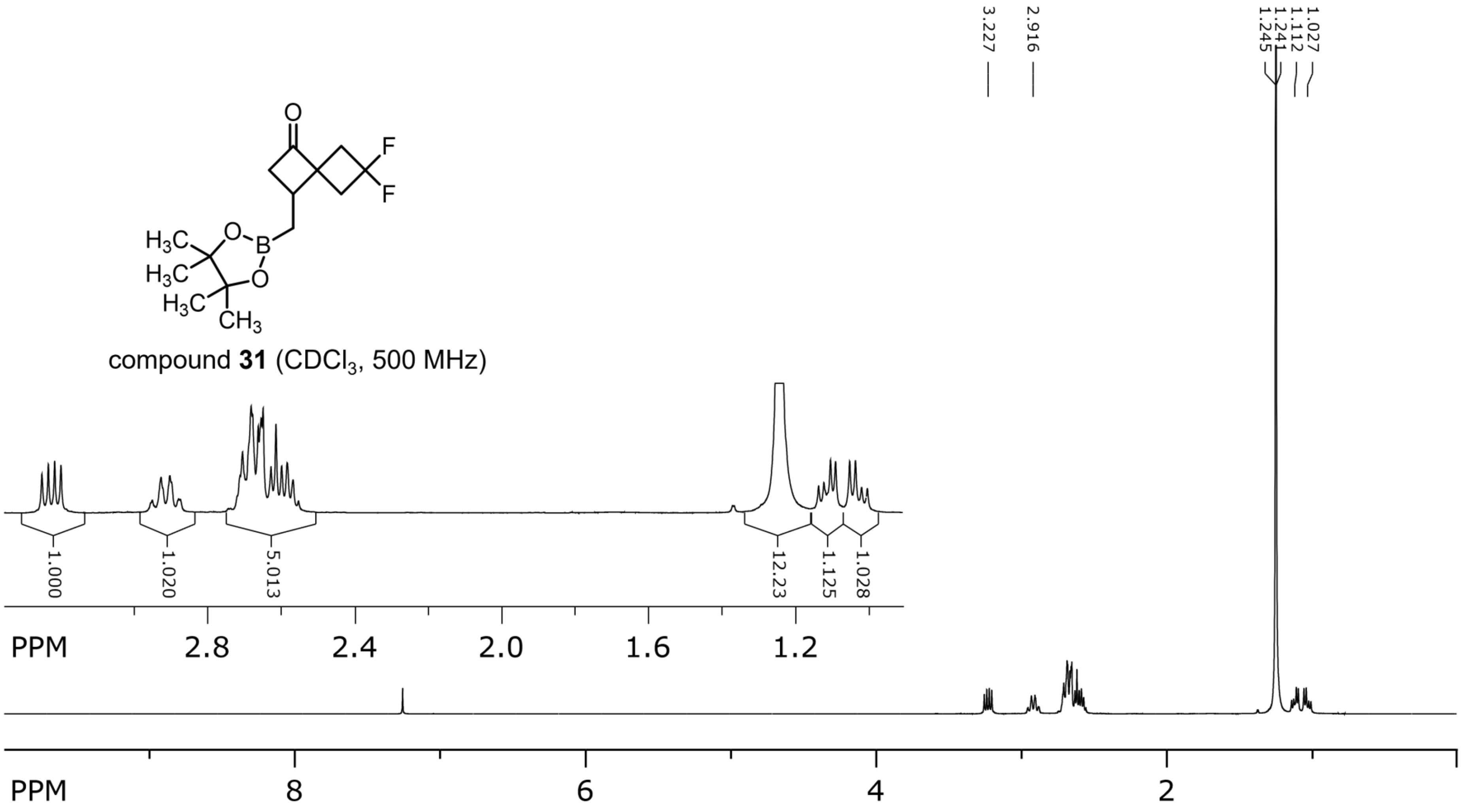

*$^{13}C\{^{1}H\}$ NMR for compound **31***

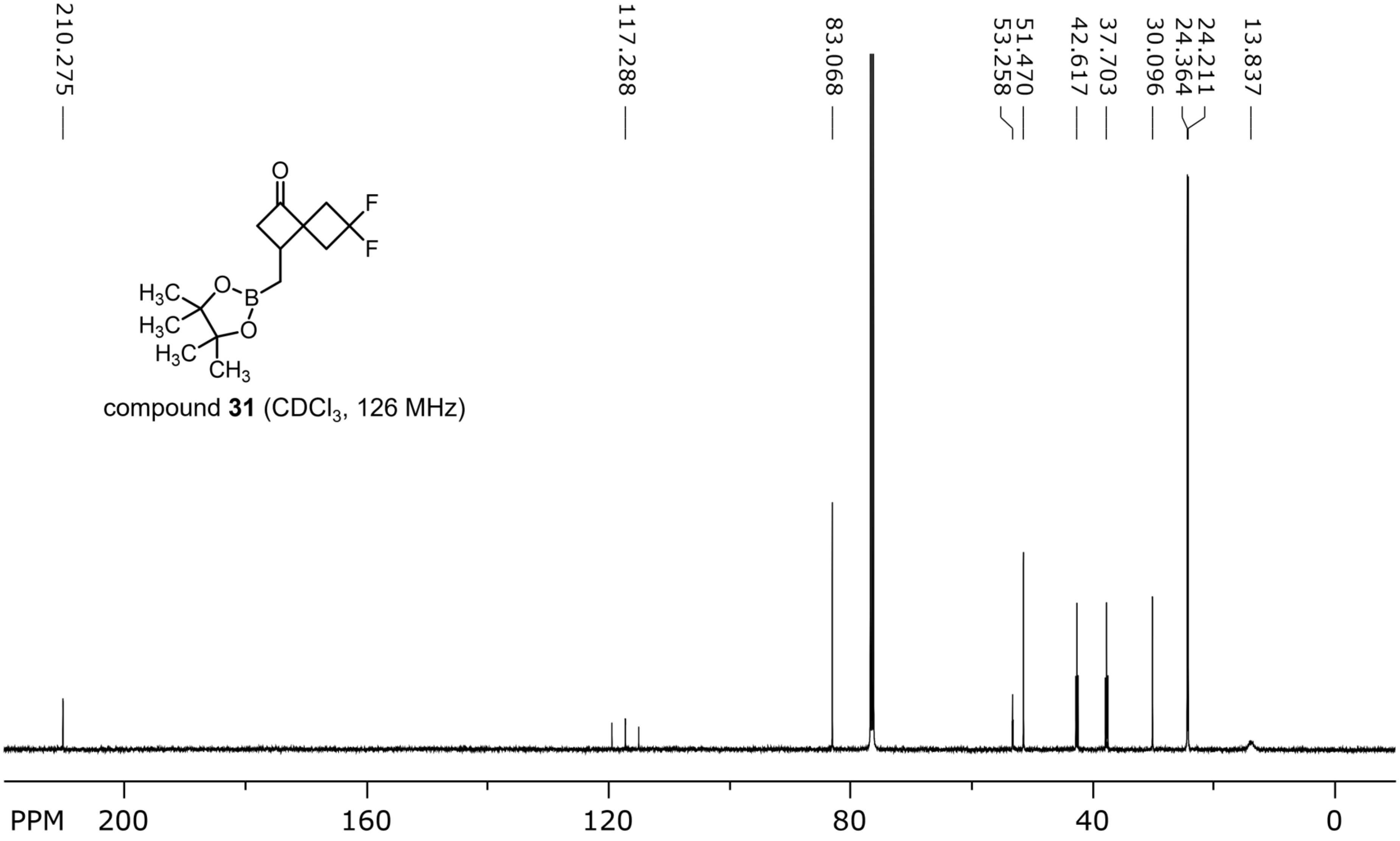

$^{19}F\{^{1}H\}$ *NMR for compound* ***31***

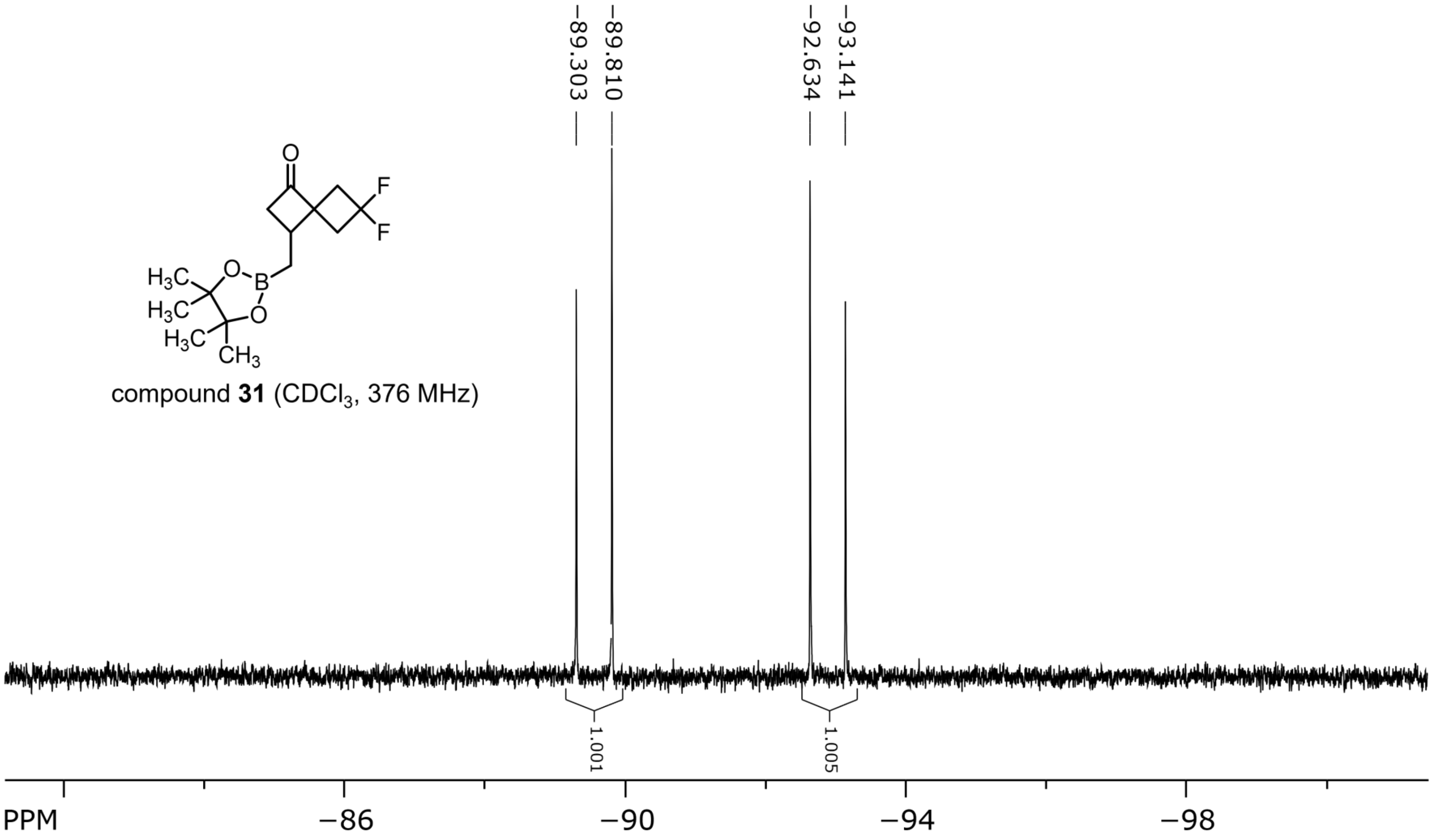

Derivative compounds

*^{1}H NMR for compound **33***

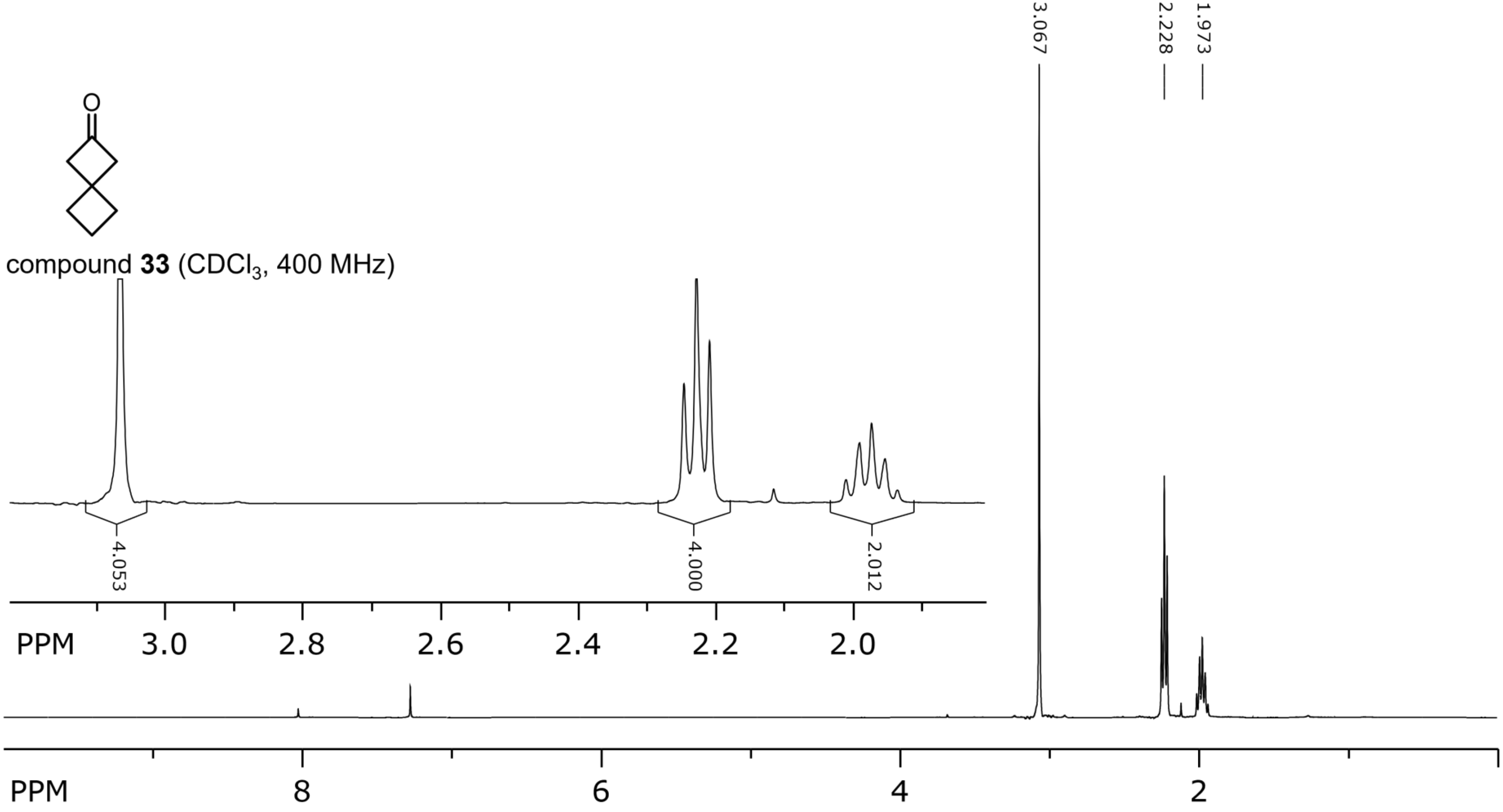

*$^{13}C\{^{1}H\}$ NMR for compound **33***

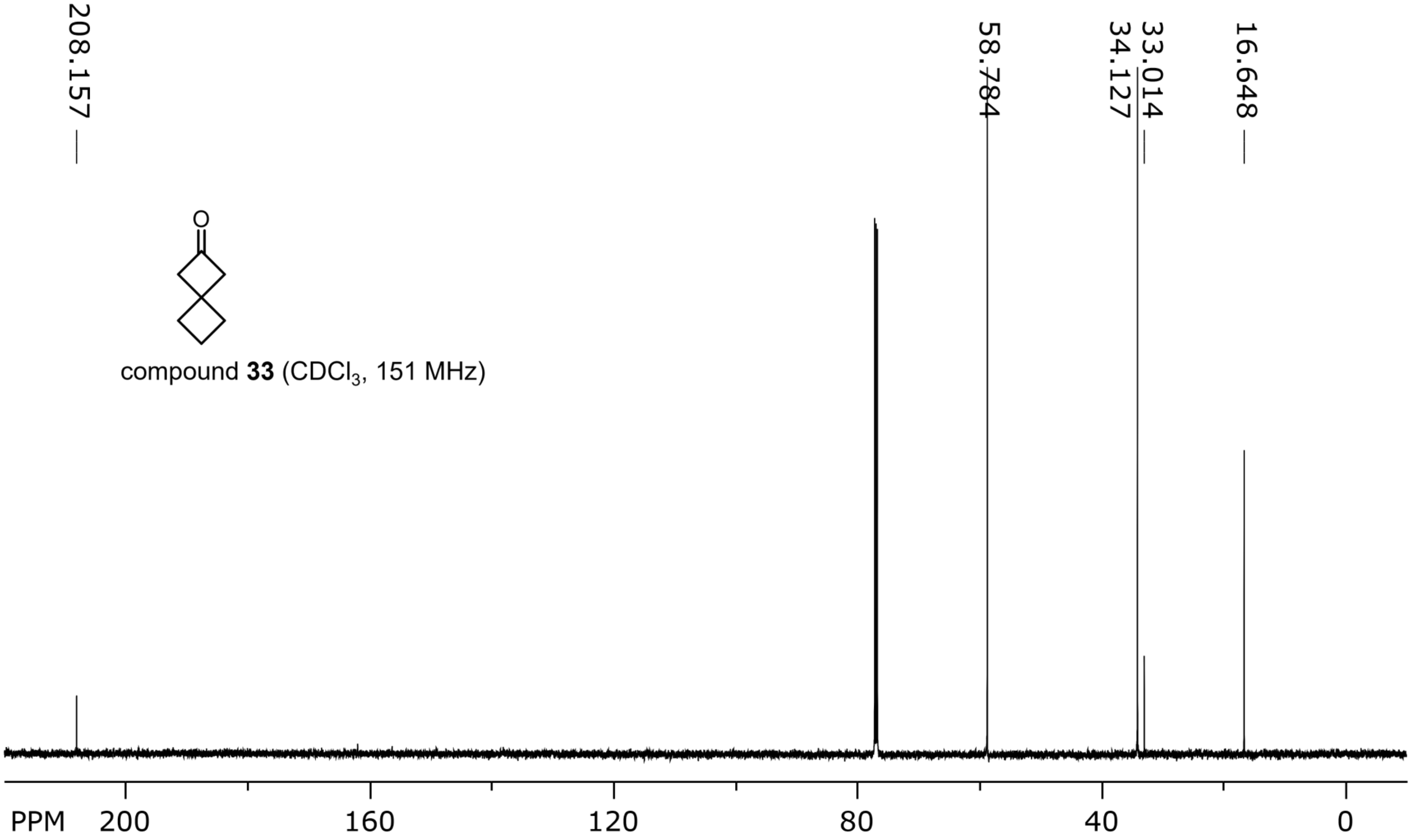

*^{1}H NMR for compound **23***

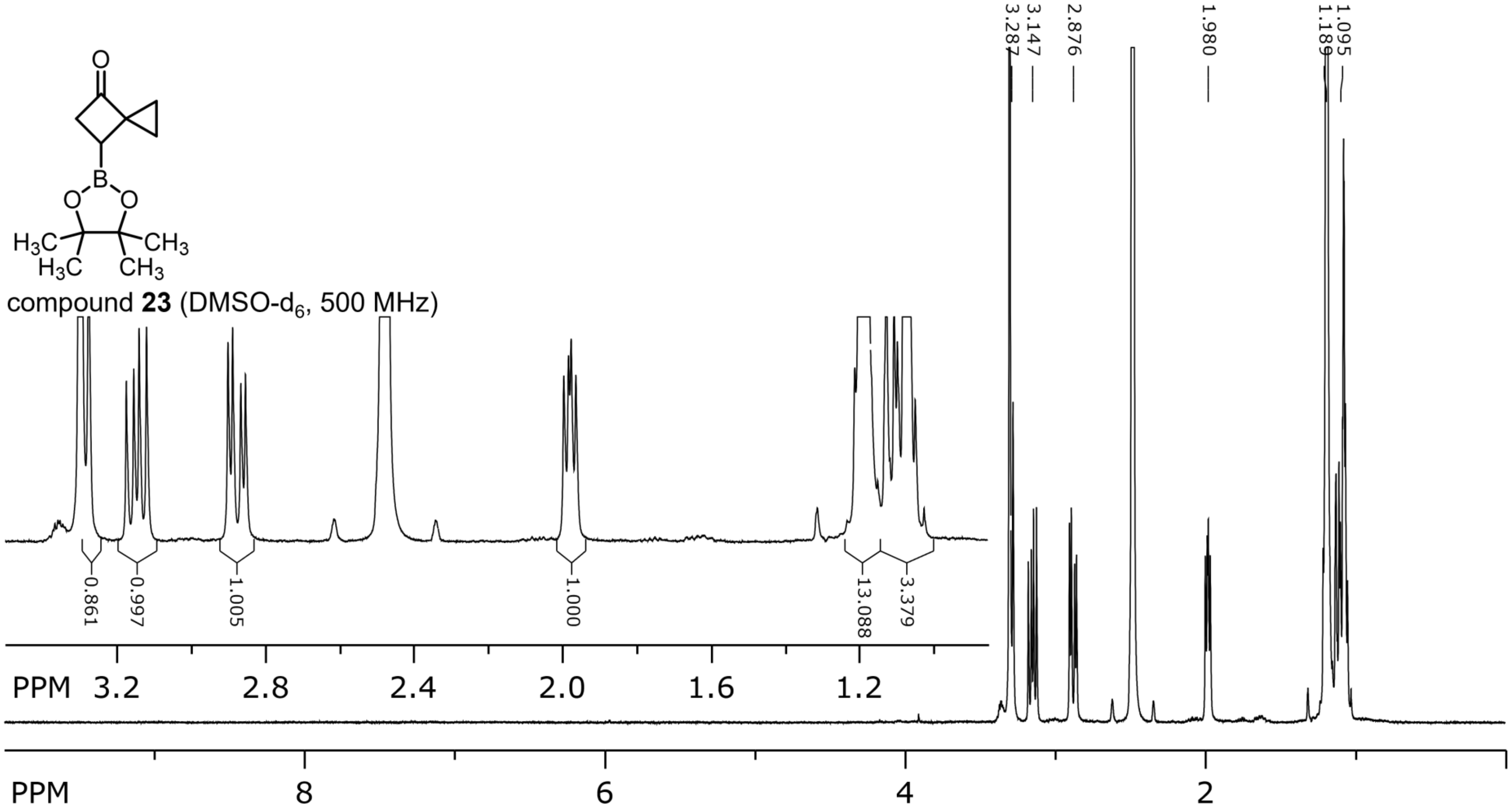

*$^{13}C\{^1H\}$ NMR for compound **23***

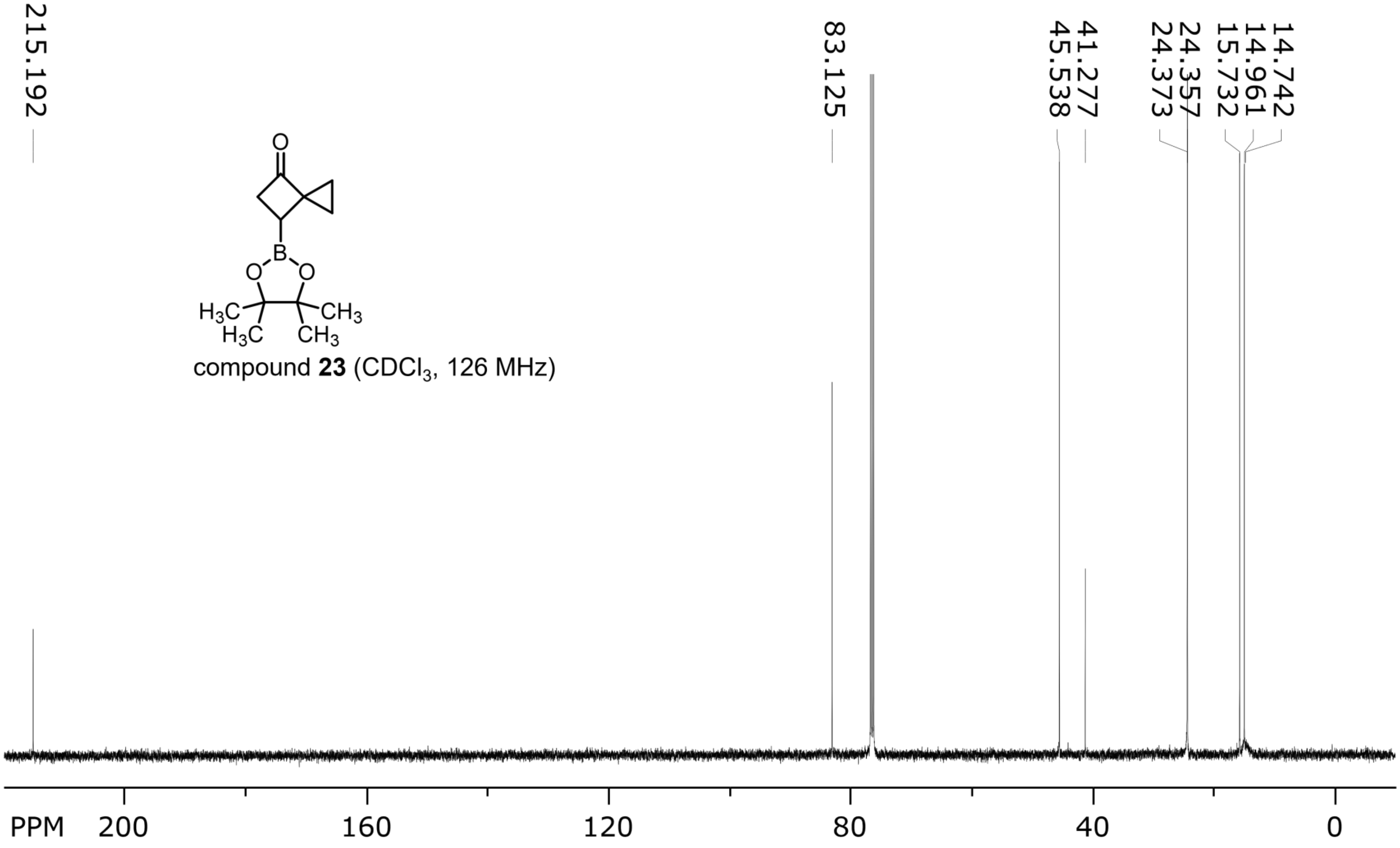

*^{1}H NMR for compound **37**×HCl*

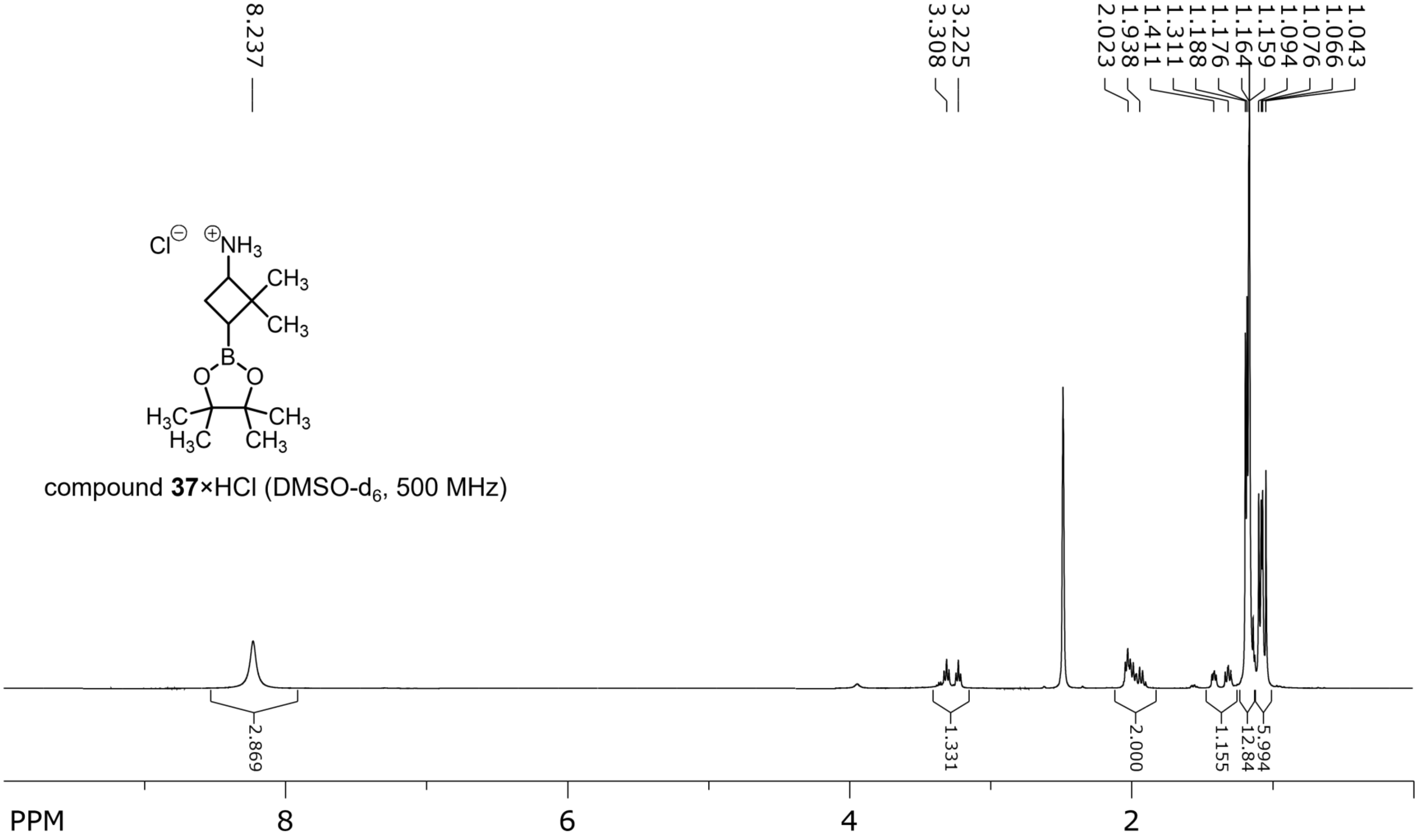

compound **37**×HCl (DMSO-d_6, 500 MHz)

^{13}C{^{1}H} NMR for compound **37**×HCl

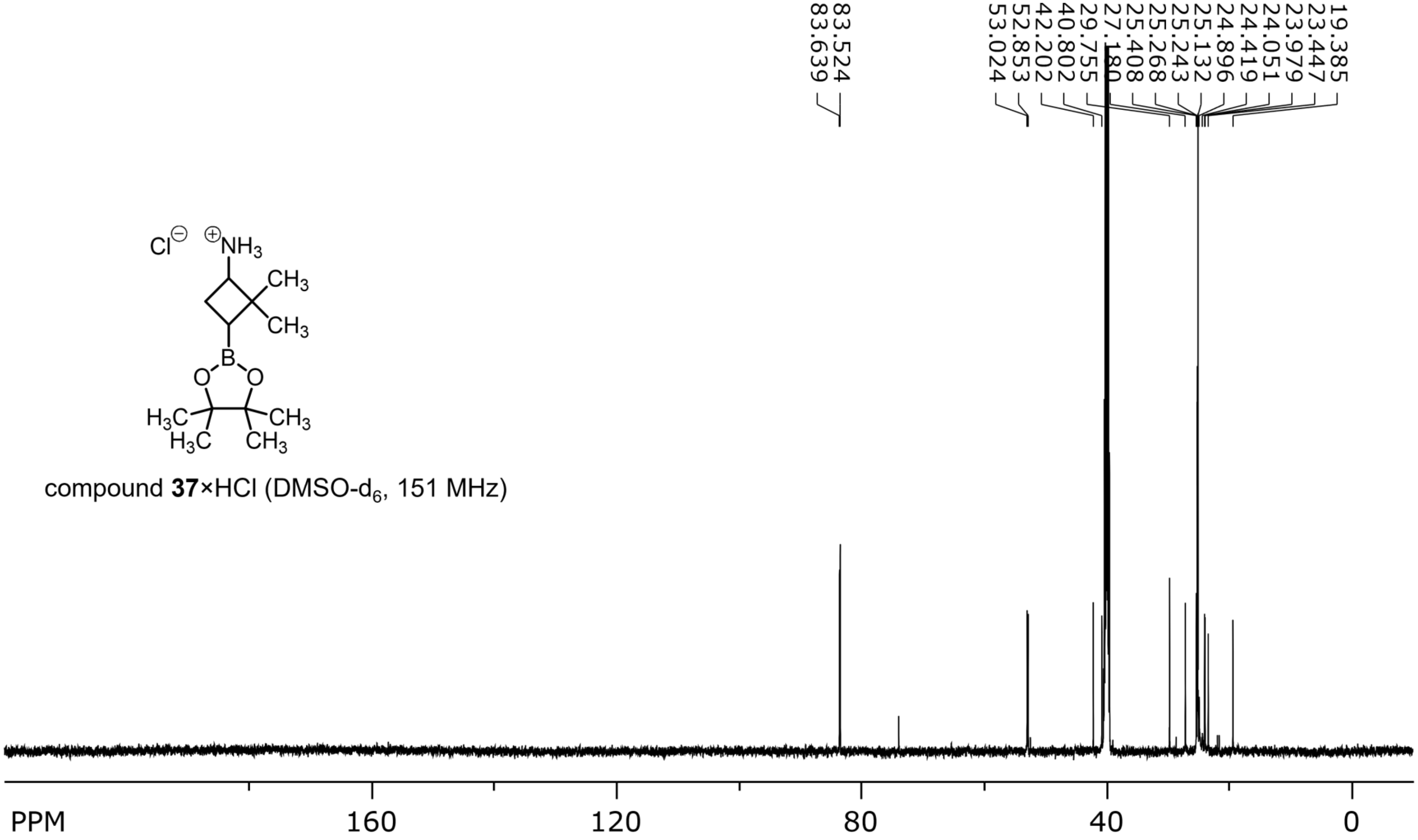

compound **37**×HCl (DMSO-d_6, 151 MHz)

*^{1}H NMR for compound **38***

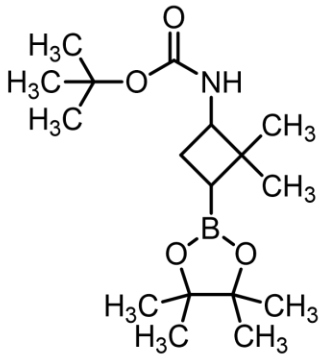

compound **38** (DMSO-d_6, 500 MHz)

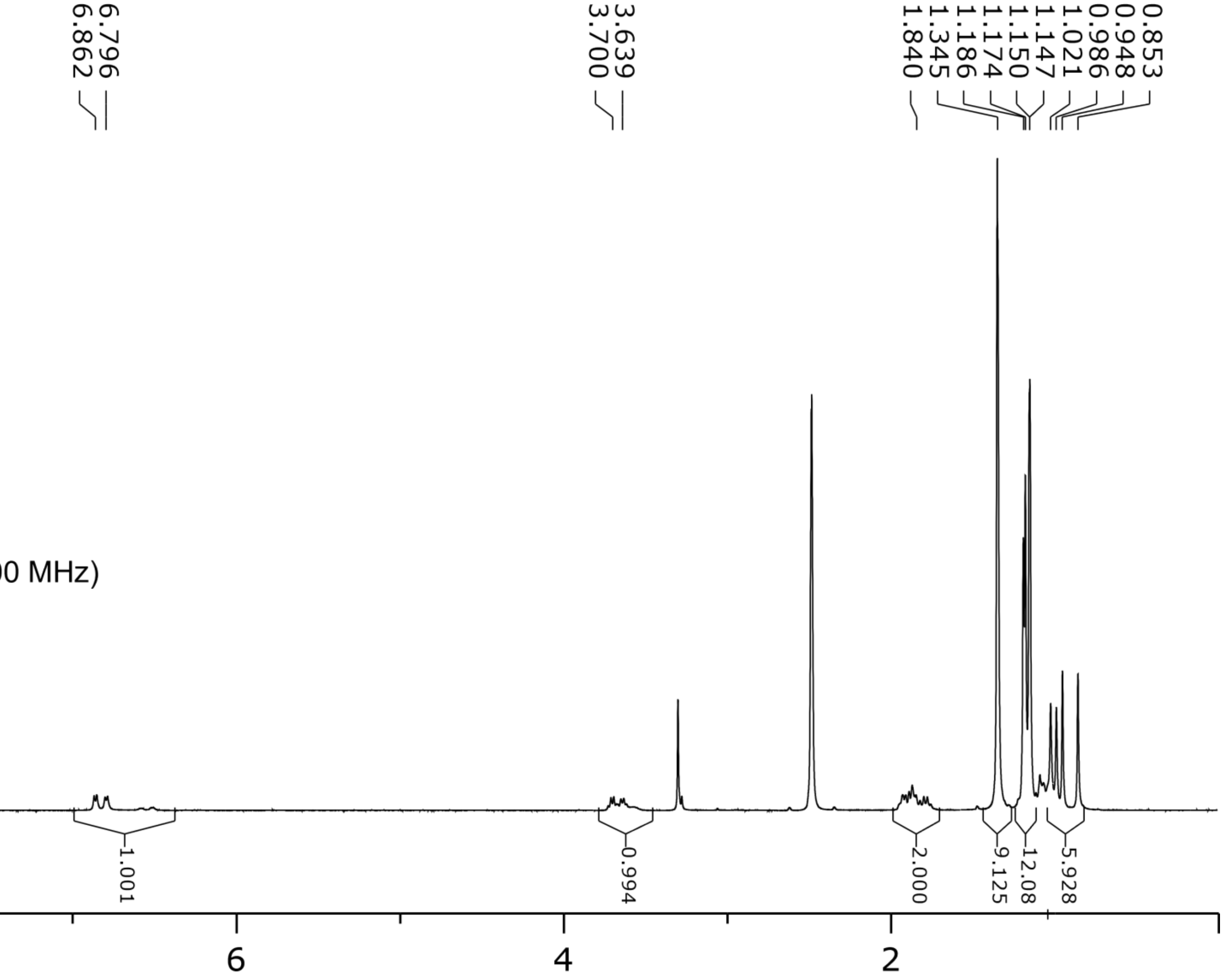

$^{13}C\{^1H\}$ *NMR for compound* ***38***

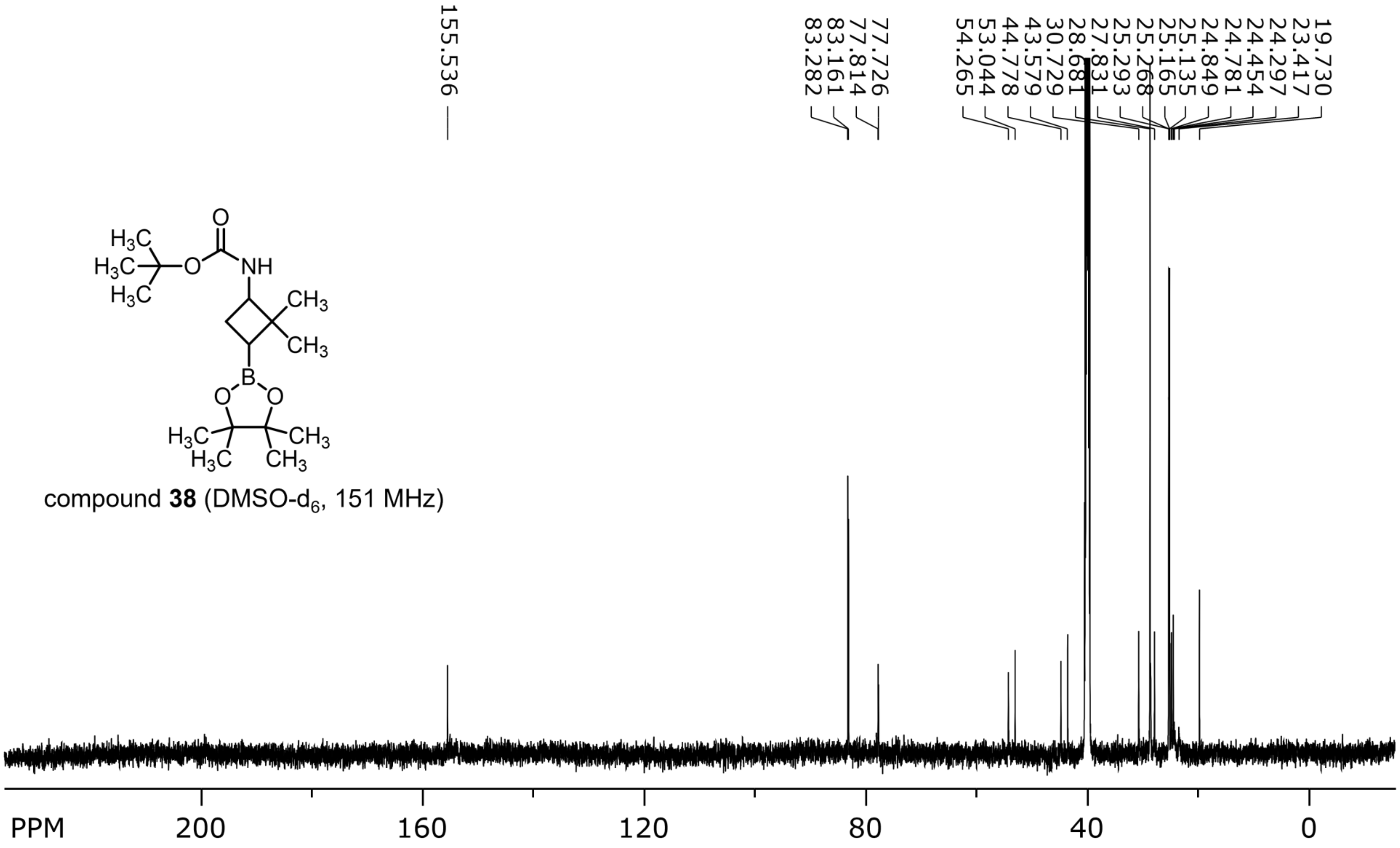

*^{1}H NMR for compound **39**×CF_3CO_2H*

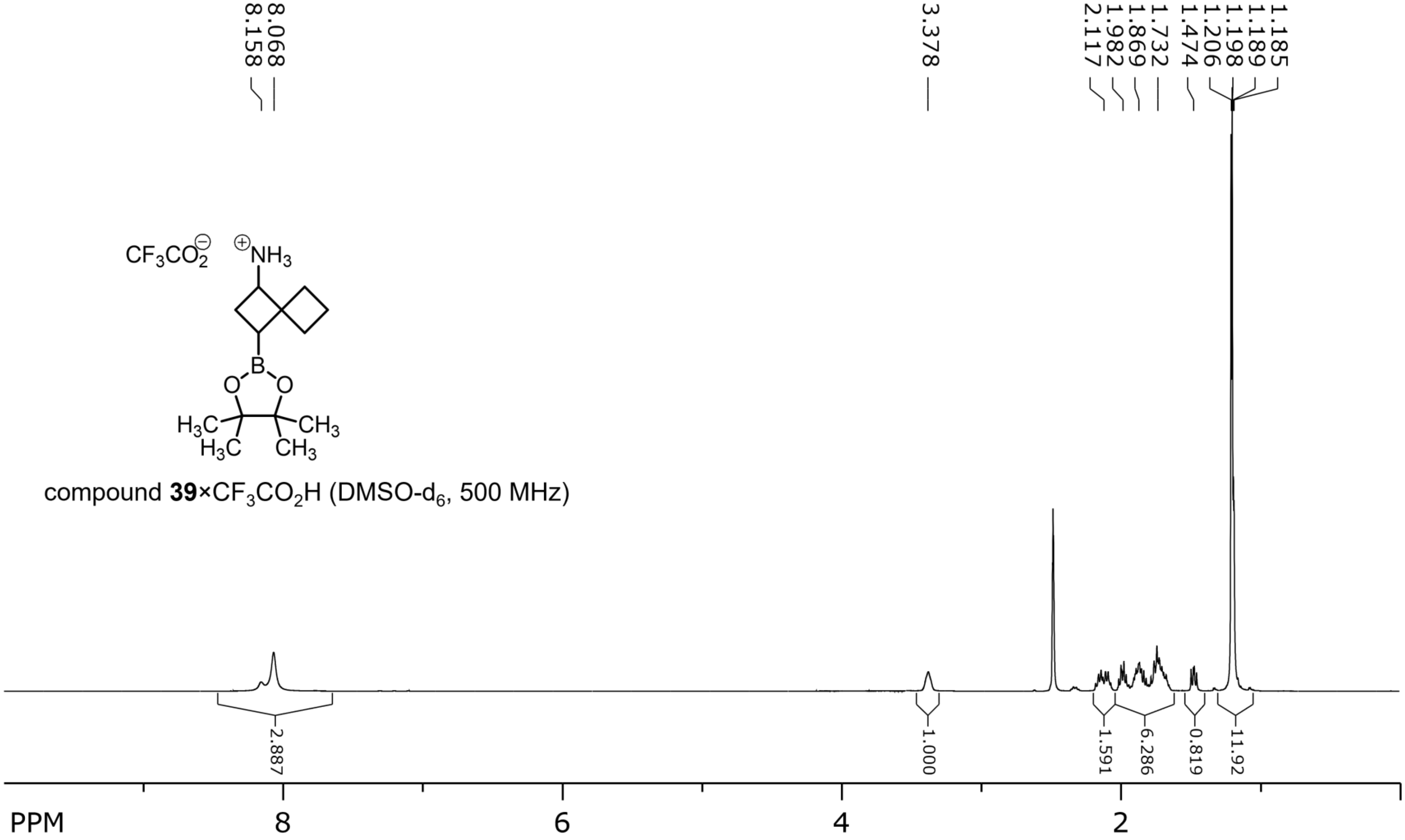

compound **39**×CF_3CO_2H (DMSO-d_6, 500 MHz)

*^{13}C{^{1}H} NMR for compound **39**×CF_3CO_2H*

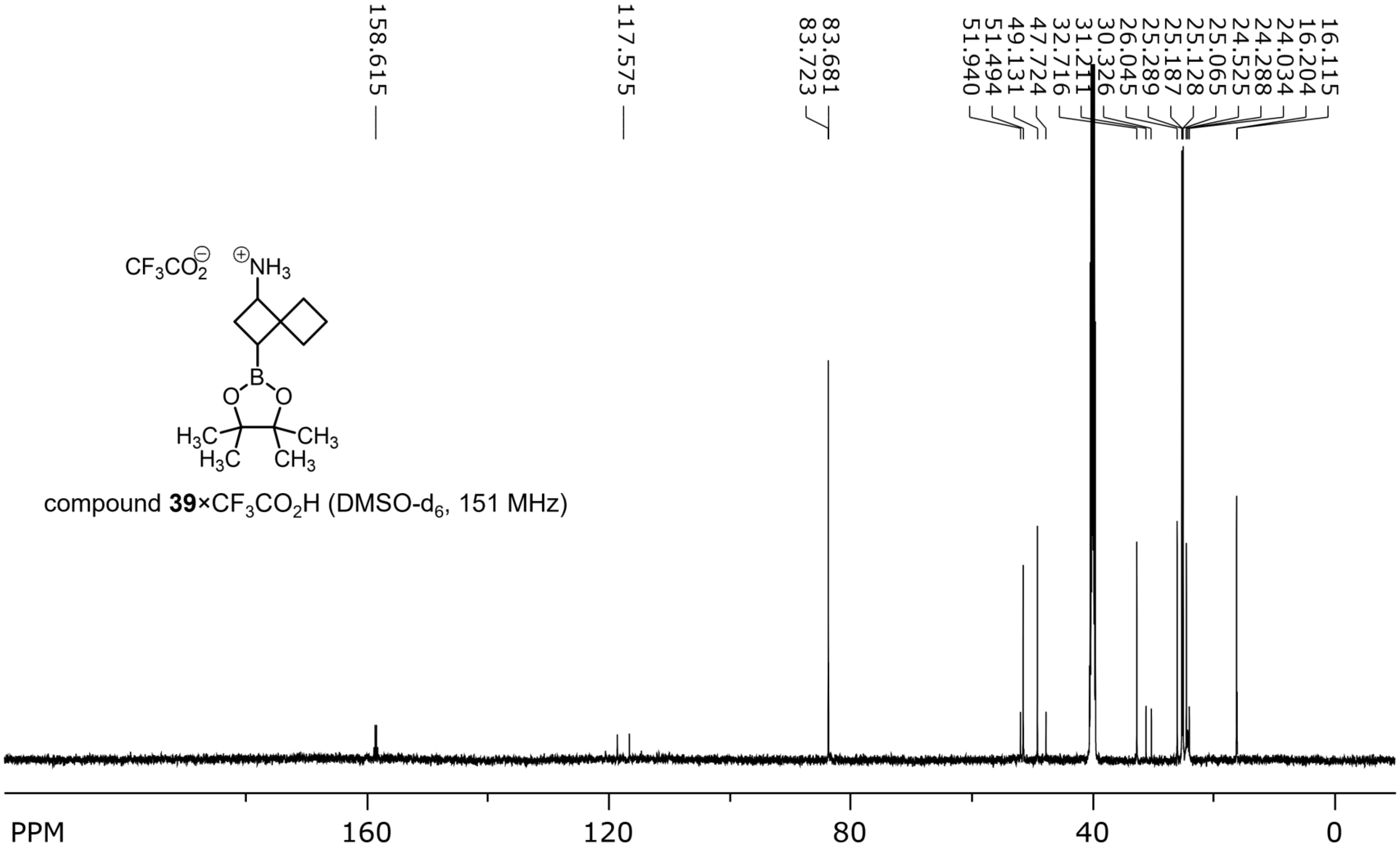

*^{1}H NMR for compound **40***

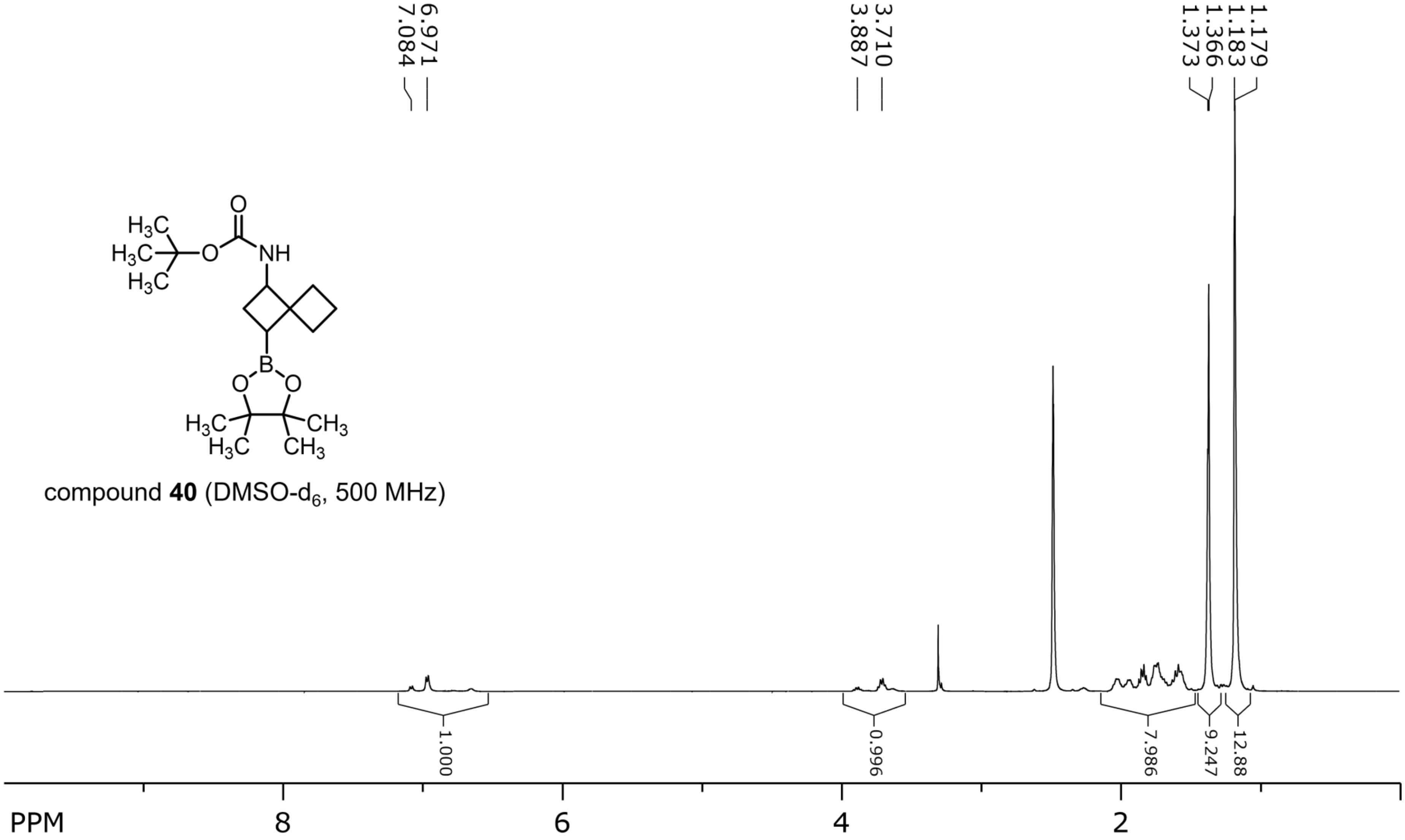

compound **40** (DMSO-d_6, 500 MHz)

^{13}C{^{1}H} NMR for compound ***40***

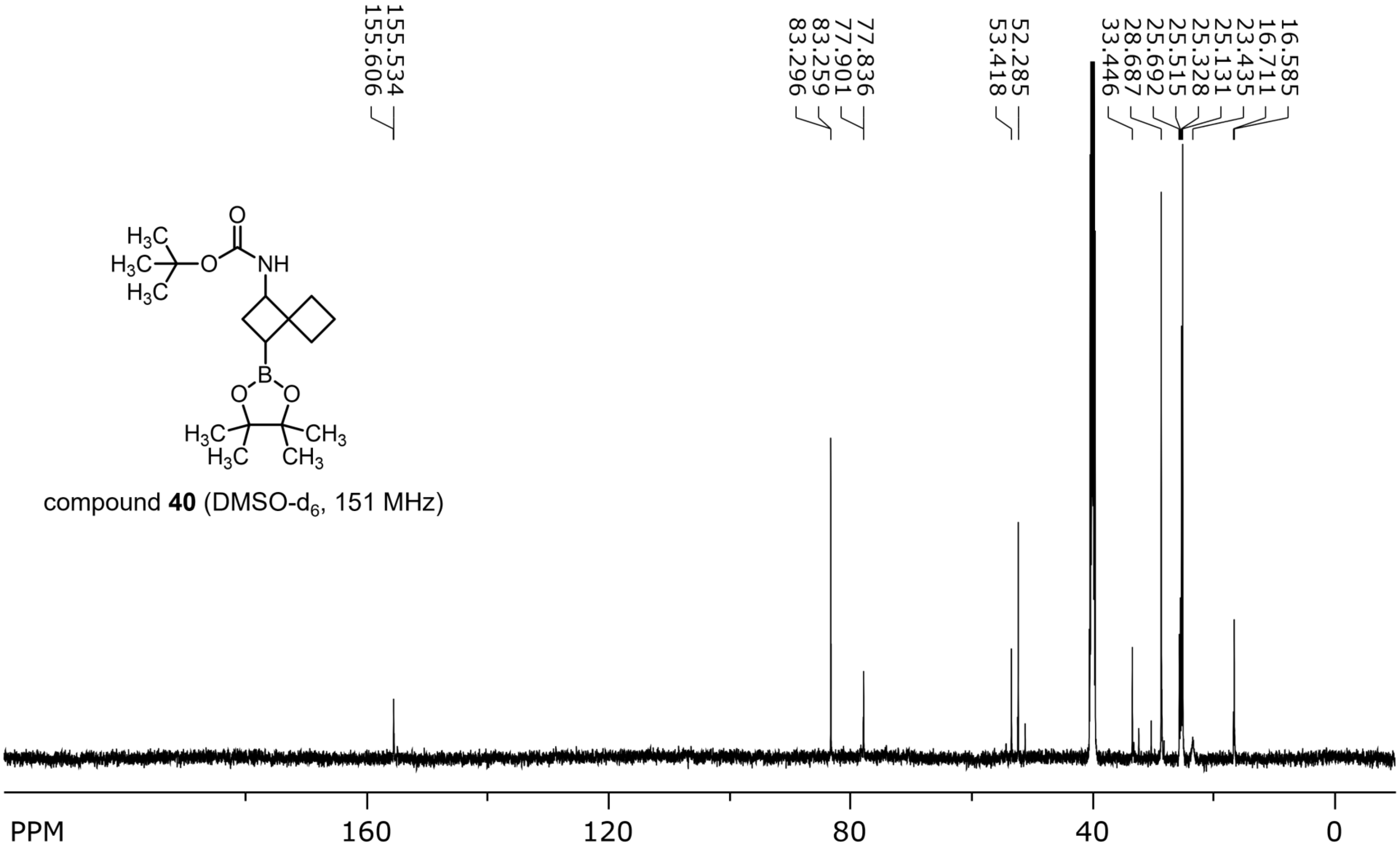

compound **40** (DMSO-d_6, 151 MHz)

^{1}H NMR for compound ***6a***

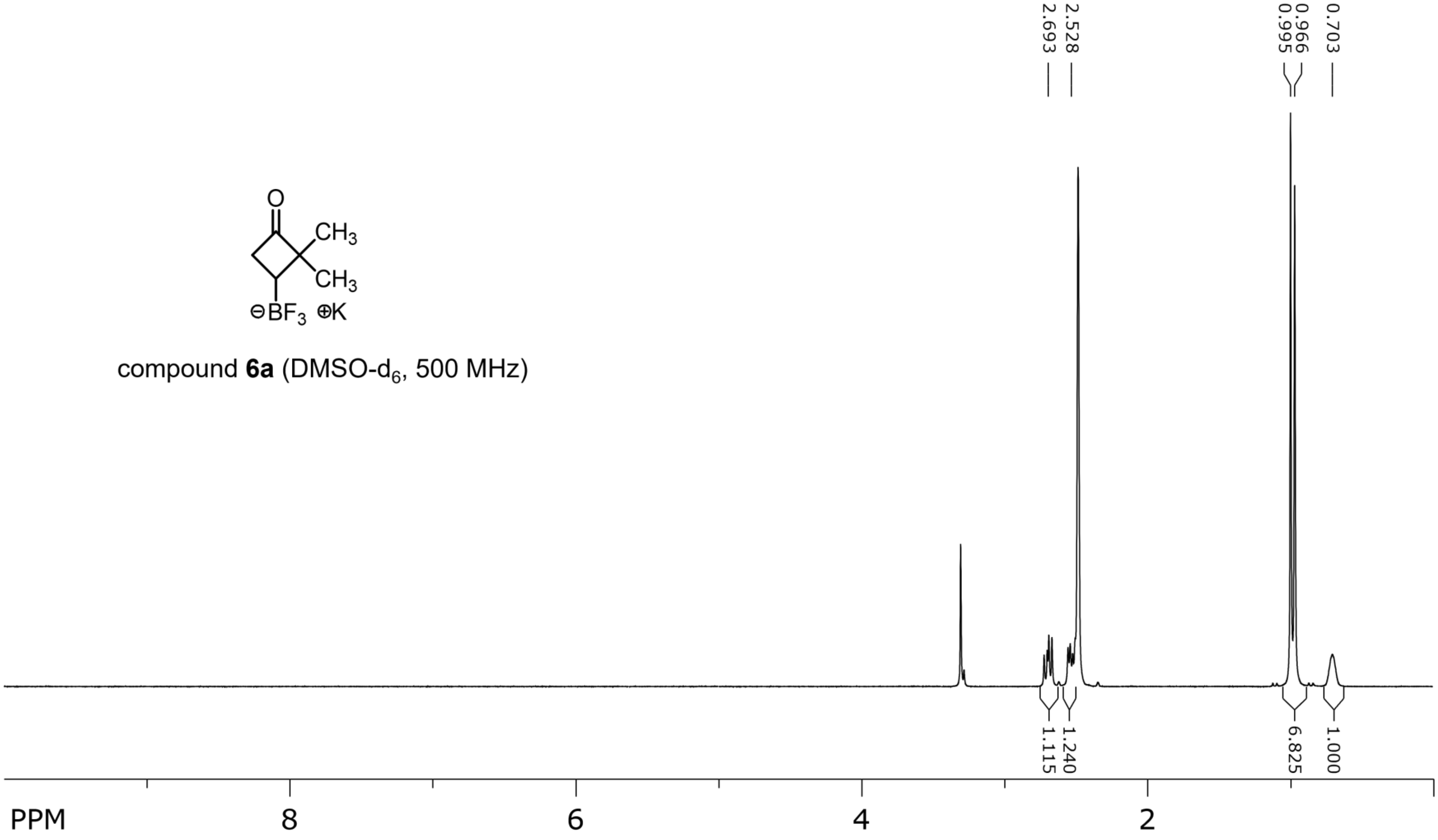

*^{13}C{^{1}H} NMR for compound **6a***

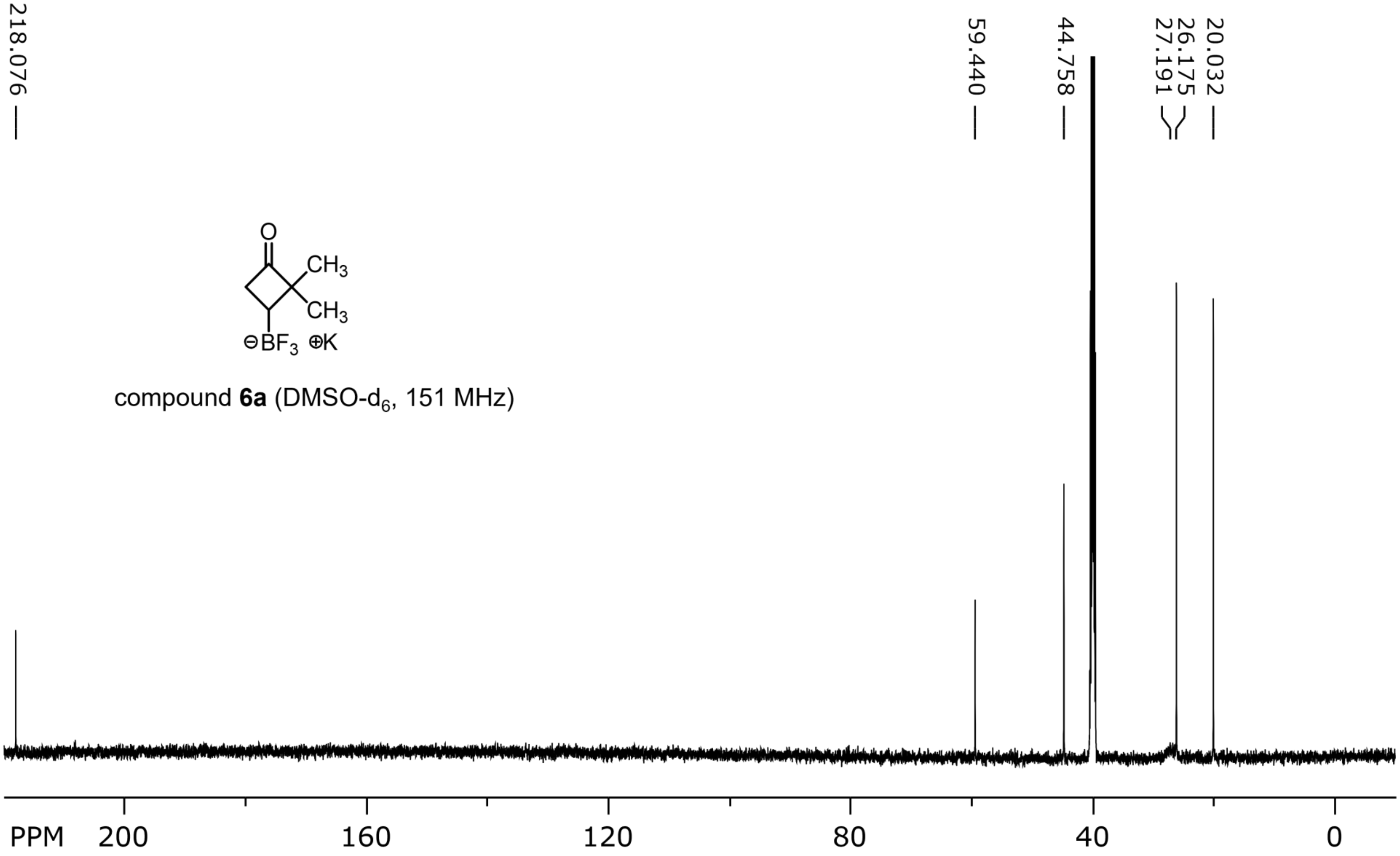

$^{19}F\{^{1}H\}$ *NMR for compound* ***6a***

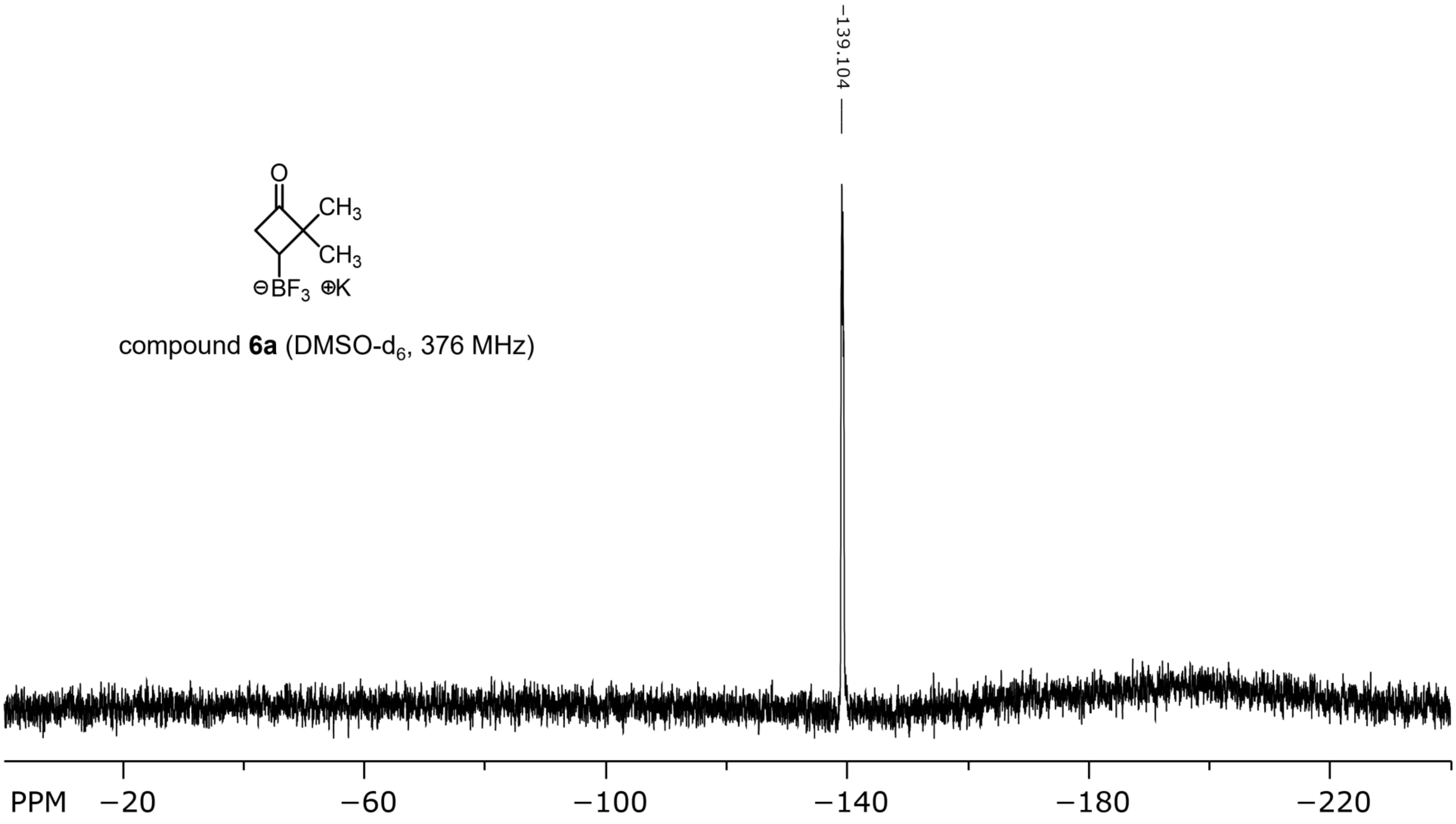

*^{1}H NMR for compound **9a***

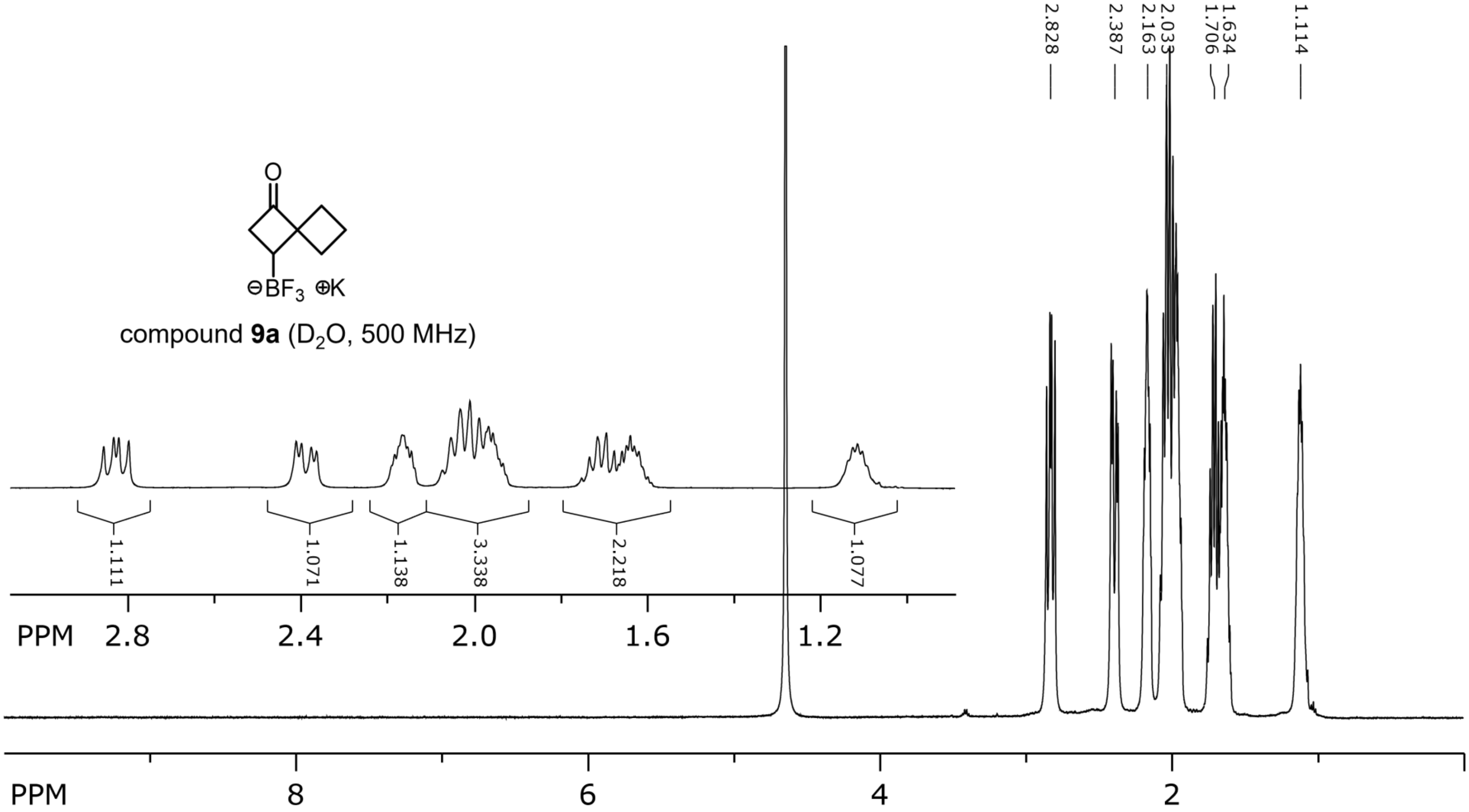

aa

$^{13}C\{^1H\}$ *NMR for compound* ***9a***

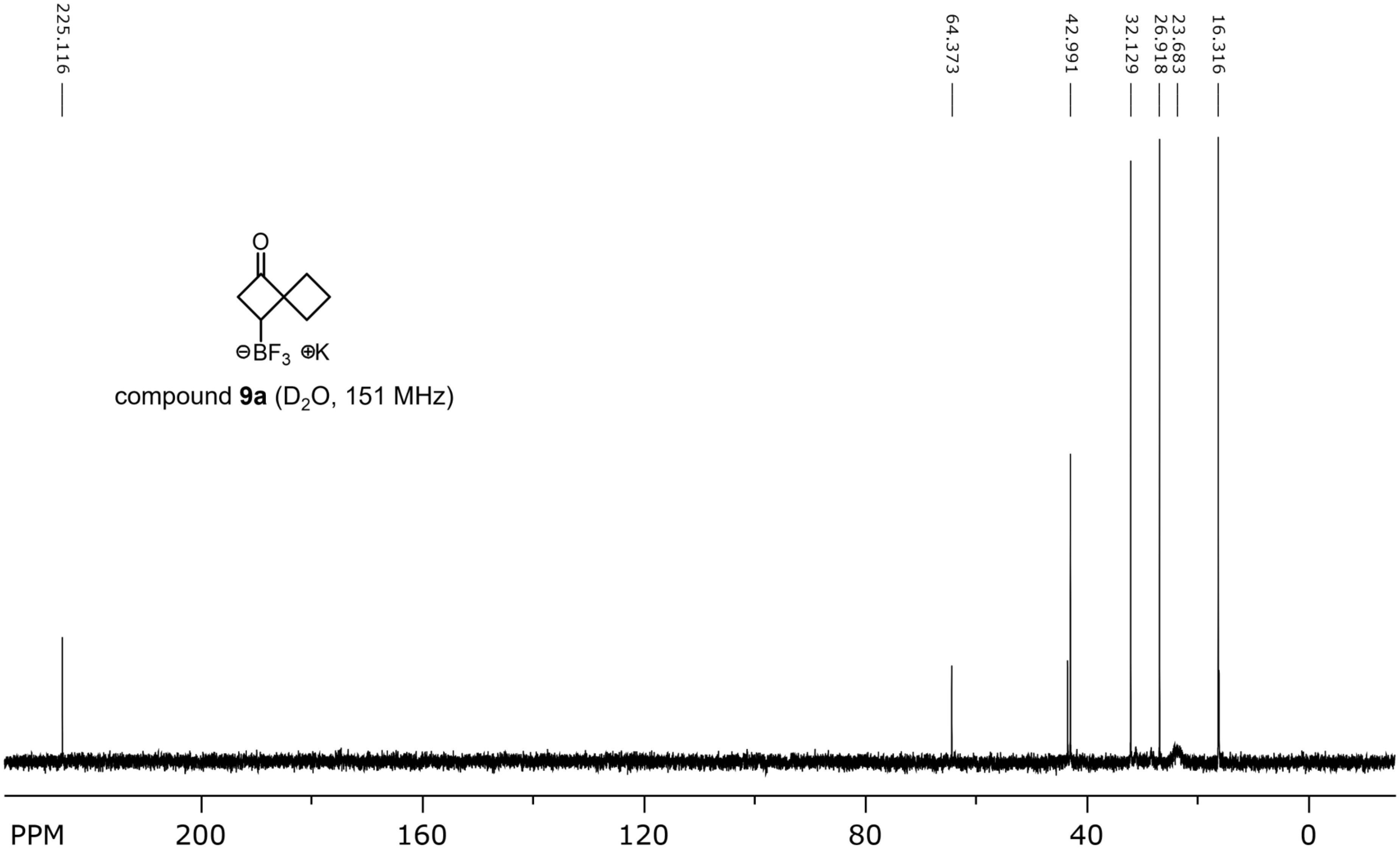

^{19}F{^{1}H} NMR for compound ***9a***

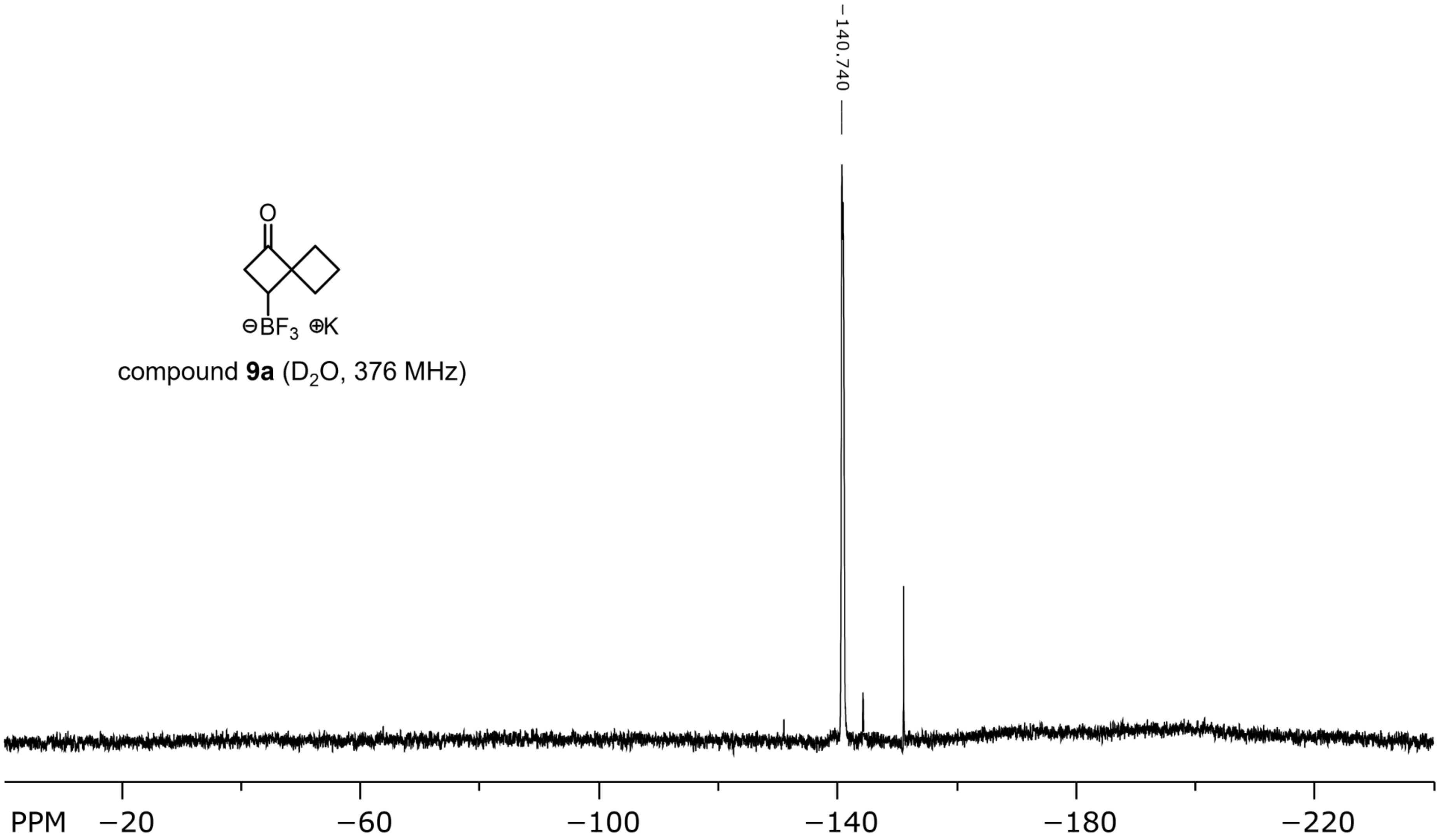

*^{1}H NMR for compound **13a***

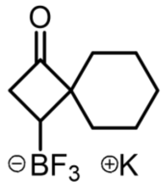

compound **13a** (D_2O, 500 MHz)

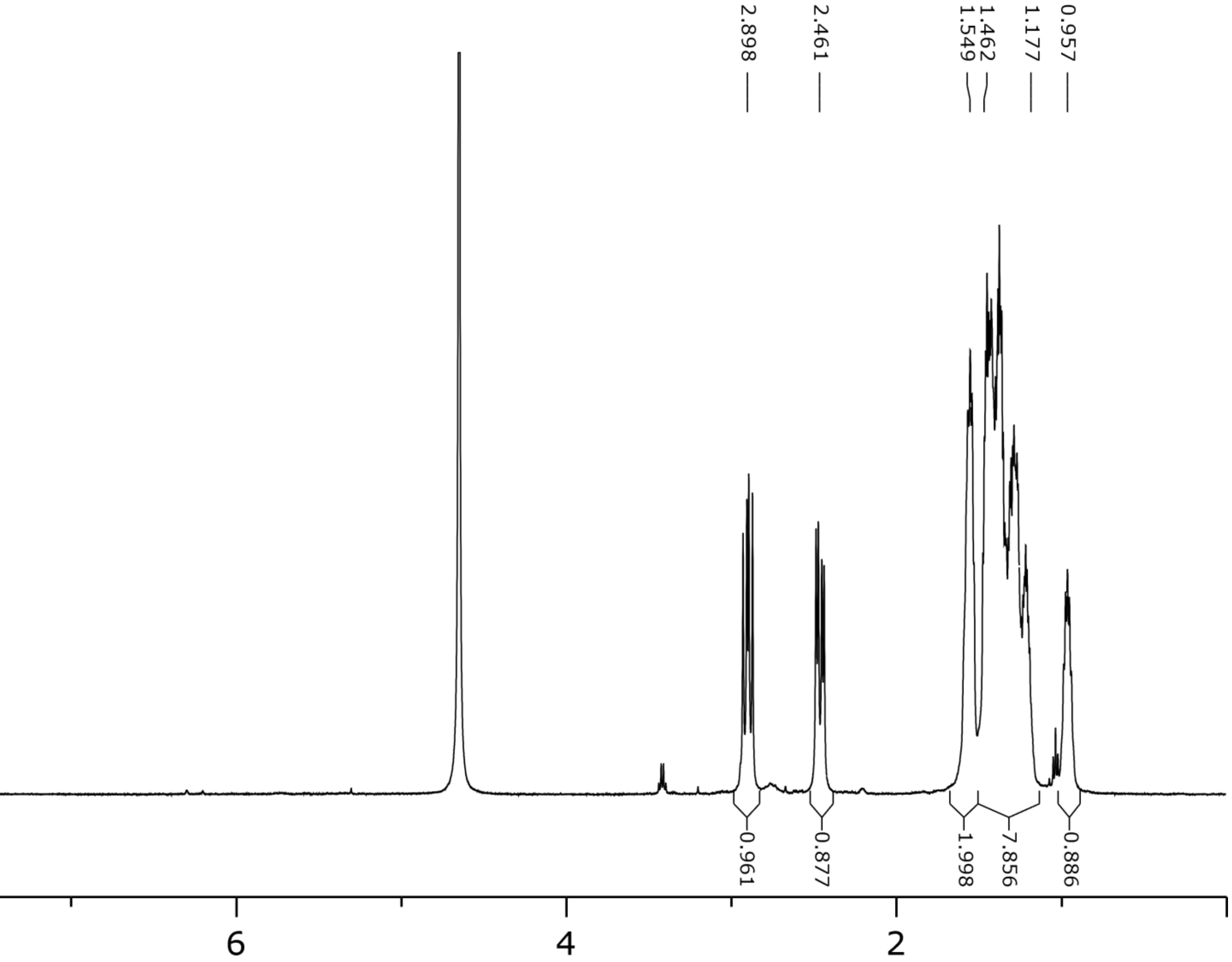

*$^{13}C\{^{1}H\}$ NMR for compound **13a***

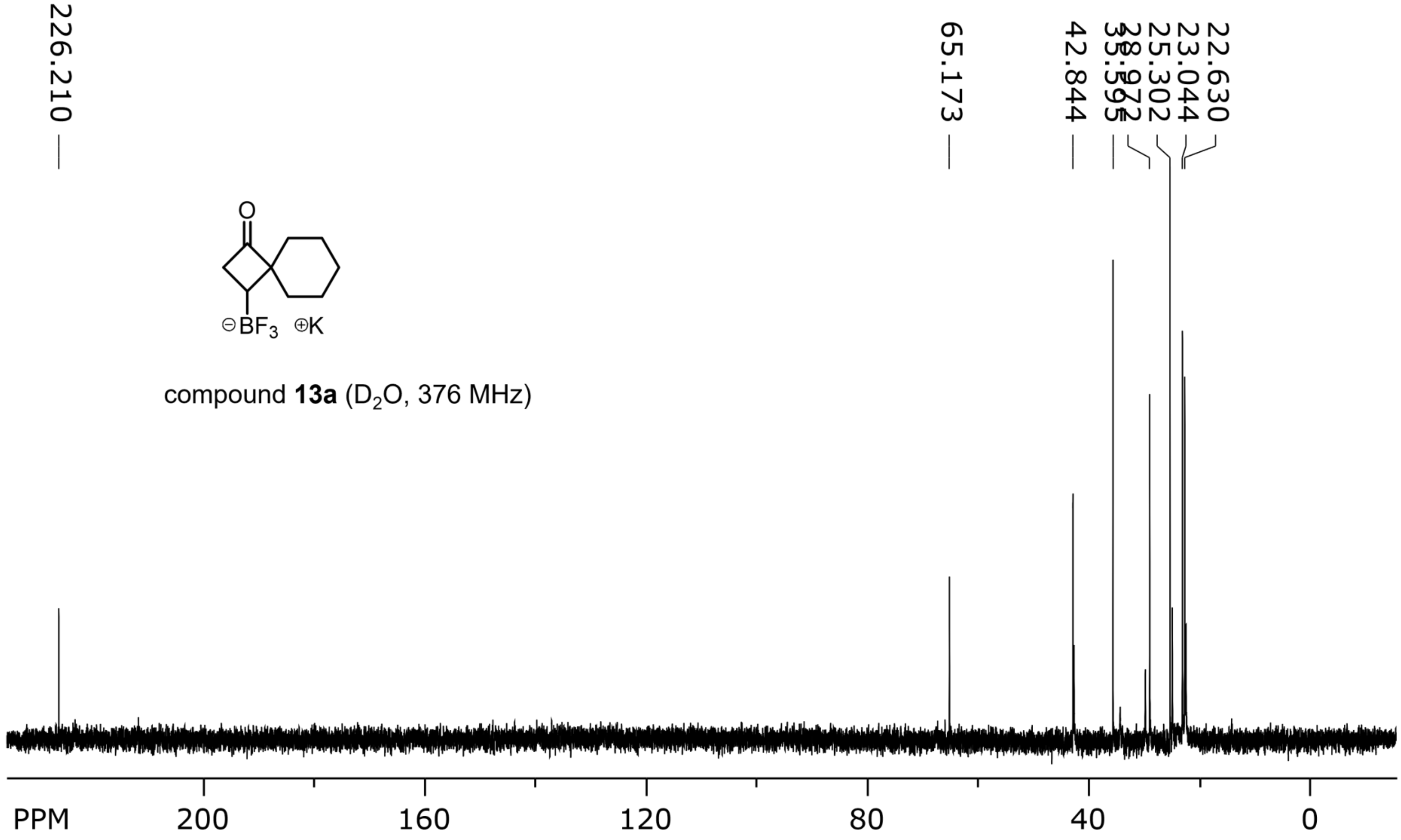

*^{19}F{^{1}H} NMR for compound **13a***

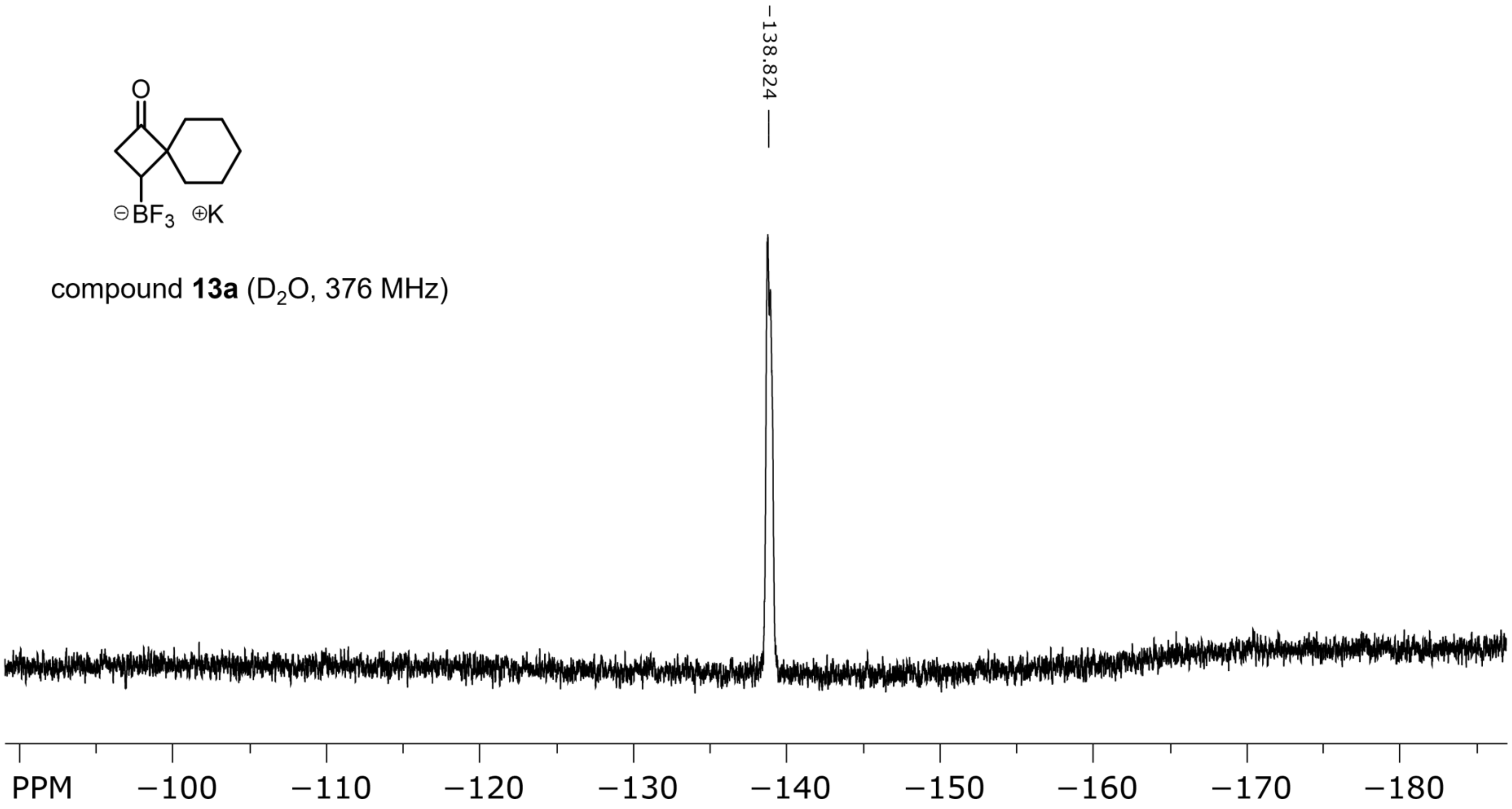

*^{1}H NMR for compound **43***

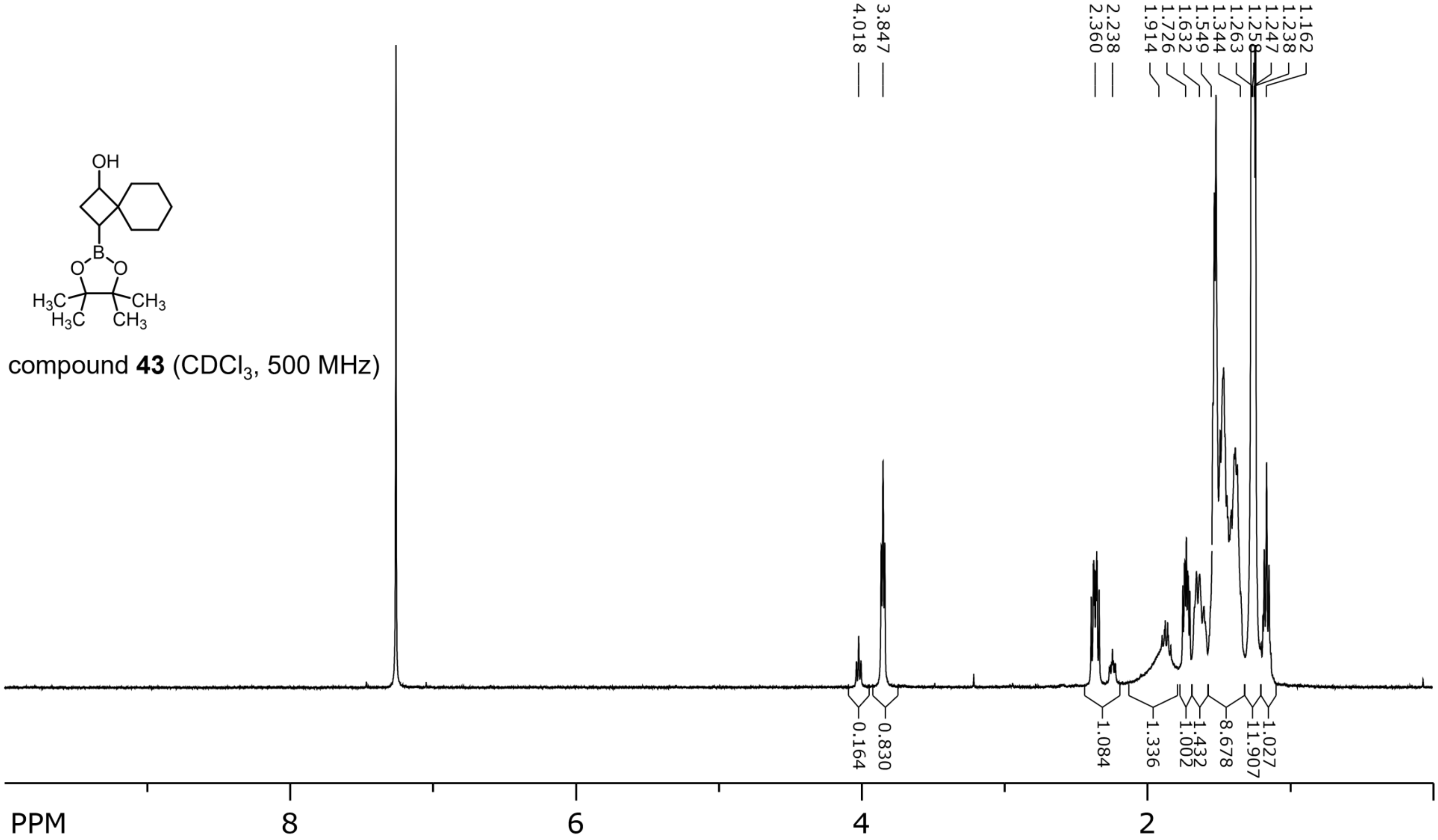

*^{13}C{^{1}H} NMR for compound **43***

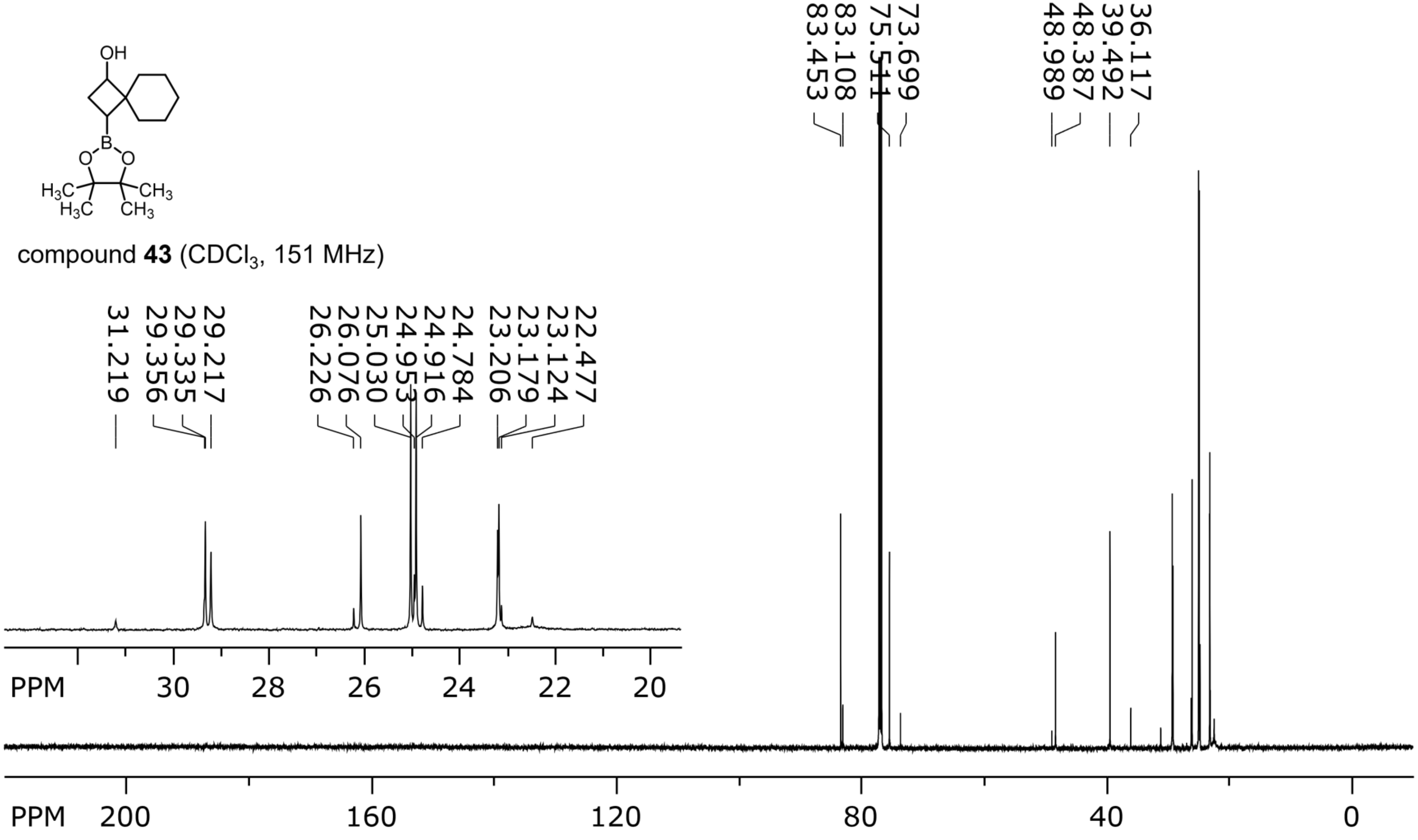

^{1}H NMR for compound ***44***

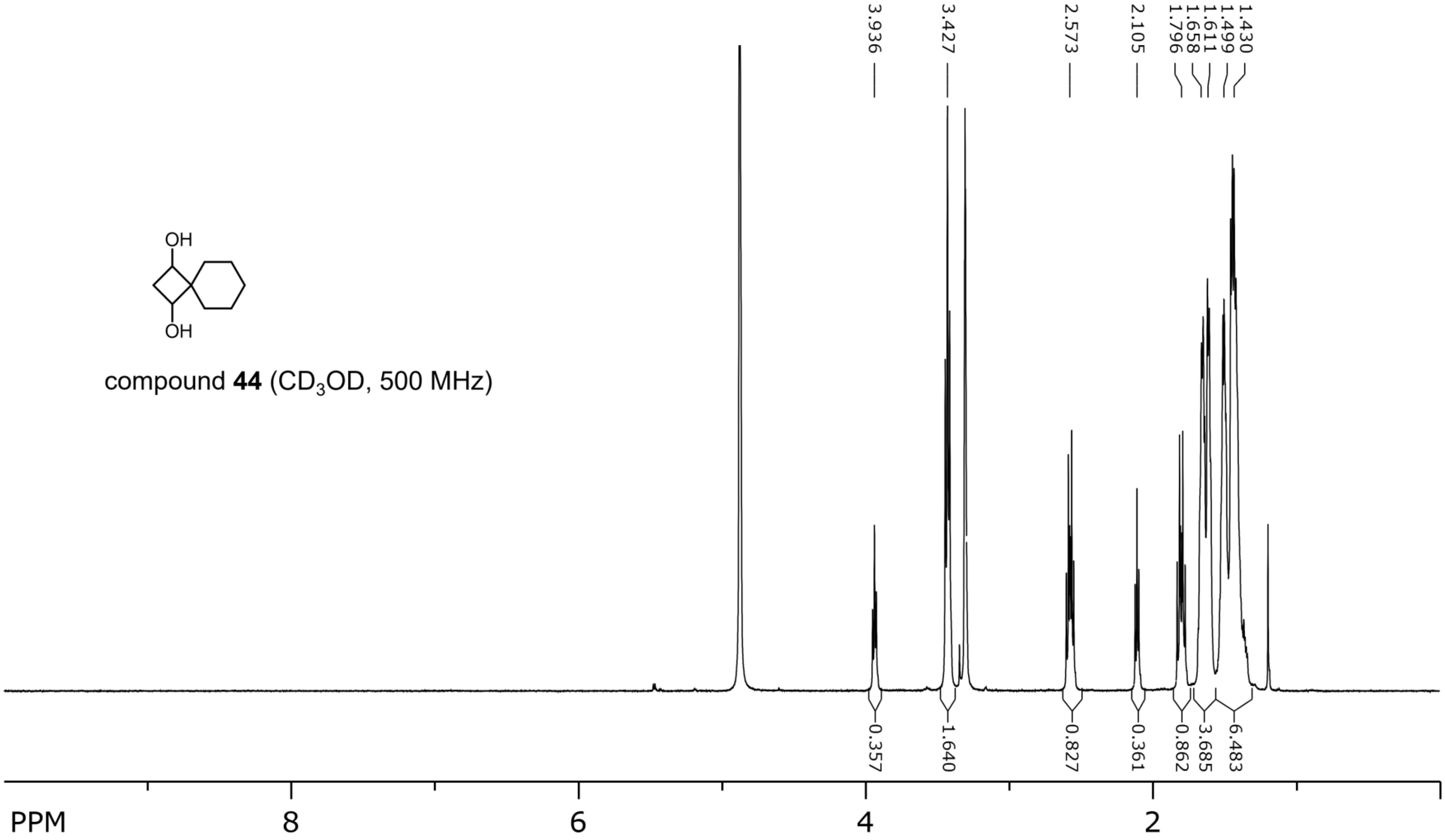

$^{13}C\{^1H\}$ *NMR for compound* ***44***

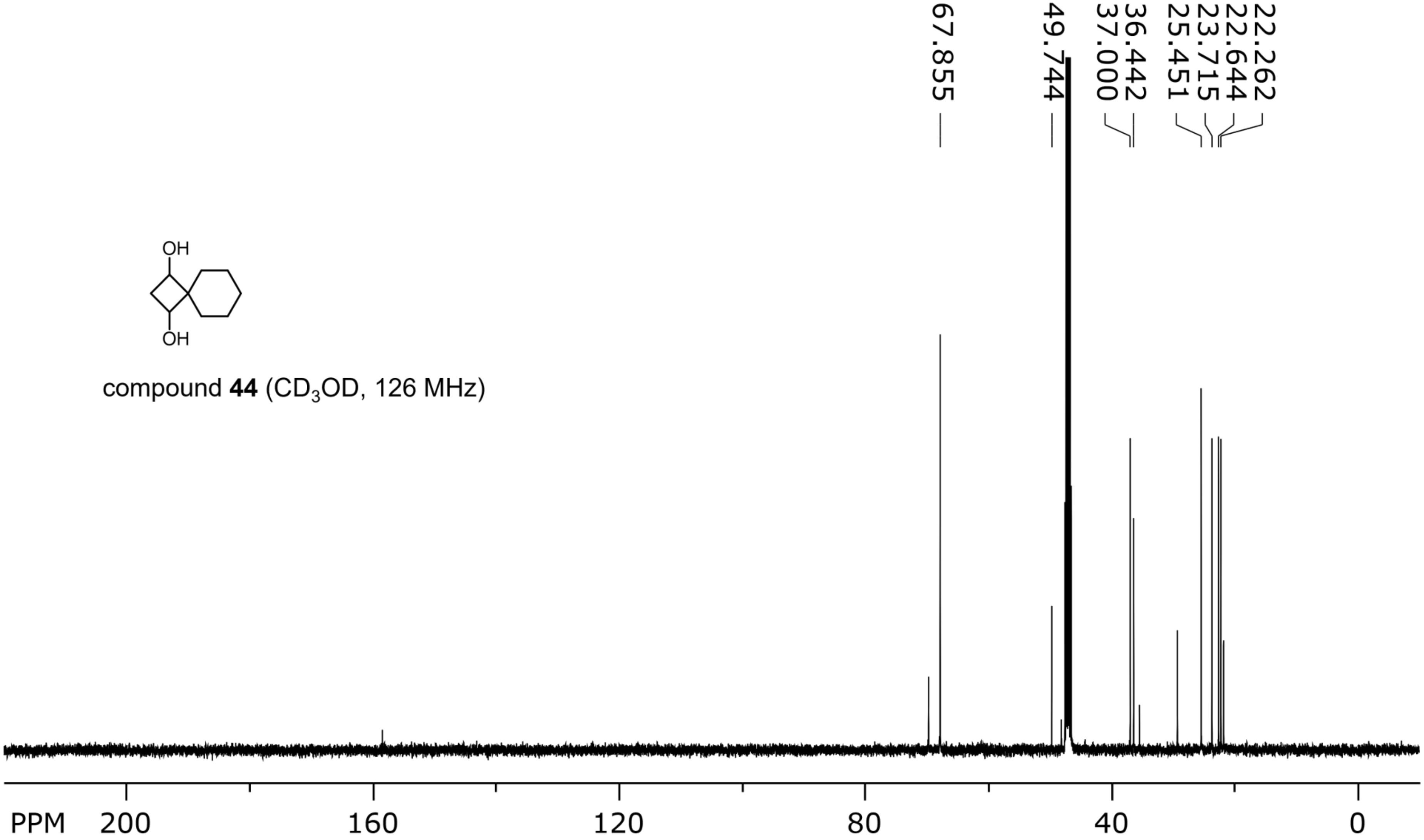

compound **44** (CD_3OD, 126 MHz)

6. Supporting references

[S1] Zhang, Y.-L.; Guo, R.-T.; He, J.-H.; Wang, X.-C. Catalytic Intermolecular Coupling of Rhodacyclopentanones with Alcohols Enabled by Dual Directing Strategy. *Org. Lett.* **2019**, *21*, 4239-4244.
https://doi.org/10.1021/acs.orglett.9b01420

[S2] Huang, Y.; Zhang, J. Potassium *tert*-Butoxide Facilitated Amination of Carboxylic Acids with *N*,*N*-Dimethylformamide. *Synthesis* **2022**, *54*, 3595-3604.
https://doi.org/10.1055/a-1817-1965

[S3] Kobeissi, M.; Cherry, K.; Jomaa, W. Bromination of Enamines from Tertiary Amides Using the Petasis Reagent: A Convenient One-Pot Regioselective Route to Bromomethyl Ketones. *Synth. Commun.* **2013**, *43*, 2955-2065.
https://doi.org/10.1080/00397911.2013.765484

[S4] Chen, J.; Lim, J. W.; Ong, D. Y.; Chiba, S. Iterative addition of carbon nucleophiles to *N*,*N*-dialkyl carboxamides for synthesis of α-tertiary amines. *Chem. Sci.* **2022**, *13*, 99-104.
https://doi.org/10.1039/D1SC05876B

[S5] O'Brien, J. M.; Kingsbury, J. S. A Practical Synthesis of 3-Acyl Cyclobutanones by [2+2] Annulation. Mechanism and Utility of the Zn(II)-Catalyzed Condensation of α-Chloroenamines with Electron-Deficient Alkenes. *J. Org. Chem.* **2011**, *76*, 1662-1672.
https://doi.org/10.1021/jo102257k

[S6] Bouillon, J.-P.; Maliverney, C.; Merényi, R.; Viehe, H. G. Trifluoromethylation of aliphatic halogen compounds. *J. Chem. Soc., Perkin Trans. 1* **1991**, 2147-2149.
https://doi.org/10.1039/P19910002147

[S7] Mantani, T.; Shiomi, K.; Konno, T.; Ishihara, T.; Yamanaka, H. A convenient preparation of 3,3,3-trifluoro-1-propynylamines and their Lewis acid catalyzed reaction with carbonyl compounds leading to (*Z*)-α-(trifluoromethyl)-α,β-unsaturated amides. *J. Org. Chem.* **2001**, *66*, 3442-3448.
https://doi.org/10.1021/jo001760v

[S8] Yardley, J. P.; Husbands, G. E. M.; Stack, G.; Butch, J.; Bicksler, J.; Moyer, J. A.; Muth, E. A.; Andree, T.; Fletcher, H., III; James, M. N. G.; Sielecki, A. R. 2-Phenyl-2-(1-hydroxycycloalkyl)ethylamine derivatives: synthesis and antidepressant activity. *J. Med. Chem.* **1990**, *33*, 2899-2905.
https://doi.org/10.1021/jm00172a035

[S9] Tinnis, F.; Lundberg, H.; Adolfsson, H. Direct Catalytic Formation of Primary and Tertiary Amides from Non-Activated Carboxylic Acids, Employing Carbamates as Amine Source. *Adv. Synth. Catal.* **2012**, *354*, 2531-2536.
https://doi.org/10.1002/adsc.201200436

[S10] Clement, H. A.; Boghi, M.; McDonald, R. M.; Bernier, L.; Coe, J. W.; Farrell, W.; Helal, C. J.; Reese, M. R.; Sach, N. W.; Lee, J. C.; Hall, D. G. High-throughput ligand screening enables the enantioselective conjugate borylation of cyclobutenones to access synthetically versatile tertiary cyclobutylboronates. *Angew. Chem. Int. Ed.*, **2019**, *58*, 18405-18409.
https://doi.org/10.1002/anie.201909308

[S11] Cui, M.; Zhao, Z.-Y.; Oestreich, M. Boosting the Enantioselectivity of Conjugate Borylation of α,β-Disubstituted Cyclobutenones with Monooxides of Chiral C_2-Symmetric Bis(phosphine) Ligands. *Chem. Eur. J.* **2022**, *28*, e202202163.
https://doi.org/10.1002/chem.202202163
